# An Enantiospecific Synthesis of Isoneoamphilectane Confirms Its Strained Tricyclic Structure

**DOI:** 10.1021/jacs.2c13137

**Published:** 2023-02-02

**Authors:** Natalie C. Dwulet, Zeinab Chahine, Karine G. Le Roch, Christopher D. Vanderwal

**Affiliations:** † Department of Chemistry, University of California, Irvine, California 92697-2025, United States; ‡ Institute for Integrative Genome Biology, Center for Infectious Disease and Vector Research, 900 University Avenue, Department of Molecular, Cell, and Systems Biology, University of California, Riverside, California 92521, United States; § Department of Pharmaceutical Sciences, 101 Theory, University of California, Irvine, California 92697, United States

## Abstract

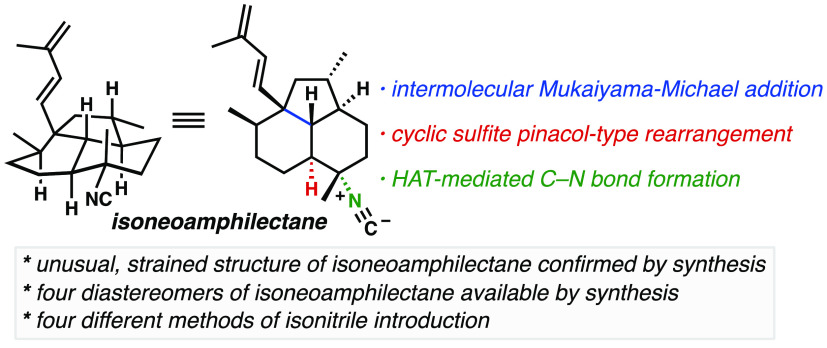

We describe a total synthesis of the rare isocyanoterpene natural product isoneoamphilectane and two of its unnatural diastereomers. The significantly strained ring system of the reported natural product—along with a hypothesis about a biosynthetic relationship to related family members—inspired us to consider a potential misassignment in the structure’s relative configuration. As a result, we initially targeted two less strained, more accessible, stereoisomers of the reported natural product. When these compounds failed to exhibit spectroscopic data that matched those of isoneoamphilectane, we embarked on a synthesis of the originally proposed strained structure via an approach that hinged on a challenging *cis*-to-*trans* decalone epimerization. Ultimately, we implemented a novel cyclic sulfite pinacol-type rearrangement to generate the strained ring system. Additional features of this work include the application of a stereocontrolled Mukaiyama–Michael addition of an acyclic silylketene acetal, an unusual intramolecular alkoxide-mediated regioselective elimination, and an HAT-mediated alkene hydroazidation to forge the C–N bond of the tertiary isonitrile. Throughout this work, our synthetic planning was heavily guided by computational analyses to inform on key issues of stereochemical control.

## Introduction

**Figure 1:**
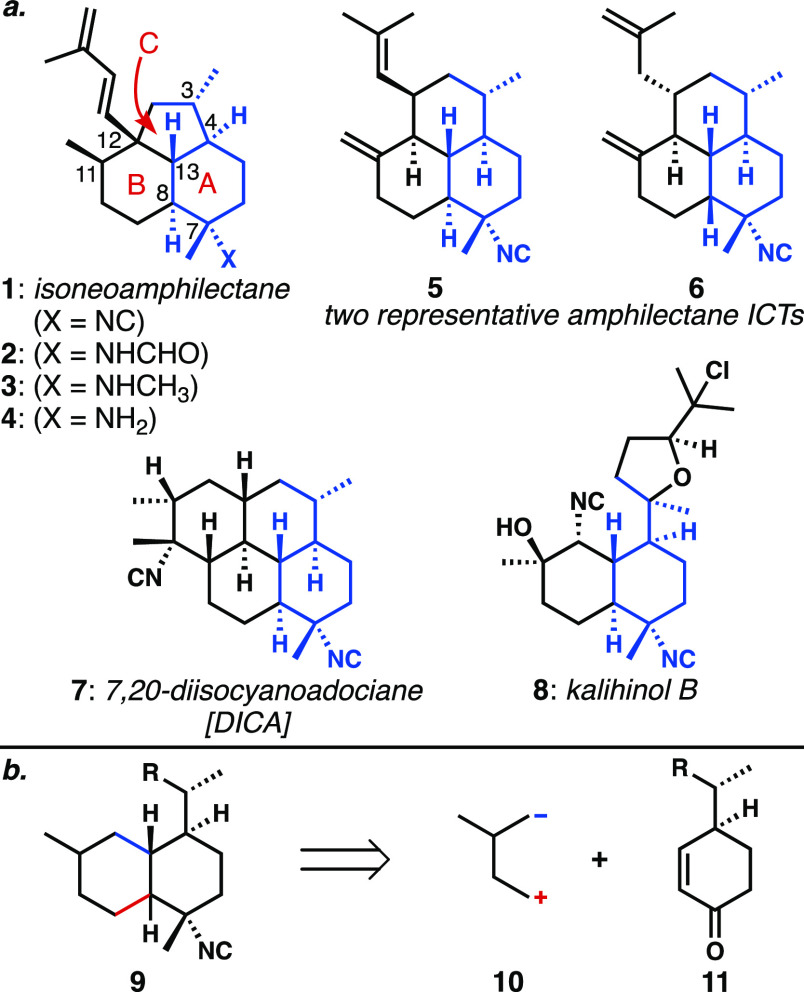
(a) Isoneoamphilectane and other representative ICTs. (b) General conjugate addition/enolate trapping strategy applicable to several ICT targets.

For decades, the isocyanoterpene (ICT) natural products have attracted the interest of synthetic chemists for their complex polycyclic structures (**1**–**8**, Figure 1a), their unusual isonitrile functional groups, and their potent bioactivities.^1^ In 1996, König et al. isolated and characterized 12 new ICTs from the tropical marine sponge *Cymbastela hooperi*.^2^ One of these new natural products was isoneoamphilectane (**1**), the first of a novel class of tricyclic amphilectane diterpenes with an unprecedented 6/6/5 fused ring system.

Studies by König and colleagues demonstrated the potent antiplasmodial activity of many ICTs against both drug-sensitive and drug-resistant strains of *Plasmodium falciparum*.^3,4^ Although not the most potent member of the family, isoneoamphilectane exhibited an IC_50_ of 100 nM against the W2 strain of the chloroquine-resistant malaria-causing parasite.^2^ Nearly two decades after the initial discovery of isoneoamphilectane, Rodríguez and co-workers isolated **1** along with analogues **2** and **3** from the extracts of the marine sponge *Svenzea flava*; primary amine derivative **4** was made by hydrolysis of **2**.^5^ They showed that **1**–**4** exhibited *in vitro* activity against *Mycobacterium tuberculosis* H37Rv. The most active of these compounds was the primary amine, which displayed an MIC of 6 μg/mL with no detectable cytotoxicity against mammalian cells. These data suggest that isoneoamphilectane could be a novel scaffold for the development of antituberculosis drugs; however, the low natural abundance (0.0004–0.007% by weight for **1**–**3**^5^) has prevented any further studies into structure/activity relationships or mechanisms of action of these compounds.

Several structural features of isoneoamphilectane conspire to set it apart from the majority of diterpenoid ICTs:^6^ (1) it has a cyclopentane ring, whereas most others are comprised of only fused six-membered carbocycles; (2) it bears a rarely observed ring junction quaternary center at C12;^7^ and (3) it has a *trans*-decalin (A/B rings) substructure. While the latter is not unusual among ring-expanded congeners (see **5**), when combined with the fused cyclopentane, it generates a particularly strained ring system that forces the B ring into a boat conformation and also results in a *trans*-hydrindane (B/C rings). In short, isoneoamphilectane’s complexity arises from seven contiguous stereocenters—including one tertiary isonitrile—imposed on a highly strained tricyclic core. The combination of these unusual structural features with the valuable bioactivity rendered isoneoamphilectane an enticing target among this family of natural products. Our group has been involved with the synthetic and biological perspectives of related natural products for nearly a decade.^8^

It is straightforward to identify a conserved structural motif in many ICTs, a 2-isocyanodecalin, whose structure might be a minimal pharmacophoric unit in the class (**9**, Figure 1b).^9^ In our work toward the ICT family, we developed a strategy that specifically targets this substructure.^10^ It features an intermolecular conjugate addition of a bifunctional reagent (**10**)/enolate trapping sequence starting from a chiral C4-functionalized enone (**11**), whose vestiges are highlighted in blue in compounds **1**–**8**. Versions of this approach have been successfully deployed in our syntheses of 7,20-diisocyanoadociane (**7**),^8c,8e^ as well as kalihinol B (**8**)^8b^ and simplified analogues,^8d^ leading to the shortest routes to these targets. We envisioned applying a related strategy to access the decalin system of isoneoamphilectane.

**Figure 2:**
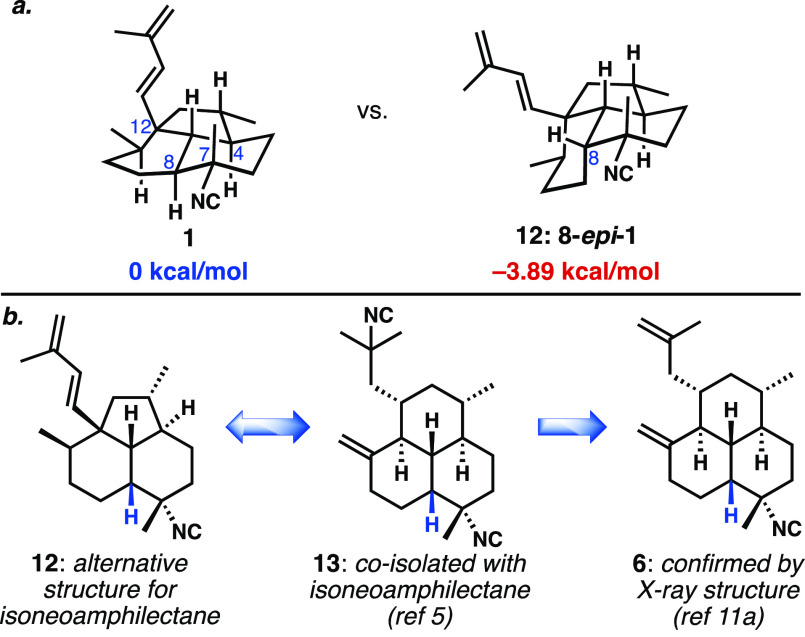
(a) Computed energies^12^ of **1** and its *cis*-isomer **12**. (b) *cis*-Amphilectanes and potential biosynthetic relationships.

The most striking feature of **1** is the confinement of the A ring to a boat conformation (Figure 2a), which is a consequence of the *trans*-decalin whose C4 and C12 positions are bridged by two carbon atoms in a *trans* orientation, completing the fused cyclopentane. The amphilectane family counts many members with embedded *trans*-decalins,^1,3^ most of which are not appreciably strained. During its initial characterization, the relative configurations of isoneoamphilectane’s C13 and C8 stereogenic centers were proposed by König to be *trans* solely based on correlation to the equivalent stereocenters of previously characterized molecules,^2^ and the natural product’s structure was not confirmed by X-ray crystallography. The stereochemical characterization of ICTs is a known challenge,^1,11^ particularly owing to the many overlapping signals in the NMR spectra. As a result, there are instances where the stereochemical assignment, particularly at C7, has been incorrect or remains to be defined.^11^ We quickly recognized that the corresponding *cis*-decalin version of isoneoamphilectane (**12**) would have significantly reduced ring strain, allowing both cyclohexanes to exist in chair conformations akin to other members of the amphilectane family. Our comparison of computed NMR shifts of structures **1** and **12** with the data available for isoneoamphilectane was inconclusive.^12^ Not surprisingly, computational analysis of the lowest energy conformations of the two epimers **1** and **12** revealed that the *trans-*decalin was found to be higher in energy than the *cis* form by ∼3.89 kcal/mol.^12^

Importantly, multiple *cis*-decalin (A/B rings) compounds are also known in the family of tricyclic amphilectanes, including **6** and **13** (Figure 2b).^11a^ The former compound was in fact originally assigned an AB *trans*-fusion, which was corrected upon X-ray crystallographic analysis. When considering the biosynthesis of these compounds, it is possible that the cyclopentane of isoneoamphilectane arises from a ring contraction of a perhydrophenalene amphilectane core. The opposite could also be operative, wherein the more common core ring system is a ring-expansion product of the cyclopentane-containing isoneoamphilectane scaffold. Though rarer than their *trans*-counterparts, we believed the existence of these *cis*-amphilectanes provided good support for the possibility that isoneoamphilectane might contain the *cis-*fused A/B ring system. Intriguingly, *cis*-amphilectane **13** was co-isolated with isoneoamphilectane by Rodríguez,^5^ furthering this possibility. Considering the lack of evidence to support the assignment of the C7 and C8 stereocenters, the strain of the *trans*-decalin, and the potential biosynthetic relationship to other *cis*-family members, *we were compelled to design a synthesis that was flexible, to permit access to all four C7/C8 diastereomers*; the isomers bearing the *cis*-ring fusion were expected to be easier to make.

**Scheme 1:**
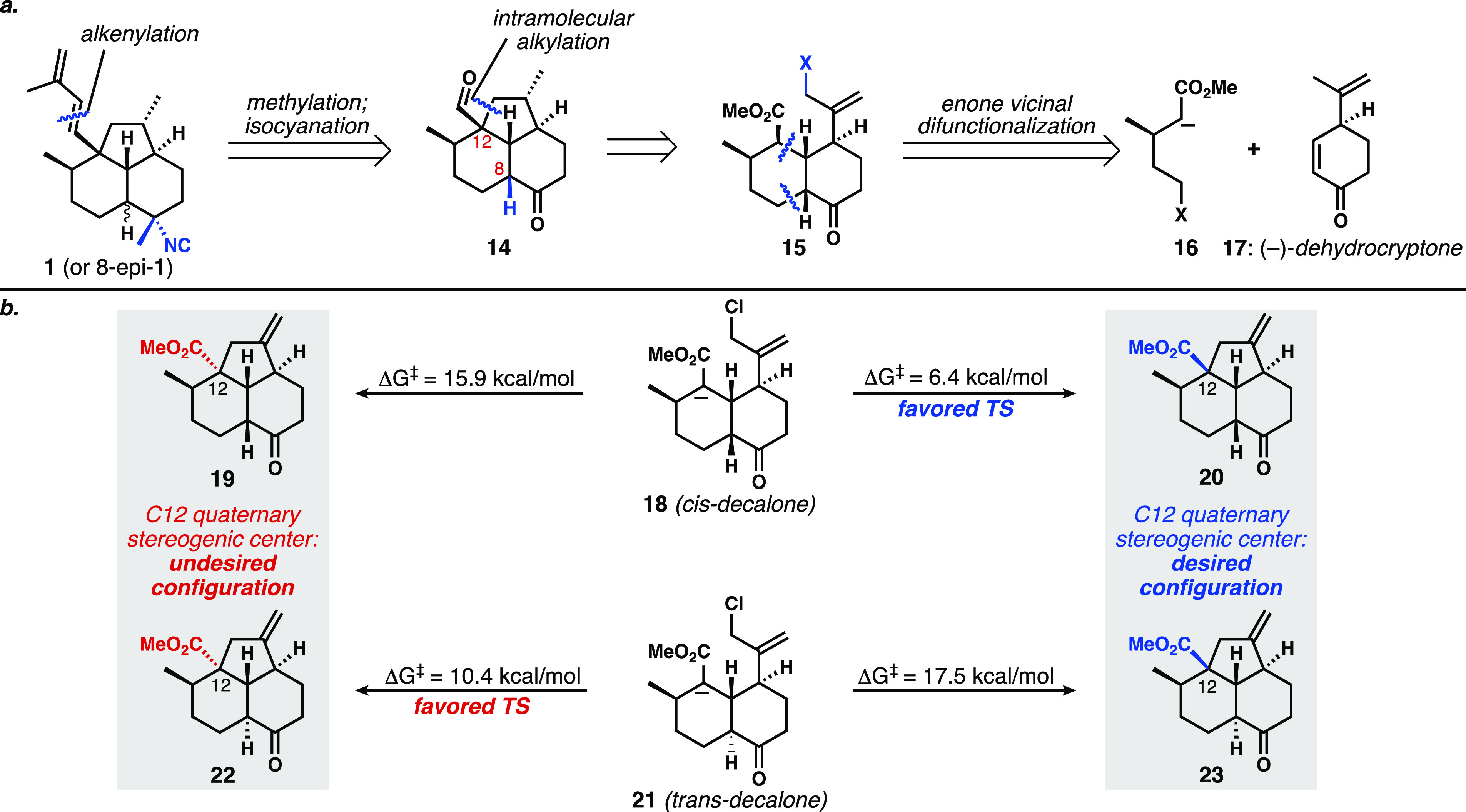
(a) Retrosynthetic Plan for Isoneoamphilectane (Potentially as *cis*- or *trans*-A/B Ring System); (b) Computational Analysis of the Transition States for Enolate Alkylation to Form the Cyclopentane Ring (ωB97X-D/6-31G(d)) For further explanations, including conformational renderings, see the Supporting Information.

Our approach would take advantage of a late-stage installation of the equatorial isonitrile using Shenvi’s invertive isocyanation^13^ of the precursor tertiary alcohol and an alkenylation to append the diene (Scheme 1a). We sought to access tricycle **14** via an intramolecular alkylation of allylic electrophile **15**. Importantly, we chose to pursue the intermediate *cis*-decalone because our computational data suggested^12^ that formation of the desired quaternary stereoisomer at C12 would be favored by nearly 10 kcal/mol from the *cis*-bicycle **18** (Scheme 1b). The same study revealed that, with the *trans*-ring fusion already established (**21**), alkylation would kinetically favor the undesired configuration at C12. We envisioned making the decalone system through a Michael addition/enolate alkylation of a bifunctional reagent of type **16** to (−)-dehydrocryptone (**17**), which is readily available on a large scale from the inexpensive monoterpenoid (*S*)-(−)-perillaldehyde.^8c,14^ With the lack of concrete evidence for the existence of the A/B *trans*-fusion and the likely challenge of generating the strained, boat-containing tricyclic ring system, we sought to first synthesize the two C7-diastereomers of the *cis*-decalin-containing isoneoamphilectane (**12** and its C7-epimer). Should neither of these compounds prove to be a spectroscopic match for the natural compound (**1**), we would embark on the presumably more difficult problem of C8 epimerization of tricyclic intermediates.

## Results and Discussion

### Synthesis of the A/B *cis*-Decalin Ring System

**Scheme 2:**
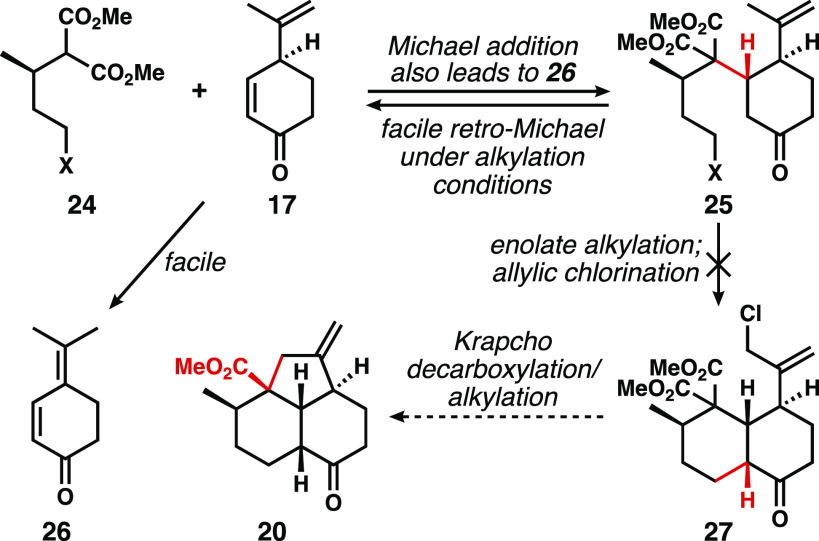
Initial Plan to Access the Tricyclic “Core” in *cis*-Decalin Form Fails Owing to Isomerization of **17** and Retro-Michael Fragmentation of **25**

Michael addition to (−)-dehydrocryptone **17** was initially envisioned using a substituted malonate nucleophile, such as **24**, to construct the decalone core via intermediate **25** (Scheme 2). After allylic chlorination, the diester **27** might be used in a Krapcho decarboxylation/alkylation event^15,16^ to forge the cyclopentane ring. However, over months of experimentation, two major issues with this approach emerged: (1) the propensity for dehydrocryptone to isomerize to the conjugated dienone **26** under Michael conditions and (2) a rapid retro-Michael fragmentation pathway of compounds of type **25** upon attempted closure of the decalin ring system by enolate alkylation. We therefore turned to Mukaiyama–Michael additions^17^ of lactone/monoester precursors.

**Scheme 3:**
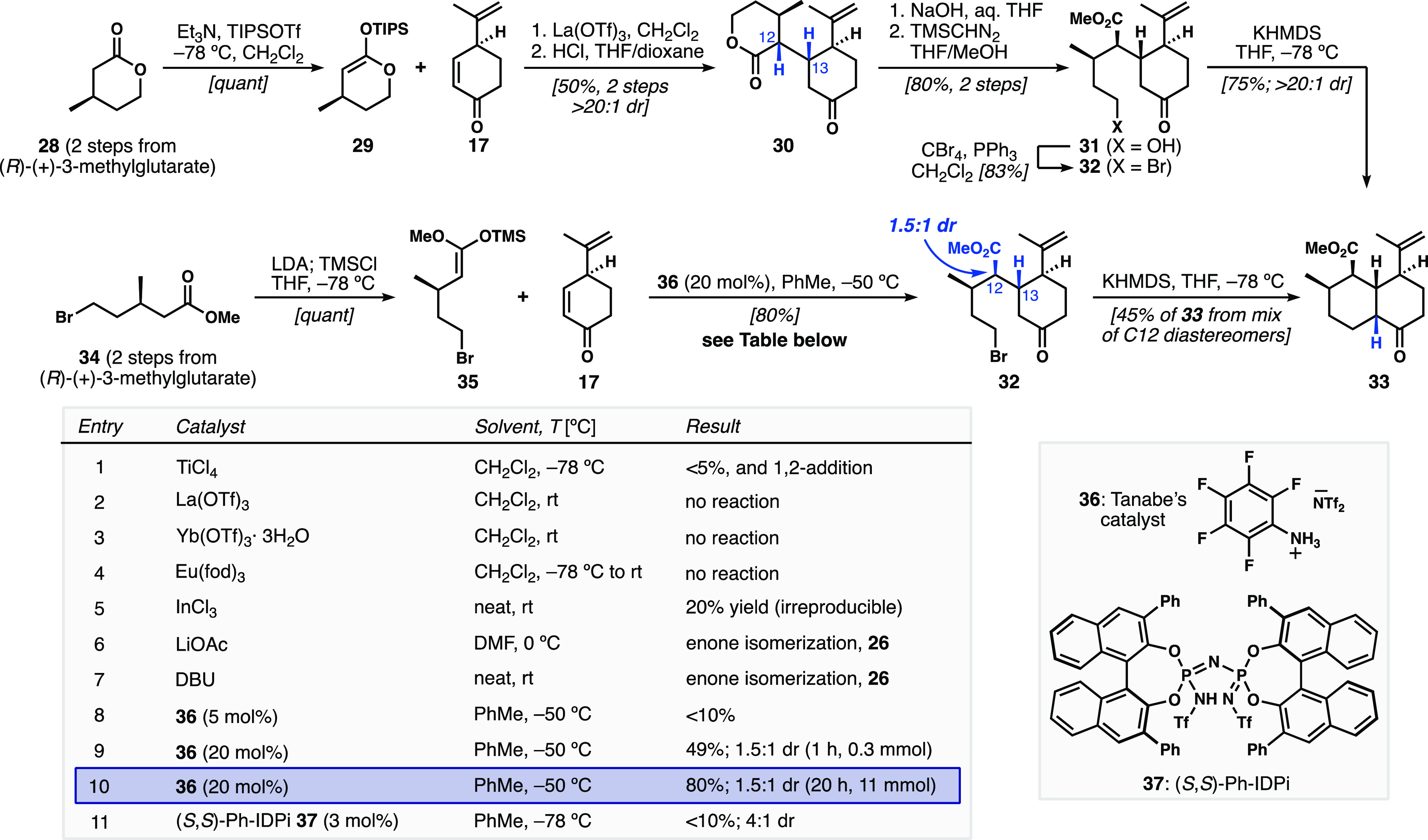
Cyclic and Acyclic Silylketene Acetals in Mukaiyama–Michael Approaches to *cis*-Decalone **33**

We found that the lactone-derived TIPS-silyl ketene acetal (SKA) **29** (Scheme 3), made from known chiral lactone **28**,^18^ was a competent nucleophile for conjugate addition to **17** with catalytic quantities of La(OTf)_3_. A similar Mukaiyama–Michael addition was reported by our lab in our efforts toward the synthesis of ineleganolide.^19^ Interestingly, this transformation gave complete selectivity at both of the newly formed stereocenters (C12 and C13), providing 1,4-adduct **30** after TIPS alkenyl ether cleavage. A two-step sequence of saponification and methylation afforded methyl ester **31** with a pendent primary alcohol. Finally, Appel reaction to alkyl bromide **32** and treatment with KHMDS afforded the desired *cis*-decalone **33** as a single diastereomer with no retro-Michael reactivity observed.

While our first-generation *cis*-decalone route proved successful and allowed us to explore downstream chemistry, its length and the resulting poor scalability were limiting. To enhance the efficiency of the synthesis, we revisited the Mukaiyama–Michael addition with an acyclic SKA. Unfortunately, there are limited examples in the literature that describe the addition of linear SKAs to cyclohexenones. Furthermore, transformations that tolerate substitution on the enone are rare, and Mukaiyama–Michael additions involving β-branched alkyl-substituted SKA are unknown. Nevertheless, we made TMS-SKA **35** from known ester **34**.^20^ Traditional Lewis-acid-catalyzed Mukaiyama–Michael additions were ineffective (table in Scheme 3, entries 1–5), and Lewis base catalysis^21^ was incompatible with enone **17** leading to **26** (entries 6 and 7). We finally found success upon adoption of the Brønsted acid catalyst pentafluorophenylammonium triflimide (C_6_F_5_NH_3_^+^·NTf_2_^–^, **36**), developed by Tanabe for Mukaiyama aldol and Mukaiyama–Mannich reactions.^22^

This catalytic system is believed to generate trimethylsilyl bistriflimide *in situ*, which behaves as the active Lewis acid catalyst. Applying the reported conditions provided trace amounts of ketone product **32** (entry 8); however, increasing the catalyst loading from 5 mol % to 20 mol % furnished a 49% yield with 1.5:1 dr at the methyl-ester-bearing stereocenter (C12) (entries 8, 9) while maintaining perfect control at C13. After further optimization, we found that increasing the reaction time led to full consumption of the enone and an 80% isolated yield of **32** (entry 10). Unfortunately, when the mixture of diastereomers was treated with KHMDS, only the major component cyclized to **33** and the other diastereomer was left unchanged, leading to a lowered yield in the subsequent alkylation step. If equilibrating basic conditions were used (e.g., NaOMe or KO*t*-Bu), a mixture of diastereomeric products was obtained in a much-diminished yield. No evidence of C12 equilibration was ever observed in the conditions (KHMDS) that were successful for ring closure.

We briefly attempted improving the diastereoselectivity of the transformation by utilizing the imidodiphosphorimidate (IDPi) Brønsted acid catalysts developed by the List group.^23^ Preliminary results with the (*S*,*S*)-phenyl-IDPi **37** gave a promising outcome of a 4:1 dr; however, the conversion was very low (entry 11). This is likely due to the extremely low catalyst loadings (0.01–1 mol %) typically used by the List group in related systems. These loadings can prove challenging because traces of basic impurities in the starting materials can render the catalyst inactive and halt turnover. Unfortunately, in our system, even with careful purification of the substrates by distillation, and using 3 mol % catalyst, the yield was still low. We believe this transformation might be efficient with higher loadings of **37**, but the multigram quantities of this large, expensive catalyst needed for material throughput were not readily available. In conclusion, both routes (lactone-derived SKA and acyclic SKA) provided material to explore downstream chemistry, with the second-generation route being more concise (5 linear steps from (*R*)-(+)-3-methylglutarate, 6 linear steps from perillaldehyde), and providing a higher throughput in a shorter time.

### Synthesis of 7,8-Di-*epi*-isoneoamphilectane

**Scheme 4:**
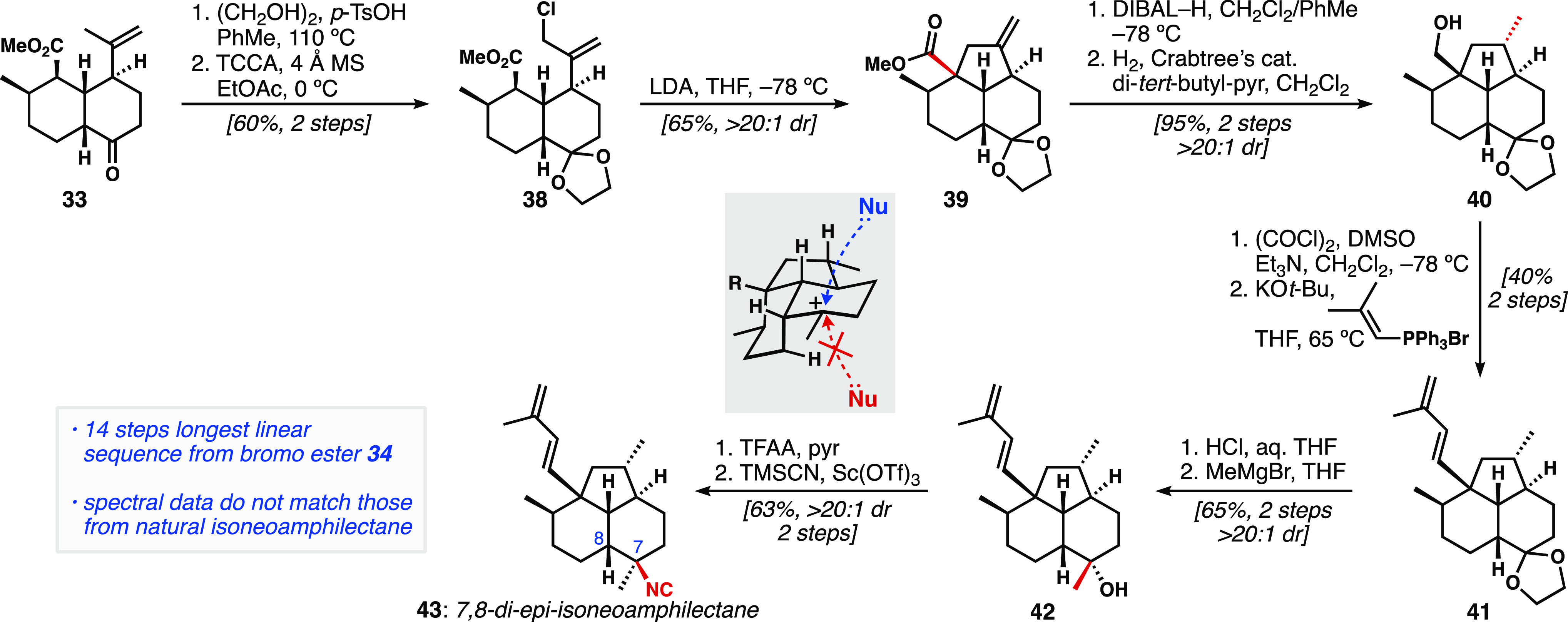
Synthesis of 7,8-Di-*epi*-isoneoamphilectane (**43**) TCCA: trichloroisocyanuric acid.

With *cis*-decalone **33** in hand, efforts were focused on formation of the fused cyclopentane ring (Scheme 4). Ketone protection followed by allylic chlorination afforded tricycle precursor **38**. Generation of the ester enolate with LDA allowed for intramolecular alkylation, forging the five-membered ring and C12 quaternary stereogenic center of **39** with >20:1 dr. Importantly, this transformation substantiated our initial strategy of using the *cis-*decalin for stereoselective ring closure (Scheme 1b). Next, reduction of the methyl ester to the primary alcohol provided the necessary directing group for hydrogenation with Crabtree’s catalyst,^24^ allowing us to access **40** as a single diastereomer; the addition of di-*tert*-butylpyridine was required to prevent partial ketal cleavage. Swern oxidation and Wittig alkenylation furnished diene **41**. Ketal hydrolysis preceded nucleophilic ketone methylation, which took place stereoselectively from the convex face of the molecule, providing tertiary alcohol **42** as a single diastereomer. Installation of the tertiary isonitrile was accomplished using Shenvi’s two-step procedure of trifluoroacetylation and treatment with catalytic Sc(OTf)_3_ in TMSCN.^14^ Despite the rarely perfect diastereoselectivity reported in the literature, this reaction provided a single diastereomer of the axial isonitrile product **43**. This selectivity highlights the inherent favorability for axial approach of the nucleophile in this general system, biased by the concavity of the *cis*-decalin fused tricycle (see inset). This sequence generated 7,8-di-*epi*-isoneoamphilectane, which differs from the reported structure of the natural product both at the ring fusion (C8) and at the isonitrile-bearing stereocenter (C7). Comparison of our NMR data with those of the reported spectra revealed that **43** was indeed a stereoisomer of the natural product.^12^

### Synthesis of 8-*epi*-Isoneoamphilectane

Next, we attempted to synthesize 8-*epi*-isoneoamphilectane (**12**, Figure 2b, equatorial C7-isonitrile). To obtain this configuration of isonitrile, we aimed to leverage Tada’s diastereoselective conversion of 1,1-disubstituted alkenes to tertiary isonitriles.^25^ To apply this transformation to our system, we elaborated tricycle **39** to the exocyclic methylidene **44** (eq 1).^12^ The reaction is proposed to proceed by silver-catalyzed isomerization of a 1,1-disubstituted alkene to the corresponding trisubstituted alkene. Next, the silver is hypothesized to coordinate to the alkene from its less-hindered face, and, in an antiparallel fashion, TMSCN adds from the opposite face. After protodemetalation, tertiary isonitriles are formed in high yields and diastereoselectivities. We hoped that the silver reagent would engage the trisubstituted alkene from the less-hindered, convex β-face, thereby forcing TMSCN addition to the α-face, giving rise to the desired equatorial isonitrile; however, treatment of **44** under these conditions led exclusively to the axial isonitrile **45**; the mass balance consisted of isomerized endocyclic alkene.
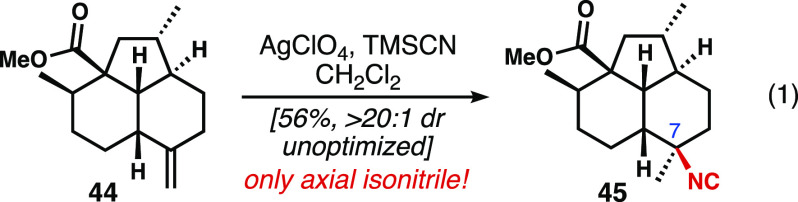
1

This stereochemical outcome was difficult to ascertain until the diene was introduced. At that stage, 7,8-di-*epi*-isoneoamphilectane (**43**) was isolated once again; this 13-step longest linear sequence from **34** proved one step shorter than that shown in Scheme 4.^12^ This stereochemical result suggests that the hydroisocyanation likely favored axial attack through a more discrete carbocation-like intermediate (see inset). Considering the incredibly high diastereoselectivities obtained from both the Shenvi and Tada conditions, it became apparent that any attempt at nucleophilic isocyanation would greatly favor addition from the convex, β-face of the *cis*-tricycle, and thus lead to 7,8-di-*epi*-isoneoamphilectane (**43**).

**Scheme 5:**
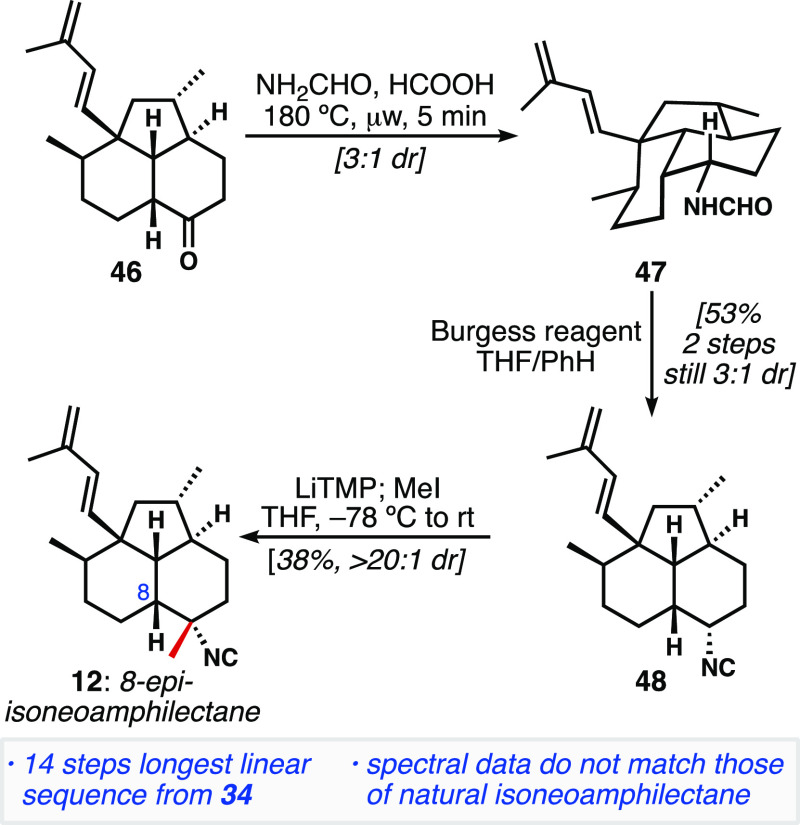
Synthesis of 8-*epi*-Isoneoamphilectane via Leuckart Reaction and an Uncommon Methylation of an Isonitrile-Stabilized Anion LiTMP: lithium 2,2,6,6-tetramethylpiperidide.

To access *cis*-isoneoamphilectane **12** with an equatorial isonitrile at C7, an alternative synthesis was developed (Scheme 5). We pursued a different order of operations by first installing the isonitrile and then performing an axial methylation. Subjecting **46** (the hydrolysis product of **41**) to a Leuckart–Wallach reaction^26^ yielded formamide **47** as a 3:1 mixture favoring the equatorial isonitrile. This reaction showed that hydride delivery in the reduction step is more facile from the convex β-face, as expected. Subsequent dehydration of the formamide with Burgess reagent^27^ furnished secondary isonitrile **48**. Unlike most electron-withdrawing functional groups, deprotonation alpha to isocyanides poses a significant challenge; productive metalation typically only occurs when an additional electron-withdrawing group is present. A standalone example of an unactivated isonitrile alkylation is reported by Fleming et al. where the alkylation of cyclohexyl isonitrile is accomplished using (TMP)_2_Mg·LiCl and excess propyl iodide in 63% yield.^28^ Unfortunately, in our hands these conditions were not applicable to the use of methyl iodide as the electrophile. As a result, we investigated a variety of bases (e.g., NaH, *n-*BuLi, LDA) to accomplish the deprotonation/alkylation transformation in our system. We found that LiTMP was the most productive base, and we were able to obtain 8-*epi*-isoneoamphilectane **12** in 38% yield (largely unoptimized). NMR comparison of **12** to the spectra for the natural product isoneoamphilectane showed no match, indicating that our hypothesis that the natural product might exist as the *cis*-decalin was incorrect. Clearly, we needed to target compounds bearing the *trans*-A/B ring fusion. While we did not attempt to further optimize the α-methylation, we believe that this transformation is an underutilized and potentially powerful approach to the synthesis of tertiary isonitriles.

While isoneoamphilectane was not one of the *cis*-diastereomers we produced, we believe there is the potential that **43** and/or **12** exist in nature but have yet to be isolated and characterized. We hope that our efforts can serve as a resource for isolation chemists during future sponge-derived ICT characterization and that the *cis*-fused epimers are eventually found in nature.

### Epimerization Strategies

**Scheme 6:**
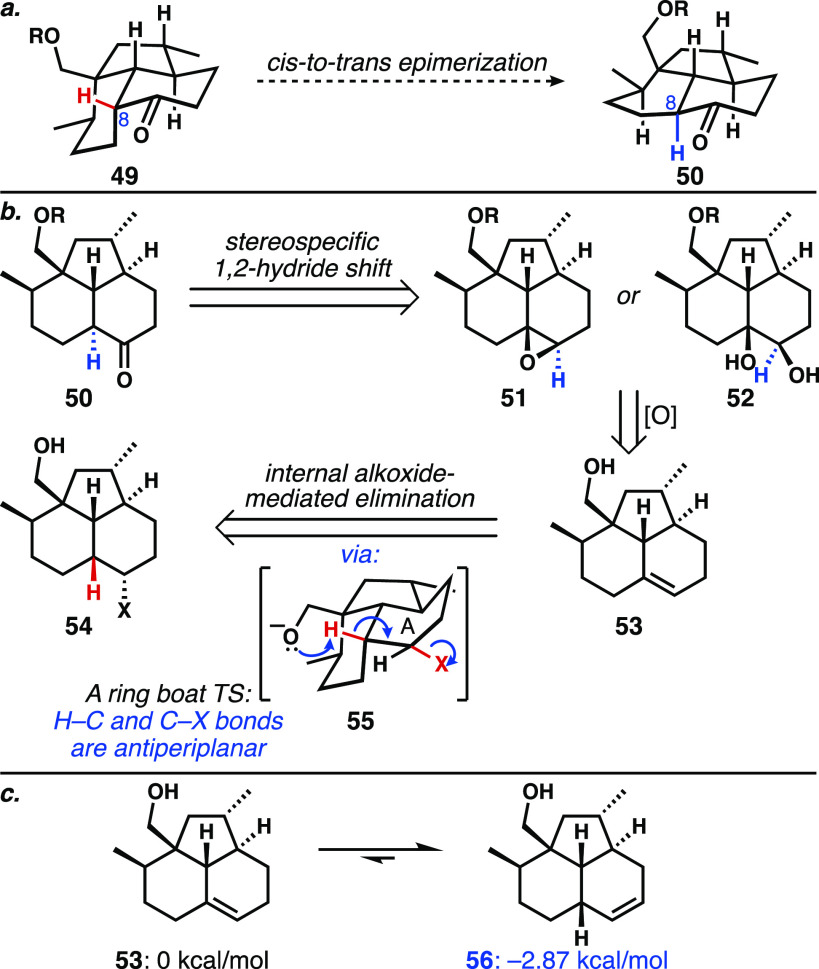
(a) Requisite Epimerization; (b) Plan for a Stereospecific Hydride Migration Necessitates an Alkene of Type **53**; (c) Computation Shows That Alkene **53** Is Less Stable than Its Regioisomer **56** (Using ωB97X-D/6-31G(d))

With newfound confidence that **1** exists as the isolation chemists originally proposed^2,3^—a *trans*-fused decalin—we began investigating epimerization strategies (**49** to **50**, Scheme 6a). With the knowledge that the *trans*-tricycle is significantly higher in energy relative to the *cis*-tricycle (Figure 2a), the development of a contrathermodynamic (kinetically driven) epimerization approach (Scheme 6b) was required. Efforts were directed toward a stereospecific 1,2-hydride shift using epoxide **51** or diol **52** (or its derivative). To pursue this strategy, we needed to access alkene **53**. Unfortunately, the desired regioisomer **53** was found by computation to be higher in energy than the undesired disubstituted alkene **56** by ∼2.87 kcal/mol (Scheme 6c).^12^ To overcome this hurdle, we investigated the use of the pendent alcohol to generate an alkoxide that might intramolecularly effect an elimination of a leaving group at C7 (see **55**, note that hand-held models do not indicate particularly good overlap for an E2 process). The 6-*endo-tet*-like deprotonation event does not appear to be subject to the same strict geometrical constraints as reactions involving an electrophilic carbon atom, and related processes are known.^29^ An intramolecular alkoxide-assisted elimination was reported by Seike and Sorensen in their formal synthesis of FR901483,^29c^ and the use of an alkoxide to intramolecularly direct an alkene isomerization was demonstrated by Shenvi in the synthesis of kalihinol C.^30^

**Scheme 7:**
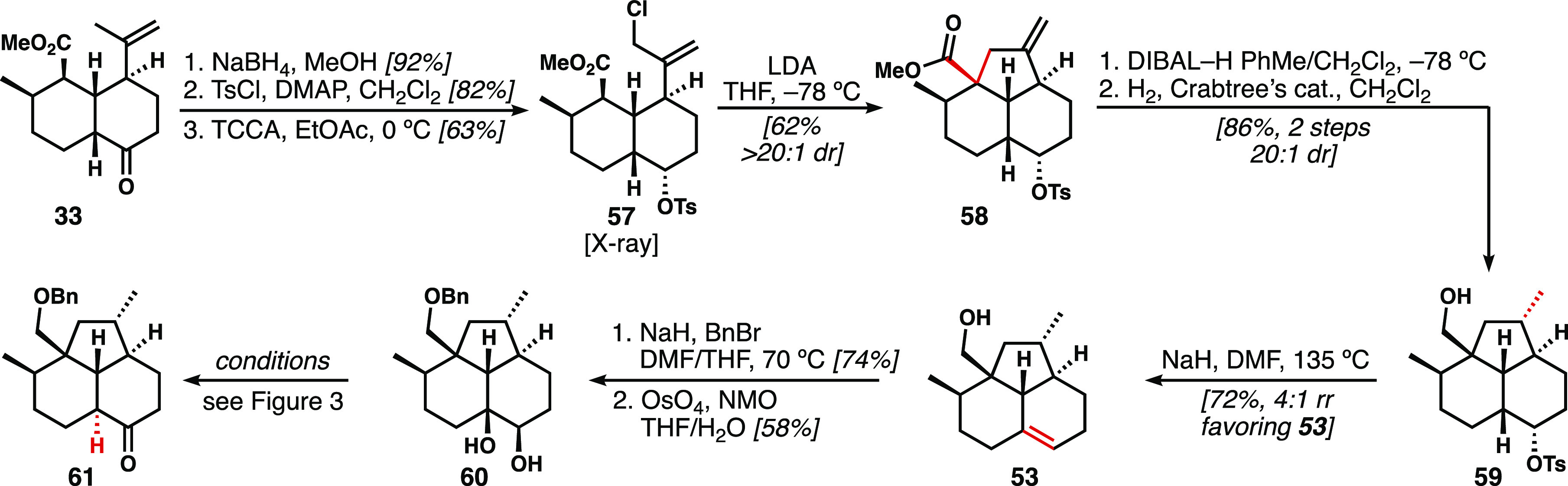
Alkoxide-Directed Elimination Provides Alkene **53**, a Key Precursor to Intermediates for Stereospecific, Hydride-Shift-Induced *cis*- to *trans*-Decalin Transformation TCCA: trichloroisocyanuric acid.

The synthesis of a substrate to investigate the proposed regioselective alkoxide-directed elimination commenced from *cis*-decalin **33** (Scheme 7). Sodium borohydride reduction, tosylation, and allylic chlorination furnished tricycle precursor **57** (structure confirmed by X-ray crystallography). Of note, installation of the tosylate prior to cyclization circumvented the necessity for ketone protection as in the *cis*-isoneoamphilectane route. Treatment with LDA smoothly afforded **58** as a single diastereomer, in the face of a potentially destructive enolate-induced Grob fragmentation. Subsequent ester and alkene reductions generated the desired substrate **59** for elimination studies. After exploring a wide range of bases and solvents, the most successful conditions found involved heating in DMF with excess NaH, yielding 72% and a 4:1 ratio of alkene regioisomeric products, favoring the desired product **53** (for a complete table of conditions see Supporting Information). Importantly, application of these same conditions to an *O-*protected version of **59** led primarily to the undesired regioisomer. Benzyl ether formation followed by dihydroxylation furnished diol **60**, which was used to investigate pinacol-type epimerization strategies toward ketone **61**.

**Figure 3:**
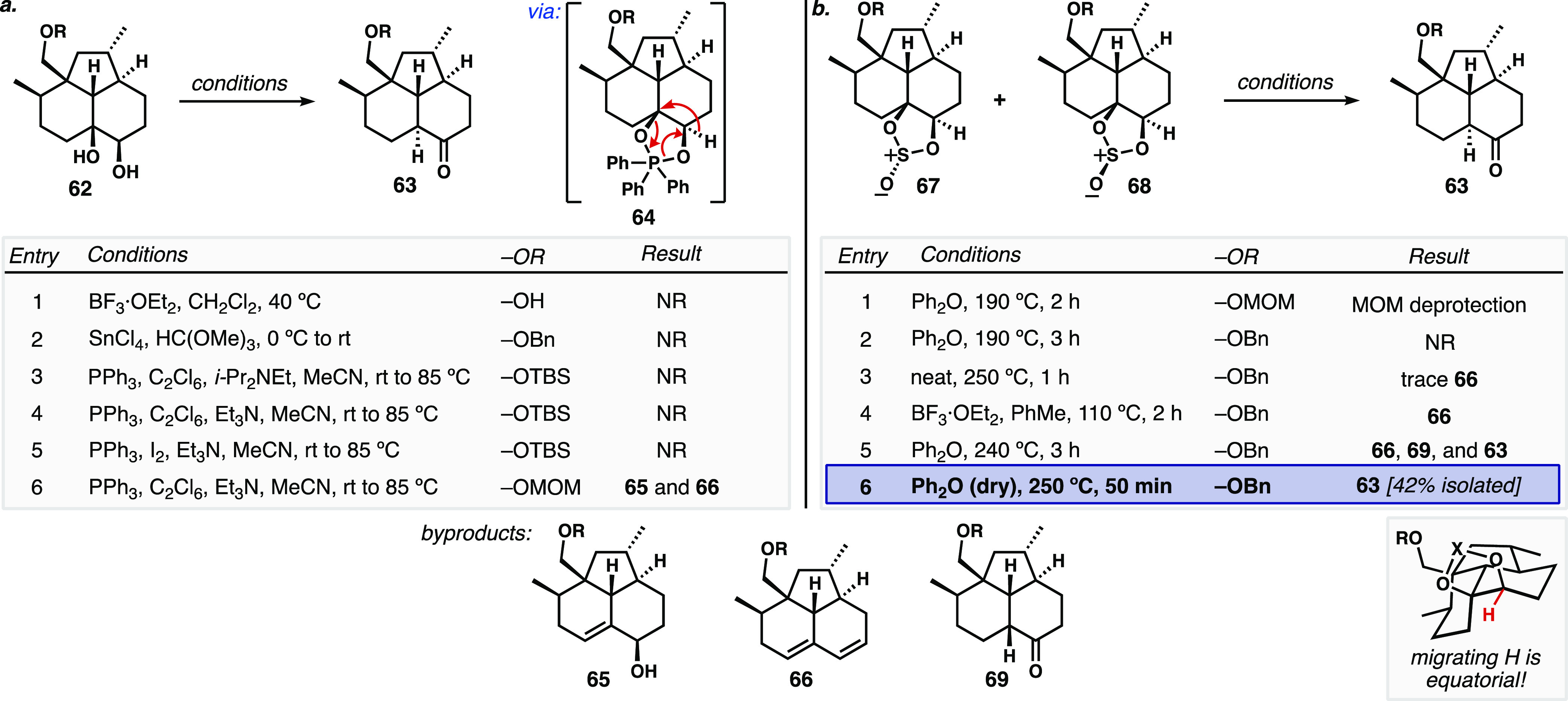
(a) Representative attempts to achieve pinacol-like rearrangements of diol **62** via Lewis acid catalysis, orthoformate, or dioxaphosphorane intermediates. (b) Optimization of pinacol-like rearrangement of cyclic sulfites **67/68**.

Lewis acid-catalyzed pinacol rearrangements^31^ of **62** catalyzed by BF_3_·OEt_2_ (R = H), or SnCl_4_ with trimethyl orthoformate (R = Bn),^32^ were unproductive (Figure 3a, entries 1 and 2). We then investigated the use of a phosphorane-mediated pinacol-like rearrangement originally developed by De Camp, Mills, and co-workers^33^ to install *trans*-decalones from *cis*-diols via a formal 1,2-hydride shift. Grainger also extended this transformation to the conversion of *cis*-diols into *trans*-hydrindanones;^34^ this method was a key step in Trauner’s total synthesis of wickerol A.^35^ When *cis*-diol **62** (R = TBS) was exposed to the standard conditions of triphenylphosphine, hexachloroethane, and an amine base (Et_3_N or *i*-Pr_2_NEt), no reaction occurred (entries 3 and 4). Increasing the electrophilicity of the phosphonium dihalide from the chloride to the iodide was also ineffective (entry 5). Because we were not observing formation of the intermediate cyclic phosphorane in any of our attempts, we posited that the steric demand of having both the TBS protecting group and the cyclic phosphorane on the β-face was impeding reactivity. As a result, we installed a MOM ether, and in this case, formation of an intermediate that we believe to be the cyclic phosphorane **64** was observed by thin layer chromatography and mass spectrometry (entry 6). Upon heating the reaction to induce the desired rearrangement, we instead observed the formation of elimination products, allylic alcohol **65**, and diene **66**. The small quantities of **65** (and *O-*protected variants) produced in this way were evaluated for hydrogenation to set the C8 stereogenic center, but without success. We briefly explored Meinwald-type rearrangements of the β-epoxide corresponding to **51** (Scheme 6b),^34,36^ but the epoxidation step was inefficient and capricious, and the attempted rearrangements of any epoxide that could be isolated led primarily to the formation of undesired elimination products **64** and **65**. It is worth noting that hand-held models show—in either Meinwald-type or cyclic phosphorane pinacol-type rearrangements—that the σ_C–H_ and σ*_C–O_ orbitals have very poor overlap as a result of the constraints imposed by the strained tricyclic ring systems. The hydrogen (hydride) in question is actually forced into a pseudoequatorial orientation in this ring system (see Figure 3b inset).

The main drawbacks of the cyclic phosphorane pinacol approach seemed to be high leaving group ability of Ph_3_P=O and the need for basic conditions which proved problematic due to the propensity for elimination instead of a concerted rearrangement. With hopes of finding neutral conditions that would invoke a similar rearrangement, we turned to the use of cyclic sulfites. As opposed to cyclic sulfates, whose utility in organic synthesis has been well exploited for use in double displacement-type reactions, uses of the less oxidized cyclic sulfite are uncommon.^37^ In a report by the Grainger group in 2012, semipinacol rearrangements of *cis*-fused β-lactams were showcased via either a cyclic phosphorane or cyclic sulfite intermediates.^38^ To our knowledge, this report is the only example of using cyclic sulfites in pinacol-type reactions. In this work, Grainger and co-workers observe an *N*-acyl-group migration upon loss of sulfur dioxide when heating cyclic sulfites to 190 °C in diphenyl ether. Although prior examples of hydride migrations in cyclic sulfite thermolyses have not been reported, we hoped that such a transformation could be applied to solve our stereochemical problem. Treatment of diol **62** with thionyl chloride and triethylamine quantitatively forms cyclic sulfites **67** and **68** in ∼1.4:1 dr (Figure 3b, major isomer not identified). Heating to 190 °C in diphenyl ether with a MOM protecting group simply led to acetal cleavage to the primary alcohol, and with a benzyl ether in place, no reaction was observed (entries 1 and 2). Attempted thermolysis neat at 250 °C (microwave) or Lewis acid activation in toluene at reflux each resulted in some elimination to the diene (**66**, entries 3 and 4). Heating to 240 °C in diphenyl ether (microwave) gave incomplete conversion to a mixture of *cis*- and *trans*-decalone products (**69** and **63**, respectively) as well as some diene **66** (entry 5). We suspected an undesired *in situ* epimerization of the *trans* product could be caused by adventitious water at the extremely high reaction temperature. After taking extensive precautions to exclude moisture from the reaction, we were gratified to reliably obtain a 42% isolated yield of the desired *trans*-decalin product **63** (entry 6) when heated to 250 °C (microwave). There is no indication of preferential rearrangement of either diastereomer of cyclic sulfite (**67**/**68**).

### Synthesis of Isoneoamphilectane

With the *trans*-decalone finally in hand, the synthesis of isoneoamphilectane (**1**) appeared within reach, given the endgames established for the two diastereomers (**12** and **43**) we had already made. Thus, we converted ketone **61** into tertiary trifluoroacetate **70**(12) (eq 2) and subjected it to the standard conditions of catalytic quantities of Sc(OTf)_3_ in neat TMSCN with the expectation of obtaining the natural product, isoneoamphilectane (**1**). Unfortunately, all attempts at introducing the desired tertiary isonitrile were unproductive regardless of reaction temperature or source/activation mode of Sc(OTf)_3_. Instead, these attempts resulted in the formation of two new products that appeared to be the result of complex carbocation rearrangements (likely driven by strain relief) and which could not be structurally characterized. The Shenvi isocyanation has proven to be a powerful transformation in many ICT syntheses in both their lab^30,39^ and ours;^8b,8d,8e^ we attribute the significant ring strain in our substrate as the reason for the unfavorable outcome.
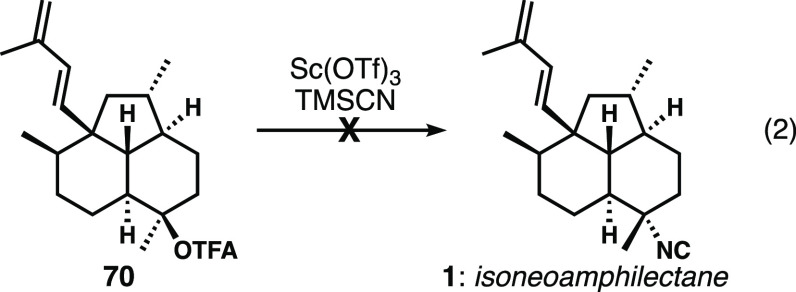
2

Drawing inspiration from prior synthetic work on ICTs, we considered other options for isonitrile introduction. Nucleophilic methylation of an imine electrophile derived from **61**, which would likely provide the desired configuration at C7, was deprioritized on account of facile epimerization of C8 to reform the *cis*-decalin under the conditions of imine formation. A second approach that is well precedented in the ICT literature is the aziridination of an exocyclic alkene, reductive opening of the heterocycle, and subsequent conversion to the tertiary isocyanide. This transformation has been utilized by Wood in the synthesis of kalihinol C^40^ as well as by Miyaoka in the synthesis of (±)-7-isocyanoamphilecta-11(20),15-diene.^41^ An alternative approach we envisioned—which had not at the time been applied to the synthesis of ICTs^42^—is a hydrogen-atom-transfer-(HAT)-mediated hydroazidation of an alkene. Simple functional group interconversions would transform the tertiary azide to the desired isonitrile. We were encouraged by precedence from the groups of Carreira^43^ and Boger,^44^ each of whom reported conditions for alkene hydroazidation, using cobalt and iron catalysts, respectively.

**Scheme 8:**
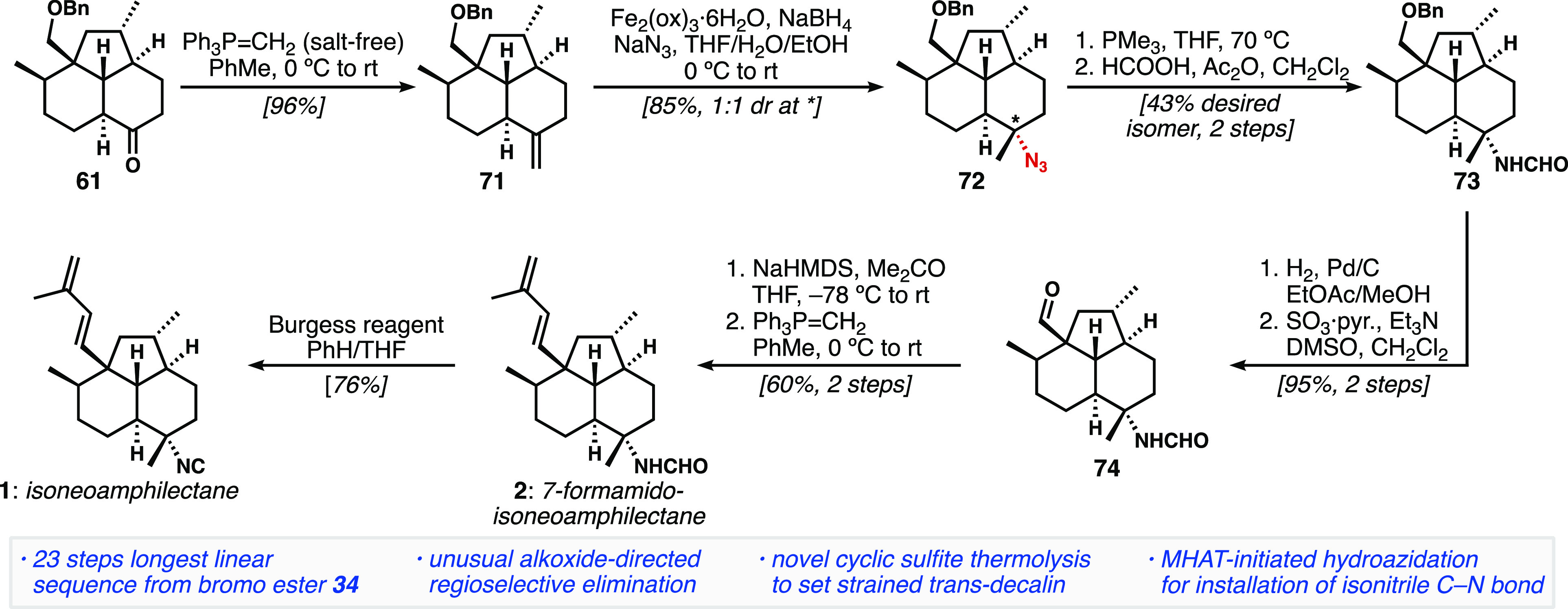
Endgame for the Synthesis of Isoneoamphilectane (**1**) Relying on an MHAT-Initiated Alkene Hydroazidation for Introduction of the Isonitrile Nitrogen Atom

Both of our proposed strategies relied on the synthesis of exocyclic cyclohexene **71** (Scheme 8) from our pinacol-like rearrangement product, **61**. We were wary of potential undesired epimerization upon treatment of **61** under standard Wittig alkenylation conditions (using strong base to generate the ylide *in situ*), but found that salt-free Wittig methylenation provided **71** without issues.

On the basis of precedent, we believed the aziridination would have a high likelihood of success; however, treatment of **71** with *N*-tosyliminobenzyliodinane and Cu(OTf)_2_^40,45^ led to the corresponding spirocyclic aziridine in poor yield and diastereoselectivity, along with debenzylated starting material, and a mixture of other products. Brief attempts at optimization were unsuccessful. As a result, we turned to hydroazidation of **71**. Azide **72** could be obtained in high yield using the conditions of either Carreira^43^ or Boger,^44^ but the latter was operationally simpler. In both cases, presumably owing to the early transition state associated with radical reactions and the small size of azide donor reagents, the products were obtained as a 1:1 ratio of inseparable diastereomers. Although not ideal, we continued with our synthetic efforts toward isoneoamphilectane from this mixture of tertiary azides **72**.

Staudinger reduction afforded an amine that was directly formylated to give **73**, at which point the desired, equatorial formamide could be separated from the undesired axial formamide with high efficiency. Hydrogenolysis of the benzyl ether proceeded smoothly, and the alcohol was converted to aldehyde **74** via Parikh–Doering oxidation. Interestingly, azide reduction and hydrogenolysis could not be successfully combined into a single operation. The diene was installed using our previously established sequence of aldol condensation with acetone and enone methylenation. Gratifyingly, the spectral data of equatorial formamide **2** matched the natural product reported by Rodríguez^5^ and thus completed the total synthesis of 7-formamidoisoneoamphilectane in 22 steps from known compounds. Dehydration of **2** with Burgess reagent furnished the isonitrile isoneoamphilectane (**1**), whose spectral data were consistent with those reported by König and Wright.^2,46,47^

## Conclusions

We have completed the first total synthesis of the strained ICT isoneoamphilectane (**1**) and its naturally occurring formamide congener (**2**) in 23 and 22 steps, respectively, from known alkyl bromide **34**. The synthesis plan was designed to be flexible with respect to accessing C7/C8 diastereomers in the event that the originally reported stereochemical structure was incorrect, and, as a result, we developed efficient routes to 8-*epi*- and 7,8-di-*epi*-isoneoamphilectanes. By completing an enantiospecific total synthesis of **1** and **2** from the chiral pool starting material (−)-dehydrocryptone (**17**), we were also able to confirm the absolute configuration of the unique isoneoamphilectane class of natural products. *Critically, this body of work serves as a rigorous determination of the stereochemical relationships in these particularly strained, complex diterpenoids*.

Broadly applicable lessons and unusual/novel transformations that will transcend this specific total synthesis endeavor include (1) a Mukaiyama–Michael addition of a linear, β-branched silyl ketene acetal uniquely mediated by an ammonium triflimide precatalyst; (2) an intramolecular alkoxide-directed regioselective and contrathermodynamic elimination; (3) a thermally induced pinacol-like 1,2-hydride shift of a cyclic sulfite to access a strained *trans*-decalin; (4) the novel generation of a tertiary isonitrile via Leuckart–Wallach reaction/dehydration/isonitrile-stabilized carbanion alkylation sequence; and (5) the introduction of a tertiary isonitrile via an HAT-mediated hydroazidation of an alkene. In total, four different methods were showcased for introduction of the salient isonitrile functional groups onto the hydrocarbon scaffold of the natural product or its diastereomers.

Our work toward isoneoamphilectane exemplifies the challenges associated with the synthesis of compact, strained polycyclic molecules. This is demonstrated by comparing our syntheses of the *cis*-decalin- and *trans*-decalin-containing isoneoamphilectanes. The *cis*-fused (unnatural) isoneoamphilectanes—which might well be as-yet-undiscovered secondary metabolites—only required 13 or 14 steps to construct. In contrast, the strained, *trans*-fused isoneoamphilectane natural product required 9 additional operations, despite differing at only one stereogenic center. Overall, the synthetic challenges we faced throughout this work brought a wealth of opportunities for the development and implementation of new or underutilized chemical transformations.

## Supporting Information Available

Experimental procedures, characterization data, and NMR spectra for all new compounds (PDF)
