# Supplementary material for: An Enantiospecific Synthesis of Isoneoamphilectane Confirms Its Strained Tricyclic Structure
Source: J Am Chem Soc. 2023 Feb 2;145(6):3716–26. doi: 10.1021/jacs.2c13137 (PMC9936588; doi:10.1021/jacs.2c13137)
Supplement: Supplementary file 1 — ja2c13137_si_001.pdf [file ja2c13137_si_001.pdf]

An Enantiospecific Synthesis of Isoneoamphilectane Confirms Its Strained Tricyclic Structure

Natalie C. Dwulet[a], Zeinab Chahine[b], Karine G. Le Roch[b], and Christopher D. Vanderwal[a,c]

[a]*Department of Chemistry, University of California, Irvine, California 92697-2025, United States*

[b]*Institute for Integrative Genome Biology, Center for Infectious Disease and Vector Research, 900 University Avenue, Department of Molecular, Cell, and Systems Biology, University of California, Riverside, CA 92521, United States*

[c]*Department of Pharmaceutical Sciences, 101 Theory, University of California, Irvine, California 92697, United States*

cdv@uci.edu

Supporting Information

Table of Contents

A. General Experimental Details S3

B. Experimental Procedures.. S4

Synthesis of 7,8-di-*epi*-isoneoamphilectane (via Shenvi isocyanation)....................... S15

Synthesis of 7,8-di-*epi*-isoneoamphilectane (via Tada isocyanation) S20

Synthesis of 8-*epi*-isoneoamphilectane.. S26

Synthesis of *trans*-decalone containing tricycle S29

Attempted synthesis isoneoamphilectane (via Shenvi isocyanation) S40

Synthesis of 7-formamidoisoneoamphilectane and isoneoamphilectane.................... S44

C. Computational Studies and Procedures S53

Relative energy calculations.. S53

Predicted NMR spectra for isoneoamphilectane and 8-*epi*-isoneoamphilectane........ S59

Transition state calculations S66

D. X-Ray Crystal Structure of Tosylate **57** S65

E. NMR Spectra.. S84

F. Biological Data S206

G. References S208

A. General Experimental Details

All reactions were conducted in flame- or oven-dried glassware under an inert atmosphere of argon (Ar) unless otherwise noted. Reaction solvents including dichloromethane (CH_2Cl_2, Fisher, HPLC Grade), tetrahydrofuran (THF, Fisher, HPLC Grade), toluene (PhMe, Fisher, ACS Grade), methanol (MeOH, Fisher, low water Grade), benzene (PhH, Fisher, ACS Grade), and dimethylsulfoxide (DMSO, Fisher, ACS Grade) were dried by percolation through a column packed with neutral alumina and a column packed with Q5 reactant (a supported copper catalyst for scavenging oxygen) under a positive pressure of Ar. Dimethylformamide (DMF, Fisher, ACS Grade) was dried by percolation through a column packed with molecular sieves under a positive pressure of Ar. Ethyl acetate (EtOAc, Fisher), methanol (MeOH, Fisher), ethanol (EtOH, 200 proof, Fisher) were used as reaction solvents as received. Argon gas (5.0 grade, AR 5.0UH-T, Praxair) was dispensed from size T cylinders. Gases were dispensed into 12" helium quality latex balloons (CTI Industries or Sigma-Aldrich). All other commercially available solvents and/or reagents were used as received, unless otherwise noted. Microwave reactions were performed in a Monowave 300 microwave reactor.

Solvents for workup and chromatography were hexanes (Fisher or EMD, ACS Grade), ethyl acetate (EtOAc, Fisher, ACS Grade), dichloromethane (CH_2Cl_2, Fisher, ACS Grade), chloroform ($CHCl_3$, Fisher, ACS Grade), and diethyl ether (Et_2O, Fisher, ACS Grade). Column chromatography was performed using EMD Millipore 60 Å (0.040–0.063 mm) mesh silica gel (SiO_2). Analytical thin-layer chromatography was performed on Merck silica gel 60 F254 TLC plates. Visualization was accomplished with UV (254 or 210 nm), and potassium permanganate ($KMnO_4$), cerium ammonium molybdate (CAM), or phosphomolybdic acid (PMA) staining solutions (unless otherwise noted). ^{1}H NMR and ^{13}C NMR spectra were recorded at 298 K on Bruker GN500 (499 MHz, ^{1}H), Bruker CRYO500 (500 MHz, ^{1}H; 126 MHz, ^{13}C), and Bruker AVANCE600 (600 MHz, ^{1}H; 150 MHz, ^{13}C) spectrometers. ^{1}H NMR spectra were referenced to residual chloroform (7.26 ppm) and ^{13}C NMR spectra were referenced to $CDCl_3$ (77.16 ppm). Chemical shifts are reported in ppm and multiplicities are indicated by s (singlet), d (doublet), t (triplet), q (quartet), m (multiplet), dd (doublet of doublets), dt (doublet of triplets), dq (doublet of

quartets), td (triplet of doublets), tt (triplet of triplets), ddd (doublet of doublet of doublets), and ddt (doublet of doublet of triplets). Coupling constants (*J* values) are reported in Hertz. The raw fid files were processed into the included NMR spectra using MestReNova 10.0 (Mestrelab Research S.L.). Mass spectrometry data were obtained from the University of California, Irvine Mass Spectrometry Facility. High-resolution mass spectra (HRMS) were recorded on a Waters LCT Premier spectrometer using ESI-S3 TOF (electrospray ionization-time of flight) or a Waters GCT Premier Micromass GC-MS (chemical ionization), and data are reported in the form of *m/z*.

B. Experimental Procedures

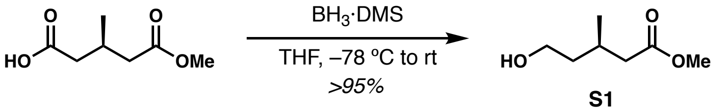

S1

Alcohol S1: BH_3·DMS (118 mL, 236 mmol, 2.0M in THF) was cannulated into a solution of (*R*)-1-methyl hydrogen 3-methylglutarate[1](19.0 g, 118 mmol) in THF (390 mL) at –78 °C. After addition, the flask was removed from the dry/ice acetone bath and slowly allowed to warm to rt. After 3 h, the reaction was cooled to 0 °C and quenched by careful addition of MeOH (~120 mL) until gas evolution was no longer observed. The volatiles were removed *in vacuo* to give **S1** (17.0 g, quant.) as a colorless oil, which was used without purification in the following step. The spectral data matched previous literature reports.[2]

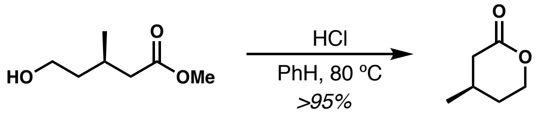

S1 28

Lactone 28: The crude alcohol **S1** (17.0 g, 118 mmol) was dissolved in benzene (390 mL) and HCl (2 mL of 12N HCl, 2.4 mmol) was added. The solution was heated to 80 °C in a Dean–Stark apparatus overnight. After 20 h, the solution was cooled to rt and concentrated *in vacuo*. The residue was then diluted with Et_2O (200 mL), dried with $MgSO_4$, filtered, and concentrated *in vacuo* to give crude lactone **28** (13.4 g, quant.), which was used in the following step without further purification. The spectral data matched previous literature reports.[3]

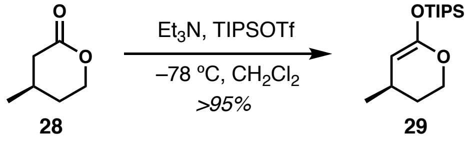

TIPS-SKA 29: TIPSOTf (3.2 mL, 12 mmol) was added dropwise to a solution of crude lactone **28** (1.14 g, 10 mmol) and Et_3N (2.8 mL, 20 mmol) in CH_2Cl_2 (50 mL) at –78 ºC. After the addition was complete, the –78 ºC bath was removed and the solution was allowed to warm to rt. After 1 h, the reaction was diluted with hexanes (100 mL) and brine (50 mL), the layers were separated, and the aqueous phase was extracted with hexanes (3 x 50 mL). The combined organic extracts were dried over $MgSO_4$, filtered, and concentrated *in vacuo*. The resulting TIPS silyl ketene acetal **29** (2.7 g, quant.) was used without purification in the following step.

^{1}H NMR (500 MHz, $CDCl_3$): δ 4.09 (ddd, *J* = 10.0, 6.2, 3.3 Hz, 1H), 3.99 (ddd, *J* = 10.9, 8.8, 2.7 Hz, 1H), 3.80 (d, *J* = 2.9 Hz, 1H), 2.36 (qt, *J* = 9.5, 4.7 Hz, 1H), 1.83 (dtd, *J* = 14.4, 6.1, 2.7 Hz, 1H), 1.39 (dddd, *J* = 13.5, 8.5, 7.3, 3.2 Hz, 1H), 1.25 – 1.15 (m, 3H), 1.09 (d, *J* = 7.2 Hz, 18H), 0.98 (d, *J* = 6.8 Hz, 3H).

^{13}C NMR (126 MHz, $CDCl_3$): δ 154.6, 80.9, 65.7, 31.1, 26.2, 23.0, 18.0, 12.7.

HRMS (CI): *m/z* calculated $C_{15}H_{30}O_2Si$ $[M+H]^+$ 270.2015, found 270.2025.

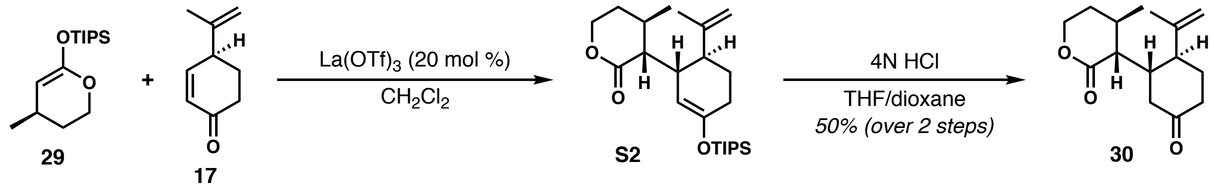

Enoxysilane S2: Enone[4] **17** (1.36 g, 10 mmol) was dissolved in CH_2Cl_2 (40 mL) and $La(OTf)_3$ (117 mg, 2 mmol) was added in one portion. The reaction was cooled to –78 ºC and crude silyl ketene acetal **29** (2.7 g, 10 mmol) in CH_2Cl_2 (40 mL) was added dropwise over 30 min. The reaction was allowed to warm to rt overnight. The reaction volume was filtered through a pad of Celite, washing with CH_2Cl_2 (100 mL), and the solvent was removed *in vacuo*. The crude yellow oil was purified by column chromatography (SiO_2, 10:1 hexanes/EtOAc) to provide TIPS enol ether adduct **S2** as a single diastereomer,

contaminated with TIPSOH, which was further purified after the next step.

^{1}H NMR (600 MHz, $CDCl_3$): δ 4.88 (d, *J* = 2.1 Hz, 1H), 4.85 (s, 1H), 4.59 (t, *J* = 2.2 Hz, 1H), 4.36 (ddd, *J* = 11.0, 7.6, 3.3 Hz, 1H), 4.26 (ddd, *J* = 11.0, 7.4, 3.5 Hz, 1H), 3.25 (d, *J* = 10.8 Hz, 1H), 2.40 (dd, *J* = 5.7, 2.7 Hz, 1H), 2.27 (td, *J* = 11.9, 5.8 Hz, 1H), 2.13 (dd, *J* = 11.5, 2.9 Hz, 1H), 2.10 – 2.01 (m, 1H), 1.89 (dddd, *J* = 13.1, 8.1, 5.2, 3.3 Hz, 1H), 1.81 – 1.74 (m, 1H), 1.73 – 1.62 (m, 1H), 1.57 – 1.49 (m, 1H), 1.31 – 1.25 (m, 1H), 1.21 – 1.11 (m, 3H), 1.09 – 1.07 (overlapping doublets, 18H and 3H).

^{13}C NMR (151 MHz, $CDCl_3$): δ 174.8, 152.7, 147.5, 112.7, 103.9, 66.6, 48.7, 45.1, 41.5, 31.6, 30.3, 29.5, 27.0, 22.7, 19.6, 18.1, 12.8.

HRMS (ESI): *m/z* calculated $C_{24}H_{42}O_3Si$ $[M+H]^+$ 407.2982, found 407.2986.

1,4-adduct 30: TIPS-enoxysilane **S2** was dissolved in THF (100 mL) and 4N HCl in dioxane (5 mL, 20 mmol) was added. The resulting solution was stirred at rt until disappearance of starting material by TLC (approx. 2–3 h). The reaction was diluted with Et_2O (100 mL), washed with $NaHCO_3$ (3 x 50 mL), and brine (50 mL). The organic extracts were dried over $MgSO_4$, filtered, and concentrated *in vacuo*. The crude oil was purified by column chromatography (SiO_2, 2:1 hexanes/EtOAc) to give ketone **30** (1.25 g, 50%) over 2 steps as a colorless oil.

^{1}H NMR (500 MHz, $CDCl_3$): δ 4.91 (s, 2H), 4.32 (dt, *J* = 11.2, 4.5 Hz, 1H), 4.20 (td, *J* = 10.5, 3.2 Hz, 1H), 2.86 (t, *J* = 13.5 Hz, 1H), 2.75 (td, *J* = 11.6, 3.5 Hz, 1H), 2.51 (dddd, *J* = 12.9, 11.2, 4.3, 1.6 Hz, 1H), 2.41 (dd, *J* = 10.1, 4.8 Hz, 2H), 2.34 (dd, *J* = 14.1, 4.3 Hz, 1H), 2.12 (dd, *J* = 8.9, 1.6 Hz, 1H), 1.97 (tq, *J* = 13.4, 4.3 Hz, 2H), 1.86 – 1.79 (m, 1H), 1.76 – 1.72 (m, 1H), 1.70 (s, 3H), 1.63 – 1.50 (m, 1H), 1.09 (d, *J* = 6.6 Hz, 3H).

^{13}C NMR (126 MHz, $CDCl_3$): δ 210.2, 172.5, 146.2, 114.5, 67.5, 51.4, 46.7, 46.5, 41.1, 40.8, 31.7, 31.2, 28.9, 21.1, 19.0.

HRMS (ESI): m/z calculated $C_{15}H_{22}O_3$ $[M+H]^+$ 251.1647, found 251.1652.

$[\alpha]^{22}_D$: +16.6 (*c* = 1, $CHCl_3$)

See Section E for additional spectra: COSY, HSQC, HMBC (optimized for 10Hz couplings), and NOESY.

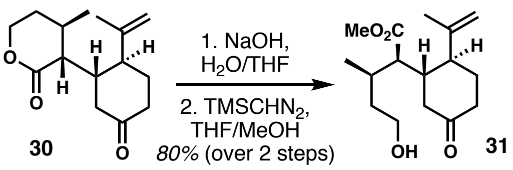

Alcohol 31: Lactone **30** (1.05 g, 4.19 mmol) was dissolved in THF (84 mL) and 1N aqueous NaOH (84 mL) was added. The solution stirred at rt overnight. The reaction was diluted with EtOAc and the pH of the aqueous layer was carefully adjusted to pH 5 using 1N HCl and extracted with EtOAc (5 x 50 mL). The combined organic extracts were dried over $MgSO_4$, filtered, and concentrated *in vacuo*. The crude acid was directly subjected to methylation without purification. The crude oil was dissolved in THF/MeOH (42 mL), cooled to 0 °C, and $TMSCHN_2$ (1.0M in hexanes, 4.19 mL, 8.38 mmol) was added dropwise. The reaction was allowed to warm to rt for 2 h before the volatiles were removed by concentrating *in vacuo*. The resulting bright yellow oil was purified by column chromatography (SiO_2, 3:1 hexanes/EtOAc) to give methyl ester **31** (945 mg, 80% over 2 steps) as a colorless oil.

^{1}H NMR (500 MHz, $CDCl_3$): δ 4.90 (s, 1H), 4.88 (s, 1H), 3.73 (ddd, J = 11.1, 6.9, 4.5 Hz, 1H), 3.63 (s, 3H), 3.63 – 3.56 (m, 1H), 2.76 – 2.59 (m, 1H), 2.51 – 2.37 (m, 4H), 2.40 – 2.31 (m, 1H), 2.32 – 2.21 (m, 1H), 2.00 – 1.93 (m, 1H), 1.89 – 1.77 (m, 1H), 1.69 (s, 3H), 1.69 – 1.61 (m, 2H), 1.35 – 1.23 (m, 1H), 1.03 (d, J = 6.8 Hz, 3H).

^{13}C NMR (126 MHz, $CDCl_3$): δ 211.1, 174.5, 146.2, 113.9, 60.8, 53.2, 51.3, 48.3, 42.4, 40.4, 40.1, 35.9, 30.2, 27.9, 19.3, 19.1.

HRMS (ESI): *m/z* calculated $C_{16}H_{26}O_4$ $[M+H]^+$ 283.1909, found 283.1898.

[α]^{22}$_D$: +72.0 (c = 1, $CHCl_3$)

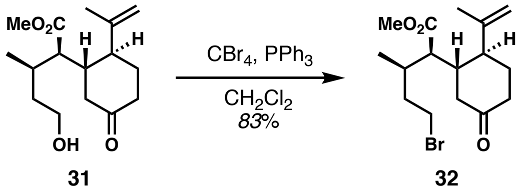

Alkyl bromide 32: Alcohol **31** (365 mg, 1.29 mmol) was dissolved in CH_2Cl_2 (12.9 mL) and cooled to 0 °C. Carbon tetrabromide (536 mg, 1.62 mmol) was added followed by triphenylphosphine (424 mg, 1.62 mmol) portionwise. After the starting material was

consumed by TLC (approx. 1 h), the volatiles were removed *in vacuo*. The crude bromide was purified by column chromatography (SiO_2, 10:1 hexanes/EtOAc) to give alkyl bromide **32** (370 mg, 83%) as a colorless solid.

^{1}H NMR (600 MHz, $CDCl_3$): δ 4.91 (t, *J* = 1.6 Hz, 1H), 4.89 (s, 1H), 3.63 (s, 3H), 3.54 (ddd, *J* = 10.6, 7.0, 3.9 Hz, 1H), 3.35 (td, *J* = 10.1, 6.0 Hz, 1H), 2.68 (dt, *J* = 14.9, 2.2 Hz, 1H), 2.49 – 2.39 (m, 4H), 2.41 – 2.32 (m, 1H), 2.30 (dd, *J* = 13.9, 9.2 Hz, 1H), 2.12 (ddd, *J* = 10.7, 6.8, 3.4 Hz, 1H), 1.97 (tdd, *J* = 15.3, 6.4, 4.4 Hz, 1H), 1.91 (ddd, *J* = 12.7, 7.2, 3.5 Hz, 1H), 1.89 – 1.78 (m, 1H), 1.73 – 1.63 (overlapping m, 1H, and s, 3H), 1.01 (d, *J* = 6.8 Hz, 3H).

^{13}C NMR (151 MHz, $CDCl_3$): δ 210.6, 174.1, 146.1, 113.8, 53.1, 51.4, 48.1, 42.5, 40.3, 39.9, 35.3, 32.1, 30.0, 29.8, 19.3, 18.3.z

HRMS (ESI): *m/z* calculated for $C_{16}H_{25}BrO_3$ $[M + Na]^+$ 367.0885, found 367.0879.

$[\alpha]^{22}_D$: –59.0 (*c* = 1, $CHCl_3$)

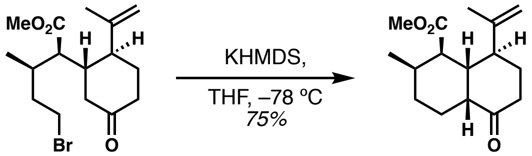

32 33

***cis*-Decalone 33:** Alkyl bromide **32** (200 mg, 0.58 mmol) was dissolved in THF (2 mL) and cooled to –78 °C. KHMDS (110 mg, 0.55 mmol) in THF (1 mL) was added dropwise (over 5 min). After 2.5 h, the reaction was quenched at –78 °C by addition of sat. aq. NH_4Cl (5 mL) and warmed to rt. The mixture was extracted with Et_2O (3 x 10 mL) and the combined organic extracts were dried over $MgSO_4$, filtered, and concentrated *in vacuo*. The crude oil was purified by column chromatography (SiO_2, 10:1 hexanes/EtOAc) to give **33** (115 mg, 75%, >20:1 dr) as a colorless oil.

^{1}H NMR (600 MHz, $CDCl_3$): δ 4.89 (p, *J* = 1.6 Hz, 1H), 4.88 – 4.86 (m, 1H), 3.63 (s, 3H), 2.85 (tdd, *J* = 8.4, 4.7, 2.4 Hz, 1H), 2.78 (td, *J* = 11.7, 3.6 Hz, 1H), 2.62 – 2.49 (m, 2H), 2.30 (dddd, *J* = 14.9, 4.4, 2.9, 1.4 Hz, 1H), 2.23 (ddd, *J* = 11.5, 5.1, 1.9 Hz, 1H), 1.93 (ddt, *J* = 13.0, 6.3, 3.2 Hz, 1H), 1.86 – 1.78 (m, 1H), 1.81 – 1.71 (m, 3H), 1.68 (overlapping s, 3H, and m, 1H), 0.95 (d, *J* = 6.6 Hz, 3H).

^{13}C NMR (151 MHz, $CDCl_3$): δ 214.1, 174.3, 146.1, 113.6, 51.3, 48.2, 46.1, 42.5, 40.7, 37.8, 30.9, 28.6, 28.0, 25.9, 19.7, 17.9.

HRMS (ESI): *m/z* calculated for $C_{16}H_{24}O_3$ $[M + Na]^+$ 287.1623, found 287.1633.

$[\alpha]^{22}_D$: –120.4 (c = 1, $CHCl_3$)

See Section E for additional spectra: COSY, HSQC, HMBC (optimized for 10Hz couplings), DEPTQ, and NOESY.

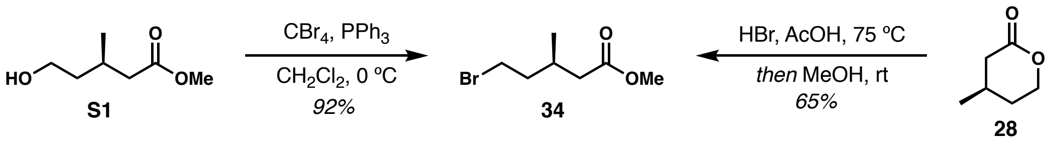

Alkyl Bromide 34:

Method A: Appel reaction for ≤12 mmol scale

Crude alcohol **S1** (12 mmol) was dissolved in CH_2Cl_2 (120 mL) and cooled to 0 °C. CBr_4 (4.97 g, 15 mmol) was added followed by PPh_3 (3.93 g, 15 mmol). The reaction was maintained at 0 °C until the starting material was consumed by TLC (approx. 1 h). The solvent was removed under reduced pressure and the crude oil was purified by column chromatography (SiO_2, 100% hexanes to 15:1 hexanes/EtOAc) to give alkyl bromide **34** (2.27 g, 92% over 2 steps) as a slightly yellow oil. The spectral data matched the previous literature report.[5]

Method B: Lactone opening for >12 mmol scale

To a 250 mL round bottom flask containing crude lactone **28** (13.4 g, 118 mmol) was added HBr in 33% acetic acid (30 mL). The flask was fitted with a reflux condenser and heated to 75 °C. After 4 h, the reaction was cooled to rt, the reflux condenser was removed, MeOH (54 mL) was added, and the mixture was left stirring overnight. The bulk of the solvent was removed and the residue diluted with EtOAc (250 mL), washed with sat. aq. $NaHCO_3$ (3 x 100 mL) and brine (100 mL). The organic extracts were dried over $MgSO_4$, filtered and concentrated *in vacuo* to give a brown oil which was purified by column chromatography (SiO_2, 15:1 hexanes/EtOAc) to afford bromide **28** (15.9 g, 65%) as a slightly yellow oil.

^{1}H NMR (500 MHz, $CDCl_3$): δ 3.68 (d, *J* = 1.2 Hz, 6H), 3.51 – 3.35 (m, 2H), 2.34 (q, *J* = 9.0 Hz, 1H), 2.25 – 2.11 (m, 2H), 2.01 – 1.88 (m, 1H), 1.77 (dq, *J* = 13.9, 7.2 Hz, 1H), 0.99 (d, *J* = 6.2 Hz, 3H).
^{13}C NMR (126 MHz, $CDCl_3$): δ 173.1, 51.7, 41.0, 39.4, 31.2, 29.3, 19.2.
HRMS (CI): *m/z* calculated for $C_7H_{13}BrO_2$ $[M+NH_4]^+$ 226.0443, found 226.0433
$[\alpha]^{22}_D$: –29.2 (*c* = 1, $CHCl_3$)

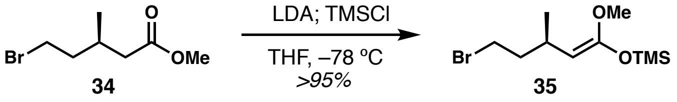

TMS-SKA 35: A solution of DIPA (2.0 mL, 14.38 mmol) in THF (20 mL) was cooled to 0 °C and *n*-BuLi (2.5M in hexanes, 5.5 mL, 13.75 mmol) was added dropwise. After 10 min at 0 °C, the solution was cooled to –78 °C and alkyl bromide **34** (2.6 g, 12.5 mmol) in THF (20 mL) was added dropwise over 15 min. The resulting orange solution was maintained at –78 °C for 30 additional min before TMSCl (freshly distilled, 1.9 mL, 15 mmol) was added dropwise. The resulting yellow solution was maintained at –78 °C for 1.5 h before the bath was removed and the reaction was allowed to warm to rt. After 30 min at rt, the volatiles were removed *in vacuo*. The resulting residue was diluted with hexanes (50 mL), causing diisopropylamine·HCl to precipitate, and the mixture was filtered over cotton. The filtrate was concentrated *in vacuo* and the crude yellow oil, **35** (~3.5 g, quant.) was used without purification in the Mukaiyama–Michael addition. The silyl ketene acetal could be purified by Kugelrohr distillation (85–95 °C at 150 mTorr) to afford a colorless oil; however, we observed no difference in reactivity upon using crude SKA.
^{1}H NMR (500 MHz, $CDCl_3$): δ 3.52 (s, 3H), 3.43 (d, *J* = 9.4 Hz, 1H), 3.43 – 3.38 (m, 1H), 3.38 – 3.33 (m, 1H), 2.56 (tddd, *J* = 8.9, 6.8, 4.7, 2.2 Hz, 1H), 1.87 (dddd, *J* = 13.6, 8.4, 7.3, 5.0 Hz, 1H), 1.71 (dddd, *J* = 13.6, 9.3, 8.2, 5.4 Hz, 1H), 0.98 (d, *J* = 6.8 Hz, 3H), 0.24 (s, 9H).
^{13}C NMR (126 MHz, $CDCl_3$): δ 153.9, 89.8, 54.9, 41.5, 32.7, 29.2, 22.2, -0.1.
HRMS (CI): *m/z* calculated for $C_7H_{13}BrO_2$ $[M - C_3H_8Si + H]^+$ 209.0177, found 209.0167.

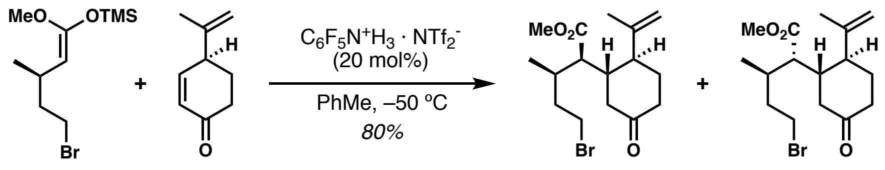

35 17 32 S3

Mukaiyama–Michael products 32 and S3: Enone **17** (1.48 g, 10.87 mmol) was dissolved in PhMe (5.4 mL) and cooled to –50 °C. The catalyst, $C_6F_5NH_3^+{\cdot}NTf_2^-$ (prepared as reported by Tanabe,[6] 1.01 g, 2.17 mmol) was added in one portion resulting in a bright yellow mixture. Next, crude silyl ketene acetal **35** (3.52 g, 12.5 mmol) was added dropwise and the reddish-brown solution was left at –50 °C overnight. After 20 h, the reaction was quenched at –50 °C by addition of (1:1 Et_2O/H_2O, 4 mL) and warmed to rt. The mixture was further diluted with Et_2O (30 mL), washed with water (15 mL), and brine (15 mL). The combined organic extracts were dried over $MgSO_4$, filtered, and concentrated *in vacuo*. The crude oil was purified by column chromatography (SiO_2, 10:1 hexanes/EtOAc) to give **32** and **S3** (2.99 g, 80%, 1.5:1 dr) as a yellow solid.

Diastereomer 32: See above for characterization data (**30** to **32**)

Diastereomer S3: Spectral data were obtained from purification after the subsequent cyclization step because **S3** is unreactive upon treatment with KHMDS.

^{1}H NMR (500 MHz, $CDCl_3$): δ 4.91 (t, *J* = 1.9 Hz, 1H), 4.77 (s, 1H), 3.68 (s, H), 3.46 (ddd, *J* = 10.1, 7.4, 4.8 Hz, 1H), 3.34 (dt, *J* = 10.0, 7.5 Hz, 1H), 2.74 (dd, *J* = 14.6, 12.3 Hz, 1H), 2.51 (dd, *J* = 14.6, 4.1 Hz, 1H), 2.43 (dd, *J* = 9.2, 3.7 Hz, 1H), 2.39 – 2.34 (m, 2H), 2.25 (td, *J* = 11.2, 3.5 Hz, 1H), 2.15 (tt, *J* = 11.3, 4.2 Hz, 1H), 2.08 – 1.91 (m, 4H), 1.69 (s, 3H), 1.63 – 1.49 (m, 1H), 0.86 (d, *J* = 6.6 Hz, 3H).

^{13}C NMR (126 MHz, $CDCl_3$): δ 210.9, 174.1, 145.8, 113.0, 51.4, 51.1, 47.5, 41.3, 40.9, 39.4, 36.7, 31.7, 31.1, 30.7, 19.8, 17.2.

HRMS (ESI): *m/z* calculated for $C_{16}H_{25}BrO_3$ [M + Na]$^+$ 367.0885, found 367.0879.

$[\alpha]^{22}_D$: –40.0 (*c* = 1, $CHCl_3$)

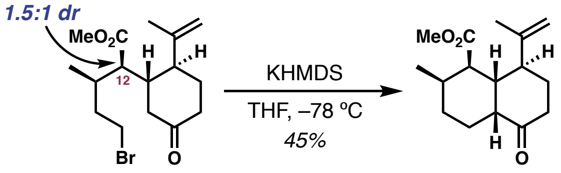

32 30

***cis*-Decalone 33:** A mixture of alkyl bromides **32** and **S2** (9.4 g, 27.2 mmol) was dissolved in THF (75 mL) and cooled to –78 °C. KHMDS (5.15 g, 25.84 mmol) in THF (60 mL) was added dropwise (over 15 min). After 2.5 h, the reaction was quenched at –78 °C by addition of sat. aq. NH_4Cl (25 mL) and warmed to rt. The mixture was extracted with Et_2O (3 x 100 mL) and the combined organic extracts were washed with brine (50 mL), dried over $MgSO_4$, filtered, and concentrated *in vacuo*. The crude oil was purified by column chromatography (SiO_2, 10:1 hexanes/EtOAc) to give **30** (3.2 g, 45%, >20:1 dr) as a colorless oil. The undesired diastereomer of starting material, **S3**, could also be isolated at this stage if desired. See above for characterization data.

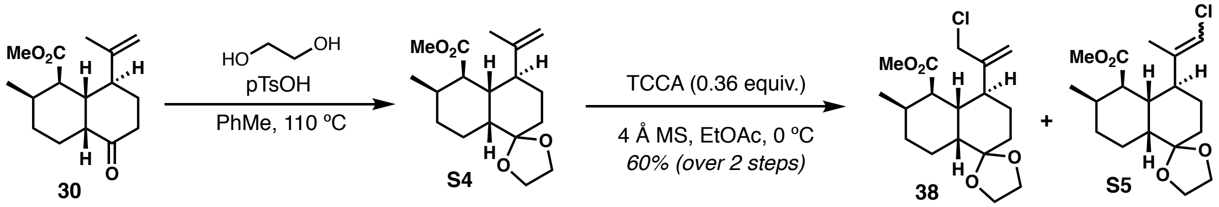

30 S4 38 S5

Ketal S4: Decalone **30** (3 g, 11.35 mmol), ethylene glycol (6.35 mL, 113.5 mmol), and *p*-TsOH·H_2O (216 mg, 1.14 mmol) was dissolved in PhMe (76 mL), affixed with a Dean–Stark apparatus, and heated to reflux (120 °C) overnight. After 16 h, the reaction was cooled to rt, diluted with EtOAc (50 mL), washed with water (2 x 75 mL), and extracted with EtOAc (3 x 50 mL). The combined organic extracts were washed with brine (50 mL), dried over $MgSO_4$, filtered and concentrated *in vacuo*. The crude yellow oil (3.45 g) was used in the next step without further purification. A small portion of the crude reaction mixture was purified by column chromatography (SiO_2, 10:1 hexanes/EtOAc) to give an analytical standard (data below) of **S4** as a yellow oil.

^{1}H NMR (600 MHz, $CDCl_3$): δ 4.78 (d, J = 1.8 Hz, 1H), 4.76 (d, J = 2.3 Hz, 1H), 4.04 – 3.88 (m, 4H), 3.63 (s, 3H), 2.52 (d, J = 3.6 Hz, 1H), 2.44 (td, J = 12.1, 3.8 Hz, 1H), 2.12 (ddd, J = 22.4, 12.6, 4.1 Hz, 2H), 1.84 – 1.69 (m, 4H), 1.67 (s, 3H), 1.66 – 1.62 (m, 1H),

1.57 (dtd, J = 13.3, 5.1, 2.2 Hz, 2H), 1.53 – 1.49 (m, 1H), 1.43 (qd, J = 12.7, 4.6 Hz, 1H), 0.90 (d, J = 5.9 Hz, 3H).

^{13}C NMR (151 MHz, $CDCl_3$): δ 175.1, 147.6, 112.6, 110.8, 64.4, 64.2, 51.1, 46.4, 42.5, 39.7, 38.7, 30.3, 29.2, 29.2, 28.1, 23.3, 19.9, 17.8.

HRMS (CI): *m/z* calculated $C_{18}H_{28}O_4$ $[M]^+$ 308.1988, found 308.1978.

$[\alpha]^{22}_D$: –45.4 (*c* = 2, $CHCl_3$)

Allylic Chloride 38: Crude alkene **S4** (3.45 g) was dissolved in EtOAc (220 mL, previously degassed over 4 Å mol sieves.), 4 Å mol sieves were added, and the mixture was stirred at rt for 15 min before cooling to 0 °C. Trichloroisocyanuric acid (TCCA, 936 mg, 4.03 mmol) was added in one portion. After disappearance of starting material by TLC (approx. 30 min), the reaction was quenched at 0 °C by addition of sat. aq. $Na_2S_2O_3$ (50 mL) and extracted with EtOAc (3 x 100 mL). The combined organic extracts were washed with brine (50 mL), dried over $MgSO_4$, filtered and concentrated *in vacuo*. The crude yellow oil was purified by column chromatography (SiO_2, 10:1 to 5:1 hexanes/EtOAc) to give **38** (2.33 g, 60%, corrected for ~65% purity with **S35** as the impurity). An analytic standard of both products was obtained after careful purification to give the data below; however, the alkenyl chloride impurity was typically carried through to the next reaction.

^{1}H NMR (500 MHz, $CDCl_3$): δ 5.32 (s, 1H), 5.14 (s, 1H), 4.05 (dd, J = 6.0, 1.0 Hz, 2H), 3.94 (qd, J = 4.5, 2.3 Hz, 5H), 3.63 (s, 1H), 2.65 (d, J = 4.7 Hz, 1H), 2.58 – 2.47 (m, 1H), 2.29 – 2.13 (m, 2H), 1.87 – 1.74 (m, 4H), 1.75 – 1.65 (m, 2H), 1.58 – 1.53 (m, 3H), 1.44 (td, J = 13.0, 4.1 Hz, 1H), 0.91 (d, J = 6.9 Hz, 4H).

^{13}C NMR (126 MHz, $CDCl_3$): δ 174.9, 147.6, 115.6, 110.5, 64.4, 64.2, 51.1, 46.2, 40.1, 40.0, 38.5, 31.5, 30.4, 29.2, 28.6, 23.3, 19.9, 19.8.

HRMS (CI): *m/z* calculated $C_{18}H_{27}ClO_4$ $[M]^+$ 342.1598, found 342.1597.

$[\alpha]^{22}_D$: –31.9 (*c* = 1, $CHCl_3$)

Alkenyl chloride S5: 1.6:1 mixture of *E*- and *Z*-isomers

^{1}H NMR (500 MHz, $CDCl_3$): δ 5.91 (d, J = 1.5 Hz, 1H, one isomer), 5.88 (d, J = 1.6 Hz, 1H, one isomer), 3.94 (td, J = 4.4, 2.1 Hz, 4H), 3.64 and 3.63 (overlapping s, 3H), 2.49 (ddd, J = 23.5, 11.9, 3.9 Hz, 1H), 2.35 (dd, J = 21.2, 4.7 Hz, 1H), 2.21 – 2.10 (m, 2H), 1.88 – 1.76 (m, 2H), 1.74 and 1.69 (d, J = 1.4 Hz and J = 1.3 Hz, 3H), 1.68 – 1.61 (m, 2H), 1.60 – 1.56 (m, 1H), 1.57 – 1.48 (m, 6H), 1.41 (td, J = 13.1, 4.1 Hz, 1H), 0.91 (overlapping d, J = 6.5 Hz, 3H).

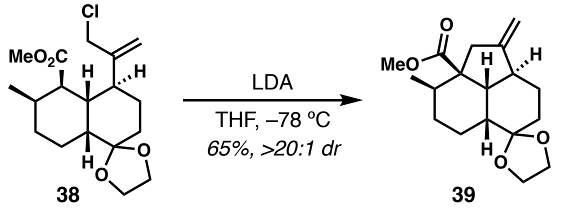

Tricycle 39: LDA was generated at 0 °C by addition of *n*-BuLi (2.5M in hexanes, 4.24 mL, 10.59 mmol) to a solution of DIPA (1.56 mL, 11.1 mmol) in THF (50 mL). The solution was allowed to stir at 0 °C for 20 min before cooling to –78 °C. In a separate flask, allylic chloride **38** (1.73 g, ~60% purity, 3.03 mmol) in THF (50 mL) was cooled to –78 °C. The LDA solution was transferred into the reaction flask by a dropwise cannula addition. After 1 h, the reaction was quenched with sat. aq. NH_4Cl (50 mL), warmed to rt, and extracted with Et_2O (3 x 100 mL). The combined organic extracts were washed with brine (50 mL), dried over $MgSO_4$, filtered, and concentrated *in vacuo*. The crude product was purified by column chromatography (SiO_2, 10:1 hexanes/EtOAc) to give **39** (615 mg, 65%, >20:1 dr) as a colorless oil.

^{1}H NMR (600 MHz, $CDCl_3$): δ 4.79 (q, J = 2.4 Hz, 1H), 4.70 (q, J = 2.6 Hz, 1H), 3.98 – 3.93 (m, 4H), 3.66 (s, 3H), 3.00 (dq, J = 17.9, 2.1 Hz, 1H), 2.59 – 2.47 (m, 2H), 2.36 (dt, J = 12.7, 4.7 Hz, 1H), 1.94 (ddd, J = 13.7, 7.8, 3.9 Hz, 2H), 1.77 – 1.73 (m, 1H), 1.73 – 1.68 (m, 1H), 1.68 – 1.62 (m, 1H), 1.58 – 1.51 (m, 1H), 1.47 (td, J = 12.5, 4.7 Hz, 1H), 1.33 (td, J = 12.4, 4.0 Hz, 1H), 1.30 – 1.25 (m, 1 H), 0.93 – 0.89 (m, 1H), 0.84 (d, J = 6.8 Hz, 3H).

^{13}C NMR (126 MHz, $CDCl_3$): δ 175.8, 151.2, 111.5, 103.8, 64.6, 64.3, 52.8, 51.3, 49.1, 41.3, 41.1, 40.5, 35.6, 30.8, 30.7, 25.2, 22.6, 17.3.

HRMS (CI): *m/z* calculated $C_{18}H_{26}O_4$ $[M]^+$ 306.1831, found 306.1826.

[α]$^{22}_{D}$: –21.5 (*c* = 1, $CHCl_3$)

See Section E for NOESY spectrum.

Synthesis of 7,8-di-*epi*-isoneoamphilectane (via Shenvi isocyanation)

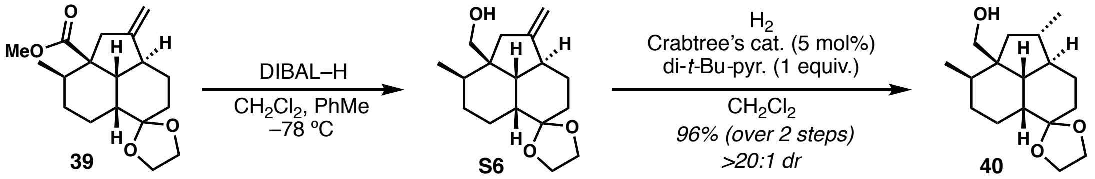

Alcohol S6: DIBAL–H (0.18 mL, 1.01 mmol) in PhMe (1 mL) was added to a solution of ester **39** (107 mg, 0.35 mmol) in CH_2Cl_2 (1.75 mL) at –78 °C. After 2 h, the reaction was diluted with Et_2O (10 mL) and quenched with H_2O (40 mL), 1N NaOH (40 mL), and an additional portion of H_2O (100 mL). After stirring for 15 min at rt, $MgSO_4$ was added and the mixture stirred for another 15 min before filtering through a fritted funnel. The aluminum salts were washed with Et_2O (20 mL) and the filtrate was concentrated *in vacuo*. The viscous pale-yellow oil, alcohol **S6** (96 mg), was used in the next step without purification.

^{1}H NMR (500 MHz, $CDCl_3$): δ 4.77 (s, 1H), 4.70 (d, *J* = 3.2 Hz, 1H), 4.00 – 3.89 (m, 4H), 3.68 (d, *J* = 11.0 Hz, 1H), 3.57 (d, *J* = 11.0 Hz, 1H), 2.54 (t, *J* = 12.8 Hz, 1H), 2.33 (s, 1H), 1.98 – 1.89 (m, 1H), 1.86 (dd, *J* = 13.3, 4.6 Hz, 1H), 1.75 – 1.62 (m, 4H), 1.63 – 1.42 (m, 2H), 1.39 – 1.32 (m, 2H), 0.93 –0.86 (overlapping m, 1H, and d, *J* = 7.1 Hz, 3H).

^{13}C NMR (126 MHz, $CDCl_3$): δ 152.6, 111.5, 103.7, 64.5, 64.3, 64.1, 47.4, 44.7, 42.0, 40.6, 39.9, 35.0, 31.2, 30.7, 25.7, 22.9, 16.4.

HRMS (ESI): *m/z* calculated for $C_{18}H_{24}O_4$ $[M + Na]^+$ 329.1729, found 329.1706.

Hydrogenated Tricycle 40: Crabtree's catalyst (13.6 mg, 17 μmol) was added to a solution of alcohol **S6** (96 mg, 0.34 mmol) and di-*tert*-butylpyridine (76 μL, 0.34 mmol) in CH_2Cl_2 (6 mL). The solution was sparged with hydrogen for 10 min. The reaction was allowed to stir under a hydrogen balloon atmosphere overnight (approx. 12 h) before the solvent was removed by concentrating *in vacuo*. The crude product was purified by column chromatography (SiO_2, 2:1 hexanes/EtOAc) to give **40** (94 mg, 96%, >20:1 dr) as

a pale-yellow viscous oil.

^{1}H NMR (500 MHz, $CDCl_3$): δ 4.02 – 3.89 (m, 4H), 3.63 (d, *J* = 11.0 Hz, 1H), 3.50 (d, *J* = 10.9 Hz, 1H), 1.91 (dd, *J* = 13.5, 9.5 Hz, 1H), 1.87 – 1.77 (m, 3H), 1.73 – 1.64 (m, 1H), 1.65 – 1.58 (m, 2H), 1.57 – 1.43 (m, 5H), 1.38 (td, *J* = 12.7, 3.2 Hz, 1H), 1.34 – 1.25 (m, 1H), 1.22 – 1.12 (m, 1H), 1.07 (dd, *J* = 13.5, 8.2 Hz, 1H), 0.98 (d, *J* = 6.3 Hz, 3H), 0.85 (d, *J* = 7.0 Hz, 3H).

^{13}C NMR (126 MHz, $CDCl_3$): δ 111.8, 64.8, 64.4, 64.2, 48.1, 45.7, 43.7, 43.2, 42.3, 38.5, 38.3, 31.7, 30.9, 27.1, 23.7, 19.7, 16.2.

HRMS (ESI): *m/z* calculated for $C_{17}H_{28}O_3$ $[M + Na]^+$ 303.1936, found 303.1940.

$[\alpha]^{22}_D$: +46.3 (c = 1, $CHCl_3$)

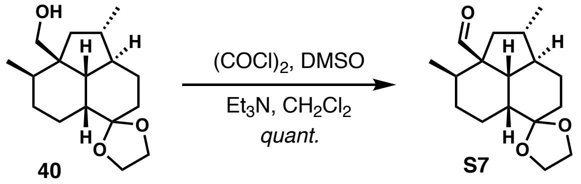

Aldehyde S7: Oxalyl chloride (8 μL, 86 μmol) in CH_2Cl_2 (0.1 mL) was cooled to –78 °C and a solution of DMSO (12 μL, 170 μmol) in CH_2Cl_2 (0.1 mL) was added dropwise (gas evolution was observed). After 15 min, a solution of alcohol **40** (20 mg, 71 μmol) in CH_2Cl_2 (0.5 mL) was added dropwise. After 45 min, Et_3N (50 μL, 360 μmol) was added dropwise. The solution was maintained at –78 °C for 30 min before warming to rt for an additional 30 min. Upon completion by TLC (approx. 2 hours), the reaction was diluted with CH_2Cl_2 (5 mL), washed with H_2O (5 mL), and extracted with CH_2Cl_2 (3 x 5 mL). The organic extracts were dried over Na_2SO_4, filtered, and concentrated *in vacuo* to give aldehyde **S7** (19.5 mg, 99%) as a colorless oil which was used in the following step without purification.

^{1}H NMR (600 MHz, $CDCl_3$): δ 9.66 (d, *J* = 1.1 Hz, 1H), 4.12 – 3.72 (m, 4H), 2.43 (dd, *J* = 14.0, 9.4 Hz, 1H), 1.92 – 1.84 (m, 2H), 1.83 (dq, *J* = 12.5, 3.7 Hz, 1H), 1.75 (dd, *J* = 12.7, 3.8 Hz, 1H), 1.67 – 1.61 (m, 4H), 1.56 – 1.50 (m, 2H), 1.50 – 1.43 (m, 1H), 1.39 (qd, *J* = 12.6, 3.1 Hz, 1H), 1.25 – 1.12 (m, 2H), 1.02 (d, *J* = 6.2 Hz, 3H), 0.97 (d, *J* = 6.9 Hz, 3H).

^{13}C NMR (151 MHz, $CDCl_3$): δ 205.7, 111.3, 64.5, 64.3, 56.9, 48.4, 43.5, 42.0, 41.0, 38.5, 37.9, 31.7, 31.1, 26.7, 23.4, 19.4, 16.3.

HRMS (ESI): *m/z* calculated for $C_{17}H_{26}O_3$ $[M + Na]^+$ 301.1780, found 301.1784.

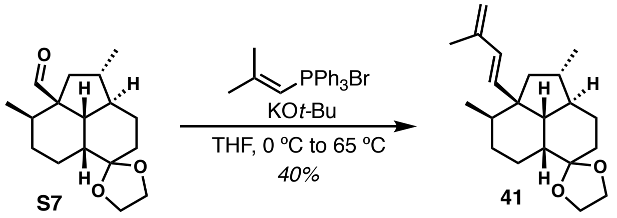

Diene 41: KO*t*-Bu (22.2 mg, 0.20 mmol) in THF (0.3 mL) was added to a suspension of the alkenylphosphonium salt (93.9 mg, 0.24 mmol) in THF (0.3 mL) at 0 °C. The resulting orange suspension was stirred for 1 h before cooling to –78 °C. Aldehyde **S7** (11 mg, 40 μmol) in THF (0.3 mL) was added dropwise. The reaction was allowed to warm to rt and then heated to reflux overnight (14 h). After cooling to rt, the reaction was quenched with sat. aq. NH_4Cl (1 mL) and H_2O (1 mL) and extracted with EtOAc (3 x 5 mL). The organic extracts were washed with brine (5 mL), dried over $MgSO_4$, filtered, and concentrated *in vacuo*. The crude diene was purified by column chromatography (SiO_2, 15:1 hexanes/EtOAc) to give **41** (5 mg; 40%) as a yellow oil.

^{1}H NMR (500 MHz, $CDCl_3$): δ 6.15 (d, *J* = 16.2 Hz, 1H), 5.85 (d, *J* = 16.1 Hz, 1H), 4.89 (s, 2H), 3.95 (q, *J* = 1.1 Hz, 4H), 2.10 (dd, *J* = 13.5, 9.4 Hz, 1H), 1.91 (dt, *J* = 13.0, 4.8 Hz, 1H), 1.85 (s, 3H), 1.83 – 1.76 (m, 1H), 1.68 (t, *J* = 4.4 Hz, 1H), 1.66 (t, *J* = 4.5 Hz, 1H), 1.65 – 1.61 (m, 1H), 1.57 (dd, *J* = 11.4, 3.5 Hz, 2H), 1.48 (dq, *J* = 13.4, 3.3 Hz, 1H), 1.43 – 1.38 (m, 1H), 1.14 – 1.05 (m, 2H), 1.00 (d, *J* = 6.2 Hz, 3H), 0.92 – 0.84 (m, 2H), 0.78 (overlapping d, *J* = 6.8 Hz, 3H and m, 1H).

^{13}C NMR (126 MHz, $CDCl_3$): δ 142.7, 134.3, 130.6, 114.3, 111.8, 64.4, 64.2, 52.4, 47.2, 45.7, 43.5, 41.8, 38.9, 37.9, 31.4, 31.1, 27.1, 23.9, 19.8, 19.0, 16.7.

HRMS (CI): *m/z* calculated for $C_{21}H_{32}O_2$ $[M + H]^+$ 317.2480, found 317.2464.

$[\alpha]^{21}_D$: +55.6 (c = 1, $CHCl_3$)

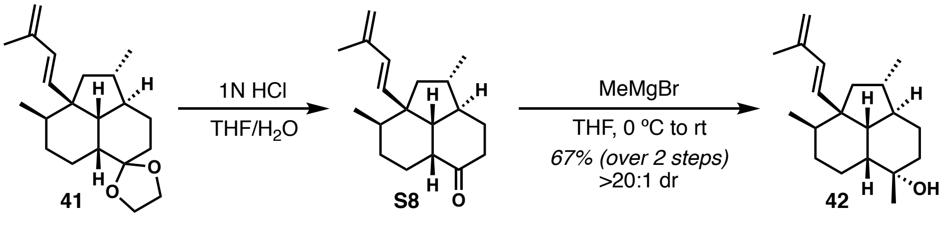

Ketone S8: Ketal **41** (5 mg, 16 μmol) was dissolved in THF (0.32 mL) and 1N aq. HCl (0.16 mL) was added. After 20 h, the solution was diluted with EtOAc (5 mL), quenched with sat. aq. $NaHCO_3$ (5 mL) and the resulting mixture was extracted with EtOAc (2 x 2 mL). The organic extracts were washed with brine (5 mL), dried over $MgSO_4$, filtered, and concentrated *in vacuo*. The resulting ketone **S8** was used in the next step without purification.

^{1}H NMR (600 MHz, $CDCl_3$): δ 6.12 (d, *J* = 16.1 Hz, 1H), 5.79 (d, *J* = 16.2 Hz, 1H), 4.91 (s, 2H), 2.68 (dt, *J* = 12.1, 5.9 Hz, 1H), 2.44 (ddd, *J* = 15.6, 13.6, 6.3 Hz, 1H), 2.36 – 2.30 (m, 1H), 2.18 (dd, *J* = 13.6, 10.0 Hz, 1H), 2.14 (dt, *J* = 12.6, 3.3 Hz, 1H), 1.96 (ddd, *J* = 21.8, 11.5, 3.4 Hz, 1H), 1.91 – 1.86 (m, 1H), 1.83 (s, 3H), 1.71 – 1.64 (m, 1H), 1.66 – 1.59 (m, 2H), 1.51 (dq, *J* = 13.5, 3.2 Hz, 1H), 1.43 – 1.38 (m, 1H), 1.34 – 1.28 (m, 1H), 1.08 (d, *J* = 6.2 Hz, 3H), 0.89 – 0.82 (m, 2H), 0.82 (d, *J* = 6.9 Hz, 3H).

^{13}C NMR (151 MHz, $CDCl_3$): δ 215.7, 142.3, 133.5, 131.0, 115.1, 54.4, 50.1, 46.8, 46.1, 43.4, 39.1, 38.0, 30.8, 28.8, 26.4, 22.9, 19.7, 18.9, 16.7.

HRMS (CI): *m/z* calculated for $C_{19}H_{28}O$ $[M]^+$ 272.2140, found 272.2129.

Tertiary alcohol 42: Methylmagnesium bromide (27 μL, 80 μmol, ~3M in diethyl ether) was added to a solution of crude ketone in THF (0.8 mL) at 0 °C. After 30 min at 0 °C, the reaction was allowed to warm to rt. After 1.5 h at rt, the reaction was quenched with sat. aq. NH_4Cl (2 mL) and extracted with EtOAc (3 x 5 mL). The combined organic extracts were washed with brine (5 mL), dried over $MgSO_4$, filtered, and concentrated *in vacuo*. The crude oil was purified by column chromatography (SiO_2, 5:1 pentane/EtOAc) to give a single diastereomer (>20:1 dr) of tertiary alcohol **42** (3.1 mg; 67% over 2 steps).

^{1}H NMR (500 MHz, $CDCl_3$): δ 6.14 (d, *J* = 16.1 Hz, 1H), 5.87 (d, *J* = 16.1 Hz, 1H), 5.06 – 4.62 (m, 2H), 2.14 – 2.01 (m, 1H), 1.85 (s, 3H), 1.81 (dq, *J* = 10.4, 3.5 Hz, 2H), 1.78 – 1.71 (m, 1H), 1.60 (t, *J* = 3.6 Hz, 1H), 1.58 – 1.56 (m, 1H), 1.53 – 1.49 (m, 3H), 1.40 (dd, *J* = 12.9, 3.3 Hz, 2H), 1.33 (s, 3H), 1.31 – 1.28 (m, 1H), 1.26 – 1.21 (m, 2H), 1.13 (td, *J* = 13.4, 3.2 Hz, 2H), 1.00 (d, *J* = 6.3 Hz, 3H), 0.77 (d, *J* = 6.6 Hz, 3H).

^{13}C NMR (151 MHz, $CDCl_3$): δ 142.8, 134.7, 130.5, 114.5, 73.8, 52.8, 47.5, 45.4, 44.6, 44.1, 38.7, 38.0, 36.3, 31.6, 28.5, 27.8, 22.7, 19.9, 19.0, 16.8.

HRMS (CI): *m/z* calculated for $C_{20}H_{32}O$ $[M]^+$ 288.2453, found 288.2453.

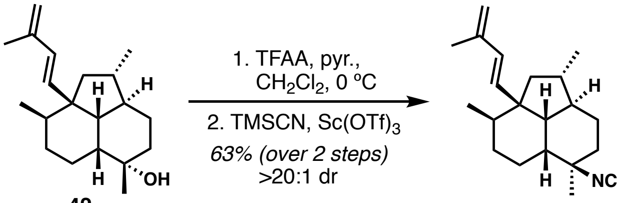

42

7,8-di-epi-isoneoamphilectane (43)

7,8-di-*epi*-isoneoamphilectane (43): A solution of tertiary alcohol **42** (3.1 mg, 10.7 μmol) in CH_2Cl_2 (0.5 mL) was cooled to 0 °C. Pyridine (7 μL, 86 μmol) and trifluoroacetic anhydride (TFAA; 6 μL, 43 μmol) were added sequentially. After 30 min, the reaction was quenched by addition of 0.5N HCl (0.25 mL), allowed to warm to rt, and stirred vigorously for 5 min. The mixture was extracted with CH_2Cl_2 (3 x 2 mL) and the combined organic extracts were washed with H_2O (2 mL), sat. aq. $NaHCO_3$ (2 mL), and brine (2 mL). The organic extracts were dried over $MgSO_4$, filtered, and concentrated *in vacuo* to give the crude trifluoroacetate which was used without purification. A solution of $Sc(OTf)_3$* in TMSCN (0.5M, 40 μL, 2 μmol) was added to a solution of crude trifluoroacetate in TMSCN (40 μL) at 0 °C. The solution solidified at 0 °C and was allowed to warm to rt. After 45 h, the reaction was quenched with TMEDA (1 drop), diluted with EtOAc (5 mL), and sat. aq. $NaHCO_3$ (5 mL) was added. The biphasic mixture was extracted with EtOAc (3 x 5 mL) and the combined organic extracts were dried over $MgSO_4$, filtered, and concentrated *in vacuo*. The crude oil was purified by column chromatography (SiO_2, 20:1 hexanes/EtOAc) to give a single diastereomer (>20:1) of isonitrile **43** (2 mg, 63% over 2 steps) a colorless film.

*Note: the solid $Sc(OTf)_3$ was briefly dried by heating with a torch under vacuum (ca. 200 mTorr).

^{1}H NMR (500 MHz, $CDCl_3$): δ 6.18 (d, *J* = 16.2 Hz, 1H), 5.82 (d, *J* = 16.1 Hz, 1H), 4.93 (dd, *J* = 12.9, 2.1 Hz, 2H), 2.11 – 1.96 (m, 3H), 1.86 (s, 3H), 1.83 – 1.77 (m, 1H), 1.65 – 1.57 (m, 1H), 1.53 – 1.44 (m, 6H), 1.40 (s, 3H), 1.27 – 1.22 (m, 2H), 1.23 – 1.17 (m, 1H), 1.14 (td, *J* = 13.0, 2.8 Hz, 1H), 1.01 (d, *J* = 6.6 Hz, 3H), 0.80 (d, *J* = 6.7 Hz, 3H).

^{13}C NMR (151 MHz, $CDCl_3$): δ 142.4, 133.4, 131.0, 114.9, 61.9, 49.4, 47.6, 45.3, 43.5, 43.2, 38.4, 37.9, 34.6, 31.5, 27.5, 26.3, 23.2, 19.7, 18.9, 16.5.

HRMS (CI): *m/z* calculated for $C_{21}H_{31}N$ $[M]^+$ 297.2456, found 297.2448.

$[\alpha]^{22}_D$: +34.2 (c = 0.4 $CHCl_3$)

Synthesis of 7,8-di-*epi*-isoneoamphilectane (via Tada isocyanation)

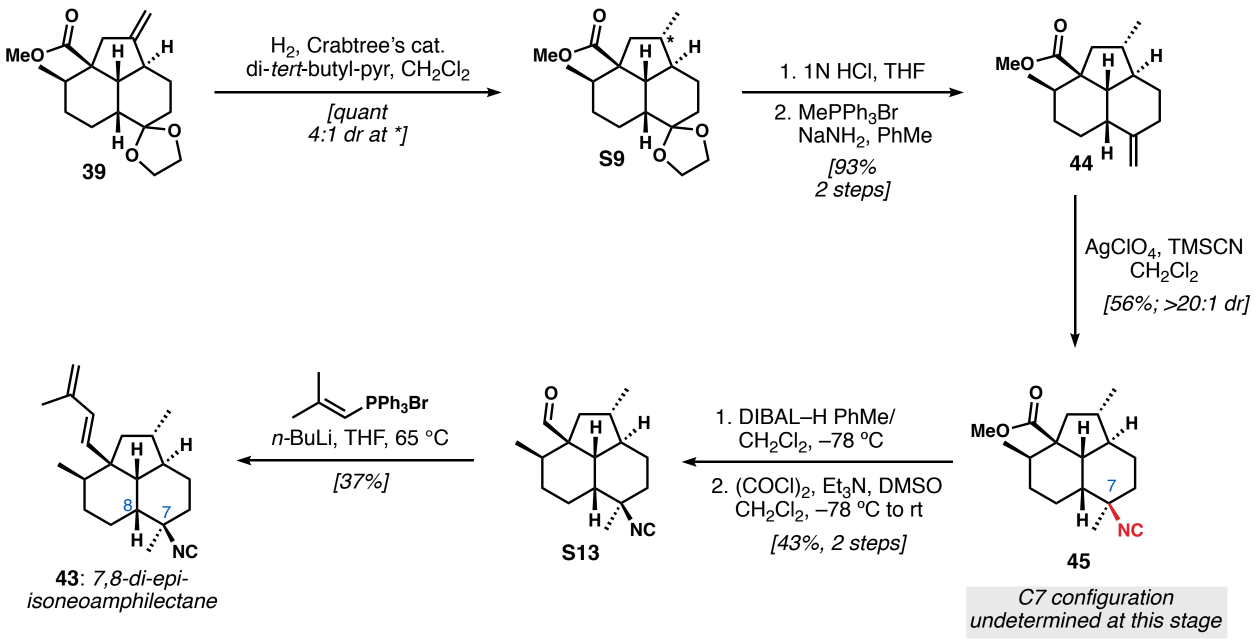

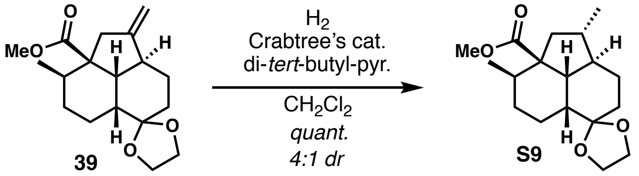

Hydrogenated Tricycle S9: Crabtree's catalyst (6.6 mg, 8 μmol) in CH_2Cl_2 (1 mL) was added to a solution of alkene **39** (50 mg, 0.16 mmol) and di-*tert*-butylpyridine (16 μL, 0.16 mmol) in CH_2Cl_2 (2.2 mL). The orange solution was sparged with hydrogen gas for 5 min. After 2 h under a balloon atmosphere of hydrogen, the reaction was exposed to air and the volatiles were removed *in vacuo*. The crude material was purified by column chromatography (SiO_2, 5:1 hexanes/EtOAc) to give a 4:1 mixture of inseparable diastereomers of **S9** (49 mg, quant.) as a colorless oil. The product was isolated as an

inseparable mixture of diastereomers (4:1) but the peaks for the major diastereomer at C3 are reported below.

^{1}H NMR (500 MHz, CDCl$_3$): δ 3.98 – 3.91 (m, 4H), 3.65 (d, *J* = 1.9 Hz, 3H), 2.74 (dd, *J* = 14.0, 9.4 Hz, 1H), 2.24 (dt, *J* = 12.8, 4.6 Hz, 1H), 1.89 (dd, *J* = 12.1, 4.9 Hz, 1H), 1.80 (dq, *J* = 12.4, 3.5 Hz, 1H), 1.76 – 1.68 (m, 1H), 1.71 – 1.64 (m, 1H), 1.66 – 1.58 (m, 2H), 1.57 – 1.46 (m, 5H), 1.44 – 1.31 (m, 1H), 1.19 (dd, *J* = 14.0, 8.8 Hz, 1H), 1.18 – 1.09 (m, 1H), 0.99 (d, *J* = 6.2 Hz, 3H), 0.82 (d, *J* = 6.2 Hz, 3H).

^{13}C NMR (126 MHz, CDCl$_3$): δ 176.5, 111.8, 64.5, 64.2, 53.5, 51.3, 50.3, 44.2, 43.6, 41.5, 39.0, 38.1, 31.2, 30.9, 26.6, 23.4, 19.2, 17.0.

HRMS (CI): *m/z* calculated $C_{18}H_{26}O_4$ $[M]^+$ 306.1831, found 306.1826.

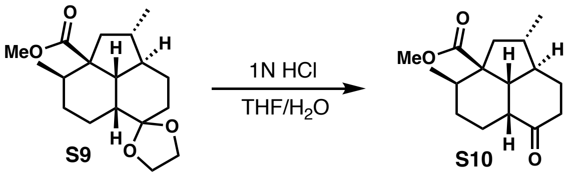

Ketone S10: 1N aq. HCl (1.6 mL) was added to a solution of ketal **S9** (50 mg, 0.16 mmol) in THF (3.2 mL). After 20 h, the reaction was diluted with EtOAc (10 mL), quenched with sat. aq. $NaHCO_3$ (20 mL), and the resulting mixture was extracted with EtOAc (3 x 5 mL). The combined organic extracts were washed with brine (10 mL), dried over $MgSO_4$, filtered, and concentrated *in vacuo*. The resulting slightly yellow oil, ketone **S10** (42 mg) was used in the next step without purification. The product was isolated as an inseparable mixture of diastereomers (~4:1) but the peaks for the major diastereomer at C3 are reported below.

^{1}H NMR (500 MHz, CDCl$_3$): δ 3.66 (d, *J* = 1.3 Hz, 3H), 2.96 (dt, *J* = 12.1, 5.3 Hz, 1H), 2.84 (dd, *J* = 14.1, 9.7 Hz, 1H), 2.53 – 2.27 (m, 3H), 2.13 – 2.08 (m, 2H), 1.99 (dd, *J* = 12.5, 5.5 Hz, 1H), 1.94 – 1.80 (m, 1H), 1.78 – 1.67 (m, 2H), 1.66 – 1.59 (m, 2H), 1.60 – 1.53 (m, 1H), 1.32 (dd, *J* = 14.2, 9.6 Hz, 1H), 1.07 (d, *J* = 6.5 Hz, 3H), 0.85 (d, *J* = 6.3 Hz, 3H).

^{13}C NMR (126 MHz, CDCl$_3$): δ 215.5, 176.0, 51.9, 51.5, 49.8, 44.7, 43.5, 39.0, 38.2, 37.7, 30.6(2C), 28.1, 26.0, 19.0, 17.1.

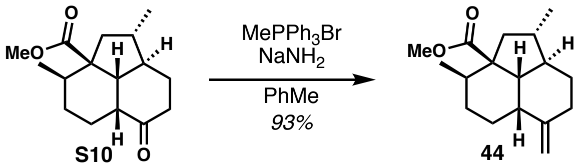

Alkene 44: A suspension of $MePPh_3Br$* (536 mg, 1.5 mmol) and $NaNH_2$ (146 mg, 1.88 mmol, 50 wt. % in PhMe) in PhMe (5 mL) in a 2-necked conical flask was heated to reflux. After 2 h, the resulting yellow suspension was cooled to rt, the stirring was stopped, and the solids were allowed to settle to the bottom of the flask for 2 h. The clear yellow, salt-free ylide solution was used in the following reaction. A solution of ketone **S10** (42 mg, 0.16 mmol) was dissolved in PhMe (0.5 mL) and cooled to 0 ºC. The methylene ylide (1.6 mL, 0.48 mmol, 0.3M in PhMe) was added dropwise. The yellow solution was warmed to rt. After 2 h, the reaction was quenched by addition of brine (5 mL) and extracted with Et_2O (3 x 5 mL). The combined organic extracts were washed with H_2O (5 mL), brine (5 mL) and dried over $MgSO_4$, filtered, and concentrated *in vacuo*. The crude oil was purified by column chromatography (SiO_2, 100% hexanes to 20:1 hexanes/EtOAc) to give alkene **44** (39 mg, 93% over 2 steps) as a yellow oil. The product was isolated as an inseparable mixture of diastereomers (~4:1) but the peaks for the major diastereomer at C3 are reported below.

*Note: $MePPh_3Br$ was dried by azeotroping with PhH and placing under vacuum (ca. 200 mTorr) overnight (12 h) prior to use.

^{1}H NMR (600 MHz, $CDCl_3$): δ 4.77 (t, J = 2.2 Hz, 1H), 4.68 (t, J = 2.3 Hz, 1H), 3.67 (s, 3H), 2.86 (dt, J = 12.2, 5.0 Hz, 1H), 2.73 (dd, J = 14.0, 9.4 Hz, 1H), 2.25 – 2.09 (m, 3H), 2.01 – 1.91 (m, 1H), 1.68 – 1.44 (m, 7H), 1.18 (dd, J = 14.0, 8.9 Hz, 1H), 1.00 (d, J = 6.3 Hz, 3H), 0.91 – 0.84 (m, 1H), 0.83 (d, J = 6.6 Hz, 3H).

^{13}C NMR (151 MHz, $CDCl_3$): δ 176.8, 153.0, 109.3, 53.9, 53.1, 51.3, 44.3, 44.2, 42.6, 38.9, 38.5, 31.5, 31.2, 31.1, 28.1, 19.0, 17.1.

HRMS (CI): *m/z* calculated for $C_{17}H_{26}O_2$ $[M]^+$ 262.1933, found 262.1931.

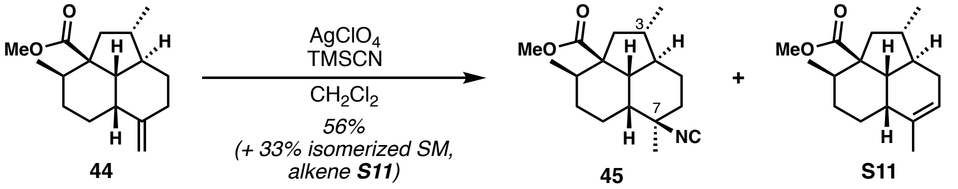

Isonitrile 45: TMSCN (56 μL, 0.45 mmol) and $AgClO_4$ (62 mg, 0.30 mmol) were sequentially added to a solution of alkene **44** (39 mg, 0.15 mmol) in CH_2Cl_2 (0.6 mL). After 18 h, the reaction was quenched with the addition of sat. aq. $NaHCO_3$ (0.6 mL) and the mixture was vigorously stirred for 10 min before extracting with Et_2O (3 x 5 mL). The combined organic extracts were washed with H_2O (5 mL), brine (5 mL) and dried over Na_2SO_4, filtered, and concentrated *in vacuo*. The crude oil was purified by column chromatography (SiO_2, 20:1 hexanes/EtOAc) to give a single diastereomer at C7 (>20:1 dr) of isonitrile **45** (24 mg, 56%) and isomerized trisubstituted alkene **S11** (13 mg, 33%) which could be resubjected to the reaction conditions if desired. The product was isolated as an inseparable mixture of diastereomers (~4:1) at C3 but the peaks for the major diastereomer at C3 are reported below.

^{1}H NMR (500 MHz, CDCl$_3$): δ 3.68 (s, 3H), 2.74 (dd, *J* = 14.0, 9.9 Hz, 1H), 2.38 (dt, *J* = 13.9, 4.4 Hz, 1H), 2.08 (dd, *J* = 12.0, 4.6 Hz, 1H), 2.02 – 1.90 (m, 1H), 1.86 – 1.70 (m, 3H), 1.57 – 1.47 (m, 4H), 1.40 (d, *J* = 1.9 Hz, 3H), 1.38 – 1.30 (m, 2H), 1.23 – 1.13 (m, 2H), 1.00 (d, *J* = 6.6 Hz, 3H), 0.82 (d, *J* = 6.3 Hz, 3H).

^{13}C NMR (151 MHz, CDCl$_3$): δ 176.1, 153.4, 62.0, 54.1, 51.4, 47.9, 43.4, 43.3, 43.2, 38.4, 38.1, 34.2, 31.1, 27.4, 25.9, 22.6, 19.1, 16.8.

Endocyclic alkene S11:

^{1}H NMR (500 MHz, CDCl$_3$): δ 5.39 – 5.32 (m, 1H), 3.66 (s, 3H), 2.77 (dd, *J* = 14.0, 9.3 Hz, 1H), 2.37 (dd, *J* = 12.0, 5.6 Hz, 1H), 2.22 (ddq, *J* = 12.2, 5.6, 2.8, 1.9 Hz, 1H), 1.94 (qd, *J* = 12.0, 10.6, 4.1 Hz, 2H), 1.80 – 1.72 (m, 1H), 1.70 (s, 3H), 1.66 – 1.62 (m, 1H), 1.58 – 1.53 (m, 1H), 1.53 – 1.48 (m, 2H), 1.45 – 1.42 (m, 1H), 1.21 (dd, *J* = 14.0, 9.0 Hz, 1H), 1.12 (qd, *J* = 13.0, 3.6 Hz, 1H), 1.01 (d, *J* = 6.3 Hz, 3H), 0.85 (d, *J* = 6.6 Hz, 3H).

^{13}C NMR (151 MHz, CDCl$_3$): δ 177.2, 138.9, 120.4, 54.7, 51.3, 49.8, 44.7, 40.8, 40.0, (2C), 39.1, 31.5, 31.0, 28.4, 21.9, 19.3, 17.4.

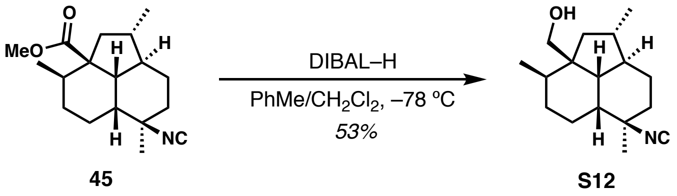

Alcohol S12: DIBAL–H (a freshly prepared 1.0M solution in PhMe, 0.15 mL, 0.15 mmol) was added to a solution of isonitrile **45** (12.5 mg, 43 μmol) in CH_2Cl_2 (0.43 mL) at –78 °C. After 3 h, the reaction was quenched with H_2O (0.1 mL) and 1N NaOH (0.1 mL), and then warmed to rt for 15 min. $MgSO_4$ was added and the mixture stirred for an additional 15 min at rt before filtering over cotton. The filtrate was concentrated *in vacuo* and the crude oil was purified by column chromatography (SiO_2, 10:1 hexanes/EtOAc) to give alcohol **S12** (6 mg, 53%). The product was isolated as an inseparable mixture of diastereomers (~4:1) but the peaks for the major diastereomer at C3 are reported below.

^{1}H NMR (500 MHz, $CDCl_3$): δ 3.69 (d, J = 10.3 Hz, 1H), 3.54 (d, J = 10.8 Hz, 1H), 2.09 – 1.97 (m, 2H), 1.88 (dd, J = 13.7, 9.9 Hz, 1H), 1.84 – 1.73 (m, 2H), 1.58 – 1.49 (m, 4H), 1.48 – 1.42 (m, 1H), 1.39 (s, 3H and m, 1H), 1.38 – 1.31 (m, 1H), 1.28 – 1.18 (m, 3H), 1.05 (dd, J = 13.4, 8.5 Hz, 1H), 0.99 (d, J = 6.6 Hz, 3H), 0.88 (d, J = 6.9 Hz, 3H).

^{13}C NMR (126 MHz, $CDCl_3$): δ 65.4, 62.0, 46.40, 46.36, 44.0, 43.5, 42.7, 38.5, 37.9, 34.5, 31.8, 27.5, 26.3, 23.0, 19.6, 16.0, (–NC not observed).

HRMS (ESI): *m/z* calculated for $C_{17}H_{27}NO$ $[M + Na]^+$ 284.1990, found 284.1979.

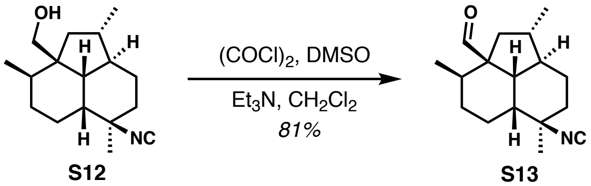

Aldehyde S13: Oxalyl chloride (56 μL, 28 μmol, 0.5M in CH_2Cl_2) was cooled to –78 °C and DMSO (0.11 mL, 55 μmol, 0.5 M in CH_2Cl_2) was added dropwise (gas evolution was observed). After 20 min, a solution of alcohol **S12** (6 mg, 23 mmol) in CH_2Cl_2 (0.23 mL) was added dropwise over 5 min. After 30 min, Et_3N (16 μL, 115 μmol) was added dropwise. The solution was maintained at –78 °C for 1 h before warming to rt for an

additional 1 h. The reaction was diluted with CH_2Cl_2 (5 mL), washed with H_2O (5 mL), and extracted with CH_2Cl_2 (3 x 5 mL). The organic extracts were dried over Na_2SO_4, filtered, and concentrated *in vacuo*. The crude material was purified by column chromatography (SiO_2, 5:1 hexanes/EtOAc) to give aldehyde **S13** (4.9 mg, 81%) and recovered starting material (1.1 mg). The product was isolated as an inseparable mixture of diastereomers (~4:1) but the peaks for the major diastereomer at C3 are reported below.

^{1}H NMR (500 MHz, $CDCl_3$): δ 9.68 (s, 1H, *minor diastereomer*), 9.67 (s, 1H, *major diastereomer*), 2.50 – 2.39 (m, 2H), 2.26 – 2.18 (m, 1H), 2.13 (d, J = 14.3 Hz, 1H), 1.87 – 1.82 (m, 1H), 1.80 – 1.73 (m, 3H), 1.68 – 1.59 (m, 5H), 1.40 (s, 3H), 1.37 – 1.32 (m, 2H), 1.04 (d, J = 6.5 Hz, 3H, *major diastereomer*), 1.03 (d, J = 6.4 Hz, 3H, *minor diastereomer*), 0.99 (d, J = 6.5 Hz, 3H, *major diastereomer*), 0.97 (d, J = 6.4 Hz, 3H, *minor diastereomer*).

^{13}C NMR (126 MHz, $CDCl_3$): δ 205.2, 47.2, 46.1, 43.6, 43.3, 40.5, 38.7, 37.5, 34.4, 31.6, 29.9, 27.4, 25.9, 22.8, 19.3, 16.1.

HRMS (ESI): *m/z* calculated for $C_{17}H_{25}NO$ $[M + Na]^+$ 282.1834, found 282.1823.

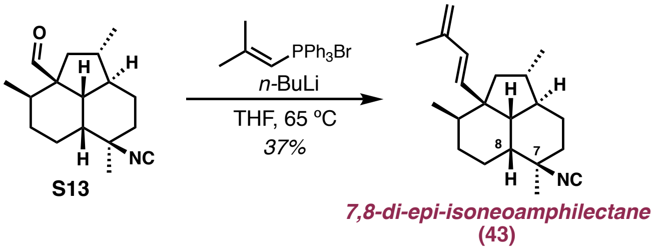

7,8-di-*epi*-isoneoamphilectane (43): *n*-BuLi (36 μL, 91 μmol, 2.5M in hexanes) was added to a suspension of phosphonium bromide (43 mg, 0.11 mmol) in THF (0.15 mL) at 0 °C. The bright orange solution was warmed to rt for 1 h. Aldehyde **S13** (4.7 mg, 18 μmol) in THF (0.25 mL) was added dropwise and then heated to reflux for 5 h. The reaction was cooled to rt, quenched with sat. aq. NH_4Cl (1 mL) and extracted with EtOAc (3 x 3 mL). The combined organic extracts were washed with brine (5 mL), dried over $MgSO_4$, filtered, and concentrated *in vacuo*. The crude material was purified by column chromatography (SiO_2, 100% hexanes to 20:1 hexanes/EtOAc) to give isonitrile **43** (2.0 mg, 37%) and unreacted aldehyde (2.0 mg). The data for 7,8-di-*epi*-isoneoamphilectane

(**43**) matched those reported for the Shenvi isocyanation product above.

Synthesis of 8-*epi*-isoneoamphilectane:

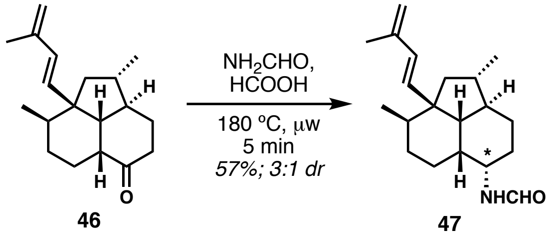

Formamide 47: A microwave vial was charged with ketone **46** (described earlier in the SI as **S8**, because the structure was not explicitly shown in the main text earlier in the narrative, 10 mg, 37 μmol), formaldehyde (73 μL, 1.84 mmol), and formic acid (2 drops). The reaction vial was placed in a microwave reactor and heated to 180 °C for 4 min. A second batch of ketone **46** (10 mg) was subjected to the same conditions. After completion of both reactions, the reactions were combined in a separatory funnel, diluted with $CHCl_3$ (10 mL) and washed with H_2O (5 mL). The aqueous phase was extracted with $CHCl_3$ (2 x 5 mL) and the combined organic extracts were washed with brine (5 mL), dried over $MgSO_4$, filtered, and concentrated *in vacuo*. The crude formamide purified by column chromatography (SiO_2, 5:1 to 1:1 hexanes/EtOAc) to give two diastereomeric formamides **47** (12.7 mg, 57%, 3:1 dr) as a colorless semisolid. The individual formamides were separated for spectroscopic purposes but combined for the following reactions.

*Major diastereomer (**47** as drawn): more polar*

^{1}H NMR (500 MHz, $CDCl_3$): 8.16 (d, *J* = 1.9 Hz, 1H), 6.14 (d, *J* = 16.1 Hz, 1H), 5.85 (d, *J* = 16.1 Hz, 1H), 5.43 (bd, *J* = 8.9 Hz, 1H, *–NH*), 4.92 (s, 1H), 4.24 – 4.03 (m, 1H), 2.24 (tt, *J* = 8.9, 4.4 Hz, 1H), 2.18 – 2.06 (m, 1H), 1.92 (dd, *J* = 12.7, 3.2 Hz, 1H), 1.84 (s, 3H), 1.77 – 1.67 (m, 2H), 1.67 – 1.59 (m, 1H), 1.58 – 1.45 (m, 4H), 1.47 – 1.38 (m, 1H), 1.34 (dd, *J* = 12.8, 3.8 Hz, 1H), 1.29 – 1.22 (m, 2H), 1.19 – 1.12 (m, 1H), 1.00 (d, *J* = 5.5 Hz, 3H), 0.77 (d, *J* = 6.5 Hz, 3H). (only major rotamer listed)

^{13}C NMR (126 MHz, $CDCl_3$): δ 160.4, 142.5, 133.9, 130.9, 114.8, 54.3, 50.1, 47.7, 45.0, 43.3, 39.1, 38.2, 36.2, 31.2, 29.0, 28.2, 21.2, 19.7, 19.0, 16.7. (only major rotamer listed)

Minor diastereomer (not drawn): less polar

^{1}H NMR (500 MHz, CDCl$_3$): δ 8.15 (s, 1H), 6.13 (d, *J* = 16.1 Hz, 1H), 5.87 (d, *J* = 16.1 Hz, 1H), 4.91 (s, 2H), 4.06 (d, *J* = 8.5 Hz, 1H), 2.17 – 2.07 (m, 1H), 2.04 – 1.95 (m, 3H), 1.84 (s, 6H), 1.77 – 1.37 (m, 8H), 1.26 (s, 3H), 1.24 – 1.17 (m, 1H), 1.02 (d, *J* = 6.0 Hz, 3H), 0.94 – 0.84 (m, 1H), 0.76 (d, *J* = 6.6 Hz, 3H). (only major rotamer listed)
^{13}C NMR (126 MHz, CDCl$_3$): δ 160.2, 142.5, 134.3, 130.7, 114.8, 51.0, 48.3, 47.9, 44.2, 43.8, 39.0, 38.8, 38.3, 31.4, 26.5, 26.4, 26.0, 19.5, 19.0, 16.7. (only major rotamer listed)
HRMS (ESI) [of 3:1 mixture of diastereomers]**:** *m/z* calculated for $C_{20}H_{29}N$ [M + Na]$^+$ 324.2303, found 324.2306.

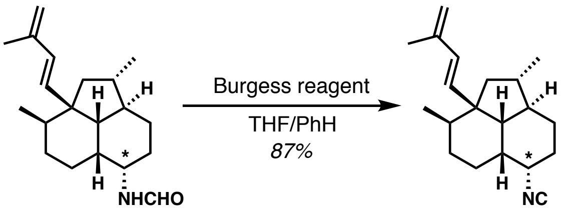

47 48

Isonitrile 48: Burgess reagent (5.2 mg, 22 μmol) was added to a solution of formamide **47** (4.4 mg, 15 μmol; 3:1 dr) in a 1:1 mixture of PhH/THF (0.15 mL). After 16 h, the reaction was filtered through a short plug of SiO_2, rinsing with EtOAc (10 mL). The filtrate was concentrated *in vacuo* and the crude product was further purified by column chromatography (SiO_2, 100% hexanes to 20:1 hexanes/EtOAc) to afford isonitrile **48** (3.7 mg, 87%, 3:1 dr, inseparable).
^{1}H NMR (500 MHz, CDCl$_3$): δ 6.18 (d, *J* = 16.2 Hz, 1H, *minor diastereomer*), 6.13 (d, *J* = 16.1 Hz, 1H, *major diastereomer*), 5.85 (d, *J* = 16.2 Hz, 1H, *major diastereomer*), 5.83 (d, *J* = 15.9 Hz, 1H, *minor diastereomer*), 4.92 (s, 2H), 3.71 – 3.56 (m, 1H), 2.33 – 2.24 (m, 1H), 2.18 – 2.06 (m, 2H), 2.00 (dt, *J* = 12.6, 3.6 Hz, 1H), 1.93 (dd, *J* = 13.0, 3.4 Hz, 1H), 1.85 (s, 3H), 1.82 – 1.75 (m, 1H), 1.72 – 1.65 (m, 3H), 1.53 – 1.48 (m, 3H), 1.47 – 1.43 (m, 1H), 1.42 – 1.37 (m, 1H), 1.21 – 1.17 (m, 1H), 1.00 (d, *J* = 5.7 Hz, 3H), 0.79 (d, *J* = 6.7 Hz, 3H).
^{13}C NMR (151 MHz, CDCl$_3$): For the major diastereomer δ 142.5, 133.8, 130.9, 115.0, 56.4, 53.8, 47.7, 45.0, 43.0, 39.1, 38.3, 37.2, 31.1, 29.9, 29.1, 21.4, 19.6, 19.0, 16.7, (–NC not observed).

HRMS (CI): *m/z* calculated for $C_{20}H_{29}N$ $[M]^+$ 283.2300, found 283.2312.

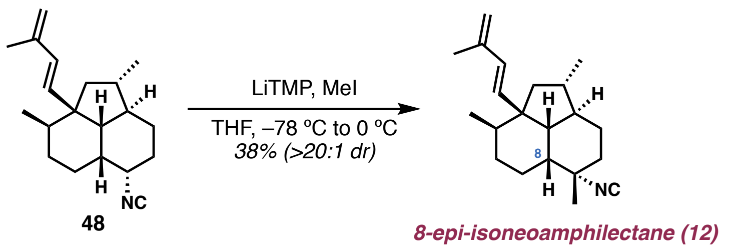

8-*epi*-isoneoamphilectane (12): *n*-BuLi (63 mL, 97 μmol; 1.55M in hexanes) was added to a solution of TMP (17 μl, 99 mmol) in THF (0.1 mL) at 0 °C. After 10 min, the LiTMP solution was cooled to –78 °C and a solution of isonitrile **48** (5.2 mg, 19 μmol) in THF (0.3 mL) was added dropwise. After 1 h, iodomethane (24 μL, 0.39 mmol) was added dropwise and the solution was allowed to warm to 0 °C over 2 h. The reaction was quenched at 0 °C with the addition of sat. aq. NH_4Cl (0.25 mL) and extracted with EtOAc (3 x 1 mL). The combined organic extracts were washed with brine (2 mL), dried over $MgSO_4$, filtered, and concentrated *in vacuo*. Poor conversion (~20%) was observed by NMR analysis of the crude reaction mixture, so the crude material was resubjected to a slightly modified procedure: LiTMP was generated at 0 °C from *n*-BuLi (0.13 mL) and TMP (34 μL) in THF (0.1 mL) and cooled to –78 °C before the substrate was added as a solution in THF (0.3 mL). The reaction vial was allowed to slowly warm to –60 °C over 1 h before cooling to –78 °C for the addition of MeI (50 μL). The reaction was then allowed to warm to rt in the –78 °C overnight (14 h). The same workup as described above was performed. The crude isonitrile was purified by column chromatography (SiO_2, 2:1 hexane/toluene) to give **12** (2.0 mg, 38%).

^{1}H NMR (500 MHz, $CDCl_3$): δ 6.11 (d, *J* = 16.1 Hz, 1H), 5.83 (d, *J* = 16.1 Hz, 1H), 4.91 (d, *J* = 12.3 Hz, 2H), 2.06 (dd, *J* = 13.5, 9.4 Hz, 1H), 1.99 (d, *J* = 13.3 Hz, 1H), 1.85 (s, 3H), 1.85 – 1.75 (m, 1H), 1.76 – 1.69 (m, 2H), 1.69 – 1.62 (m, 1H), 1.54 – 1.47 (m, 3H), 1.44 (s, 3H), 1.41 – 1.33 (m, 2H), 1.19 – 1.11 (m, 2H), 1.00 (d, *J* = 5.9 Hz, 3H), 0.99 – 0.91 (m, 2H), 0.79 (d, *J* = 6.7 Hz, 3H).

^{13}C NMR (126 MHz, $CDCl_3$): δ 142.5, 133.91, 130.7, 114.6, 50.0, 47.7, 45.0, 43.2, 40.1, 38.6, 38.1, 367.0, 33.4, 31.4, 26.0, 24.2, 22.9, 19.7, 16.6, 14.3.

HRMS (CI): *m/z* calculated for $C_{21}H_{31}N$ $[M]^+$ 297.2456, found 297.2450.

$[\alpha]^{22}_D$: +104.0 (c = 0.3 $CDCl_3$)

Synthesis of *trans*-decalone containing tricycle:

First generation route to **58**:

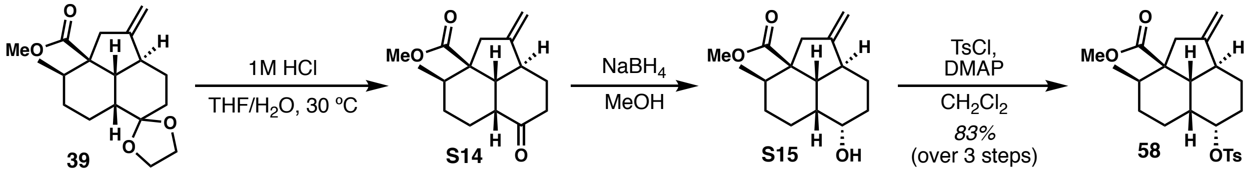

Ketone S14: Ketal **39** (600 mg, 1.95 mmol) was dissolved in THF (39 mL) and 1N aq. HCl (20 mL, 19.6 mmol) was added. The solution was heated to 30 °C for 17 h. The reaction mixture was diluted with EtOAc (100 mL) and washed with sat. aq. $NaHCO_3$ (25 mL). The aqueous phase was extracted with EtOAc (3 x 10 mL) and the combined organic extracts were washed with brine (25 mL), dried over $MgSO_4$, filtered, and concentrated *in vacuo*. The crude ketone **S14** (510 mg) was used in the following step without purification.

^{1}H NMR (499 MHz, $CDCl_3$): δ 4.89 (q, *J* = 2.4 Hz, 1H), 4.79 (q, *J* = 2.6 Hz, 1H), 3.67 (s, 3H), 3.16 – 2.99 (m, 2H), 2.90 (t, *J* = 12.6 Hz, 1H), 2.63 (dq, *J* = 18.0, 2.1 Hz, 1H), 2.48 (ddd, *J* = 16.2, 13.0, 6.6 Hz, 1H), 2.40 (dddd, *J* = 16.2, 5.2, 2.3, 1.3 Hz, 1H), 2.25 (dddd, *J* = 12.7, 6.3, 3.4, 2.2 Hz, 1H), 2.02 (dd, *J* = 13.3, 5.2 Hz, 1H), 1.79 – 1.65 (m, 3H), 1.63 – 1.50 (m, 2H), 1.45 (tdd, *J* = 13.0, 11.3, 5.1 Hz, 1H), 0.89 (d, *J* = 6.7 Hz, 3H).

^{13}C NMR (125 MHz, $CDCl_3$): δ 214.6, 175.3, 149.9, 105.3, 52.1, 51.6, 50.5, 49.5, 41.5, 40.1, 37.4, 35.4, 30.3, 26.6, 25.1, 17.3.

HRMS (ESI): *m/z* calculated for $C_{16}H_{22}O_3$ $[M + Na]^+$ 285.1467, found 285.1472.

Alcohol S15: Sodium borohydride (148 mg, 3.9 mmol) was added portionwise to a solution of ketone **S14** (510 mg) in MeOH (20 mL) at 0 °C. No precautions were taken to exclude air or moisture. After 4 h, the methanol was removed *in vacuo* and the resulting residue was diluted with EtOAc (20 mL) and washed with H_2O (10 mL). The aqueous layer was extracted with EtOAc (3 x 5 mL), and the combined organic extracts were

washed with brine (5 mL), dried over $MgSO_4$, filtered, and concentrated *in vacuo*. The resulting colorless oil, alcohol **S15** (500 mg) was used in the next step without purification.

^{1}H NMR (500 MHz, $CDCl_3$) δ 4.78 (td, J = 3.1, 1.5 Hz, 1H), 4.70 (qd, J = 2.6, 1.0 Hz, 1H), 3.81 (dt, J = 11.8, 5.0 Hz, 1H), 3.67 (s, 3H), 3.00 (dq, J = 18.0, 2.2 Hz, 1H), 2.60 (dq, J = 13.3, 4.7 Hz, 1H), 2.56 – 2.47 (m, 1H), 2.01 (dq, J = 12.8, 3.5 Hz, 1H), 1.84 – 1.77 (m, 1H), 1.74 – 1.66 (m, 2H), 1.66 – 1.58 (m, 3H), 1.59 – 1.51 (m, 2H), 1.52 – 1.42 (m, 1H), 1.11 (tdd, J = 13.1, 11.3, 3.6 Hz, 1H), 0.86 (d, J = 6.7 Hz, 3H).

^{13}C NMR (126 MHz, $CDCl_3$) δ 175.9, 151.4, 103.7, 73.3, 52.7, 51.4, 50.7, 41.3, 40.6, 38.8, 36.1, 30.8, 29.7, 26.1, 19.3, 17.3.

Tosylate 58: DMAP (715 mg, 5.85 mmol) and TsCl (558 mg, 2.92 mmol) were sequentially added to a solution of alcohol **S15** (500 mg) in CH_2Cl_2 (7.8 mL) at 0 °C and the reaction was allowed to warm to rt overnight. After 18 h, the reaction mixture was diluted with CH_2Cl_2 (10 mL), washed with 1N HCl (10 mL), and the aqueous phase was extracted with CH_2Cl_2 (5 x 5 mL). The combined organic extracts were washed with sat. aq. $NaHCO_3$ (10 mL) and brine (10 mL), dried over $NaSO_4$, filtered, and concentrated *in vacuo*. The crude tosylate was purified by column chromatography (SiO_2, 10:1 hexanes/EtOAc) to give **58** (675 mg, 83% over 3 steps) as a viscous colorless oil (foam under vacuum).

^{1}H NMR (500 MHz, $CDCl_3$): δ 7.81 (d, J = 8.3 Hz, 2H), 7.35 (d, J = 8.0 Hz, 2H), 4.77 (d, J = 2.4 Hz, 1H), 4.65 (q, J = 2.4 Hz, 1H), 4.62 (dd, J = 11.6, 5.8 Hz, 1H), 3.64 (s, 3H), 2.96 (dq, J = 18.0, 2.1 Hz, 1H), 2.68 – 2.56 (m, 1H), 2.52 – 2.41 (m, 2H), 2.46 (s, 3H), 1.98 (dq, J = 13.1, 3.7 Hz, 1H), 1.79 (dt, J = 12.3, 4.0 Hz, 1H), 1.73 – 1.64 (m, 3H), 1.63 – 1.51 (m, 2H), 1.51 – 1.39 (m, 2H), 1.06 (qd, J = 13.2, 3.8 Hz, 1H), 0.82 (d, J = 6.8 Hz, 3H).

^{13}C NMR (126 MHz, $CDCl_3$): δ 175.4, 150.4, 144.5, 134.8, 129.9, 127.76, 127.73, 127.68, 104.2, 84.2, 52.6, 51.4, 50.0, 41.2, 40.1, 37.2, 35.7, 30.3, 27.1, 25.9, 21.8, 19.7, 17.1.

HRMS (ESI): *m/z* calculated for $C_{23}H_{30}O_5S$ $[M + Na]^+$ 441.1712, found 441.1720.

$[\alpha]^{22}_D$: –26.2 (c = 2, $CHCl_3$)

Second generation approach to **58**:

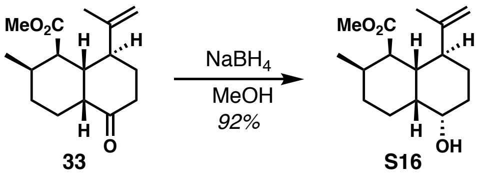

Alcohol S16: Sodium borohydride (150 mg, 3.97 mmol) was added portionwise to a solution of decalone **33** (525 mg, 1.99 mmol**)** in MeOH (20 mL) at 0 °C (no precautions were taken to exclude air or moisture). After 1 h, the methanol was removed *in vacuo* and the resulting residue was diluted with EtOAc (20 mL) and washed with H_2O (10 mL). The aqueous phase was extracted with EtOAc (3 x 5 mL), and the combined organic extracts were washed with brine (5 mL), dried over $MgSO_4$, filtered, and concentrated *in vacuo*. The resulting colorless oil, alcohol **S16** (490 mg; 92%) was analytically pure and used in the next step without purification.

^{1}H NMR (499 MHz, $CDCl_3$): δ 4.79 (dd, J = 2.4, 1.4 Hz, 1H), 4.76 (d, J = 2.3 Hz, 1H), 3.78 – 3.71 (m, 1H), 3.64 (s, 3H), 2.58 – 2.50 (m, 1H), 2.47 – 2.30 (m, 2H), 1.83 (dt, J = 12.0, 2.3 Hz, 1H), 1.76 – 1.69 (m, 5H), 1.66 (dd, J = 9.5, 2.8 Hz, H), 1.65 (s, 3H), 1.54 (dd, J = 12.2, 3.5 Hz, 1H), 1.49 – 1.35 (m, 3H), 0.93 (d, J = 6.3 Hz, 3H).

^{13}C NMR (125 MHz, $CDCl_3$): δ 175.2, 147.4, 112.7, 73.0, 51.1, 46.7 (2-C), 42.4, 40.4, 38.1, 30.3, 29.20, 29.15, 28.6, 19.9, 19.5.

HRMS (ESI): *m/z* calculated for $C_{16}H_{22}O_3$ $[M + Na]^+$ 289.1780, found 289.1782.

$[\alpha]^{22}_D$: –54.8 (c = 1, $CHCl_3$)

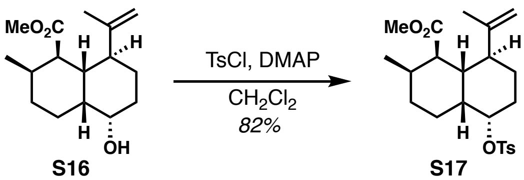

Tosylate S17: DMAP (652 mg, 5.34 mmol) and TsCl (510 mg, 2.67 mmol) were sequentially added to a solution of alcohol **S16** (475 mg, 1.78 mmol) in CH_2Cl_2 (7.1 mL) at 0 °C and the reaction was allowed to warm to rt overnight. After 20 h, the reaction was diluted with CH_2Cl_2 (10 mL), washed with 1N HCl (10 mL), and the aqueous layer was extracted with CH_2Cl_2 (5 x 5 mL). The combined organic extracts were washed with sat.

aq. $NaHCO_3$ (10 mL) and brine (10 mL), dried over $NaSO_4$, filtered, and concentrated *in vacuo*. The crude tosylate was purified by column chromatography (SiO_2, 10:1 hexanes/EtOAc) to give **S17** (615 mg, 82%) as a viscous colorless oil (foam under vacuum).

^{1}H NMR (500 MHz, $CDCl_3$): δ 7.80 (d, J = 8.3 Hz, 2H), 7.34 (d, J = 8.0 Hz, 2H), 4.78 (t, J = 1.8 Hz, 1H), 4.73 (d, J = 2.2 Hz, 1H), 4.61 – 4.52 (m, 1H), 3.60 (s, 3H), 2.48–2.44 (overlapping m, 1H and s, 3H), 2.35 (td, J = 12.3, 3.9 Hz, 1H), 2.29 (dt, J = 12.5, 4.2 Hz, 1H), 1.82 – 1.71 (m, 3H), 1.69 – 1.62 (m, 2H), 1.60 (s, 3H), 1.54 (td, J = 12.9, 4.1 Hz, 1H), 1.49 – 1.42 (m, 2H), 1.41 – 1.25 (m, 2H), 0.88 (d, J = 6.8 Hz, 3H).

^{13}C NMR (126 MHz, $CDCl_3$): δ 174.4, 146.5, 144.6, 134.9, 129.9 (2-C), 127.7 (2-C), 113.2, 84.1, 51.2, 46.4, 41.9, 40.2, 36.1, 29.9, 28.7, 28.2, 26.9, 21.8, 19.9, 19.8.

HRMS (ESI): *m/z* calculated for $C_{23}H_{32}O_5S$ $[M + Na]^+$ 443.1863, found 443.1869.

$[\alpha]^{22}_D$: –53.4 (c = 1, $CHCl_3$)

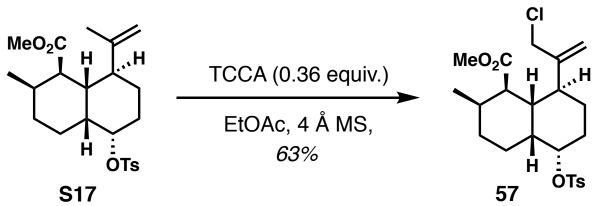

S17 → 57

Allylic chloride 57: Alkene **S17** (229 mg, 0.86 mmol) was dissolved in EtOAc (17.2 mL, previously degassed over 4 Å mol sieves.), 4 Å mol sieves were added, and the mixture stirred at rt for 15 min before cooling to 0 °C. Trichloroisocyanuric acid (TCCA; 72 mg, 0.31 mmol) was added in one portion. After disappearance of starting material by TLC (approx. 30 min), the reaction was quenched at 0 °C by addition of sat. aq. $Na_2S_2O_3$ (10 mL) and extracted with CH_2Cl_2 (3 x 15 mL). The combined organic extracts were washed with brine (25 mL), dried over Na_2SO_4, filtered and concentrated *in vacuo*. The crude yellow oil contained a 2.2:1 mixture of **57** and the corresponding alkenyl chloride. The allylic chloride could be purified through multiple rounds of column chromatography (SiO_2, 10:1 to 5:1 hexanes/EtOAc) to give **57** (227 mg, 63%), as a white solid, but was typically used as an impure mixture in the following step. Caution: the product is not very soluble in EtOAc so CH_2Cl_2 was used to aid in loading material onto column.

X-ray quality crystals were grown by slow evaporation a mixture of hexanes/CH_2Cl_2 (ca. 10:1) over 36 h. The setup for crystallization was as follows: the purified product was transferred to a 1-dram vial and dissolved in a minimum amount of the solvent mixture. This vial was then placed in a 20 mL scintillation vial containing hexanes (ca. 5–8 mL) and capped. Slow diffusion of CH_2Cl_2 out of the inner vial produced crystals of suitable quality for X-ray diffraction studies.

^{1}H NMR (500 MHz, $CDCl_3$): δ 7.80 (d, *J* = 8.4 Hz, 2H), 7.35 (d, *J* = 8.0 Hz, 2H), 5.30 (s, 1H), 5.05 (s, 1H), 4.62 (dt, *J* = 11.3, 5.4 Hz, 1H), 3.99 (dd, *J* = 2.4, 0.9 Hz, 2H), 3.60 (s, 3H), 2.59 (d, *J* = 4.6 Hz, 1H), 2.46 (s, 3H), 2.43 (dd, *J* = 12.3, 3.7 Hz, 1H), 2.38 (dq, *J* = 12.9, 4.3 Hz, 1H), 1.94 (dd, *J* = 12.2, 3.9 Hz, 1H), 1.88 (dq, *J* = 13.7, 3.7 Hz, 1H), 1.81 – 1.71 (m, 3H), 1.68 – 1.62 (m, 1H), 1.57 – 1.41 (m, 3H), 1.32 – 1.20 (m, 1H), 0.88 (d, *J* = 6.9 Hz, 3H).

^{13}C NMR (126 MHz, $CDCl_3$): δ 174.2, 147.0, 144.7, 134.8, 129.9 (2-C), 127.7 (2-C), 116.1, 83.7, 51.2, 46.2(2-C), 41.6 (2-C), 36.4, 32.4, 28.8, 28.7, 27.1, 21.8, 19.9, 19.7.

HRMS (ESI): *m/z* calculated for $C_{23}H_{31}ClO_5S$ $[M + Na]^+$ 477.1479, found 477.1475.

$[\alpha]^{22}_D$: –37.3 (c = 1, $CHCl_3$)

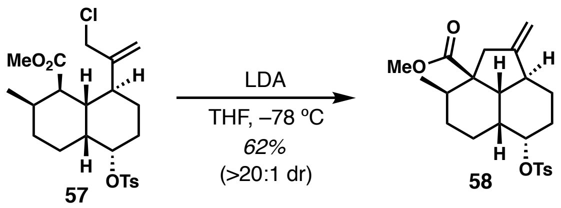

Tricycle 58: LDA was generated by addition of *n*-BuLi (0.42 mL, 1.05 mmol) to a solution of DIPA (0.15 mL, 1.1 mmol) in THF (3 mL) at 0 °C. In a separate flask, allylic chloride **57** (227 mg, 0.5 mmol) was dissolved in THF (7 mL) and cooled to –78 °C. LDA was added dropwise. After 1 h, the reaction was quenched with sat. aq. NH_4Cl (10 mL) and extracted with EtOAc (3 x 10 mL). The combined organic extracts were washed with brine (10 mL), dried over $MgSO_4$, filtered and concentrated *in vacuo*. The crude oil was purified by column chromatography (SiO_2, 5:1 hexanes/EtOAc) to give **58** (130 mg, 62%) as a colorless oil. See above for characterization data.

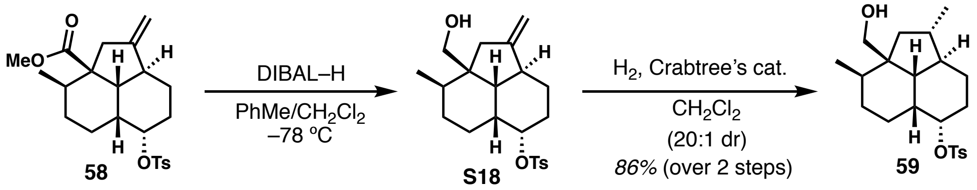

Alcohol S18: A freshly prepared solution of 1M DIBAL–H (0.72 mL, 4.03 mmol) in PhMe (4 mL) was added dropwise to a solution of ester **58** (675 mg, 1.61 mmol) in CH_2Cl_2 (8.1 mL) at –78 °C. After 3 h, the reaction was diluted with Et_2O (10 mL) and H_2O (0.15 mL) was added. The flask was removed from the –78 °C bath and 1N NaOH (0.15 mL) and an additional portion of H_2O (0.4 mL) was added. The mixture was allowed to stir at rt for 15 min before $MgSO_4$ was added. After stirring vigorously for 15 min, the mixture was filtered, and the salts were washed with Et_2O (15 mL). The filtrate was concentrated *in vacuo* to give crude alcohol **S18** (500 mg), which was used in the following step without purification. A small portion of the crude product was purified by column chromatography (SiO_2, 2:1 hexanes/EtOAc) for analytical purposes.

^{1}H NMR (500 MHz, $CDCl_3$): δ 7.80 (d, *J* = 8.3 Hz, 2H), 7.34 (d, *J* = 8.0 Hz, 2H), 4.75 (d, *J* = 2.7 Hz, 1H), 4.67 – 4.62 (m, 1H), 4.65 – 4.58 (m, 1zH), 3.61 (d, *J* = 10.9 Hz, 1H), 3.52 (d, *J* = 10.9 Hz, 1H), 2.5 – 2.4 (m, 2 H), 2.46 (s, 3H), 2.38 – 2.23 (m, 3H), 1.98 (dq, *J* = 13.0, 3.6 Hz, 1H), 1.76 – 1.62 (m, 4H), 1.64 – 1.53 (m, 3H), 1.49 (td, *J* = 13.0, 3.5 Hz, 1H), 1.27 – 1.20 (m, 1H), 1.08 (ddt, *J* = 17.2, 11.6, 5.3 Hz, 1H), 0.85 (d, *J* = 6.9 Hz, 3H).

^{13}C NMR (126 MHz, $CDCl_3$): δ 151.9, 144.6, 134.7, 129.9 (2-C), 127.8 (2-C), 104.0, 84.5, 63.0, 47.8, 44.85, 40.0, 39.6, 37.5, 35.1, 30.6, 27.3, 26.4, 21.8, 20.0, 16.2.

HRMS (ESI): *m/z* calculated for $C_{22}H_{30}O_4S$ $[M + Na]^+$ 413.1762, found 413.1759.

$[\alpha]^{22}_D$: –64.9 (c = 1, $CHCl_3$)

Hydrogenated tricycle 59: Alkene **S18** (500 mg, 1.28 mmol) was dissolved in CH_2Cl_2 (26 mL) and Crabtree's catalyst (51 mg, 64 μmol) was added. The solution was sparged with hydrogen for 5 min and the reaction remained under an atmosphere of hydrogen for 20 h. The reaction was concentrated *in vacuo* and the crude material was purified by column chromatography (SiO_2, 5:1 hexanes/EtOAc) to give **59** (430 mg; 86% over 2 steps; 20:1 dr) as a viscous colorless oil (foam under vacuum).

^{1}H NMR (500 MHz, CDCl$_3$): δ 7.79 (d, *J* = 8.4 Hz, 2H), 7.33 (d, *J* = 8.0 Hz, 2H), 4.62 (dt, *J* = 11.3, 5.4 Hz, 1H), 3.55 (d, *J* = 10.7 Hz, 1H), 3.47 (d, *J* = 10.7 Hz, 1H), 2.45 (s, 3H), 2.18 (dq, *J* = 13.7, 4.8 Hz, 1H), 1.90 (dd, *J* = 13.5, 9.6 Hz, 1H), 1.84 (dq, *J* = 12.8, 3.7 Hz, 1H), 1.70 – 1.34 (m, 10H), 1.17 (qd, *J* = 13.3, 3.2 Hz, 1H), 1.05 (dd, *J* = 13.6, 8.3 Hz, 1H), 0.94 (d, *J* = 6.4 Hz, 3H), 0.92 – 0.85 (m, 1H), 0.81 (d, *J* = 6.9 Hz, 3H).
^{13}C NMR (126 MHz, CDCl$_3$): δ 144.5, 134.8, 129.8 (2-C), 127.7 (2-C), 85.2, 63.7, 48.4, 45.8, 43.03, 42.95, 38.5, 38.4, 37.9, 31.1, 27.9, 27.5, 21.8, 20.7, 19.5, 16.0.
HRMS (ESI): *m/z* calculated for $C_{22}H_{32}O_4S$ [M + Na]$^+$ 415.1919, found 415.1931.
[α]$^{22}_{D}$: –61.6 (c = 1, $CHCl_3$)

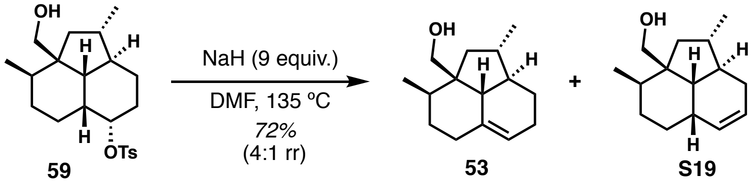

Alkene 53: *Caution: NaH/DMF can cause an exotherm upon heating; take all necessary safety precautions*

NaH (78 mg, 3.1 mmol; dry 95%) in DMF (10 mL) was added to a solution of tosylate **59** (135 mg, 0.34 mmol) in DMF (24.4 mL) in a 50 mL ACE pressure tube at 0 °C. The septum was replaced for the ACE tube screw cap and the suspension was allowed to stir for 10 min at 0 °C and 10 min at rt. The reaction was placed into a preheated oil bath (100 °C) behind a blast shield as the bath continued to warm to 135 °C. A series of color changes were observed from colorless to green, blue, purple, and finally brown for the remainder of the reaction. After 2 h, the reaction vessel was allowed to cool to rt and diluted with EtOAc (150 mL). The solution was washed with H_2O (300 mL) and extracted with EtOAc (3 x 75 mL). The combined organic extracts were washed with brine (5 x 100 mL), dried over $MgSO_4$, filtered, and concentrated *in vacuo*. The crude oil was purified by column chromatography (pH 7 SiO_2, 10:1 hexanes/EtOAc) to give **53** and **S19** (54 mg; 72%, 4:1 rr) as an inseparable mixture of alkene positional isomers.
^{1}H NMR (500 MHz, CDCl$_3$): δ 5.78 – 5.58 (m, 1H, *minor*), 5.19 (t, *J* = 2.9 Hz, 1H, *major*), 3.71 (d, *J* = 10.7 Hz, 1H, *minor*), 3.67 (d, *J* = 10.9 Hz, 1H, *major*), 3.53 (overlapping d, *J*

= 11.0 Hz and J = 11.5 Hz, 1H), 2.33 – 2.09 (m, 3H), 1.98 (dd, J = 12.9, 6.9 Hz, 1H), 1.95 – 1.82 (m, 1H), 1.80 – 1.67 (m, 2H), 1.62 – 1.56 (m, 1H), 1.57 – 1.44 (m, 2H), 1.41 – 1.36 (m, 1H), 1.35 – 1.29 (m, 1H), 1.24 – 1.18 (m, 1H), 1.15 – 1.05 (m, 2H), 0.98 (d, J = 6.7 Hz, 3H, *minor*), 0.95 (d, J = 6.4 Hz, 3H, *major*), 0.89 (overlapping d, J = 6.8 Hz, 3H).

5.6: ^{13}C NMR (126 MHz, $CDCl_3$): δ 140.1, 119.2, 66.7, 49.8, 48.9, 46.2, 45.8, 38.1, 36.2, 29.8, 28.2, 27.0, 25.7, 17.5, 15.8.

S5.4: ^{13}C NMR (126 MHz, $CDCl_3$): δ 132.7, 125.7, 65.7, 46.6, 43.7, 41.0, 40.7, 39.5, 35.2, 31.8, 31.4, 30.4, 19.6, 16.5, 14.3.

HRMS (CI): *m/z* calculated for $C_{15}H_{24}O$ $[M + H]^+$ 221.1905, found 221.1911.

Selection of attempted elimination conditions:

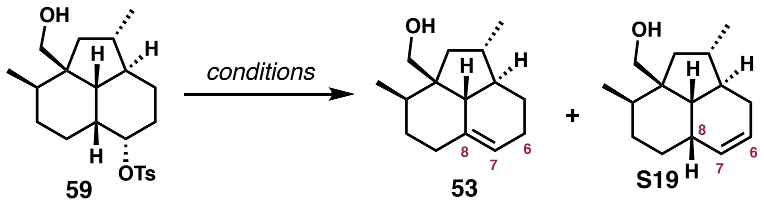

Entry	Base	Solvent, T [ºC]	Result [$\Delta^{7,8}$: $\Delta^{6,7}$; % conv.]
1	*n*-BuLi (0.9 equiv.)	THF, 70 ºC	NR
2	NaH (0.9 equiv.)	DMF, 90 ºC	1:1; *45%*
3	KO*t*-Bu (5 equiv.)	*t*-BuOH, 80 ºC†	NR
4	NaH (5 equiv.)	DME, 90 ºC	NR
5	NaH (5 equiv.)	dioxane, 100 ºC	NR
6	NaH (5 equiv.)	PhMe, 110 ºC	NR
7	NaH (5 equiv.)	DMAc, 135 ºC	2.5:1; *>95%*
8	NaH (9 equiv.)	DMF, 135 ºC	3:1; *>95%*
9	NaH (9 equiv.)	DMF, 135 ºC†	4:1; *>95%*
10	NaH (0.9 equiv.)	HMPA, 100 ºC	2:1; 60%
11	NaH (9 equiv.)	MeCN, 120 ºC	1.1:1; 40%
12	NaH (9 equiv.)	DMPU, 135 ºC†	1.6:1 *80%*

† reaction concentration was [0.01 M], all other entries: [0.025 M]

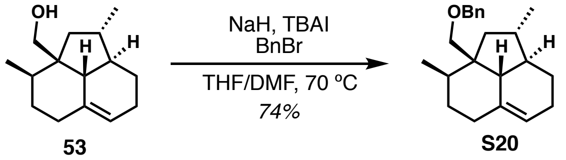

Benzyl ether S20: Alcohol **53** (41 mg, 0.19 mmol; 4:1 rr **53**/**S19**) was dissolved in 4:1

THF/DMF (1.9 mL) in a 2 dram vial affixed with a septum cap. NaH (9.4 mg, 0.37 mmol; 95% dry), TBAI (34 mg, 0.09 mmol), and BnBr (55 mL, 0.47 mmol) were added and the solution was heated to 70 °C under an Ar filled balloon. After 24 h, the reaction was cooled to rt, diluted with Et_2O (20 mL), and quenched with H_2O (10 mL). The aqueous phase was extracted with Et_2O (3 x 5 mL), and the combined organic extracts were washed with brine (5 x 10 mL), dried over $MgSO_4$, filtered, and concentrated *in vacuo*. The crude yellow oil was purified by column chromatography (pH 7 SiO_2, 100% hexanes) to give **S20** (42 mg, 74%, 4:1 mixture of alkene positional isomers). The resonances for the major regioisomer, **S20**, are reported below.

^{1}H NMR (500 MHz, $CDCl_3$): δ 7.40 – 7.31 (m, 5H), 5.20 – 5.13 (m, 1H), 4.52 (d, *J* = 4.1 Hz, 2H), 3.39 (q, *J* = 8.9 Hz, 2H), 2.31 – 2.21 (m, 2H), 2.17 – 2.05 (m, 2H), 2.02 (dd, *J* = 12.7, 6.7 Hz, 2H), 1.90 (ddd, *J* = 11.1, 6.8, 2.4 Hz, 1H), 1.73 – 1.63 (m, 1H), 1.55 – 1.47 (m, 1H), 1.41 – 1.30 (m, 3H), 1.12 – 1.04 (m, 2H), 1.03 – 0.98 (m, 1H), 0.94 (d, *J* = 6.4 Hz, 3H), 0.88 (d, *J* = 6.4 Hz, 3H).

^{13}C NMR (126 MHz, $CDCl_3$): δ 140.9, 139.4, 128.4 (2-C), 127.4, 127.3, 127.3, 118.7, 75.1, 73.6, 50.1, 49.8, 47.3, 45.1, 37.7, 36.4, 30.1, 28.4, 27.1, 25.9, 17.6, 15.9.

HRMS (CI): *m/z* calculated for $C_{22}H_{30}O$ $[M]^+$ 310.2297, found 310.2297.

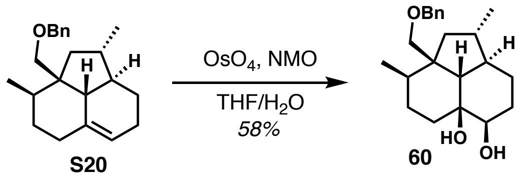

Diol 60: NMO (20 mg, 0.17 μmol) and OsO_4 (60 μL, 2.5 wt% in *t*-BuOH) were added to a solution of alkene **S20** (26.5 mg, 85 μmol; 4:1 rr) in THF (0.85 mL) and H_2O (0.43 mL). The resulting black solution was stirred for 18 h. The reaction was quenched with a 1:1 solution of $H_2O/Na_2S_2O_3$ (5 mL) and extracted with EtOAc (3 x 5 mL). The combined organic extracts were washed with brine (5 x 10 mL), dried over Na_2SO_4, filtered, and concentrated *in vacuo*. The crude diol was purified by column chromatography (SiO_2, 10:1 to 5:1 hexanes/ethyl acetate) to give **60** (17 mg, 58%) which could be separated from the undesired isomeric diol (not isolated).

^{1}H NMR (500 MHz, CDCl$_3$): δ 7.49 – 7.30 (m, 5H), 5.89 (s, 1H), 4.54 (d, *J* = 3.9 Hz, 2H), 3.68 (d, *J* = 9.2 Hz, 1H), 3.59 (t, *J* = 2.8 Hz, 1H), 3.39 (bs, 1H), 3.10 (d, *J* = 9.2 Hz, 1H), 1.99 (dq, *J* = 14.2, 3.0 Hz, 1H), 1.81 (qd, *J* = 13.1, 3.3 Hz, 1H), 1.70 (td, *J* = 13.3, 3.8 Hz, 1H), 1.64 – 1.57 (m, 1H), 1.57 – 1.52 (m, 2H), 1.51 – 1.44 (m, 4H), 1.42 – 1.33 (m, 2H), 1.34 – 1.22 (m, 2H), 0.96 (d, *J* = 5.8 Hz, 3H), 0.89 (d, *J* = 6.9 Hz, 3H).
^{13}C NMR (126 MHz, CDCl$_3$): δ 136.8, 128.8 (2-C), 128.3, 128.1 (2-C), 77.9, 74.0, 73.0, 72.3, 55.9, 48.8, 45.7, 45.0, 38.6, 38.4, 31.7, 29.0, 28.8, 23.7, 19.2, 15.8.
HRMS (ESI): *m/z* calculated for $C_{22}H_{32}O_3$ [M + Na]$^+$ 367.2249, found 367.2251.
[α]$^{22}_{D}$: –25.8 (c = 1, CHCl$_3$)

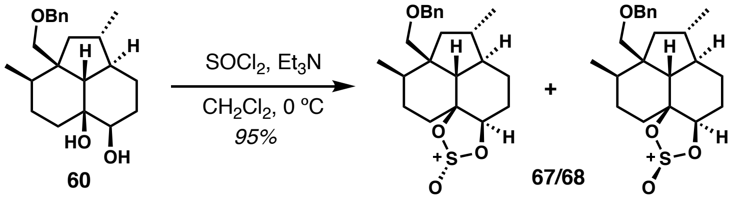

Cyclic sulfites 67 and 68: A stock solution of $SOCl_2$ (63 μL) in CH_2Cl_2 (940 μL) was prepared and a small portion of the stock solution (0.1 mL, 87 μmol) was added dropwise to a solution of diol **60** (15 mg, 44 μmol) and pyridine (18 μL, 17 μmol) in CH_2Cl_2 (0.9 mL) at 0 °C. After 30 min, the reaction was quenched by addition of H_2O (1 mL) and extracted with CH_2Cl_2 (3 x 3 mL). The combined organic extracts were washed with 0.5N HCl (5 mL) and brine (5 mL), dried over Na_2SO_4, filtered, and concentrated *in vacuo*. The crude yellow oil was purified by column chromatography (SiO_2, 10:1 hexanes/EtOAc) to give **67** and **68** (16.4 mg, 95%, 1.4:1 dr, relative configuration of each undetermined) as a slightly yellow semisolid. The diastereomers were separated for analytical purposes but were typically collected as a mixture for use in the following reaction.

Minor diastereomer (less polar) compound:

^{1}H NMR (600 MHz, CDCl$_3$): δ 7.37 (d, *J* = 6.6 Hz, 2H), 7.33 (t, *J* = 7.6 Hz, 2H), 7.25 (t, *J* = 7.5 Hz, 1H), 4.61 (d, *J* = 12.1 Hz, 1H), 4.41 (d, *J* = 12.2 Hz, 1H), 4.13 (dd, *J* = 3.4, 2.4 Hz, 1H), 3.57 (d, *J* = 9.0 Hz, 1H), 3.42 (d, *J* = 9.0 Hz, 1H), 2.57 (dd, *J* = 12.9, 1.8 Hz, 1H), 2.37 (ddt, *J* = 15.4, 3.7, 2.4 Hz, 1H), 2.21 (dd, *J* = 13.5, 9.7 Hz, 1H), 1.80 – 1.59 (m, 6H), 1.53 – 1.40 (m, 3H), 1.25 (tdd, *J* = 13.6, 11.0, 3.2 Hz, 1H), 1.06 (dd, *J* = 13.5, 9.1 Hz, 1H),

0.98 (d, J = 6.6 Hz, 3H), 0.92 (d, J = 6.9 Hz, 3H).

^{13}C NMR (151 MHz, $CDCl_3$): δ 139.5, 128.2 (2-C), 127.5 (2-C), 127.1, 89.3, 86.3, 73.3, 72.8, 52.5, 48.3, 45.9, 43.1, 38.8, 38.5, 30.9, 28.1, 27.8, 24.5, 19.3, 16.6.

Major diastereomer (more polar) compound:

^{1}H NMR (600 MHz, $CDCl_3$): δ 7.40 – 7.27 (m, 5H), 4.57 (t, J = 2.8 Hz, 1H), 4.56 – 4.46 (m, 2H), 3.55 (d, J = 8.6 Hz, 1H), 3.39 (d, J = 8.5 Hz, 1H), 2.39 (ddt, J = 15.6, 4.2, 2.1 Hz, 1H), 2.12 (dd, J = 13.3, 9.6 Hz, 1H), 2.00 (dd, J = 12.8, 1.5 Hz, 1H), 1.92 – 1.82 (m, 2H), 1.81 – 1.64 (m, 4H), 1.57 – 1.46 (m, 3H), 1.31 – 1.21 (m, 2H), 1.21 – 1.12 (m, 1H), 1.08 (dd, J = 13.2, 9.1 Hz, 1H), 0.97 (d, J = 6.6 Hz, 3H), 0.86 (d, J = 6.9 Hz, 3H).

^{13}C NMR (151 MHz, $CDCl_3$): δ 139.0, 128.4 (2-C), 127.53 (2-C), 127.48, 92.9, 80.2, 73.4, 71.2, 49.5, 47.4, 45.3, 43.6, 39.2, 38.3, 30.4, 27.8, 26.6, 24.7, 19.2, 15.9.

HRMS (ESI): *m/z* calculated for $C_{22}H_{30}O_4S$ [M + Na]$^+$ 413.1762, found 413.1756.

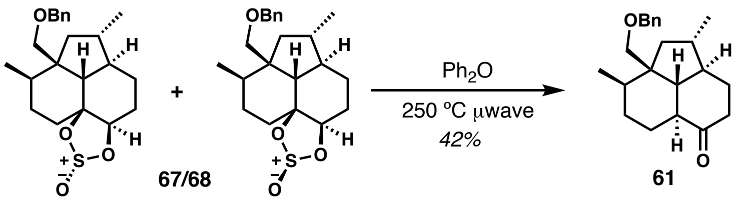

***trans*-Tricycle 61:** Cyclic sulfites **67** and **68** (8 mg, 20 μmol) were passed through a plug of pH 7 SiO_2 eluting with 5:1 hexanes/ethyl acetate into a 2 dram vial. The solvent was removed by concentrating *in vacuo* and then azeotroped with PhMe (2 x 2 mL). The starting material was then transferred to a microwave vial (G10) using alternating rinses of PhMe (2 x 0.2 mL) and Et_2O (2 x 0.2 mL). The solvent was removed *in vacuo* and the microwave vial was placed under vacuum (~200 mTorr) for 30 min. The vial was backfilled (3 x) with Ar before dry Ph_2O (0.45 mL; distilled over CaH_2 and stored in a Schlenk flask over molecular sieves) was added. The reaction vial was heated to 250 ºC for 50 min in a microwave reactor. Upon completion, the reaction solution was directly purified using column chromatography (pH 7 SiO_2 10:1 hexanes/EtOAc) to give **61** (2.8 mg, 42%).

^{1}H NMR (600 MHz, $CDCl_3$): δ 7.38 – 7.28 (m, 5H), 4.49 – 4.41 (m, 2H), 3.29 (d, J = 8.9 Hz, 1H), 3.20 (d, J = 8.9 Hz, 1H), 2.43 (ddd, J = 15.0, 5.2, 1.5 Hz, 1H), 2.36 – 2.26 (m, 2H), 2.17 – 2.09 (m, 1H), 2.04 (dd, J = 12.6, 5.9 Hz, 1H), 1.82 (ddd, J = 14.2, 9.7, 3.1 Hz,

1H), 1.70 – 1.63 (m, 1H), 1.63 – 1.58 (m, 2H), 1.51 – 1.45 (m, 2H), 1.41 (td, *J* = 11.1, 3.1 Hz, 1H), 1.36 – 1.28 (m, 1H), 1.26 – 1.21 (m, 1H), 1.15 (dd, *J* = 12.6, 11.3 Hz, 1H), 0.97 (d, *J* = 6.4 Hz, 3H), 0.89 (d, *J* = 6.5 Hz, 3H).

^{13}C NMR (151 MHz, $CDCl_3$): δ 213.1, 139.0, 128.4 (2-C), 127.4, 127.3 (2-C), 75.2, 73.6, 54.8, 53.9, 51.3, 49.3, 44.5, 41.5, 37.5, 35.6, 29.2, 27.9, 19.2, 17.6, 15.9.

HRMS (ESI): *m/z* calculated for $C_{22}H_{30}O_2$ $[M + Na]^+$ 349.2144, found 349.2148.

$[\alpha]^{22}_D$: –112.4 (c = 0.3, $CHCl_3$)

See Section E for additional spectra including DEPTQ, COSY, HSQC, and NOESY.

Attempted synthesis of isoneoamphilectane (Shenvi isocyanation):

Note: some intermediates are incompletely characterized because this route did not lead to the natural product and our focus transitioned to an alternative approach

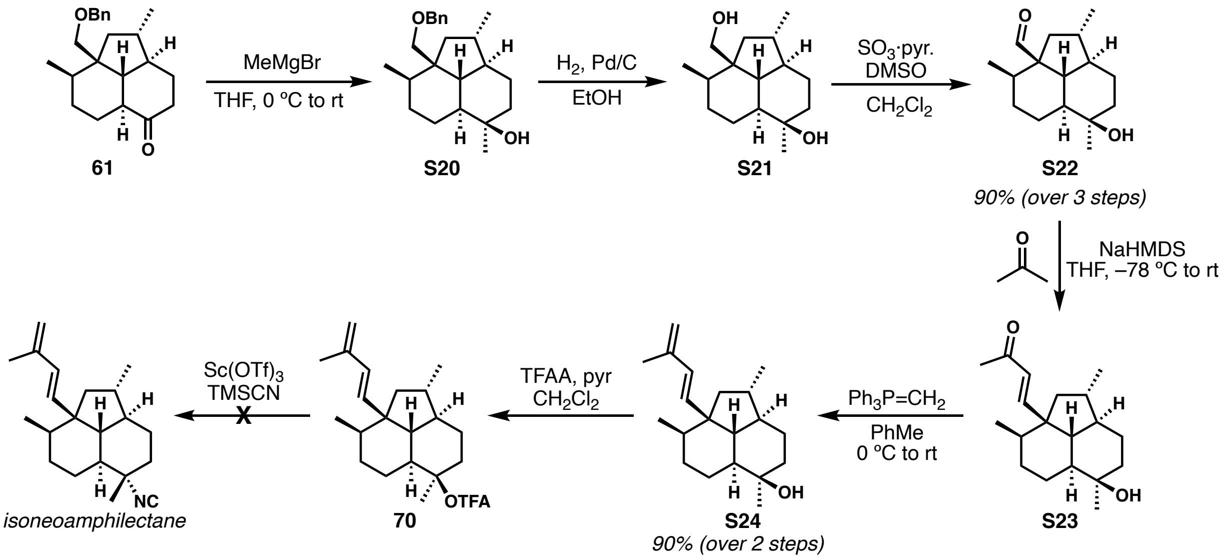

Scheme S1. Isoneoamphilectane synthesis thwarted by terminal isonitrile introduction

With *trans*-decalone **61** finally in hand, the synthesis of isoneoamphilectane (**1**) appeared within reach, given the endgames established for the two diastereomers (**12** and **43**) we had already made. Thus, we began with nucleophilic methylation of ketone **61** (Scheme S1), which from the *trans*-decalone provides the expected axial alcohol **S20**. Gratifyingly, the *trans*-ring junction is stable to these conditions and installing the tertiary alcohol immediately after *trans*-decalin formation avoided the risk of C8

epimerization throughout subsequent manipulations. Hydrogenolysis of the benzyl ether gives **S21** and subsequent oxidation under Parikh–Doering conditions efficiently furnished aldehyde **S22**. Installation of the diene proceeded through Wittig alkenylation as described in Schemes 4 and 5 in the main text; however, we found at this stage that a two-step sequence of aldol condensation with acetone to give **S23** and Wittig methylenation provided a much cleaner reaction profile and an increased yield of diene **S24**. To install the tertiary isonitrile using Shenvi's invertive isocyanation protocol, we converted the tertiary alcohol to the trifluoroacetate ester **70** and subjected it to the standard conditions of catalytic quantities of $Sc(OTf)_3$ in neat TMSCN with the expectation of obtaining the natural product, isoneoamphilectane (**1**). Unfortunately, all attempts at introducing the desired tertiary isonitrile were unproductive regardless of reaction temperature or source/activation mode of $Sc(OTf)_3$ (see below for details). Instead, these attempts resulted in the formation of two new products that appeared to be the result of complex carbocation rearrangements (likely driven by the relief of ring strain), and which could not be structurally characterized. The Shenvi isocyanation has proven to be a powerful transformation in many ICT syntheses in both their lab and ours; we attribute the significant ring strain in our substrate as the reason for the unfavorable outcome.

Tertiary alcohol S20: Methylmagnesium bromide (5 drops, ~3.0M in Et_2O) was added dropwise to a solution of ketone **61** (2.5 mg, 7 μmol; 85% pure containing ~15% *cis*-tricycle) in THF (0.12 mL) at 0 °C. The reaction stirred at 0 °C for 20 min, then rt for 10 additional min before it was quenched with sat. aq. NH_4Cl (2 mL). The mixture was extracted with EtOAc (3 x 5 mL) and the combined organic extracts were dried over $MgSO_4$, filtered, and concentrated *in vacuo*. The crude tertiary alcohol **S20** (2.5 mg) was used without purification in the next step.

^{1}H NMR (500 MHz, $CDCl_3$): δ 7.42 – 7.31 (m, 5H), 4.51 (d, *J* = 12.4 Hz, 1H), 4.45 (d, *J* = 12.4 Hz, 1H), 3.31 (d, *J* = 8.9 Hz, 1H), 3.20 (d, *J* = 8.9 Hz, 1H), 1.99 (dd, *J* = 12.5, 5.8 Hz, 1H), 1.80 – 1.71 (m, 3H), 1.66 – 1.59 (m, 2H), 1.59 – 1.54 (m, 1H), 1.53 – 1.45 (m, 1H),

1.43 – 1.27 (m, 6H), 1.18 (s, 3H), 1.13 – 1.03 (m, 2H), 0.91 (d, *J* = 6.4 Hz, 3H), 0.88 (d, *J* = 6.9 Hz, 3H).

Diol S21: Crude tertiary alcohol **S20** (2.5 mg) was dissolved in MeOH (0.6 mL) and Pd/C (2 mg; 10 % wt.) was added open to air. The atmosphere was exchanged for hydrogen and the solution was sparged for 1 min. After 18 h, the reaction was filtered through a short plug of Celite, washing with EtOAc (5 mL). The filtrate was concentrated *in vacuo*. The crude diol **S21** (2 mg) was used without purification in the next step.
^{1}H NMR (500 MHz, $CDCl_3$): δ 3.55 (d, *J* = 10.8 Hz, 1H), 3.40 (d, *J* = 10.8 Hz, 1H), 1.94 (dd, *J* = 12.8, 5.9 Hz, 1H), 1.82 – 1.73 (m, 2H), 1.72 – 1.65 (m, 1H), 1.61 – 1.46 (m, 5H), 1.45 – 1.27 (m, 7H), 1.21 (s, 3H), 1.15 – 1.06 (m, 1H), 0.92 (d, *J* = 6.4 Hz, 3H), 0.90 (d, *J* = 6.9 Hz, 3H).

Aldehyde S22: Triethylamine (10 μL, 70 μmol) was added to a solution of crude diol **S21** (2 mg) in CH_2Cl_2 (0.15 mL) and DMSO (75 μL) at 0 °C. SO_3·pyr (7.5 mg, 24 μmol; active SO_3 *ca.* 48–50%) was added. The solution was allowed to warm to rt overnight. After 20 h, the reaction was quenched with H_2O (1 mL) and extracted with $CHCl_3$ (3 x 3 mL). The combined organic extracts were washed with brine (2 x 5 mL), dried over Na_2SO_4, filtered, and concentrated *in vacuo*. The crude aldehyde was purified by column chromatography (pH 7 SiO_2, 10:1 to 3:1 hexanes/EtOAc) to give aldehyde **S22** (1.7 mg; 90% over 3 steps) as thin film.
^{1}H NMR (500 MHz, $CDCl_3$): δ 9.65 (s, 1H), 2.45 (dd, *J* = 12.9, 5.8 Hz, 1H), 1.85 (t, *J* = 11.3 Hz, 1H), 1.81 – 1.74 (m, 4H), 1.72 – 1.67 (m, 1H), 1.52 – 1.46 (m, 2H), 1.46 – 1.42 (m, 1H), 1.42 – 1.37 (m, 1H), 1.40 – 1.34 (m, 1H), 1.36 – 1.34 (m, 2H), 1.35 – 1.29 (m, 2H), 1.23 (s, 3H), 1.23 – 1.15 (m, 1H), 0.96 (d, *J* = 5.4 Hz, 3H), 0.94 (d, *J* = 4.9 Hz, 3H).
Enone S23: A stock solution was made using acetone (22 μL) in THF (200 μL) and a small portion (22 μL, 30 μmol) was added to a solution of NaHMDS (5.5 mg, 30 μmol) in THF (0.1 mL) at –78 °C. After 20 min, a solution of aldehyde **S22** (1.7 mg, 6 μmol) in THF (0.5 mL) was added dropwise over 20 min. After addition of the aldehyde, the reaction vial was removed from the cooling bath and immediately placed in a rt water bath. The

reaction was stirred at rt for 1 h. The reaction was diluted with Et_2O (5 mL) and quenched with sat. aq. $NaHCO_3$ (5 mL). The aqueous phase was extracted with Et_2O (3 x 5 mL) and the combined organic extracts were washed with brine (5 mL), dried over $MgSO_4$, filtered, and concentrated *in vacuo*. The crude enone **S23** was used in the following step without purification.

Diene S24: A suspension of $MePPh_3Br$* (357 mg, 1 mmol) and $NaNH_2$ (98 mg, 1.25 mmol, 50 wt.% in PhMe) in PhMe (3.3 mL) in a 2-necked conical flask was heated to reflux. After 2 h, the resulting yellow suspension was cooled to rt, the stirring was stopped, and the solids were allowed to settle to the bottom of the flask for 2.5 h. The resulting clear yellow, salt-free ylide solution was used in the following reaction. A solution of enone **S23** in PhMe (0.12 mL) was cooled to 0 °C and the methylene ylide (0.1 mL, 30 μmol; 0.3M in PhMe) was added dropwise. After addition, the solution was warmed to rt. After 30 min, the solution was diluted with Et_2O (5 mL) and quenched with brine (5 mL). The aqueous phase was extracted with Et_2O (3 x 5 mL) and the combined organic extracts were washed with brine (5 mL), dried over $MgSO_4$, filtered, and concentrated *in vacuo*. The crude diene was purified by column chromatography (pH 7 SiO_2, 100% hexanes to 10:1 hexanes/EtOAc) to give **S24** (1.7 mg, 90% over 2 steps) as a thin film.
*Note: $MePPh_3Br$ was dried by azeotroping with PhH and placing under vacuum (ca. 200 mTorr) overnight (12 h) prior to use.
^{1}H NMR (500 MHz, $CDCl_3$): δ 6.16 (d, *J* = 16.1 Hz, 1H), 5.68 (d, *J* = 16.1 Hz, 1H), 4.90 (s, 1H), 4.88 (s, 1H), 2.14 (dd, *J* = 12.7, 5.4 Hz, 1H), 1.84 (s, 3H), 1.81 – 1.73 (m, 2H), 1.72 – 1.66 (m, 1H), 1.64 – 1.58 (m, 1H), 1.50 – 1.43 (m, 1H), 1.42 – 1.37 (m, 1H), 1.36 – 1.32 (m, 2H), 1.32 – 1.28 (m, 4H), 1.21 (s, 3H), 1.19 – 1.13 (m, 2H), 1.02 (t, *J* = 12.2 Hz, 1H), 0.93 (d, *J* = 6.4 Hz, 3H), 0.78 (d, *J* = 6.5 Hz, 3H).

Tertiary trifluoroacetate 70: Pyridine (4 drops) and TFFA (2 drops) were added to a solution of tertiary alcohol **S24** (1.7 mg, 6 μmol) in CH_2Cl_2 (0.3 mL) at 0 °C. After 30 min, the reaction was carefully quenched by addition of ice water (0.5 mL) followed by sat. aq. $NaHCO_3$ (0.5 mL). The mixture was warmed to rt and extracted with CH_2Cl_2 (3 x 3 mL).

The combined organic extracts were dried over Na_2SO_4, filtered, and concentrated *in vacuo* to give the crude trifluoroacetate **70** which was treated to Shenvi's isocyanation without purification. Upon no conversion, purification by column chromatography (pH 7 buffered SiO_2, 100% hexanes) provided an analytically pure sample of **70** whose spectral data is reported below.

^{1}H NMR (500 MHz, $CDCl_3$): δ 6.20 (d, J = 16.1 Hz, 1H), 5.71 (d, J = 16.1 Hz, 1H), 4.90 (s, 2H), 2.87 (dt, J = 15.2, 3.4 Hz, 1H), 2.03 (dd, J = 12.6, 5.5 Hz, 1H), 1.85 (s, 3H), 1.85 – 1.76 (m, 2H), 1.75 – 1.67 (m, 2H), 1.60 (s, 3H), 1.48 – 1.36 (m, 5H), 1.22 – 1.15 (m, 2H), 1.11 (m. 2H), 0.92 (d, J = 6.4 Hz, 3H), 0.84 (d, J = 6.4 Hz, 3H).

^{13}C NMR (151 MHz, $CDCl_3$): δ 142.4, 136.9, 130.4, 114.6, 110.1, 90.9, 54.2, 50.1, 50.0, 48.4, 46.0, 37.1, 36.4, 36.1, 28.1, 26.9, 23.2, 18.9, 16.8, 15.7, 14.3, (–CF_3 quartet not observed).

HRMS (CI): *m/z* calculated for $C_{22}H_{31}F_3O_2$ $[M – OCF_3 + H]^+$ 271.2426, found 271.2413.

<u>**Synthesis of 7-formamidoisoneoamphilectane and isoneoamphilectane:**</u>

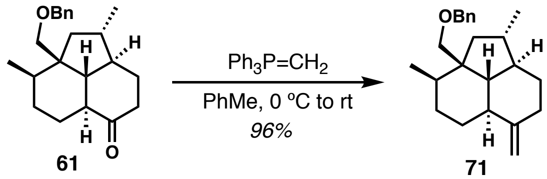

61 71

Alkene 71: A suspension of $MePPh_3Br$* (536 mg, 1.5 mmol) and $NaNH_2$ (146 mg, 1.88 mmol, 50 wt.% in PhMe) in PhMe (5 mL) in a 2-necked conical flask was heated to reflux. After 2 h, the resulting yellow suspension was cooled to rt, the stirring was stopped, and the solids were allowed to settle to the bottom of the flask overnight. The resulting clear yellow, salt-free ylide solution was used in the following reaction. A solution of ketone **61** (2.7 mg, 8 μmol) was dissolved in PhMe (0.16 mL) and cooled to 0 ºC. The methylene ylide solution (100 μL, 34 μmol; 0.3 M in PhMe) was added dropwise. After 20 min, the solution was diluted with Et_2O (5 mL) and quenched with brine (5 mL). The aqueous phase was extracted with Et_2O (3 x 5 mL) and the combined organic extracts were washed with brine (5 mL), dried over $MgSO_4$, filtered, and concentrated *in vacuo*. The

crude diene was purified by column chromatography (pH 7 SiO_2, 100% hexanes) to give **71** (2.5 mg, 96%).

*Note: $MePPh_3Br$ was dried by azeotroping with PhH and placing under vacuum (ca. 200 mTorr) overnight (12 h) prior to use.

^{1}H NMR (500 MHz, $CDCl_3$): δ 7.39 – 7.25 (m, 5H), 4.64 (d, *J* = 1.8 Hz, 1H), 4.50 – 4.44 (m, 2H), 4.43 (s, 1H), 3.29 (d, *J* = 8.9 Hz, 1H), 3.17 (d, *J* = 8.8 Hz, 1H), 2.42 (ddd, *J* = 13.9, 4.5, 1.9 Hz, 1H), 2.10 (td, *J* = 13.2, 5.4 Hz, 1H), 1.99 (dd, *J* = 12.5, 5.9 Hz, 1H), 1.95 (dq, *J* = 7.8, 2.5 Hz, 1H), 1.86 (t, *J* = 11.6 Hz, 1H), 1.77 – 1.60 (m, 3H), 1.53 (dd, *J* = 11.2, 5.9 Hz, 1H), 1.37 (td, *J* = 12.2, 11.7, 9.6 Hz, 1H), 1.25 – 1.20 (m, 1H), 1.17 (ddt, *J* = 16.5, 11.9, 5.5 Hz, 1H), 1.10 – 0.96 (m, 3H), 0.93 (d, *J* = 6.4 Hz, 3H), 0.90 (d, *J* = 6.7 Hz, 3H).

^{13}C NMR (151 MHz, $CDCl_3$): δ 154.3, 139.4, 128.9, 128.3 (2-C), 127.2 (2-C), 103.5, 76.0, 73.4, 56.8, 54.7, 48.4, 44.6, 43.4, 37.7, 36.4, 35.3, 31.1, 29.0, 22.6, 17.4, 16.2.

HRMS (CI): *m/z* calculated for $C_{23}H_{32}O$ $[M]^+$ 324.2453, found 324.2464.

$[\alpha]^{22}_D$: –152.3 (c = 0.25, $CHCl_3$)

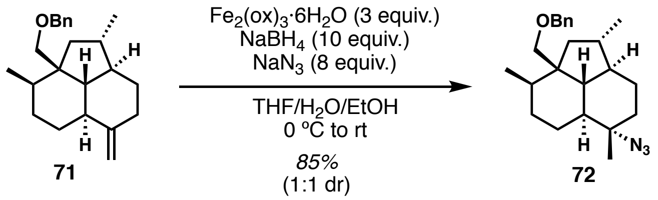

Azide 72: $Fe_2(ox)_3·6H_2O$ (18 mg, 37 μmol) was sonicated in H_2O (0.36 mL) until fully dissolved (approx. 1.5 h). The greenish yellow solution was degassed for 2 min and cooled to 0 °C. TsN_3 (9 mg, 140 mmol) was added, turning the solution a deep red color. Alkene **71** (3 mg, 9 μmol, 80% pure containing *cis*-tricycle)* was dissolved in THF (0.36 mL) and the catalyst/TsN_3 solution was added dropwise at 0 °C, rinsing the vial with EtOH (0.1 mL). $NaBH_4$ (3.6 mg, 280 μmol) was added in three portions in 10 min intervals. After addition of $NaBH_4$ was complete, the solution was warmed to rt for 30 min. The reaction was quenched with 30% aq. NH_4OH (1 mL) and H_2O (1 mL) and extracted with CH_2Cl_2 (3 x 5 mL). The combined organic extracts were dried over Na_2SO_4, filtered, and concentrated *in vacuo*. The crude azide was purified by column chromatography (pH 7 SiO_2, 100% hexanes to 10:1 hexanes/EtOAc) to give **72** (2.8 mg, 85%) as an inseparable

mixture of diastereomers (1:1 dr). Caution: the azide is not very soluble in ethyl acetate, so CH_2Cl_2 was added to aid loading the material onto the column chromatography.

*Due to material limitations of pure *trans*-decalone, we opted to use starting material that was contaminated with ~20% *cis*-decalone, this impurity was purified away at the formamide stage.

^{1}H NMR (500 MHz, $CDCl_3$): δ 7.40 – 7.29 (m, 5H), 4.57 – 4.43 (m, 2H), 3.30 (d, *J* = 8.9 Hz, 1H, one diastereomer), 3.28 (d, *J* = 8.8 Hz, 1H, one diastereomer), 3.20 (d, *J* = 8.9 Hz, 1H, overlapping diastereomers), 2.06 – 1.74 (m, 4H), 1.68 – 1.42 (m, 8H), 1.44 – 1.33 (m, 1H), 1.32 (s, 1.5H, one diastereomer), 1.25 – 1.16 (m, 1H), 1.14 (s, 1.5H, one diastereomer), 1.09 – 0.98 (m, 1H), 0.91 (d, *J* = 6.4 Hz, 1.5H, one diastereomer), 0.90 (d, *J* = 6.5 Hz, 1.5H, one diastereomer), 0.87 (d, *J* = 7.4 Hz, 1.5H, one diastereomer), 0.86 (d, J = 7.3 Hz, 1.5H, one diastereomer).

^{13}C NMR (126 MHz, $CDCl_3$) a mixture of 1:1 dr contaminated with 8-*epi*-axial-azide: δ 139.4, 139.3, 139.2, 128.39, 128.38, 128.34, 127.5, 127.4, 127.3, 127.3, 75.9, 75.6, 74.6, 73.5, 73.4, 66.0, 65.8, 65.2, 54.7, 54.5, 49.8, 49.3, 48.6, 48.1, 47.9, 47.7, 46.4, 45.6, 44.4, 43.8, 43.6, 42.1, 39.7, 39.1, 38.5, 38.1, 37.6, 37.6, 35.2, 34.7, 32.8, 32.1, 29.0, 29.0, 27.5, 27.2, 26.5, 24.6, 24.1, 23.8, 20.4, 20.0, 19.6, 17.9, 17.4, 17.3, 16.2, 16.1, 15.9.

HRMS (ESI): *m/z* calculated for $C_{22}H_{30}O_2$ $[M + Na]^+$ 390.2521, found 390.2537.

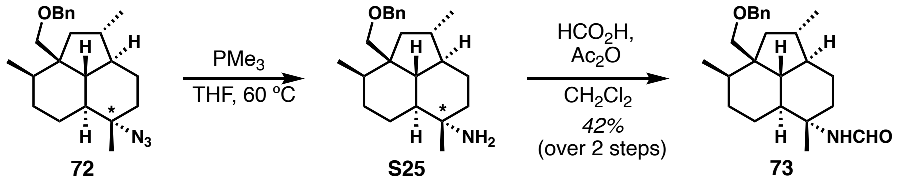

Tertiary amine S25: PMe_3 (0.16 mL, 0.16 mmol, 1.0 M in THF) was added to a solution of azide **72** (5.8 mg, 16 μmol; 1:1 dr at azide stereocenter) in THF (0.6 mL) and H_2O (3 drops). A screw cap was affixed to the vial and the reaction was heated to 60 ºC for 16 h. After cooling to rt, the reaction was concentrated *in vacuo* to give amine **S25** (5 mg) and the mixture of diastereomers was used in the following step without purification.

HRMS (ESI): *m/z* calculated for $C_{23}H_{35}NO$ $[M + Na]^+$ 342.2797, found 342.2805.

Formamide 73: Acetic formic anhydride was prepared by mixing equal parts acetic anhydride (0.2 mL) and formic acid (0.2 mL) and heating to 60 °C for 1 h. The crude amine **S25** was dissolved in CH_2Cl_2 (1.6 mL) and acetic formic anhydride (80 μL) was added at 0 °C. The reaction was allowed to warm to rt over 1 h before it was quenched with sat. aq. $NaHCO_3$ (1 mL) and extracted with CH_2Cl_2 (3 x 1 mL). The combined organic extracts were dried over Na_2SO_4, filtered, and concentrated *in vacuo*. The crude formamides were separable upon purification by column chromatography (pH 7 SiO_2, 5:1 hexanes/EtOAc to 2:1 hexanes/EtOAc) to give the desired equatorial formamide, **73** (2.5 mg, 42% over 2 steps, 1.7:1 *trans*/*cis* formamide rotamers) as a colorless solid.

^{1}H NMR (500 MHz, $CDCl_3$): 8.28 (d, *J* = 12.4 Hz, 1H, *trans*-rotamer), 8.06 (d, *J* = 2.2 Hz, 1H, *cis-rotamer*), 7.39 – 7.28 (m, 5H), 5.58 (bd, *J* = 12.4 Hz, 1H, –NH one rotamer), 5.13 (bs, 1H, –NH one rotamer), 4.53 (d, *J* = 12.4 Hz, 1H), 4.44 (d, *J* = 12.4 Hz, 1H), 3.27 (d, *J* = 8.8 Hz, 1H), 3.21 (d, *J* = 8.8 Hz, 1H), 2.18 (ddd, *J* = 13.4, 4.0, 2.6 Hz, 1H, one rotamer), 2.03 – 1.90 (m, 2H), 1.84 (dddt, *J* = 14.7, 12.5, 4.8, 2.8 Hz, 1H), 1.58 (s, 3H), 1.56 – 1.45 (m, 2H), 1.44 – 1.25 (m, 64), 1.23 (s, 3H, one rotamer), 1.20 (s, 3H, one rotamer), 1.12 – 1.00 (m, 1H), 1.02 – 0.94 (m, 1H), 0.91 (d, *J* = 6.5 Hz, 3H, one rotamer), 0.90 (d, *J* = 6.5 Hz, 3H, one rotamer), 0.86 (overlapping d, *J* = 6.9 Hz, 3H).

^{13}C NMR (126 MHz, $CDCl_3$): δ 163.0, 160.5, 139.3, 139.1, 128.4, 128.4, 127.5, 127.4, 127.34, 127.30, 75.8, 75.5, 73.4, 66.7, 58.9, 57.3, 54.8, 54.6, 49.3, 49.2, 48.6, 48.5, 48.2, 44.7, 44.4, 43.4, 38.9, 37.7, 37.6, 34.7, 34.6, 29.1, 27.4, 27.2, 20.0, 19.6, 19.0, 17.3, 16.0, 15.9.

HRMS (ESI): *m/z* calculated for $C_{24}H_{35}NO_2$ $[M + Na]^+$ 392.2566, found 392.2577.

$[\alpha]^{21}_D$: +47.2 (c = 0.4, $CDCl_3$)

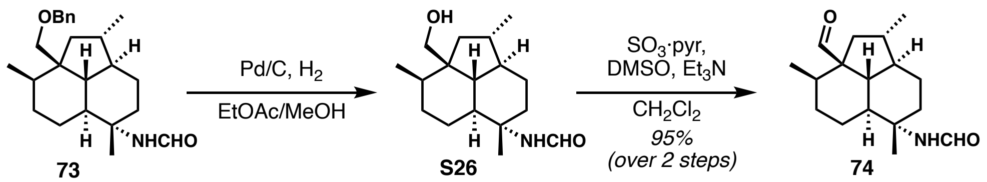

73 S26 74

Alcohol S26: Formamide **73** (2.5 mg, 7 μmol) was dissolved in EtOAc (0.8 mL) and MeOH (0.2 mL). Pd/C (3 mg) was added and the solution was sparged with hydrogen (1

min). After 14 h under a hydrogen atmosphere, the reaction was filtered through a plug of Celite and concentrated *in vacuo*. The crude alcohol **S26** (~2 mg; 1.6:1 *trans*/*cis* formamide rotamers) was used in the following step without purification.

^{1}H NMR (500 MHz, $CDCl_3$): 8.29 (d, J = 12.4 Hz, 1H, *trans*-rotamer), 8.07 (d, J = 2.2 Hz, 1H, *cis*-rotamer), 5.69 (bd, J = 12.2 Hz, 1H, –NH one rotamer), 5.14 (bs, 1H, –NH one rotamer), 3.57 (d, J = 10.6 Hz, 1H, *trans*-rotamer), 3.56 (d, J = 10.7 Hz, 1H, *cis*-rotamer), 3.38 (d, J = 10.6 Hz, 1H, *trans*-rotamer), 3.37 (d, J = 10.7 Hz, 1H, *cis*-rotamer), 2.16 – 2.09 (m, 1H), 2.01 – 1.93 (m, 1H), 1.92 – 1.81 (m, 1H), 1.69 – 1.58 (m, 3H), 1.56 – 1.43 (m, 3H), 1.37 – 1.32 (m, 2H), 1.26 (overlapping s, 6H), 1.17 – 1.08 (m, 1H), 1.01 (ddd, J = 14.7, 8.9, 3.4 Hz, 2H), 0.93 (d, J = 6.4 Hz, 3H, *trans*-rotamer), 0.92 (d, J = 6.4 Hz, 3H, *cis*-rotamer), 0.89 (d, J = 6.9 Hz, 3H, *trans*-rotamer), 0.89 (d, J = 6.8 Hz, 3H, *cis*-rotamer).

^{13}C NMR (126 MHz, $CDCl_3$): δ 163.1, 160.6, 67.9, 67.7, 58.8, 57.2, 54.6, 54.4, 48.8, 48.8, 48.1, 47.7, 47.6, 45.2, 45.1, 44.3, 43.3, 38.7, 38.1, 38.0, 34.6, 34.5, 29.0, 29.0, 27.3, 27.1, 20.1, 19.7, 19.6, 19.2, 17.37, 17.35, 16.0, 15.9.

HRMS (ESI): *m/z* calculated for $C_{17}H_{29}NO_2$ $[M + Na]^+$ 302.2096, found 302.2102.

$[\alpha]^{22}_{D}$: +10.5 (c = 0.4, $CDCl_3$)

Aldehyde 74: DMSO (32 μL) and Et_3N (22 μL, 0.16 mmol) were added to a solution of crude alcohol **S26** (~2 mg) in CH_2Cl_2 (0.2 mL) at 0 °C. The septum was briefly removed and SO_3·pyr (25 mg, 80 μmol; active SO_3 *ca*. 48–50%) was added. The reaction was allowed to warm to rt. After 4 h, the reaction was quenched with H_2O (2 mL) and extracted with $CHCl_3$ (3 x 5 mL). The combined organic extracts were washed with 1N HCl (2 mL), and brine (5 mL), dried over Na_2SO_4, filtered, and concentrated *in vacuo*. The crude aldehyde was passed through a short column (pH 7 SiO_2, 1:1 hexanes/EtOAc) to remove the amine salts which provided **74** (1.9 mg, 95% over 2 steps; 1.2:1 *trans*/*cis* formamide rotamers).

^{1}H NMR (500 MHz, $CDCl_3$): δ 9.72 (s, 1H, *trans*-rotamer), 9.70 (s, 1H, *cis*-rotamer), 8.30 (d, J = 12.3 Hz, 1H, *trans*-rotamer), 8.07 (d, J = 2.1 Hz, 1H, *cis*-rotamer), 5.69 (d, J = 12.4 Hz, 1H, –NH one rotamer), 5.14 (d, J = 5.1 Hz, 1H, –NH one rotamer), 2.39 (ddt, J = 12.3, 6.5, 3.3 Hz, 1H), 2.36 – 2.26 (m, 1H), 2.24 – 2.16 (m, 1H), 2.04 – 1.94 (m, 2H), 1.92 –

1.82 (m, 1H), 1.83 – 1.73 (m, 1H), 1.74 – 1.62 (m, 2H), 1.51 – 1.38 (m, 1H), 1.30 (s, 3H), 1.26 (s, 3H), 1.14 – 1.05 (m, 2H), 1.03 (d, J = 6.5 Hz, 3H, *trans*-rotamer), 0.99 (d, J = 6.6 Hz, 3H, *cis*-rotamer), 0.96 (d, J = 6.3 Hz, 3H, *trans*-rotamer), 0.94 (d, J = 6.5 Hz, 3H, *cis*-rotamer).

HRMS (ESI): *m/z* calculated for $C_{17}H_{27}NO_2$ $[M + Na]^+$ 300.1939, found 300.1939.

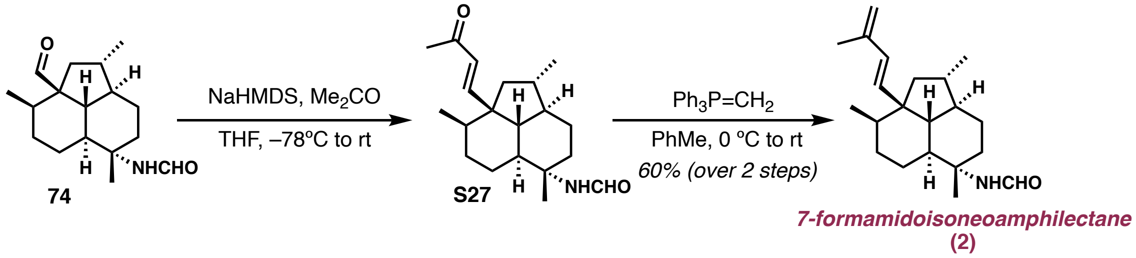

Enone S27: A stock solution was made using acetone (27 μL) in THF (100 mL) and a small portion (25 μL, 72 μmol) was added to a solution of NaHMDS (13 mg, 72 μmol) in THF (0.1 mL) at –78 °C. After 20 min, a solution of aldehyde **74** (~1.9 mg, 6 μmol) in THF (0.6 mL) was added dropwise over 20 min. After addition, the reaction vial was removed from the cooling bath and immediately placed in a rt water bath. The reaction was stirred at rt for 2 h before it was diluted with $CHCl_3$ (5 mL) and quenched with sat. aq. $NaHCO_3$ (5 mL). The aqueous phase was extracted with $CHCl_3$ (3 x 5 mL) and the combined organic extracts were washed with brine (5 mL), dried over Na_2SO_4, filtered, and concentrated *in vacuo*. The enone **S27** was used in the following step without purification.

HRMS (ESI): *m/z* calculated for $C_{20}H_{31}NO_2$ $[M + Na]^+$ 340.2253, found 340.2240.

7-formamido-isoneoamphilectane (2): A suspension of $MePPh_3Br$* (357 mg, 1 mmol) and $NaNH_2$ (98 mg, 1.25 mmol, 50 wt.% in PhMe) in PhMe (3.3 mL) in a 2-necked conical flask was heated to reflux. After 1.5 h, the yellow suspension was cooled to rt, the stirring was stopped, and the solids were allowed to settle to the bottom of the flask for 1 h. The resulting clear yellow, salt-free ylide solution was used in the following reaction. A solution of enones **S25** in PhMe (0.3 mL) was cooled to 0 °C and the methylene ylide (0.2 mL, 60 μmol; 0.3 M in PhMe) was added dropwise. After addition, the solution was warmed to rt. After 50 min, the solution was diluted with $CHCl_3$ (5 mL) and quenched with brine (5 mL).

The aqueous phase was extracted with $CHCl_3$ (3 x 5 mL) and the combined organic extracts were washed with brine (5 mL), dried over Na_2SO_4, filtered, and concentrated *in vacuo*. The crude diene was purified by column chromatography (pH 7 SiO_2, 3:1 hexanes/EtOAc) to give 7-formamidoisoneoamphilectane, **2** (1.3 mg, 1.6:1 *trans*/*cis* formamide rotamers, 60% over 2 steps) as thin film. The NMR data are listed below with the spectra referenced to 7.26 ppm and 77.0 ppm and are consistent with the isolation report.[7] The ^{13}C shifts are compared in the table below.
*Note: $MePPh_3Br$ was dried by azeotroping with PhH and placing under vacuum (ca. 200 mTorr) overnight (12 h) prior to use.

^1H NMR (500 MHz, $CDCl_3$): δ 8.29 (d, *J* = 12.4 Hz, 1H, *trans*-rotamer), 8.06 (d, *J* = 2.2 Hz, 1H, *cis*-rotamer), 6.12 (d, *J* = 16.1 Hz, 1H), 5.67 (d, *J* = 16.1 Hz, 1H, *cis*-rotamer), 5.66 (d, *J* = 16.1 Hz, 1H, *trans*-rotamer), 5.62 (bs, 1H*,–NH*), 5.13 (bs, 1H, *,–NH*), 4.91 (s, 2H), 4.90 (s, 2H, *trans*-rotamer), 2.16 – 2.07 (m, 2H), 2.05 – 1.91 (m, 2H), 1.84 (s, 3H), 1.65 – 1.58 (m, 2H), 1.53 – 1.42 (m, 2H), 1.30 – 1.25 (m, 2H), 1.26 (s, 3H, *cis*-rotamer), 1.25 (s, 3H, *trans*-rotamer), 1.24 – 1.20 (m, 1H), 1.19 – 1.13 (m, 1H), 1.08 – 1.02 (m, 1H), 0.93 (d, *J* = 6.3 Hz, 3H, *trans*-rotamer), 0.92 (d, *J* = 6.3 Hz, 3H, *cis*-rotamer), 0.78 (d, *J* = 6.4 Hz, 3H, *trans*-rotamer), 0.77 (d, *J* = 6.4 Hz, 3H, *cis*-rotamer).
^{13}C NMR (126 MHz, $CDCl_3$): δ 162.9, 160.4, 142.4, 142.2, 137.6, 137.1, 129.9, 129.6, 114.6, 114.4, 58.7, 57.1, 54.5, 54.3, 53.9, 53.6, 48.88, 48.85, 47.8, 46.12, 46.05, 44.1, 43.1, 38.6, 37.33, 37.25, 35.7, 35.6, 28.3, 28.1, 27.1, 27.0, 19.9, 19.6, 19.5, 19.0, 18.90, 18.88, 16.9 (2C), 15.80, 15.75.
HRMS (ESI): *m/z* calculated for $C_{21}H_{33}NO$ $[M + Na]^+$ 338.2460, found 338.2461.
$[\alpha]^{22}_D$: +75.5 (c = 0.26, $CHCl_3$) [synthetic]
$[\alpha]^{20}_D$: +44.0 (c =1.0, $CHCl_3$) [literature]

Synthetic	Literature	Δ
162.9	163.0	+0.1
160.4	160.4	0
142.4	142.4	0
142.2	142.2	0
137.6	137.6	0
137.1	137.1	0
129.9	129.8	–0.1

129.6	129.6	0
114.6	114.6	0
114.4	114.4	0
58.7	58.6	–0.1
57.1	57.0	–0.1
54.5	54.4	–0.1
54.3	54.3	0
53.9	53.8	–0.1
53.6	53.6	0
48.9	48.9	0
48.9	48.8	–0.1
47.8	47.7	–0.1
46.1	46.1	0
46.1	46.0	–0.1
44.1	44.1	0
43.1	43.0	–0.1
38.6	38.6	0
37.3	37.3	0
37.3	37.2	–0.1
35.7	35.6	–0.1
35.6	35.6	0
28.3	28.3	0
28.1	28.1	0
27.1	27.1	0
27.0	26.9	–0.1
19.9	19.9	0
19.6	19.5	–0.1
19.5	19.3	–0.2
19.0	18.9	–0.1
18.9	18.9	0
18.9	18.8	–0.1
16.9	16.9	0
16.9	16.8	–0.1
15.8	15.8	0
15.8	15.7	–0.1

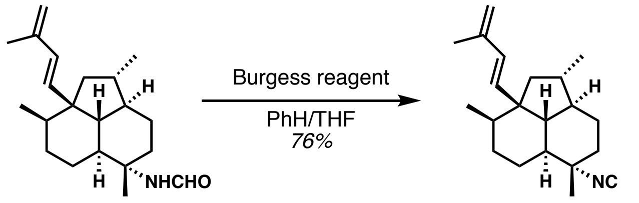

7-formamidoisoneoamphilectane
(2)

isoneoamphilectane
(1)

Isoneoamphilectane (1): Burgess reagent (4.9 mg, 21 μmol) was added to a solution formamide **2** (1.3 mg, 4 μmol) in a 1:1 mixture of PhH/THF (0.16 mL). After 15 h, the reaction was filtered through a short plug of SiO_2, rinsing with EtOAc (10 mL). The filtrate was concentrated *in vacuo* and the crude product was further purified by column

chromatography (SiO_2, 100% hexanes to 20:1 hexanes/EtOAc) to afford isoneoamphilectane **1**(0.9 mg, 76%) as a thin film. The NMR data are listed below with the spectrum referenced to 7.26 ppm and are consistent with the isolation report.[8] The ^{13}C shifts are compared in the table below.

^{1}H NMR (500 MHz, $CDCl_3$): δ 6.10 (d, J = 16.1 Hz, 1H), 5.64 (d, J = 16.1 Hz, 1H), 4.90 (s, 1H), 2.14 (dd, J = 12.8, 5.6 Hz, 1H), 2.13 – 2.05 (m, 1H), 1.88 – 1.83 (m, 1H), 1.84 (s, 3H), 1.82 – 1.79 (m, 1H), 1.78 – 1.73 (m, 1H), 1.71 – 1.66 (m, 1H), 1.60 – 1.56 (m, 1H), 1.48 – 1.39 (m, 1H), 1.32 (s, 3H and m, 1 H), 1.17 – 1.08 (m, 2H), 1.09 – 1.00 (m, 1H), 0.99 – 0.95 (m, 1H), 0.93 (d, J = 6.4 Hz, 3H), 0.79 (d, J = 6.7 Hz, 3H).

^{13}C NMR (126 MHz, $CDCl_3$): δ 142.2, 136.9, 129.9, 114.7, 61.3, 54.2, 53.0, 48.7, 47.2, 46.1, 42.0, 37.3, 35.7, 28.0, 26.6, 20.3, 20.1, 18.9, 16.8, 15.7 (–NC carbon not observed).

HRMS (CI): *m/z* calculated for $C_{21}H_{31}N$ $[M]^+$ 297.2456, found 297.2459.

$[\alpha]^{22}_{D}$: +61.8 (c = 0.2, $CHCl_3$) [synthetic]

$[\alpha]^{25}_{D}$: +67.0 (c = 0.79, $CHCl_3$) [literature]

$[\alpha]^{20}_{D}$: +57.0 (c = 1, $CHCl_3$) [literature]

Synthetic	Literature	Δ
142.2	142.2	0
136.9	136.9	0
129.9	130.0	+0.1
114.7	114.7	0
61.3	61.3	0
54.2	54.2	0
53.0	53.0	0
48.7	48.8	+0.1
47.2	47.2	0
46.1	46.1	0
42.0	42.0	0
37.3	37.3	0
35.7	35.7	0
28.0	28.0	0
26.6	26.6	0
20.3	20.3	0
20.1	20.1	0
18.9	18.8	–0.1
16.8	16.8	0
15.7	15.7	0

C. Computational Studies and Procedures

Relative Energy Calculations:

All calculations were performed using Spartan 18[9] in the gas phase. First, a conformer search was performed using Molecular Mechanics MMFF and the resulting conformer geometries were further optimized using HF/3-21G. Next, the conformers were subjected to geometry optimization at the ωB97X-D/6-31G* level. The resulting single point energies of the lowest energy conformers were compared (**1** to **12** and **53** to **56**) and the absolute difference between the two lowest energy conformers is reported in the text.

Calculated lowest energy conformer of **1**:

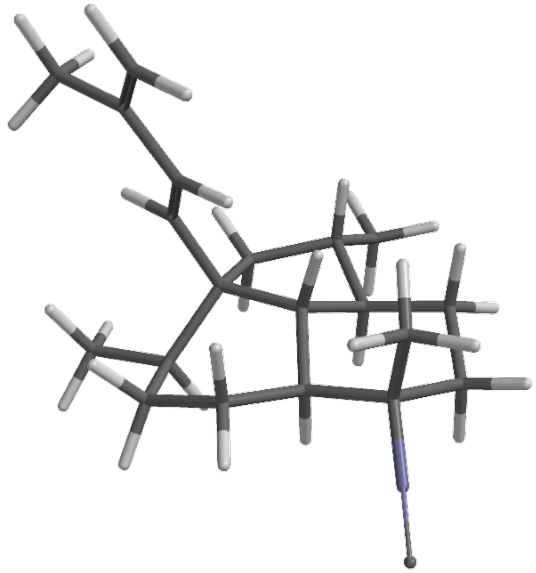

53

C	-0.466752	-1.758526	-1.473200
C	-0.803185	-0.616496	-0.459812
C	0.521451	-0.238138	1.769249
C	-1.983755	0.020864	1.717724
C	-0.646692	0.649154	2.189382
C	-1.843783	-1.046494	0.609404
C	0.557214	-0.374761	0.251262
C	1.479720	-1.514328	-0.163336
C	2.909574	-1.144870	0.214967
C	1.941044	0.164096	2.255814
C	2.979096	-0.868794	1.729245
C	1.068575	-1.772220	-1.612106
H	1.202801	-2.415115	0.411498

C	1.627788	-3.047360	-2.228708
C	-1.327184	0.582349	-1.222446
C	-0.945935	1.860810	-1.134891
C	-1.528481	2.975658	-1.896713
C	-1.065448	4.218378	-1.712946
C	-2.641483	2.684674	-2.869192
C	-3.214612	-1.406965	0.036419
H	0.990745	0.540084	-0.166720
H	0.348999	-1.233792	2.202509
C	2.340692	1.599883	1.886502
H	-0.976001	-1.634910	-2.434922
H	-0.786861	-2.727391	-1.063492
H	-2.646655	0.816025	1.357796
H	-2.494861	-0.447426	2.567215
H	-0.531203	1.641092	1.738141
H	-0.652642	0.793456	3.274893
H	-1.437398	-1.960515	1.066814
H	3.226870	-0.262774	-0.358233
H	3.615158	-1.948749	-0.025187
H	3.981684	-0.532865	2.016920
H	2.797412	-1.810233	2.262289
H	1.374587	-0.909161	-2.222519
H	1.241354	-3.202672	-3.241932
H	1.351290	-3.921721	-1.626105
H	2.721382	-3.013392	-2.290087
H	-2.135161	0.339384	-1.911692
H	-0.145643	2.146044	-0.454739
H	-1.473147	5.066948	-2.254666
H	-0.262229	4.427341	-1.010187
H	-2.982299	3.597869	-3.363575
H	-2.313442	1.978997	-3.641759
H	-3.500275	2.230722	-2.360113
H	-3.713570	-0.529305	-0.389473
H	-3.137347	-2.168295	-0.747869
H	-3.864718	-1.803927	0.823661
H	3.321963	1.828478	2.312142
H	2.402708	1.727537	0.803032
H	1.620532	2.320598	2.283228
C	1.945141	0.018661	4.865009
N	1.952981	0.082089	3.691503

Calculated lowest energy conformer of **12**:

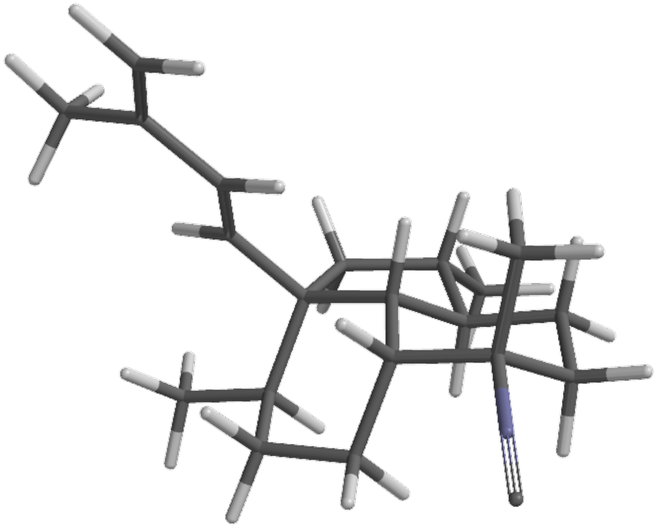

53

C	-1.176824	-0.370970	-1.965084
C	-0.544272	0.482541	-0.808934
C	0.233950	-0.193340	1.623749
C	-1.231767	1.805612	1.269871
C	-0.925451	0.628361	2.194742
C	-1.654634	1.348246	-0.128456
C	-0.039328	-0.607388	0.170508
C	-1.073462	-1.727980	0.036095
C	-0.652753	-2.968066	0.811404
C	0.611615	-1.457101	2.456981
C	-0.416578	-2.599119	2.280360
C	-1.231545	-1.842379	-1.481967
H	-2.033026	-1.386808	0.453232
C	-2.478734	-2.592444	-1.935941
C	0.544213	1.371268	-1.359213
C	1.829706	1.413640	-0.993651
C	2.849598	2.296406	-1.579452
C	4.107818	2.252267	-1.125205
C	2.442141	3.238152	-2.682158
C	-2.104519	2.542349	-0.973854
H	0.892359	-1.003590	-0.258332
H	1.113566	0.463647	1.645572
C	2.032231	-1.932976	2.110812
H	-0.598891	-0.283238	-2.890390
H	-2.188174	-0.012524	-2.192124
H	-0.343653	2.448457	1.186547
H	-2.027104	2.422819	1.704918
H	-0.664087	0.989980	3.193194
H	-1.826060	0.011084	2.310844
H	-2.528553	0.692416	-0.004131

H	0.258000	-3.393744	0.367935
H	-1.420980	-3.747943	0.755650
H	-0.073466	-3.467005	2.854006
H	-1.364816	-2.280308	2.728948
H	-0.346990	-2.370864	-1.865981
H	-2.551269	-2.619492	-3.028591
H	-3.382915	-2.104002	-1.552352
H	-2.475706	-3.627422	-1.576548
H	0.215767	2.033335	-2.159508
H	2.192244	0.757387	-0.204458
H	4.885773	2.892221	-1.531529
H	4.399898	1.573933	-0.327400
H	3.291819	3.833460	-3.026239
H	2.039012	2.688921	-3.541089
H	1.658773	3.925534	-2.341416
H	-1.336662	3.323061	-0.999966
H	-2.330940	2.257255	-2.007578
H	-3.009781	2.985738	-0.546233
H	2.107376	-2.227588	1.061787
H	2.302525	-2.794017	2.729332
H	2.754788	-1.133111	2.298384
C	0.642225	-0.829533	4.992523
N	0.627534	-1.105139	3.850389

Calculated lowest energy conformer of **53**:

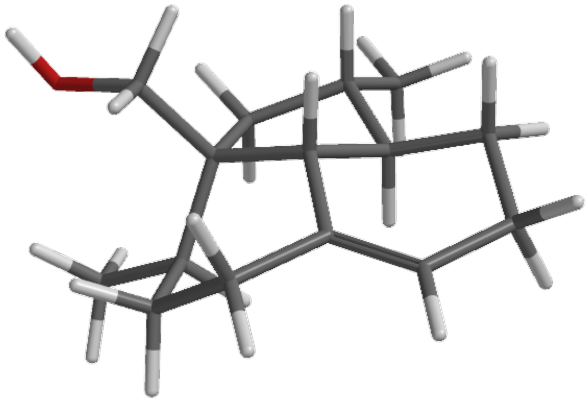

40

C	-0.212846	0.027576	-1.838413
C	0.433453	0.705790	-0.589608
C	0.359256	-0.160117	1.871966
C	0.110821	2.307156	1.384178
C	0.755532	1.226925	2.292470
C	-0.486959	1.763128	0.070403
C	0.576337	-0.475240	0.416902
C	-0.413922	-1.527541	-0.068922
C	-0.248635	-2.796169	0.752204
C	-0.157981	-1.093776	2.674548
C	-0.574024	-2.479057	2.228387
C	-0.206921	-1.496093	-1.581556
C	-1.242351	-2.262011	-2.394578
H	-1.430366	-1.141823	0.119620
H	1.588961	-0.900113	0.305541
C	1.828299	1.230539	-0.937536
C	-0.921310	2.924019	-0.826756
O	1.751783	2.235442	-1.931210
H	0.294709	0.312030	-2.764595
H	-1.256452	0.351646	-1.940797
H	0.850800	3.082464	1.148667
H	-0.694255	2.813800	1.929064
H	1.850232	1.303762	2.220285
H	0.505596	1.400778	3.344472
H	-1.397263	1.206883	0.344190
H	0.785674	-3.154563	0.661333
H	-0.899608	-3.602533	0.394658
H	-0.323157	-0.860715	3.726681
H	-0.114297	-3.229873	2.883763
H	-1.657765	-2.576550	2.388465
H	0.792314	-1.899611	-1.807052
H	-1.078940	-2.133107	-3.470296

H	-2.253932	-1.904608	-2.164513
H	-1.207205	-3.335415	-2.176670
H	2.428495	0.379123	-1.299291
H	2.324247	1.612994	-0.031448
H	-0.071628	3.557174	-1.091381
H	-1.668537	3.536785	-0.308366
H	-1.369258	2.569679	-1.760617
H	2.651104	2.481220	-2.174189

Calculated lowest energy conformer of **56**:

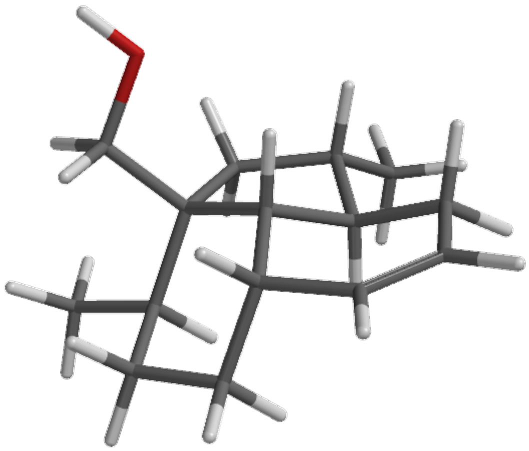

40

C	-0.219072	0.100397	-1.876019
C	0.442467	0.730923	-0.609167
C	0.770122	-0.233728	1.831238
C	-0.188100	2.075535	1.496834
C	-0.234414	0.808312	2.345480
C	-0.518464	1.776317	0.028633
C	0.641380	-0.489609	0.327433
C	-0.506195	-1.436293	-0.021417
C	-0.295258	-2.804116	0.620020
C	0.636188	-1.537967	2.581331
C	0.165279	-2.668354	2.051242
C	-0.522388	-1.381306	-1.552587
C	-1.799275	-1.907488	-2.196616
H	-1.452268	-1.019817	0.359275
H	1.562295	-0.991481	0.004037
H	1.775948	0.169471	2.027428
C	1.789120	1.372114	-0.957671
O	2.644009	0.380995	-1.500984

C	-0.634038	3.075107	-0.776031
H	0.432244	0.180275	-2.749902
H	-1.149691	0.626867	-2.120341
H	0.804615	2.541977	1.576094
H	-0.904465	2.812655	1.881126
H	-0.026451	1.036187	3.398226
H	-1.247404	0.385696	2.311787
H	-1.513664	1.308364	0.027394
H	0.457699	-3.369758	0.049135
H	-1.214562	-3.402569	0.581397
H	0.934562	-1.526564	3.629130
H	0.108737	-3.560591	2.672566
H	0.324736	-1.986417	-1.904901
H	-1.757956	-1.827105	-3.288592
H	-2.669523	-1.335458	-1.852207
H	-1.968132	-2.960531	-1.945755
H	2.239229	1.818934	-0.056813
H	1.634902	2.179112	-1.688382
H	3.463961	0.810383	-1.770275
H	0.269362	3.688835	-0.682941
H	-1.472498	3.675910	-0.407518
H	-0.803037	2.884787	-1.841687

<u>Predicted NMR shifts for 7-amino- and 8-epi-7-amino-isoneoamphilectane:</u>

All calculations were performed using Spartan 18 on structures **4** and 8-*epi*-**4** in the gas phase. First, a distribution of conformers was calculated with Molecular Mechanics MMFF and of the resulting conformers, those exceeding 13 kcal·mol^{-1} were discarded. The remaining conformers were subjected to an equilibrium geometry calculation using HF/3-21G and then assigned an alignment score. Any duplicates (conformers with the same alignment score) were discarded as well as any conformers exceeding 5 kcal/mol. All remaining conformers were refined using a single point energy calculation with ωB97X-D/6-31G* and all conformers exceeding 3 kcal·mol^{-1} were discarded. At this point the remaining conformers were subjected to a final energy refinement with ωB97XD/6-311+G(2df,2p)[6-311G*] with NMR prediction using ωB97X-D/6-31G*. Each of the conformers were assigned a Boltzmann weight and the Boltzmann averaged ^{1}H shifts and ^{13}C shifts are reported above.

We calculated the ^{13}C and ^{1}H NMR data for both the *cis* and *trans* natural product structures to compare to the reported, isolated sample to investigate the probability of our

hypothesis. For computational simplicity we opted to use Rodríguez's semi-synthetic primary amine, isoneoamphilectane **4**, in our calculations. Unfortunately, the data were inconclusive in that neither of the predicted shifts for *cis*-**4** or *trans*-**4** matched particularly well with the reported sample. In terms of the ^{13}C NMR data, the values correlated slightly better with *trans*-fused **4**; the absolute average difference in ppm between the isolated sample of **4** and the predicted data was 2.0 ppm. In contrast, the absolute average deviation between the reported data for **4** and predicted 8-*epi*-**4** was 3.6 ppm. We also analyzed the predicted proton NMR data—although typically not as accurate as calculated ^{13}C NMR for predictive studies—of the two possible epimers at C-8. Many of the predicted resonances for the two possible natural products were identical, 8 of the predicted *trans* signals and 6 of the *cis* signals were more closely aligned with the reported proton NMR of **4**. Our results highlight how difficult the characterization of these compounds can be, particularly by analysis of ^{1}H NMR data, with minor changes to the structure having a minimal effect on the resonances.

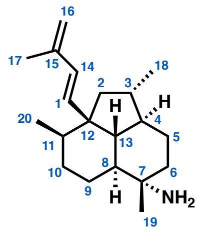

7-amino-isoneoamphilectane (4)

^{13}C and ^{1}H predicted NMR shifts for 7-amino-isoneoamphilectane and 8-*epi*-7-amino-isoneoamphilectane. Numbers listed in tables are chemical shifts in ppm and coupling constants in Hz (in parentheses). The colored cells indicate which compound more closely matches the reported sample.

C #	Reported 4	Predicted 4 (*trans*)	Predicted 8-*epi*–4 (*cis*)
1	137.9	135.6	133.2
2	49.0	46.6	43.5
3	37.3	34.9	36.3
4	55.0	51.1	41.4
5	27.7	26.0	25.6
6	44.3	42.2	35.4
7	53.2	52.3	51.1
8	49.1	46.9	43.1
9	19.6	18.8	21.5
10	28.6	26.9	29.4
11	35.7	35.8	37.9
12	46.1	46.3	47.5
13	54.9	50.2	47.3
14	129.4	126.4	127.6
15	142.5	138.6	138.6
16	114.1	114.9	115.0
17	18.9	17.4	17.3
18	17.0	15.4	17.2
19	21.6	20.0	26.9
20	15.9	14.8	15.3
		|Δ|: 2.0	|Δ|: 3.6

H#	Reported 4	Predicted 4 (*trans*)	Predicted 8-*epi*–4 (*cis*)
1	5.67 (d, 16.1)	5.6 (d, 16.8)	5.6 d (16.8)
2	2.12 m	2.1 (d, 4.5)	1.5 (d, 9.1)
2’	1.02 m	0.8 (d, 12.3)	1.2 (d, 8.5)
3	1.42 m	1.5 m	1.3 m
4	0.9 m	0.9 (ddd, 11.8, 11.7, 3.0)	1.4 (dd, 12.6, 12.2)
5	1.79 m	0.9 (d, 3.8)	0.9 (dd, 12.6, 12.2)
5’	1.14 m	1.4 (ddd, 4.3, 3.0, 2.7)	1.4 (3.9, 2.9)
6	1.31 m	1.3 (dd, 3.8, 2.7)	1.3 (dd, 3.7, 2.9)
6’	1.7 m	0.9 (4.3)	1.0 (d, 3.9)
8	1.28 m	1.0 (d, 1.8)	1.4 (ddd 12.8, 4.3, 3.8)
9	1.69 m	2.0 (ddd, 9.8, 1.8, 0.5)	1.2 (dd, 3.8, 3.1)
9’	1.01 m	0.9 (dd, 9.5, 8.5)	1.0 (d, 12.8)
10	1.27 m	1.2 (ddd, 10.8, 9.5, 0.5)	0.9 (dd, 12.6, 3.1)
10’	1.60 m	1.4 (dd, 9.8, 8.5)	1.1 (2.5)
11	1.58 m	1.3 (dq, 10.8, 6.5)	1.3 m
13	1.23 m	1.1 (d, 11.7)	1.8 dd (12.2, 4.3)
14	6.11 (d, 16.4)	5.8 (d, 16.8)	5.7 d (16.8)
16	4.89 br s	4.7 s	4.6 s
16’	“	4.6 s	4.7 s
17	1.84 s	1.4 s	1.4 s
18	0.91 (d, 6.4)	0.7 (d, 6.4)	0.7 (d, 6.4)
19	1.0 s	0.6 (s)	0.9 (s)
20	0.76 (d, 6.4)	0.5 d (6.5)	0.6 d (6.5)

Cartesian coordinates for largest contributor to NMR shift calculation (the lowest energy conformer of 15 contributing conformers) of **4**:

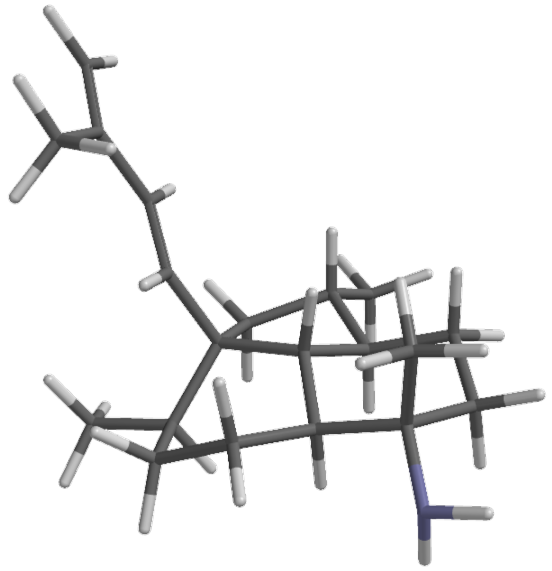

54

C	-1.741799	-0.026763	-1.374139
C	-0.719366	0.660370	-0.423786
C	-0.021820	-0.392122	1.895739
C	-0.433638	2.089003	1.705268
C	0.535883	0.994066	2.215550
C	-1.391073	1.643073	0.571441
C	-0.135182	-0.552310	0.381305
C	-0.982508	-1.766840	0.022102
C	-0.245584	-3.022806	0.476916
C	0.712694	-1.619907	2.499046
C	0.016884	-2.918895	1.993187
C	-1.345816	-1.515298	-1.442335
H	-1.916847	-1.715785	0.585680
C	-2.433395	-2.418241	-2.015492
C	0.418193	1.359353	-1.151079
C	0.660680	1.420097	-2.452726
C	1.818718	2.093062	-3.079716
C	1.959448	2.066204	-4.398189
C	2.820699	2.800725	-2.200775
C	-2.031027	2.860074	-0.101183
H	-1.028404	-0.431873	2.314005
H	0.861887	-0.733958	-0.007581
C	2.211316	-1.653279	2.172519
N	0.620361	-1.515503	3.957242
H	-1.815984	0.440416	-2.347414

H	-2.733865	0.036250	-0.932657
H	0.137242	2.955974	1.383817
H	-1.051275	2.435835	2.529663
H	1.504051	1.109955	1.736360
H	0.703804	1.106846	3.279347
H	-2.196464	1.081617	1.039473
H	0.687981	-3.125731	-0.072011
H	-0.823033	-3.922955	0.281929
H	0.594879	-3.776711	2.331113
H	-0.946362	-2.987148	2.496942
H	-0.448545	-1.617154	-2.051821
H	-2.116677	-3.456864	-2.035646
H	-2.684960	-2.133602	-3.033290
H	-3.342379	-2.359425	-1.421985
H	1.124044	1.833895	-0.490950
H	-0.008603	0.951075	-3.151675
H	2.787285	2.543297	-4.891620
H	1.249522	1.564252	-5.032269
H	3.281629	2.112269	-1.498375
H	2.345581	3.586075	-1.620010
H	3.606033	3.250019	-2.795925
H	-1.289908	3.470661	-0.606275
H	-2.773804	2.566697	-0.836288
H	-2.528246	3.484210	0.636283
H	2.409190	-1.732313	1.110964
H	2.706657	-0.768501	2.552027
H	2.670784	-2.515395	2.649390
H	1.079040	-2.292507	4.395622
H	-0.337922	-1.543485	4.252282

Cartesian coordinates for largest contributor to NMR shift calculation (the lowest energy conformer of 15 contributing conformers) of **8-epi-4**:

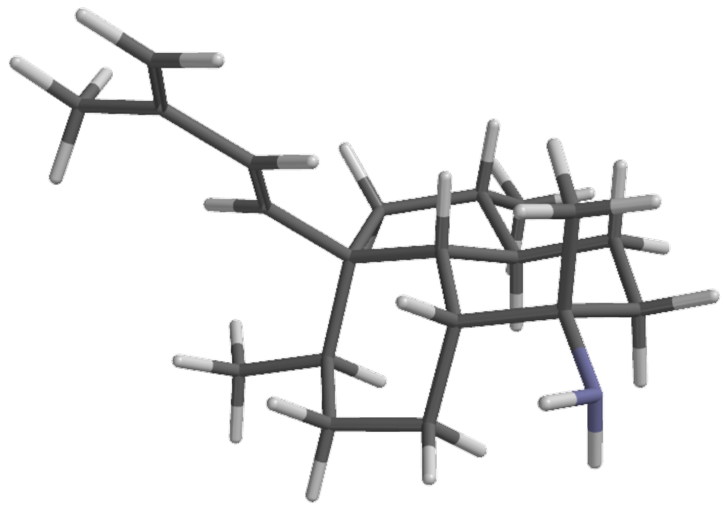

54

C	0.063722	-1.872377	-1.438021
C	-0.624434	-0.745645	-0.586718
C	0.318549	0.748509	1.399195
C	-1.895665	-0.436313	1.615717
C	-0.633012	0.011867	2.352036
C	-1.588624	-1.392638	0.459692
C	0.584922	-0.088720	0.133822
C	1.558099	-1.250114	0.362392
C	2.893205	-0.749474	0.900378
C	1.666537	1.218270	2.045107
C	2.664421	0.051248	2.187455
C	1.545800	-1.956704	-0.995998
C	-1.390816	0.184573	-1.509179
C	-1.234908	1.489729	-1.680967
C	-2.001535	2.331071	-2.624675
C	-1.748653	3.630812	-2.700083
C	-3.061811	1.688738	-3.484926
C	-2.889967	-1.945314	-0.134784
C	2.103097	-3.378145	-0.996147
H	-0.209303	1.653362	1.102715
H	1.064933	0.554228	-0.598453
H	1.143221	-1.932541	1.102810
C	2.284016	2.343880	1.200616
N	1.503778	1.789184	3.382807

H	-2.133385	-0.315995	-2.106936
H	-0.497130	2.023872	-1.109368
H	-0.008641	-1.667819	-2.500768
H	-0.430088	-2.825175	-1.274677
H	-2.424286	0.436308	1.237124
H	-2.569957	-0.926830	2.314131
H	-0.916123	0.665117	3.171171
H	-0.151808	-0.854845	2.800377
H	-1.056631	-2.240824	0.884654
H	3.387626	-0.134874	0.151172
H	3.564386	-1.578474	1.109607
H	3.599832	0.451331	2.564570
H	2.296781	-0.629840	2.953503
H	2.143885	-1.359380	-1.682412
H	-0.993697	4.096036	-2.090340
H	-2.284854	4.276581	-3.372444
H	-3.829289	1.219732	-2.875844
H	-2.636014	0.917398	-4.120191
H	-3.539419	2.423513	-4.121373
H	-3.566553	-1.149544	-0.428612
H	-2.715084	-2.572251	-1.003657
H	-3.405072	-2.551445	0.604492
H	2.032033	-3.826141	-1.983398
H	1.553720	-4.013014	-0.305483
H	3.148967	-3.395406	-0.704310
H	2.481648	2.048453	0.178054
H	3.216009	2.667413	1.649465
H	1.616819	3.201922	1.169404
H	0.855299	2.554545	3.369644
H	1.145453	1.112151	4.027654

Transition state energy barriers for tricycle formation:

The minimum energy transition states for **18** and **21** are reported. Geometries were optimized in the gas phase as described above using ωB97X-D/6-31G(d) and included full frequency analysis. Calculations were performed using Gaussian 16[10] and Spartan 18[9].

Understanding the transition state energy differences:

For alkylative cyclization of 18. *OR = either OMe or O⁻*

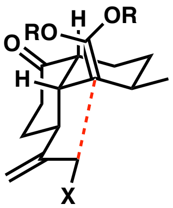

*approximation of favored TS [leads to **20** with desired quat. center configuration]*

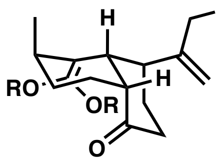

chair flip puts reactive functional groups distal

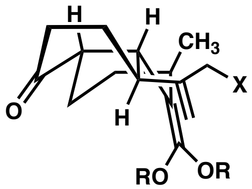

*the only alignment that could possibly lead to the undesired stereoisomer (**19**)requires both cyclohexanes in boat conformations*

For alkylative cyclization of 21. *OR = either OMe or O⁻*

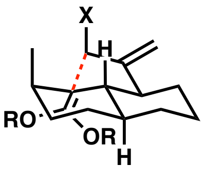

*approximation of favored TS [leads to **22** with undesired quat. center configuration]*

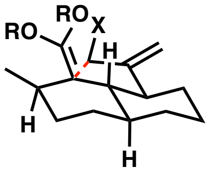

*the best alignment that could lead to the desired stereoisomer (**23**) requires the enolate-bearing cyclohexane in a boat conformation*

Calculated energy minimization of starting material **18**:

Sum of electronic and thermal Free Energies= -1308.263098

(Temperature 298.150 Kelvin. Pressure 1.00000 Atm.)

42	X	Y	Z
C	0.042219	1.25751	0.159719
C	-2.344658	0.399384	0.211859

C	-1.676034	2.221378	-1.392267
C	-2.553126	0.988648	-1.196768
C	-0.180178	1.913124	-1.188542
C	-0.848921	0.081258	0.483395
C	-0.418208	-1.243436	-0.233114
C	-1.340088	-2.395945	0.1763
C	-2.795602	-2.09869	-0.201268
C	-3.261597	-0.787229	0.408852
C	1.124793	1.551286	0.984737
O	1.043559	0.887974	2.240628
O	2.130738	2.262489	0.789902
C	2.262892	0.774543	2.918624
C	1.052165	-1.525546	0.020922
C	1.496179	-2.376415	0.945269
C	2.031432	-0.741766	-0.823173
Cl	3.196488	-1.839933	-1.738118
O	-4.320737	-0.709328	1.006236
C	0.624598	3.189871	-1.442608
H	-0.529182	-1.105048	-1.320567
H	-0.757438	-0.134854	1.551933
H	-2.65749	1.163351	0.933083
H	-1.97374	2.996189	-0.671061
H	-1.841221	2.631111	-2.400324
H	-3.61426	1.235506	-1.336113
H	-2.298245	0.242977	-1.964215
H	0.075541	1.215836	-2.02059
H	-1.286599	-2.539359	1.26409
H	-1.016214	-3.336218	-0.285951
H	-3.477993	-2.891349	0.120184
H	-2.87254	-2.012034	-1.295043
H	2.814592	1.71941	2.918363
H	2.02622	0.473297	3.945518
H	2.907637	0.001157	2.469144
H	2.560526	-2.537778	1.094494
H	0.824974	-2.939636	1.585546
H	2.658982	-0.069137	-0.238911
H	1.533779	-0.160344	-1.594382
H	0.37331	3.600584	-2.431624
H	1.696183	3.004308	-1.378376
H	0.389217	3.943952	-0.682931

Calculated energy minimization of starting material **21**:

Sum of electronic and thermal Free Energies= -1308.264982

(Temperature 298.150 Kelvin. Pressure 1.00000 Atm.)

42	X	Y	Z
C	1.198906	-0.073998	0.38417
C	-0.09258	1.892163	-0.507704
C	1.883487	2.225247	1.073275
C	0.540125	2.771835	0.574599
C	1.819166	0.739379	1.505264
C	-0.179287	0.41513	-0.000364
C	-0.953394	-0.488978	-0.974124
C	-2.333546	0.105578	-1.302364
C	-2.186985	1.50725	-1.914501
C	-1.440493	2.41624	-0.954394
O	-1.929225	3.459204	-0.555701
C	2.012829	-0.681731	-0.573906
O	3.341337	-0.880894	-0.120833
O	1.751487	-1.084789	-1.726727
C	4.177453	-1.506494	-1.053966
C	-1.057678	-1.915651	-0.465705
C	-0.952774	-2.950624	-1.30043
C	-1.209662	-2.189782	1.000162
Cl	-2.887735	-1.821122	1.65926
H	-0.803215	0.476605	0.911087
H	0.552012	1.859562	-1.400951
C	1.115735	0.577203	2.863942
H	-0.368272	-0.544119	-1.896766
H	2.249248	2.866113	1.892156
H	2.61756	2.294909	0.258913
H	-0.170214	2.843131	1.409706
H	0.658284	3.796201	0.200174
H	2.849361	0.401829	1.645117
H	-2.948961	0.168529	-0.39637
H	-2.864726	-0.551601	-2.001715
H	-3.156274	1.960083	-2.145181
H	-1.606231	1.428789	-2.844731

H	3.829133	-2.514032	-1.315933
H	4.258188	-0.937768	-1.989343
H	5.164546	-1.573982	-0.581207
H	-1.034302	-3.979237	-0.954794
H	-0.733709	-2.799898	-2.353163
H	-0.515796	-1.584268	1.582257
H	-1.05207	-3.245289	1.222509
H	0.057624	0.864743	2.828144
H	1.591823	1.193979	3.639781
H	1.160874	-0.469969	3.185245

Calculated transition state for **18** to **19**:

Sum of electronic and thermal Free Energies= -1308.237823

(Temperature 298.150 Kelvin. Pressure 1.00000 Atm.)

42	X	Y	Z
C	-0.097731	0.859335	0.382659
C	-2.074036	-0.746645	0.665384
C	-1.940228	1.34481	2.033877
C	-2.752609	0.591685	0.967689
C	-0.525707	1.776502	1.523697
C	-0.527278	-0.556438	0.648429
C	0.241018	-1.540829	-0.235494
C	-0.438065	-2.904267	-0.207622
C	-1.794531	-2.754165	-0.919528
C	-2.579021	-1.487493	-0.549545
C	-0.221846	1.36211	-0.949922
O	-0.071079	2.737121	-1.025395
O	-0.36879	0.73201	-2.002785
C	-0.094592	3.259091	-2.335041
C	1.669291	-1.39472	0.234855
C	2.351203	-2.37104	0.832447
C	2.174056	0.003094	0.066747
Cl	4.445362	-0.228026	-0.328935
O	-3.57117	-1.167473	-1.173229
C	0.471316	1.818912	2.686365
H	0.190581	-1.170262	-1.265112
H	-0.200757	-0.787949	1.673725

H	-2.29576	-1.443157	1.494163
H	-1.83112	0.689488	2.912699
H	-2.493362	2.226648	2.382881
H	-3.784435	0.429923	1.300694
H	-2.809149	1.183016	0.047004
H	-0.614973	2.792476	1.131043
H	-0.57679	-3.244754	0.829026
H	0.164176	-3.66741	-0.714763
H	-2.452267	-3.612934	-0.729841
H	-1.646137	-2.711002	-2.00445
H	-1.041643	3.041165	-2.840765
H	0.716258	2.848807	-2.946275
H	0.031446	4.340121	-2.228033
H	3.375675	-2.224164	1.155571
H	1.911112	-3.357309	0.96069
H	2.444657	0.608525	0.914028
H	2.074658	0.481458	-0.892894
H	0.683399	0.808581	3.060494
H	0.083721	2.410717	3.527542
H	1.420417	2.261526	2.365772

<u>Calculated transition state for **18** to **20**:</u>

Sum of electronic and thermal Free Energies= -1308.252938

(Temperature 298.150 Kelvin. Pressure 1.00000 Atm.)

42	X	Y	Z
C	-0.261265	-0.9606	-0.153098
C	2.243381	-0.441742	-0.000347
C	1.339631	-1.898195	-1.859295
C	2.388687	-0.854085	-1.478564
C	-0.081235	-1.359228	-1.611592
C	0.798203	-0.006767	0.359116
C	0.467335	1.434964	-0.086872
C	1.492378	2.425059	0.458924
C	2.90013	2.055809	-0.029878
C	3.268251	0.624768	0.33565
C	-0.937753	-1.809712	0.764536

O	-0.752492	-1.42027	2.093367
O	-1.692357	-2.760343	0.54228
C	-1.514407	-2.148391	3.024795
C	-0.969385	1.648843	0.339793
C	-1.334271	2.448706	1.341577
C	-1.944289	0.782494	-0.375358
Cl	-3.54696	2.162748	-1.037426
O	4.338699	0.360762	0.852119
C	-1.13456	-2.30065	-2.198644
H	0.498312	1.494901	-1.186789
H	0.740857	0.013148	1.451107
H	2.493356	-1.320049	0.606864
H	1.48776	-2.81277	-1.268052
H	1.464158	-2.169669	-2.9181
H	3.405258	-1.231944	-1.64759
H	2.263582	0.022799	-2.131021
H	-0.13339	-0.434874	-2.219179
H	1.481762	2.397493	1.556949
H	1.247749	3.451367	0.160843
H	3.668419	2.719616	0.377225
H	2.930916	2.141596	-1.126106
H	-1.271568	-3.21709	3.004939
H	-1.269145	-1.730042	4.005571
H	-2.589677	-2.04742	2.838204
H	-2.379298	2.554912	1.613634
H	-0.614574	3.049217	1.889872
H	-2.643546	0.178084	0.18085
H	-1.749328	0.491122	-1.392892
H	-0.974503	-2.401155	-3.281178
H	-2.144757	-1.91703	-2.029813
H	-1.08981	-3.287754	-1.735687

Calculated transition state for **21** to **22**:

Sum of electronic and thermal Free Energies= -1308.248343

(Temperature 298.150 Kelvin. Pressure 1.00000 Atm.)

42	X	Y	Z
C	0.098255	0.904505	0.371004
C	-2.130016	-0.022524	-0.300568
C	-1.941419	2.111507	1.112679
C	-2.802967	0.89153	0.732172
C	-0.469168	1.778271	1.485217
C	-0.711785	-0.358445	0.21487
C	0.050282	-1.455045	-0.533228
C	-0.824471	-2.695819	-0.695482
C	-2.145252	-2.326805	-1.397157
C	-2.90888	-1.2877	-0.587668
C	0.443049	1.580699	-0.838624
O	0.835052	2.895508	-0.640655
O	0.48774	1.122537	-1.987257
C	1.306075	3.549214	-1.797443
C	1.326294	-1.626291	0.273015
C	1.623377	-2.730719	0.958492
C	2.170624	-0.396721	0.330386
Cl	4.315812	-1.142884	0.194354
O	-4.037282	-1.496341	-0.180149
C	-0.349849	1.172785	2.889421
H	0.316012	-1.072368	-1.525373
H	-0.859777	-0.780632	1.221568
H	-2.000168	0.495479	-1.263945
H	-2.437373	2.653316	1.932755
H	-1.909676	2.798189	0.256022
H	-3.013809	0.288346	1.625604
H	-3.779573	1.22632	0.363098
H	0.078055	2.724963	1.503109
H	-1.060919	-3.123671	0.288842
H	-0.302455	-3.473587	-1.265357
H	-2.790261	-3.199487	-1.536297
H	-1.915553	-1.900392	-2.383607
H	2.180005	3.04174	-2.219663
H	1.580765	4.558944	-1.479637
H	0.536824	3.599742	-2.576044
H	2.549832	-2.803352	1.517746
H	0.983501	-3.608046	0.933143
H	2.417196	0.064525	1.271122
H	2.315923	0.171088	-0.572432

H	-0.945983	0.261604	3.012181
H	0.692364	0.914359	3.106176
H	-0.692716	1.887669	3.648955

Calculated transition state for **21** to **23**:

Sum of electronic and thermal Free Energies= -1308.237141

(Temperature 298.150 Kelvin. Pressure 1.00000 Atm.)

42	X	Y	Z
C	-0.027732	1.070779	-0.064683
C	-2.134269	-0.358584	-0.662515
C	-1.722345	1.706756	-1.874844
C	-2.764674	0.971694	-1.019713
C	-0.478728	2.141312	-1.06574
C	-0.832618	-0.195459	0.152572
C	-0.027946	-1.500266	-0.092326
C	-0.694004	-2.600274	0.728785
C	-2.192143	-2.707717	0.348213
C	-2.933188	-1.38038	0.114374
C	0.782079	1.513707	1.020501
O	0.689586	0.705468	2.146277
O	1.566708	2.468921	1.038382
C	1.69448	0.922803	3.108293
C	1.444203	-1.226126	0.059357
C	2.249432	-1.828873	0.931622
C	1.887766	-0.159098	-0.875226
Cl	3.557642	-1.107587	-2.028816
O	-4.066857	-1.20502	0.515796
C	-0.79796	3.493421	-0.398265
H	-0.144443	-1.771455	-1.155499
H	-1.127101	-0.219459	1.213325
H	-1.874829	-0.844246	-1.617039
H	-2.16348	2.594484	-2.345515
H	-1.427237	1.037403	-2.696536
H	-3.010936	1.542138	-0.116238
H	-3.699572	0.82239	-1.572712
H	0.328489	2.340105	-1.789656
H	-0.601402	-2.353207	1.793951
H	-0.212135	-3.573302	0.575688

H	-2.759261	-3.28411	1.085069
H	-2.264705	-3.24756	-0.608759
H	1.50754	0.200248	3.907916
H	1.659656	1.942036	3.507347
H	2.691806	0.753417	2.687131
H	3.31407	-1.618635	0.949857
H	1.870622	-2.571059	1.629127
H	2.522912	0.648605	-0.542754
H	1.327993	0.004737	-1.781886
H	-1.653048	3.398095	0.283574
H	-1.053084	4.24416	-1.158974
H	0.05863	3.847077	0.176378

D. X-Ray Crystal Structure of Tosylate 57

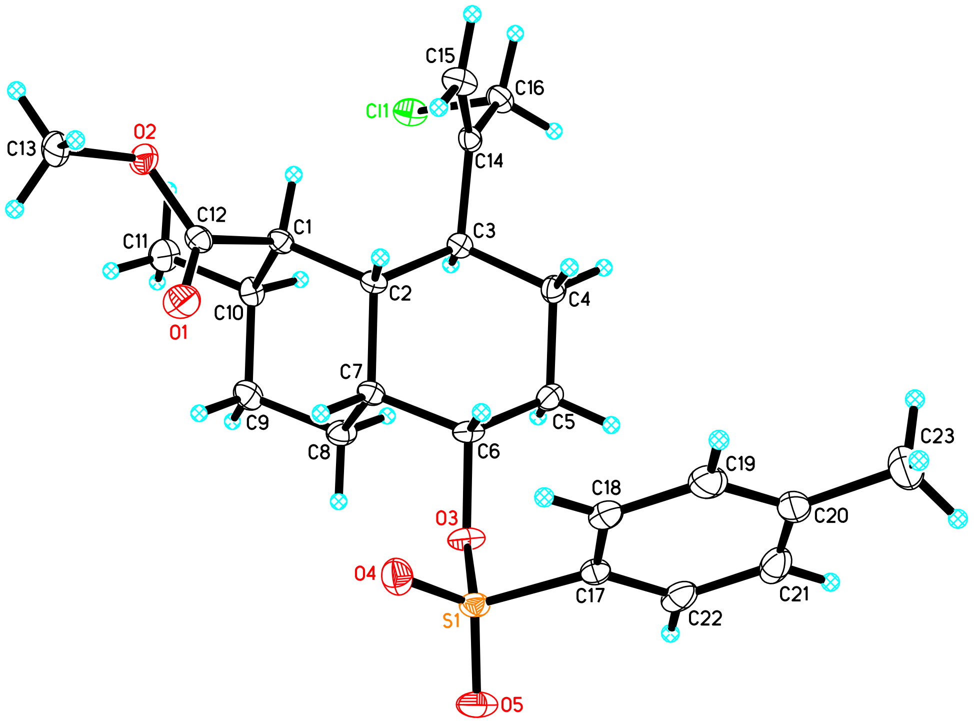

Courtesy of Joe Ziller, Ph.D.
Director, X-Ray Crystallography Facility

X-ray Data Collection, Structure Solution and Refinement for Tosylate 5.29 (cdv96)

The data for cdv96 were collected from a shock-cooled single crystal at 93(2) K on a Bruker X8 Prospector APEX II CCD ϰ-geometry diffractometer with an IuS microsource using a multi-layer optics as monochromator and a Bruker Apex II CCD detector. The diffractometer was equipped with a Cryo Industries low temperature device and used CuK_α radiation (λ = 1.54178 Å). All data were integrated with SAINT and a multi-scan absorption correction using SADABS was applied.[1,2] The structure was solved by direct methods using SHELXT and refined by full-matrix least-squares methods against F^2 by SHELXL-2018/3 using ShelXle.[3-5] All non-hydrogen atoms were refined with unrestrained anisotropic displacement parameters. All hydrogen atoms were refined isotropic on calculated positions using a riding model with their U_{iso} values constrained to 1.5 times the U_{eq} of their pivot atoms for terminal sp^3 carbon atoms and 1.2 times for all other carbon atoms. This report and the CIF file were generated using FinalCif.[6]

References

[1] Bruker, *SAINT*, *V8.38A*, Bruker AXS Inc., Madison, Wisconsin, USA.

[2] L. Krause, R. Herbst-Irmer, G. M. Sheldrick, D. Stalke, *J. Appl. Cryst.* **2015**, *48*, 3–10, doi:10.1107/S1600576714022985.

[3] G. M. Sheldrick, *Acta Cryst.* **2015**, *A71*, 3–8, doi:10.1107/S2053273314026370.

[4] G. M. Sheldrick, *Acta Cryst.* **2015**, *C71*, 3–8, doi:10.1107/S2053229614024218.

[5] C. B. Hübschle, G. M. Sheldrick, B. Dittrich, *J. Appl. Cryst.* **2011**, *44*, 1281–1284, doi:10.1107/S0021889811043202.

[6] D. Kratzert, *FinalCif*, *V108*, https://dkratzert.de/finalcif.html.

Table 1. Crystal data and Structure Refinement for cdv 96.

Identification code	cdv96 (Natalie Dwulet)
Empirical formula	$C_{23}H_{31}ClO_5S$
Formula weight	454.99
Temperature [K]	93(2)
Crystal system	orthorhombic
Space group (number)	$P2_12_12_1$ (19)
a [Å]	9.2068(3)
b [Å]	14.1404(4)
c [Å]	17.6806(5)
α [°]	90
β [°]	90
γ [°]	90
Volume [Å^3]	2301.80(12)
Z	4
ϱ_{calc} [gcm^{-3}]	1.313
μ [mm^{-1}]	2.575
F(000)	968
Crystal size [mm^3]	0.233×0.190×0.054
Crystal colour	colorless
Crystal shape	irregular
Radiation	CuK_α (λ=1.54178 Å)
2θ range [°]	8.01 to 137.01 (0.83 Å)
Index ranges	$-11 \leq h \leq 11$<br>$-16 \leq k \leq 10$<br>$-21 \leq l \leq 21$
Reflections collected	24769
Independent reflections	4200<br>$R_{int} = 0.0356$<br>$R_{sigma} = 0.0279$
Completeness to θ = 67.679°	100.0 %
Data / Restraints / Parameters	4200/0/274
Goodness-of-fit on F^2	1.026
Final *R* indexes [$I \geq 2\sigma(I)$]	$R_1 = 0.0249$<br>$wR_2 = 0.0655$
Final *R* indexes [all data]	$R_1 = 0.0256$<br>$wR_2 = 0.0662$
Largest peak/hole [eÅ^{-3}]	0.29/-0.33
Flack X parameter	0.007(5)

Refinement details for cdv96

All non-hydrogen atoms were refined anisotropically using no restraints or constraints. All hydrogen atoms were refined using a fixed model.

Table 2. Atomic coordinates and U_{eq} [Å^2] for cdv96

Atom	*x*	*y*	*z*	U_{eq}
Cl1	0.48890(5)	0.70170(3)	0.20349(3)	0.01977(12)
S1	1.00878(5)	0.19097(3)	0.19715(2)	0.01507(12)
O1	0.48107(17)	0.29841(9)	0.05854(9)	0.0244(3)
O2	0.30126(16)	0.40472(9)	0.04931(8)	0.0191(3)
O3	0.98034(15)	0.29958(9)	0.18697(8)	0.0176(3)
O4	0.89197(17)	0.13783(9)	0.16508(8)	0.0211(3)
O5	1.15305(16)	0.17720(10)	0.16977(9)	0.0240(3)
C1	0.5151(2)	0.45615(12)	0.11025(10)	0.0134(4)
H1A	0.439970	0.498666	0.132893	0.016
C2	0.6076(2)	0.41697(12)	0.17638(10)	0.0119(4)
H2A	0.549688	0.365377	0.200635	0.014
C3	0.6344(2)	0.49314(12)	0.23775(10)	0.0129(4)
H3A	0.686070	0.547504	0.213556	0.015
C4	0.7316(2)	0.45442(13)	0.30172(11)	0.0148(4)
H4A	0.751841	0.505656	0.338402	0.018
H4B	0.679225	0.403481	0.328762	0.018
C5	0.8752(2)	0.41563(14)	0.27145(11)	0.0165(4)
H5A	0.931150	0.466683	0.246563	0.020
H5B	0.934212	0.389842	0.313461	0.020
C6	0.8404(2)	0.33830(13)	0.21506(11)	0.0143(4)
H6A	0.786121	0.286828	0.241619	0.017
C7	0.7510(2)	0.37157(13)	0.14757(11)	0.0135(4)
H7A	0.723815	0.314067	0.117838	0.016
C8	0.8356(2)	0.43708(14)	0.09436(11)	0.0174(4)
H8A	0.866103	0.494580	0.122085	0.021
H8B	0.924050	0.404420	0.076091	0.021
C9	0.7408(2)	0.46483(14)	0.02717(11)	0.0185(4)
H9A	0.712192	0.407337	-0.001102	0.022

H9B	0.796855	0.506056	-0.007422	0.022
C10	0.6050(2)	0.51688(13)	0.05386(11)	0.0155(4)
H10A	0.639133	0.573678	0.082478	0.019
C11	0.5155(3)	0.55364(14)	-0.01281(11)	0.0223(4)
H11A	0.573656	0.598690	-0.042077	0.033
H11B	0.428068	0.585253	0.006191	0.033
H11C	0.487351	0.500532	-0.045275	0.033
C12	0.4347(2)	0.37686(14)	0.07029(11)	0.0152(4)
C13	0.2167(2)	0.33434(15)	0.00898(12)	0.0227(4)
H13A	0.124852	0.362436	-0.007753	0.034
H13B	0.196600	0.280796	0.042582	0.034
H13C	0.271512	0.312249	-0.035124	0.034
C14	0.4945(2)	0.53044(12)	0.27313(9)	0.0140(4)
C15	0.3826(2)	0.47675(14)	0.29261(12)	0.0196(4)
H15A	0.301916	0.503892	0.318102	0.023
H15B	0.383112	0.411136	0.281084	0.023
C16	0.4925(2)	0.63395(13)	0.29165(10)	0.0178(4)
H16A	0.579886	0.650794	0.321341	0.021
H16B	0.405601	0.649019	0.322377	0.021
C17	1.0041(2)	0.17583(12)	0.29617(10)	0.0155(4)
C18	0.8905(2)	0.12676(14)	0.32913(12)	0.0184(4)
H18A	0.817828	0.098151	0.298623	0.022
C19	0.8842(3)	0.11987(16)	0.40754(12)	0.0249(5)
H19A	0.807332	0.085570	0.430594	0.030
C20	0.9893(3)	0.16271(15)	0.45259(11)	0.0278(5)
C21	1.1036(3)	0.21001(15)	0.41800(13)	0.0270(5)
H21A	1.177129	0.237943	0.448387	0.032
C22	1.1124(2)	0.21718(13)	0.34021(13)	0.0211(4)
H22A	1.191024	0.249752	0.317103	0.025
C23	0.9794(4)	0.1574(2)	0.53778(14)	0.0501(8)
H23A	1.077318	0.159027	0.559483	0.075
H23B	0.931204	0.098363	0.552431	0.075
H23C	0.923236	0.211292	0.556697	0.075

U_{eq} is defined as 1/3 of the trace of the orthogonalized U_{ij} tensor.

Table 3. Anisotropic displacement parameters [Å^2] for cdv96. The anisotropic displacement factor exponent takes the form: $-2\pi^2[\, h^2(a^*)^2U_{11} + k^2(b^*)^2U_{22} + \ldots + 2hka^*b^*U_{12}\,]$

Atom	U_{11}	U_{22}	U_{33}	U_{23}	U_{13}	U_{12}
Cl1	0.0219(2)	0.0114(2)	0.0259(2)	0.00184(16)	0.0037(2)	0.00298(18)
S1	0.0158(2)	0.0141(2)	0.0153(2)	0.00215(15)	0.00192(18)	0.00490(19)
O1	0.0233(8)	0.0154(6)	0.0346(8)	−0.0087(6)	−0.0042(7)	0.0028(6)
O2	0.0166(7)	0.0187(6)	0.0219(7)	−0.0061(6)	−0.0034(6)	−0.0004(6)
O3	0.0133(7)	0.0153(6)	0.0243(7)	0.0049(5)	0.0059(5)	0.0041(6)
O4	0.0267(8)	0.0194(7)	0.0171(7)	−0.0002(6)	−0.0020(6)	0.0016(6)
O5	0.0211(8)	0.0251(7)	0.0259(8)	0.0054(6)	0.0083(6)	0.0097(6)
C1	0.0158(9)	0.0111(7)	0.0131(8)	−0.0002(6)	0.0016(8)	0.0017(7)
C2	0.0126(9)	0.0098(8)	0.0134(8)	0.0005(7)	0.0020(7)	0.0005(7)
C3	0.0143(9)	0.0110(8)	0.0132(9)	0.0008(7)	0.0010(7)	−0.0001(7)
C4	0.0169(10)	0.0146(9)	0.0130(9)	0.0009(7)	−0.0014(8)	0.0012(7)
C5	0.0142(10)	0.0180(9)	0.0174(9)	0.0015(7)	−0.0023(8)	0.0017(7)
C6	0.0115(9)	0.0131(8)	0.0182(9)	0.0027(7)	0.0038(7)	0.0028(7)
C7	0.0149(10)	0.0115(8)	0.0142(8)	−0.0002(7)	0.0022(7)	0.0011(7)
C8	0.0152(9)	0.0192(9)	0.0177(9)	0.0032(8)	0.0031(8)	0.0015(8)
C9	0.0227(11)	0.0182(9)	0.0148(9)	0.0027(8)	0.0036(8)	0.0000(8)
C10	0.0198(10)	0.0132(8)	0.0137(9)	0.0015(7)	−0.0005(8)	−0.0009(8)
C11	0.0253(11)	0.0237(9)	0.0179(9)	0.0081(7)	0.0004(9)	0.0018(9)
C12	0.0158(10)	0.0166(9)	0.0131(9)	0.0001(7)	0.0026(7)	−0.0006(8)
C13	0.0201(11)	0.0274(10)	0.0206(10)	−0.0084(8)	−0.0018(9)	−0.0062(9)
C14	0.0166(9)	0.0144(8)	0.0109(8)	0.0002(6)	−0.0008(8)	0.0033(8)
C15	0.0199(10)	0.0176(9)	0.0211(10)	0.0007(8)	0.0053(9)	0.0021(8)
C16	0.0201(10)	0.0174(9)	0.0157(8)	−0.0027(7)	0.0016(9)	0.0013(8)
C17	0.0166(9)	0.0133(8)	0.0165(8)	0.0000(6)	−0.0005(8)	0.0053(8)
C18	0.0156(10)	0.0189(9)	0.0208(10)	0.0008(8)	−0.0008(8)	0.0047(8)
C19	0.0243(11)	0.0282(10)	0.0222(11)	0.0059(9)	0.0036(9)	0.0094(9)
C20	0.0340(13)	0.0308(10)	0.0186(10)	−0.0022(8)	−0.0018(10)	0.0209(11)
C21	0.0316(12)	0.0226(10)	0.0268(11)	−0.0086(9)	−0.0133(10)	0.0118(10)
C22	0.0189(10)	0.0154(9)	0.0291(11)	−0.0016(8)	−0.0051(9)	0.0042(8)
C23	0.062(2)	0.0690(19)	0.0191(11)	−0.0027(11)	−0.0025(13)	0.0389(17)

Table 4. Bond lengths and angles for cdv96

Atom–Atom	Length [Å]
Cl1–C16	1.8299(19)
S1–O5	1.4270(15)
S1–O4	1.4292(16)
S1–O3	1.5683(13)
S1–C17	1.7642(18)
O1–C12	1.207(3)
O2–C12	1.342(3)
O2–C13	1.451(2)
O3–C6	1.485(2)
C1–C12	1.518(3)
C1–C2	1.549(3)
C1–C10	1.555(3)
C2–C3	1.549(2)
C2–C7	1.554(3)
C3–C14	1.526(3)
C3–C4	1.543(3)
C4–C5	1.528(3)
C5–C6	1.514(3)
C6–C7	1.524(3)
C7–C8	1.533(3)
C8–C9	1.526(3)
C9–C10	1.526(3)
C10–C11	1.529(3)
C14–C15	1.325(3)
C14–C16	1.500(2)
C17–C18	1.383(3)
C17–C22	1.394(3)
C18–C19	1.391(3)
C19–C20	1.392(3)
C20–C21	1.389(4)
C20–C23	1.511(3)
C21–C22	1.382(3)

**Atom–Atom–Atom**	**Angle [°]**
O5–S1–O4	119.60(9)
O5–S1–O3	104.48(8)
O4–S1–O3	110.10(8)
O5–S1–C17	110.06(9)
O4–S1–C17	108.14(9)
O3–S1–C17	103.22(8)
C12–O2–C13	115.19(16)
C6–O3–S1	117.86(11)
C12–C1–C2	110.80(14)
C12–C1–C10	111.66(15)
C2–C1–C10	112.90(16)
C1–C2–C3	111.59(14)
C1–C2–C7	111.56(15)
C3–C2–C7	112.40(15)
C14–C3–C4	108.16(15)
C14–C3–C2	113.13(16)
C4–C3–C2	111.08(14)
C5–C4–C3	111.86(15)
C6–C5–C4	107.88(16)
O3–C6–C5	107.63(15)
O3–C6–C7	108.70(15)
C5–C6–C7	114.01(15)
C6–C7–C8	113.09(16)
C6–C7–C2	109.27(15)
C8–C7–C2	112.55(15)
C9–C8–C7	110.01(16)
C8–C9–C10	110.62(16)
C9–C10–C11	111.54(16)
C9–C10–C1	111.61(15)
C11–C10–C1	113.28(17)
O1–C12–O2	123.10(18)

O1–C12–C1	125.91(19)
O2–C12–C1	110.99(16)
C15–C14–C16	119.49(19)
C15–C14–C3	124.38(17)
C16–C14–C3	115.95(17)
C14–C16–Cl1	108.97(12)
C18–C17–C22	121.05(18)
C18–C17–S1	119.84(15)
C22–C17–S1	119.06(16)
C17–C18–C19	119.1(2)
C18–C19–C20	120.7(2)
C21–C20–C19	118.96(19)
C21–C20–C23	120.6(3)
C19–C20–C23	120.4(3)
C22–C21–C20	121.2(2)
C21–C22–C17	118.9(2)

E. NMR Spectra

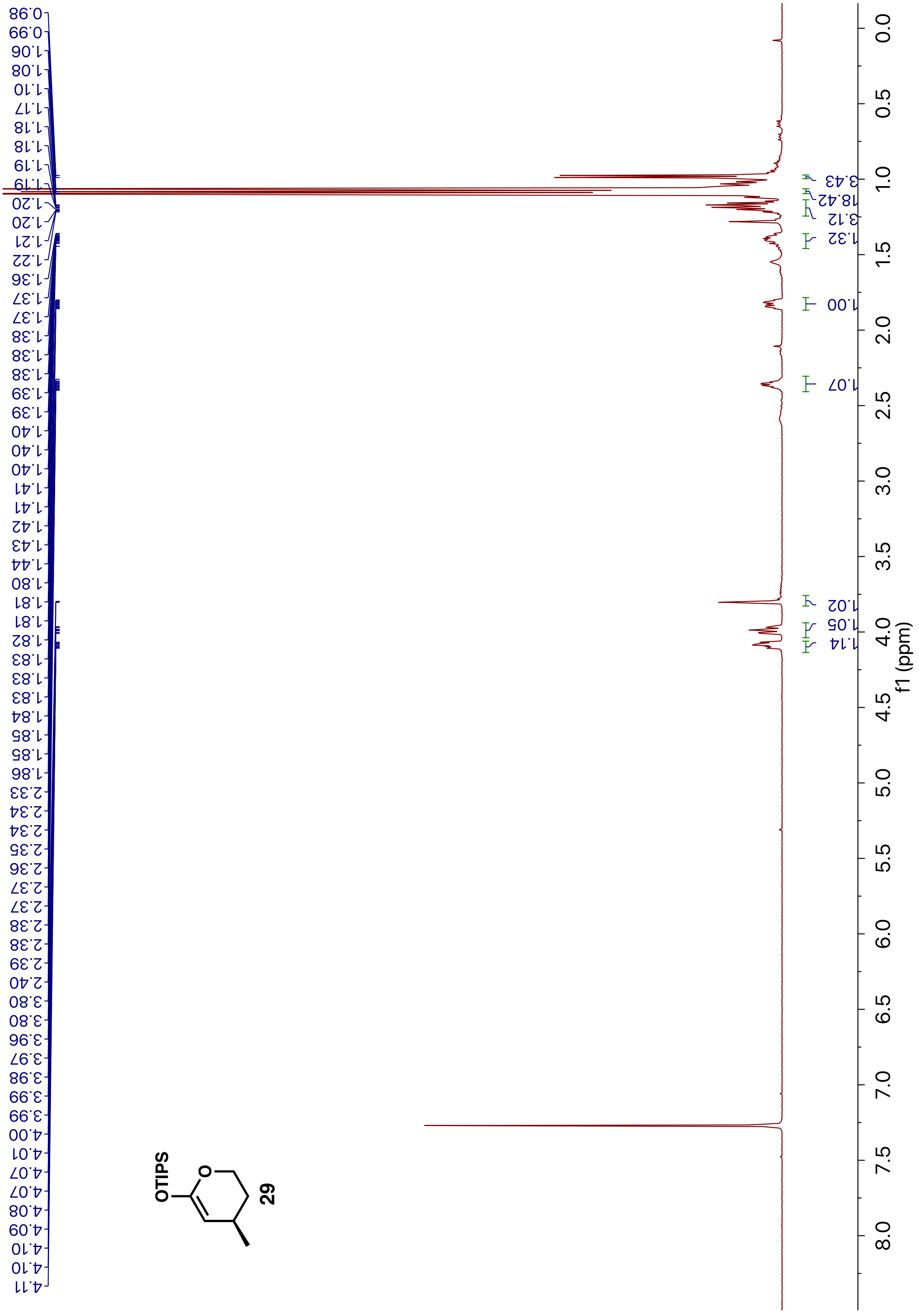

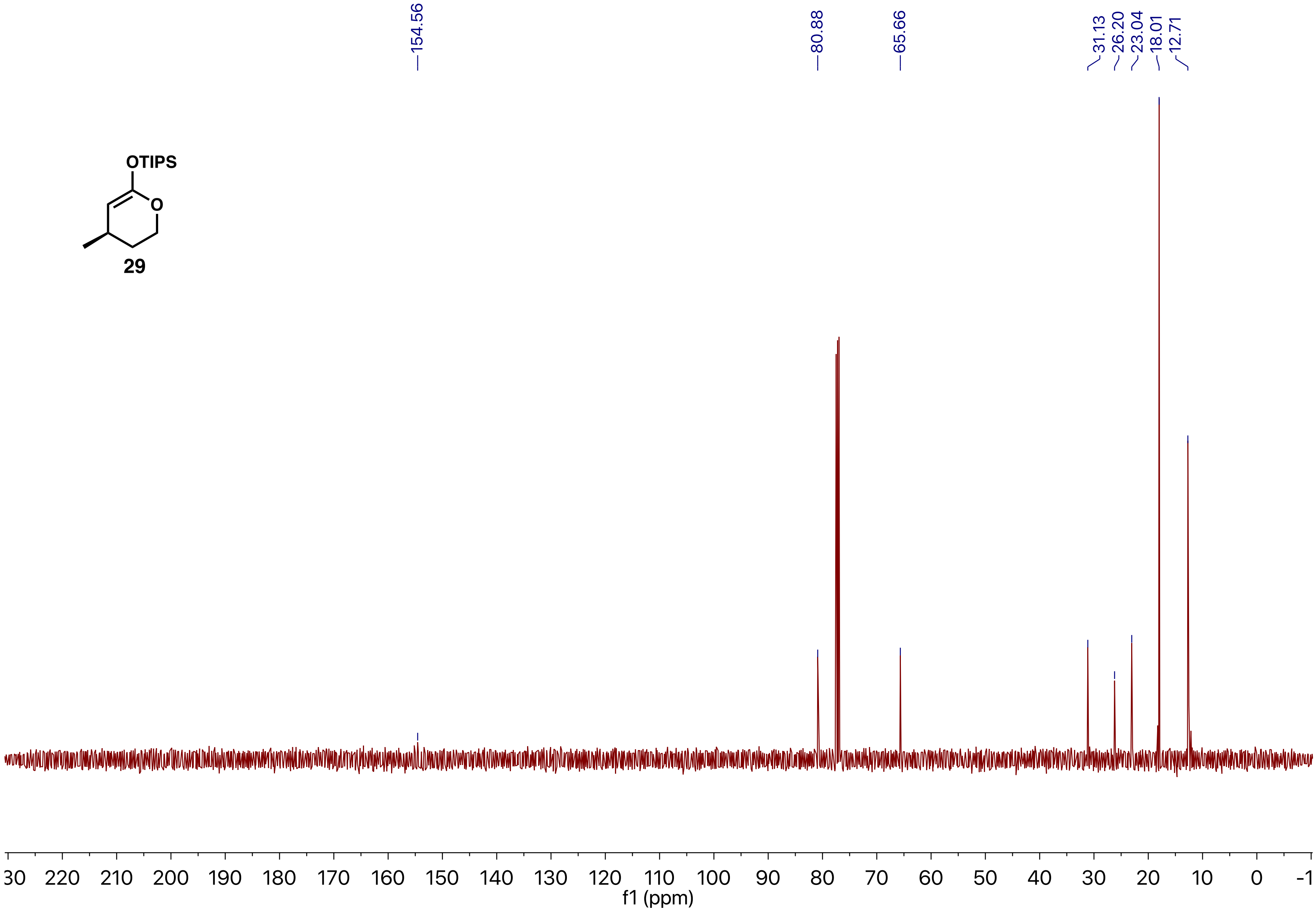
OTIPS
O
29
154.56
80.88
65.66
31.13
26.20
23.04
18.01
12.71
30 220 210 200 190 180 170 160 150 140 130 120 110 100 90 80 70 60 50 40 30 20 10 0 -1
f1 (ppm)

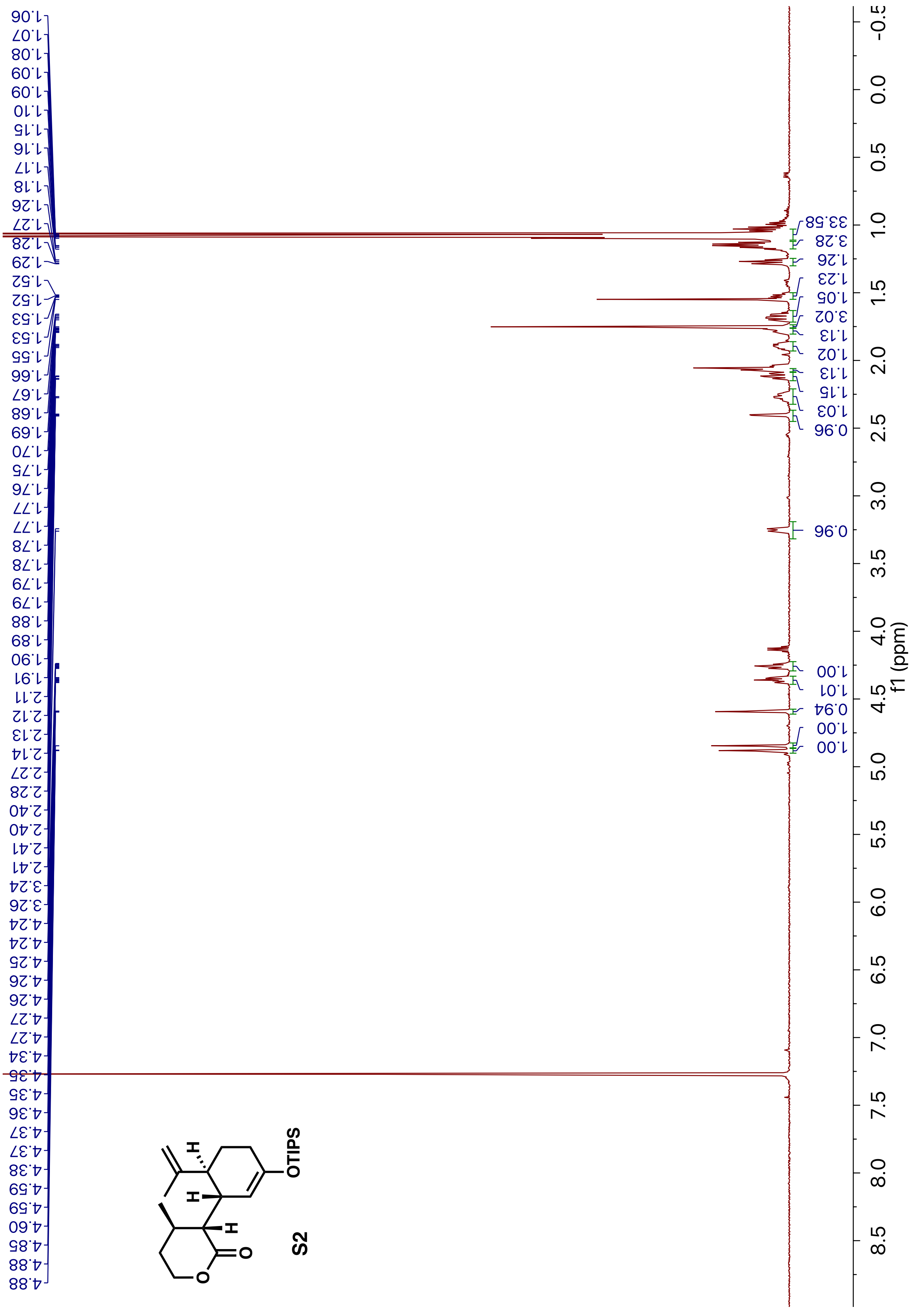

1.06
1.07
1.08
1.09
1.09
1.10
1.15
1.16
1.17
1.18
1.26
1.27
1.28
1.29
1.52
1.52
1.53
1.53
1.55
1.66
1.67
1.68
1.69
1.70
1.75
1.76
1.77
1.77
1.78
1.78
1.79
1.79
1.88
1.89
1.90
1.91
2.11
2.12
2.13
2.14
2.27
2.28
2.40
2.40
2.41
2.41
3.24
3.26
4.24
4.24
4.25
4.26
4.26
4.27
4.27
4.34
4.35
4.35
4.36
4.37
4.38
4.59
4.59
4.60
4.85
4.88
4.88
33.58
3.28
1.26
1.23
1.05
3.02
1.13
1.02
1.13
1.15
1.03
0.96
0.96
1.00
1.01
0.94
1.00
1.00
H
H
H
O
O
OTIPS
S2
-0.5
0.0
0.5
1.0
1.5
2.0
2.5
3.0
3.5
4.0
4.5
5.0
5.5
6.0
6.5
7.0
7.5
8.0
8.5
f1 (ppm)

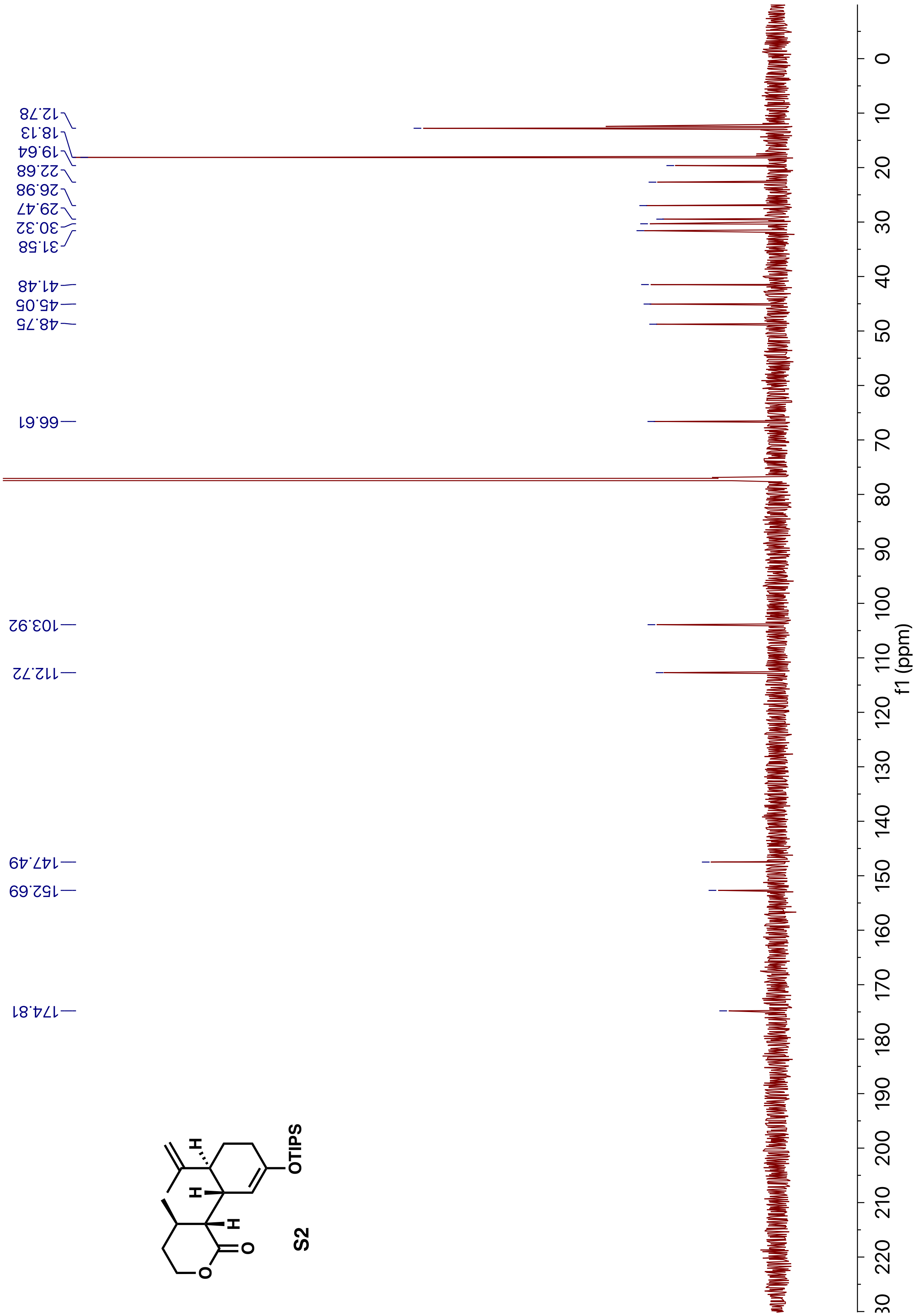
12.78
18.13
19.64
22.68
26.98
29.47
30.32
31.58
41.48
45.05
48.75
66.61
103.92
112.72
147.49
152.69
174.81
OTIPS
S2
f1 (ppm)

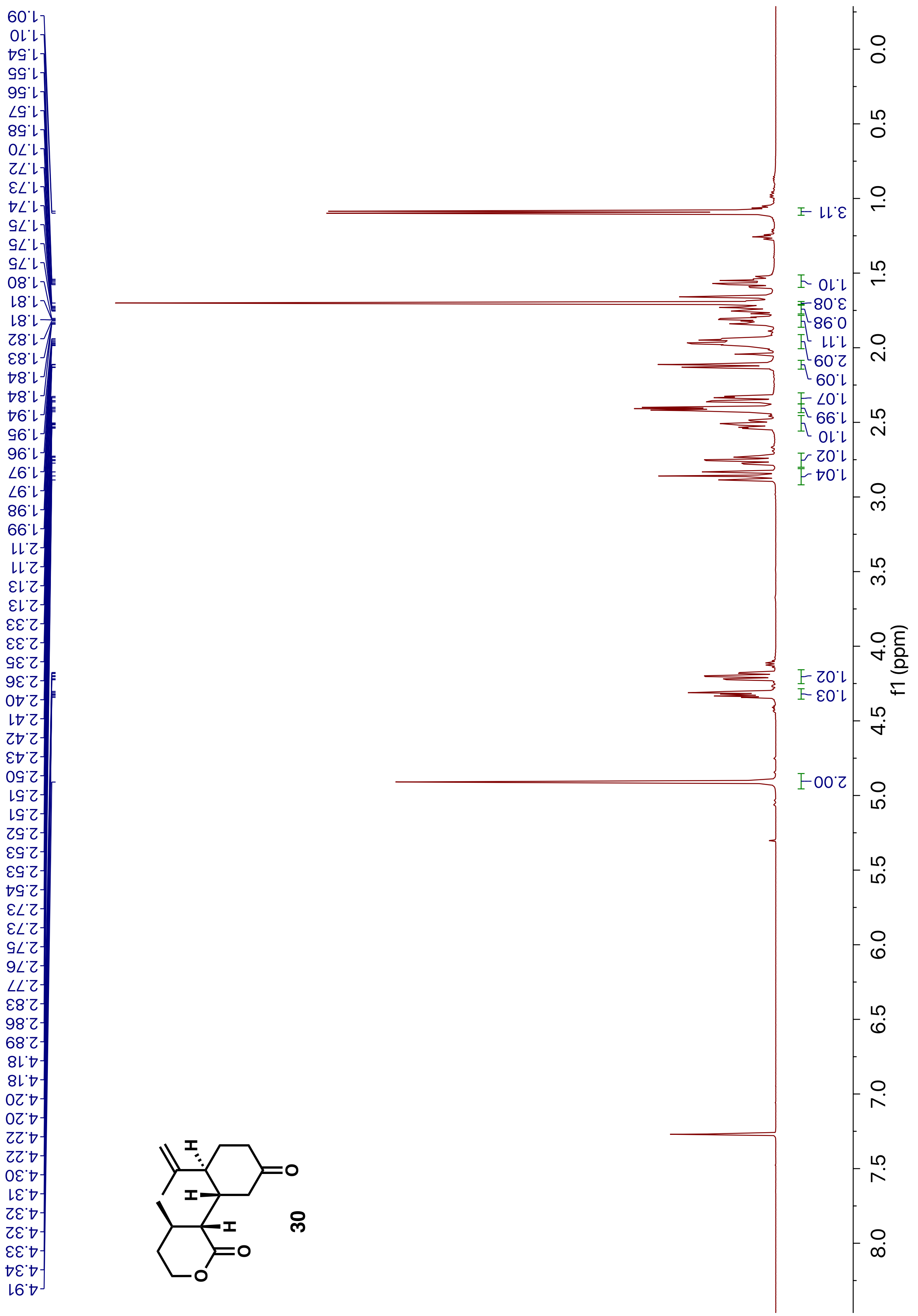
1.09
1.10
1.54
1.55
1.56
1.57
1.58
1.70
1.72
1.73
1.74
1.75
1.75
1.75
1.80
1.81
1.81
1.82
1.83
1.84
1.84
1.94
1.95
1.96
1.97
1.97
1.98
1.99
2.11
2.11
2.13
2.13
2.33
2.33
2.35
2.36
2.40
2.41
2.42
2.43
2.50
2.51
2.51
2.52
2.53
2.53
2.54
2.73
2.73
2.75
2.76
2.77
2.83
2.86
2.89
4.18
4.18
4.20
4.20
4.22
4.22
4.30
4.31
4.32
4.32
4.33
4.34
4.91
3.11
1.10
3.08
0.98
1.11
2.09
1.09
1.07
1.99
1.10
1.02
1.04
1.02
1.03
2.00
0.0
0.5
1.0
1.5
2.0
2.5
3.0
3.5
4.0
4.5
5.0
5.5
6.0
6.5
7.0
7.5
8.0
f1 (ppm)
H
H
H
O
O
O
30

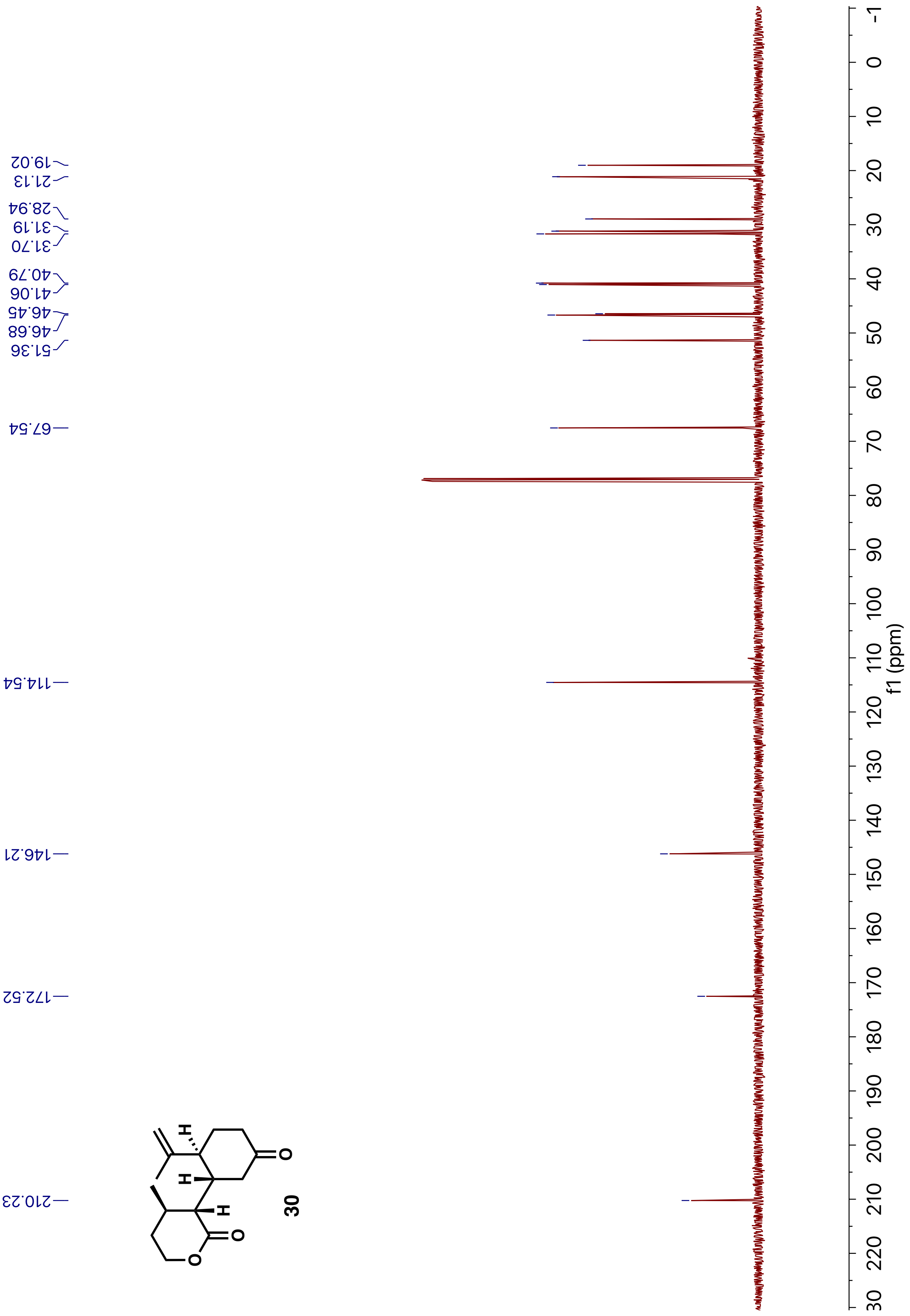
210.23
172.52
146.21
114.54
67.54
51.36
46.68
46.45
41.06
40.79
31.70
31.19
28.94
21.13
19.02
30
f1 (ppm)

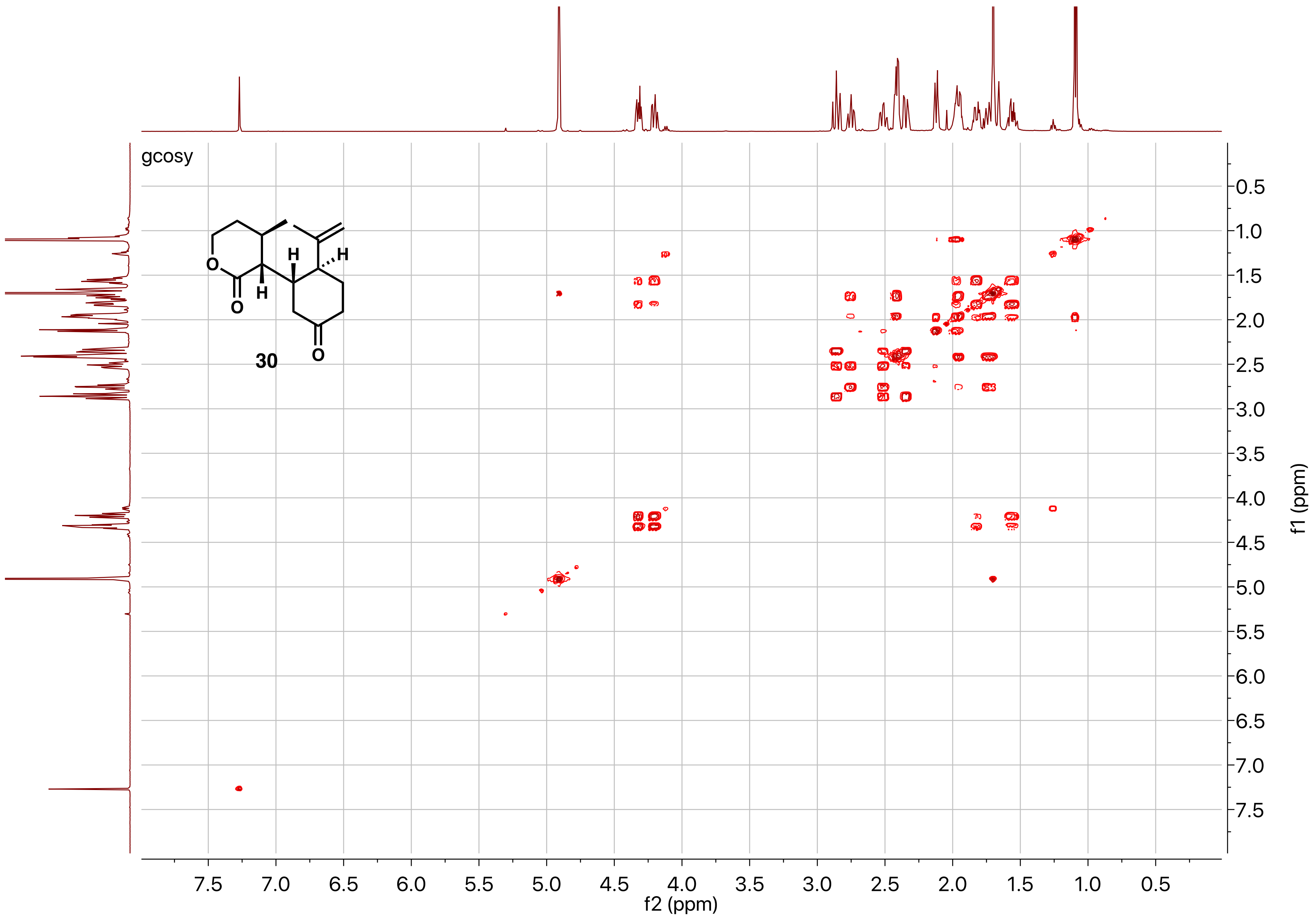
gcosy
30
f2 (ppm)
f1 (ppm)

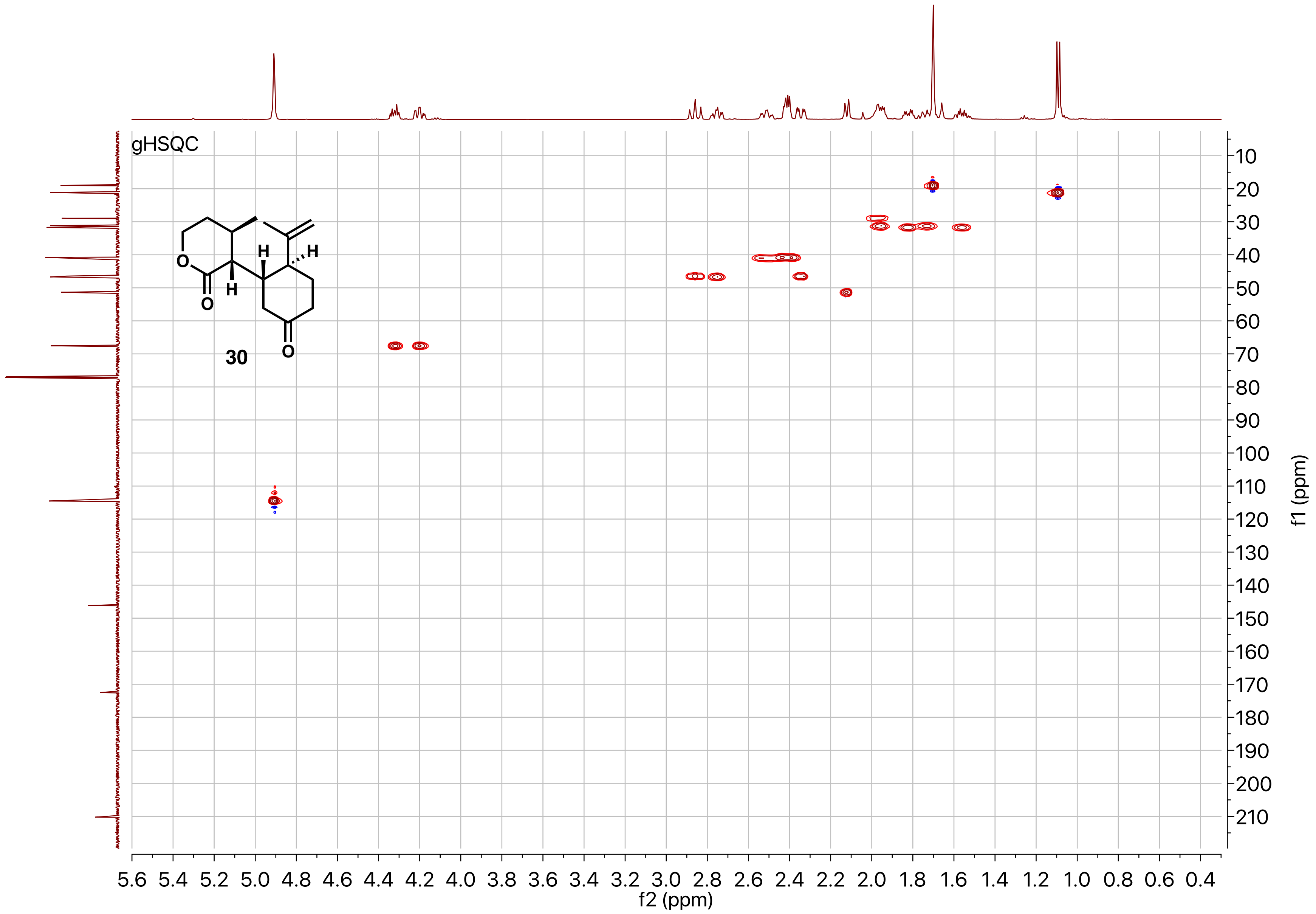
gHSQC
30
f2 (ppm)
f1 (ppm)

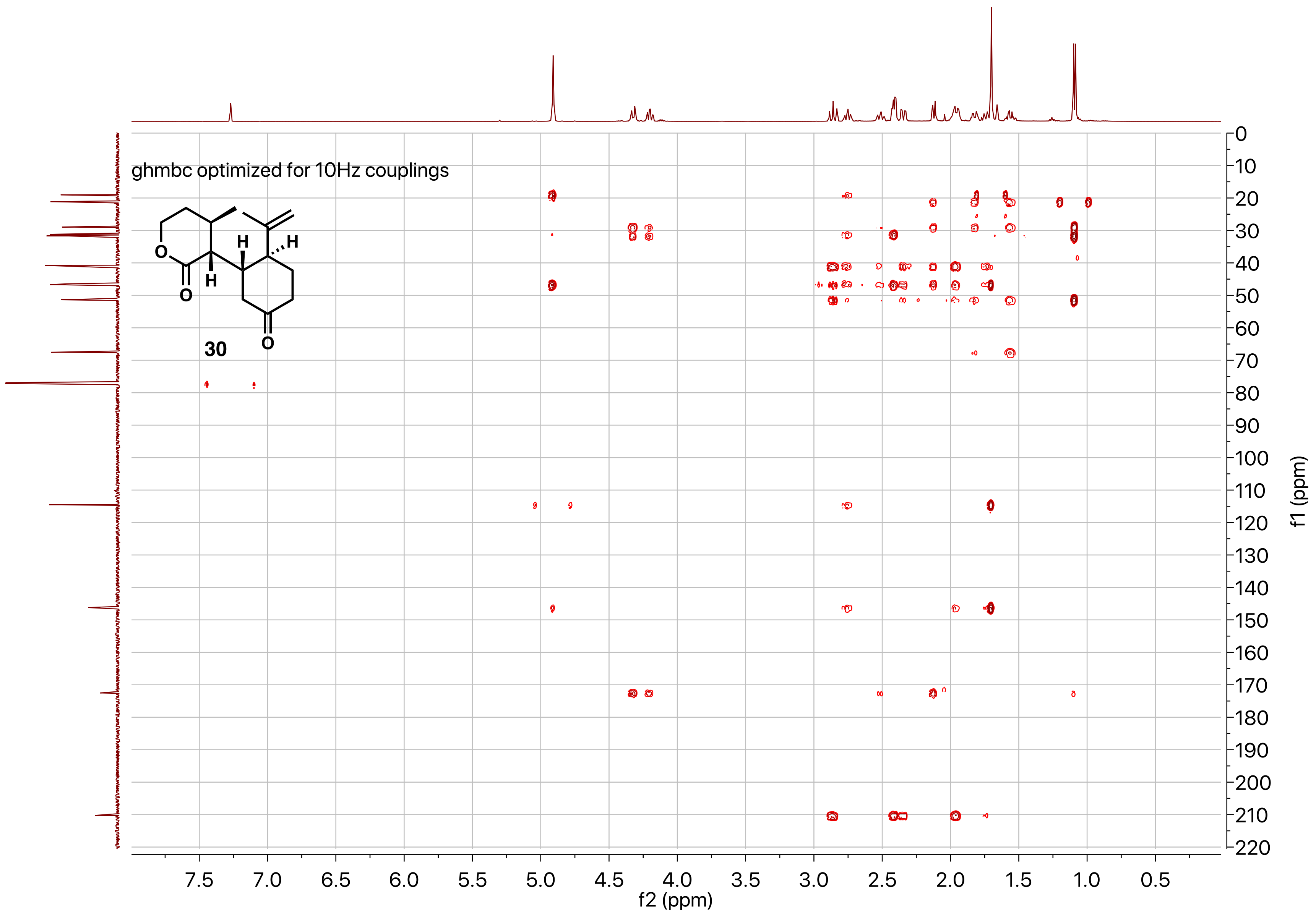
ghmbc optimized for 10Hz couplings
30
f2 (ppm)
f1 (ppm)

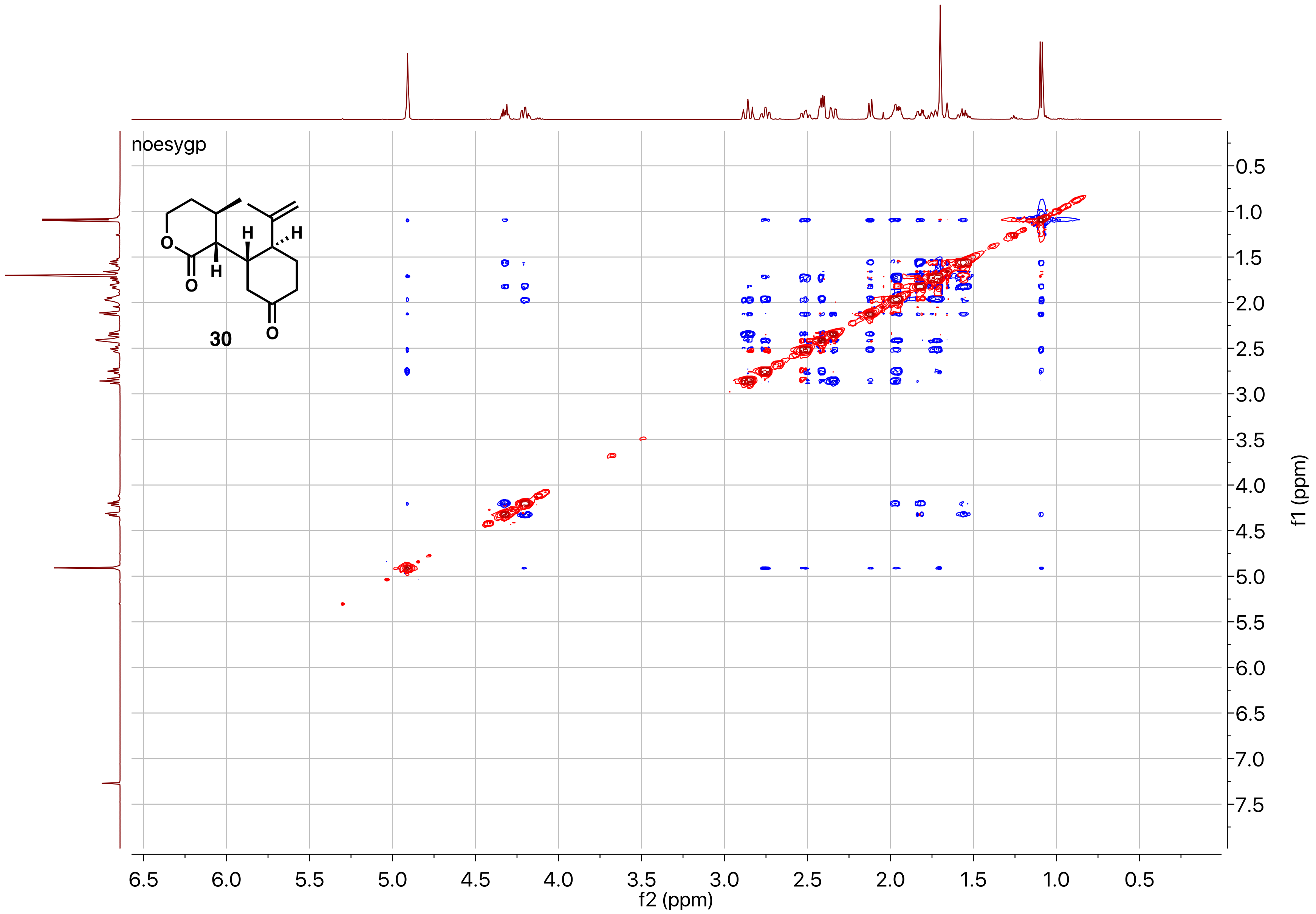
noesygp
30
f2 (ppm)
f1 (ppm)

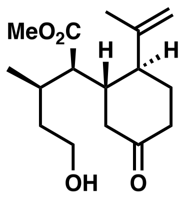

31

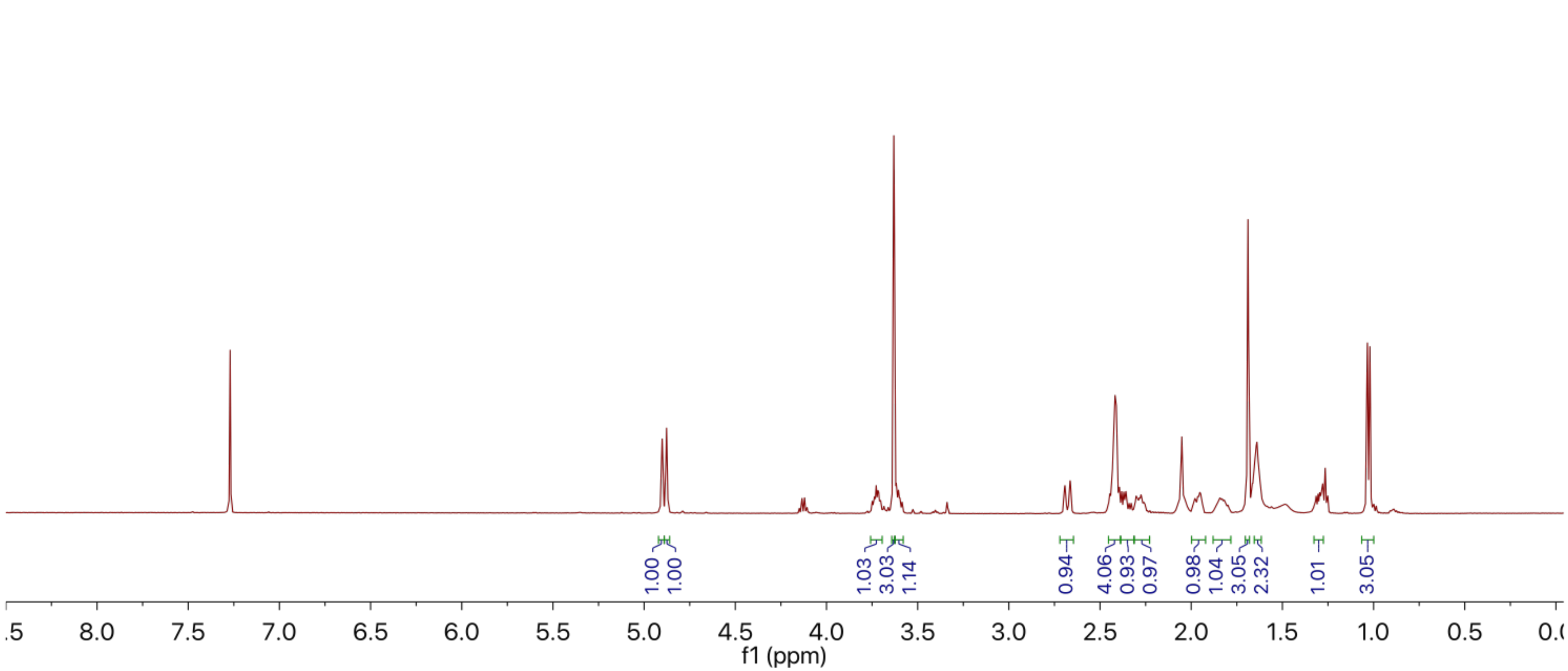
4.90 4.90 4.90 4.88 3.75 3.74 3.74 3.73 3.72 3.71 3.71 3.68 3.63 3.62 3.61 3.60 3.59 2.69 2.67 2.66 2.46 2.45 2.43 2.42 2.41 2.41 2.39 2.38 2.37 2.36 2.35 2.34 2.33 2.30 2.28 2.28 2.26 2.25 1.99 1.98 1.98 1.97 1.95 1.95 1.94 1.86 1.84 1.82 1.82 1.80 1.69 1.67 1.65 1.64 1.62 1.62 1.33 1.32 1.30 1.30 1.29 1.28 1.28 1.27 1.26 1.25 1.03 1.02
1.00
1.00
1.03
3.03
1.14
0.94
4.06
0.93
0.97
0.98
1.04
3.05
2.32
1.01
3.05
f1 (ppm)

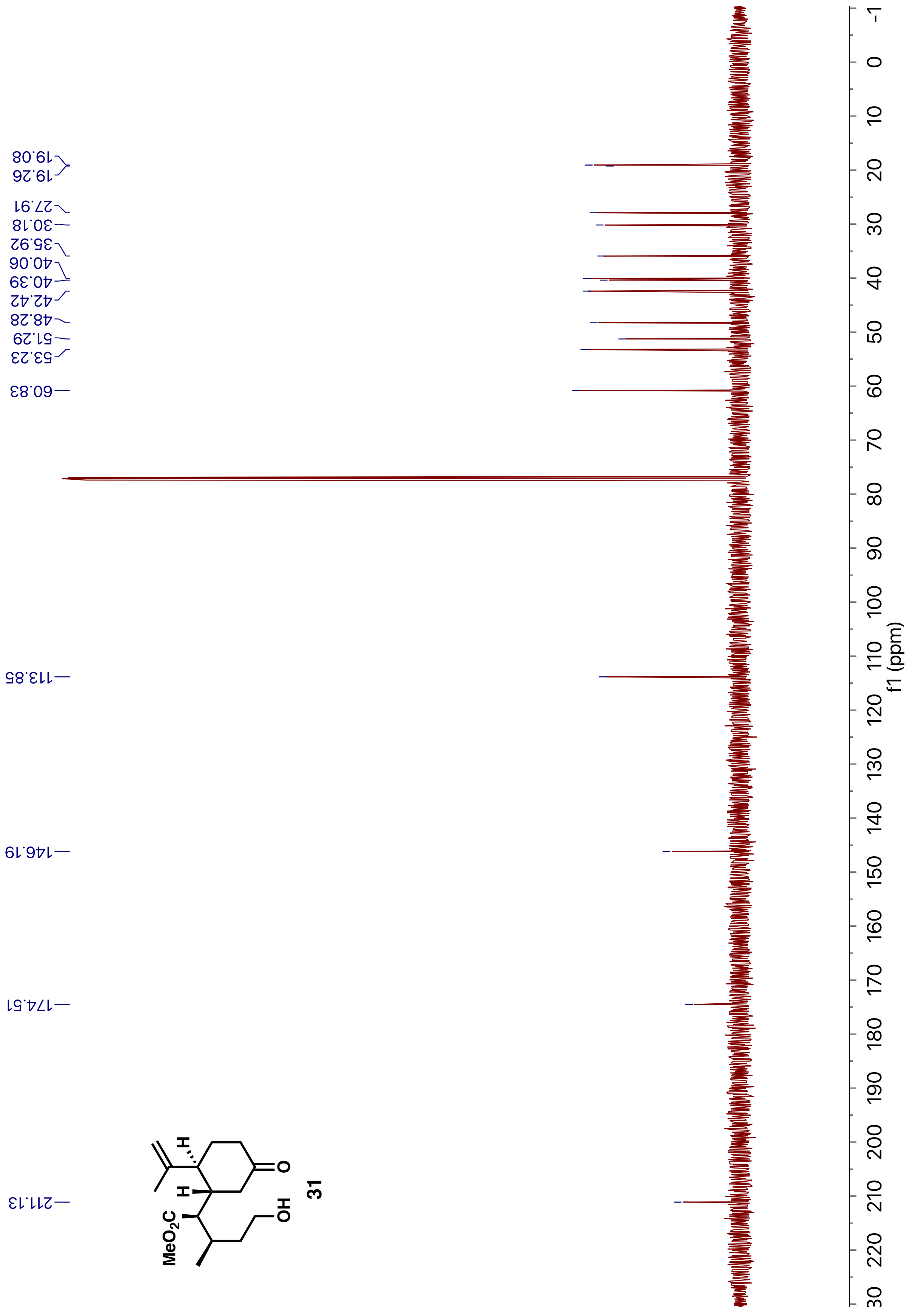
19.08
19.26
27.91
30.18
35.92
40.06
40.39
42.42
48.28
51.29
53.23
60.83
113.85
146.19
174.51
211.13
f1 (ppm)
MeO2C
H
H
O
OH
31

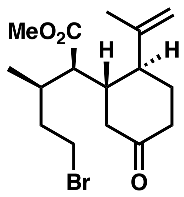

32

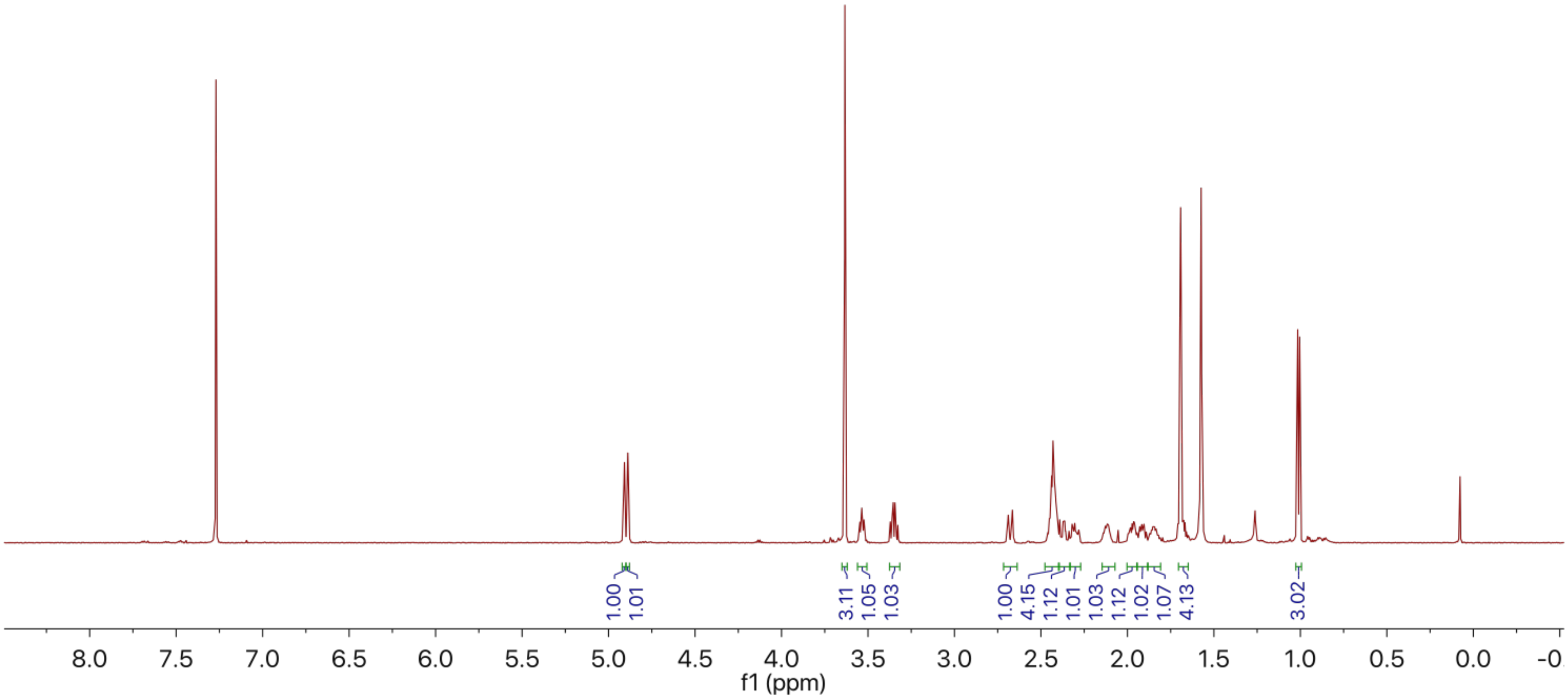

4.91 4.91 4.91 4.89 3.63 3.55 3.55 3.54 3.54 3.53 3.52 3.52 3.37 3.36 3.35 3.34 3.34 3.33 2.69 2.69 2.69 2.68 2.67 2.67 2.66 2.45 2.45 2.45 2.44 2.44 2.44 2.43 2.43 2.42 2.42 2.42 2.41 2.41 2.40 2.39 2.39 2.38 2.38 2.37 2.37 2.36 2.32 2.30 2.13 2.12 2.12 2.11 1.97 1.96 1.96 1.92 1.90 1.71 1.70 1.69 1.69 1.68 1.68 1.68 1.67 1.67 1.02 1.00
1.00
1.01
3.11
1.05
1.03
1.00
4.15
1.12
1.01
1.03
1.12
1.02
1.07
4.13
3.02
8.0 7.5 7.0 6.5 6.0 5.5 5.0 4.5 4.0 3.5 3.0 2.5 2.0 1.5 1.0 0.5 0.0 -0
f1 (ppm)

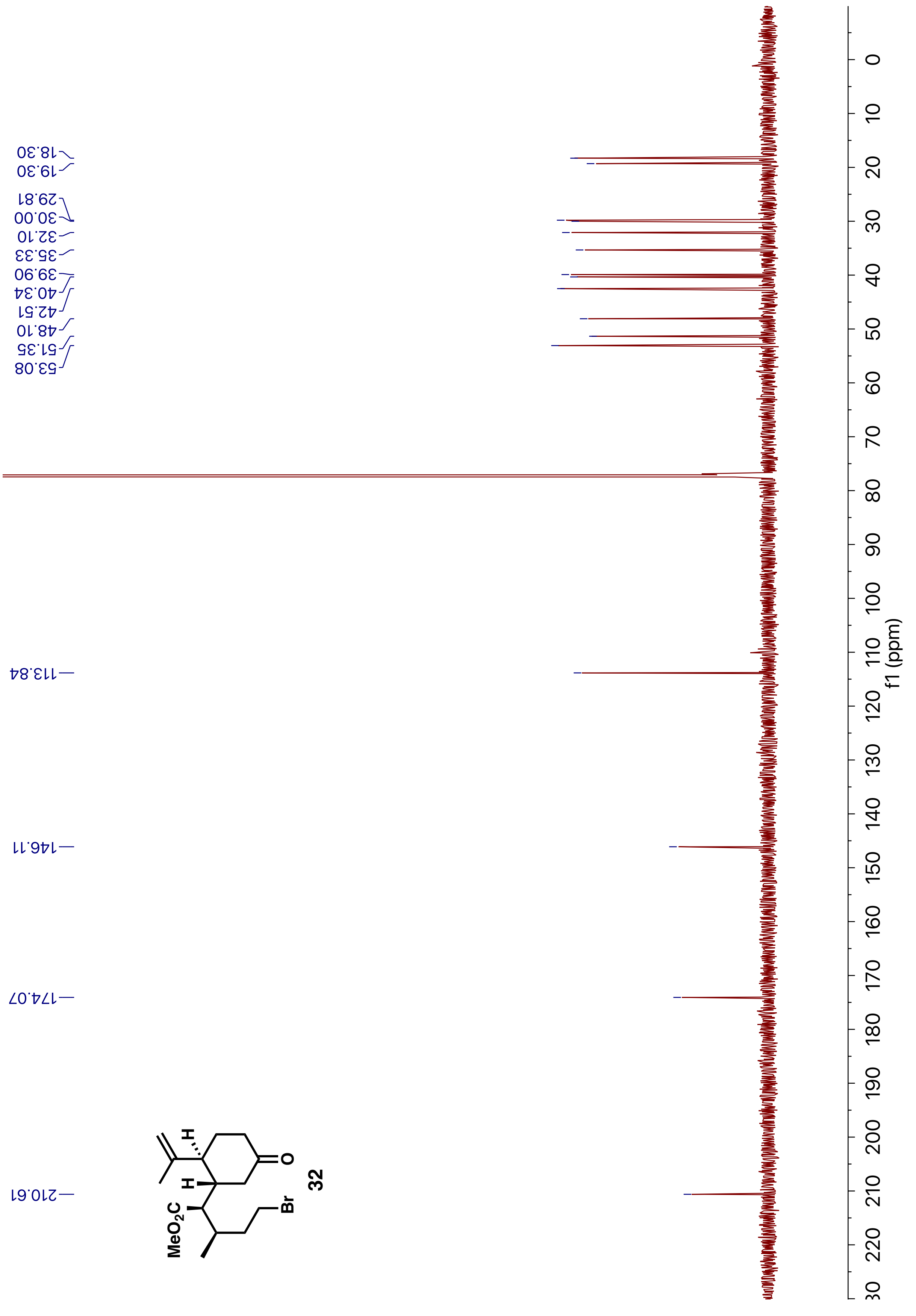
18.30
19.30
29.81
30.00
32.10
35.33
39.90
40.34
42.51
48.10
51.35
53.08
113.84
146.11
174.07
210.61
f1 (ppm)
MeO2C
H
H
O
Br

32

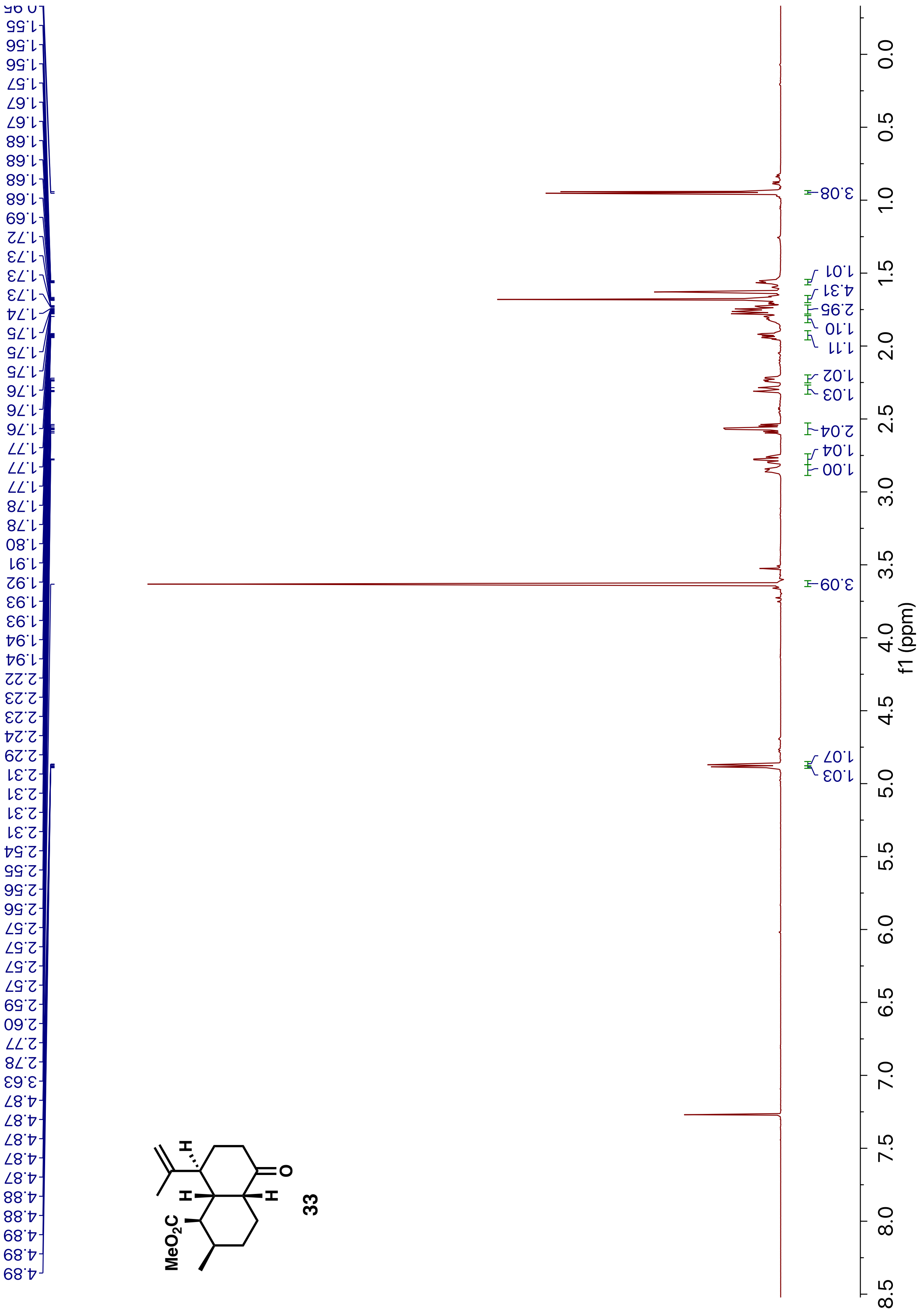

f1 (ppm)
MeO2C
H
O
33

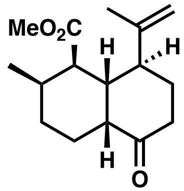
MeO2C
H
H
H
O

33

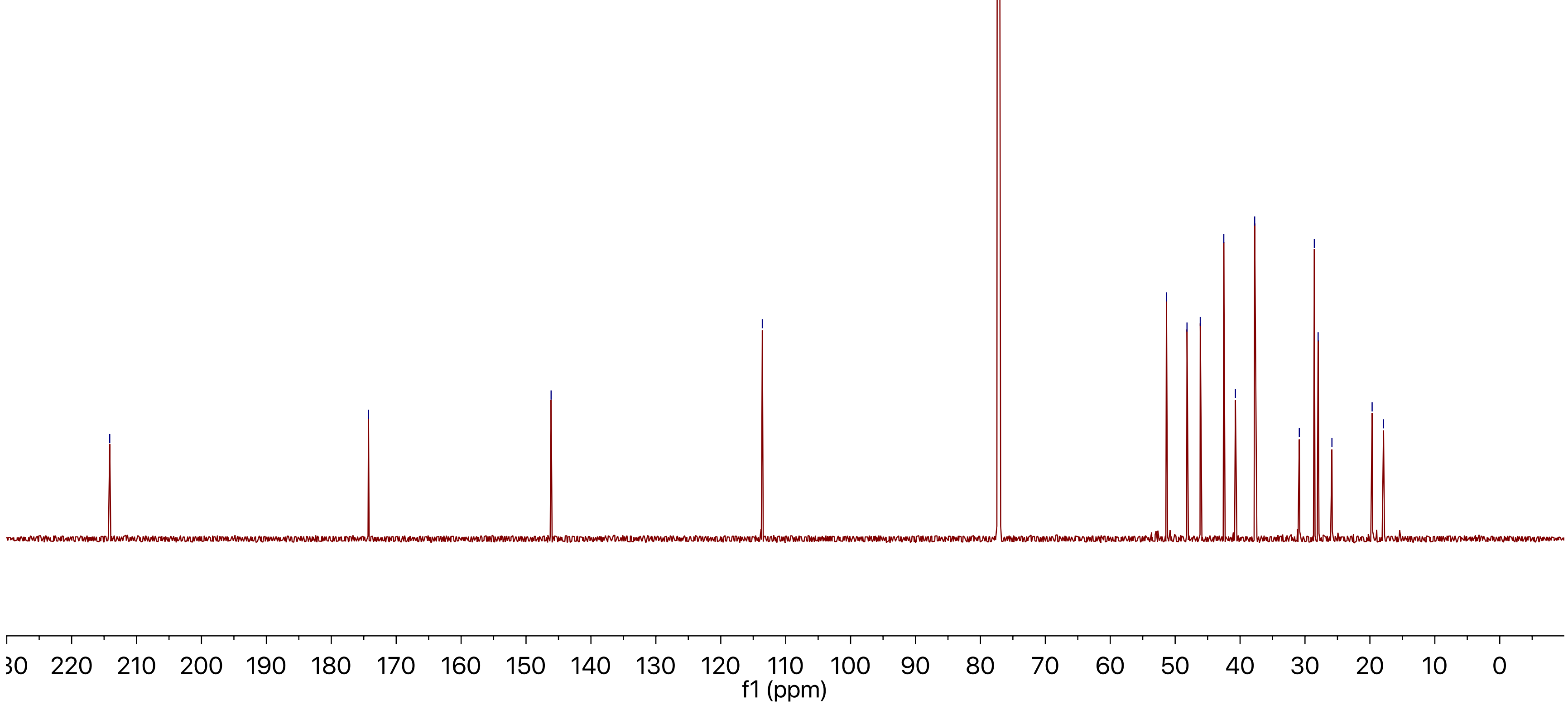
214.13
174.27
146.14
113.60
51.34
48.18
46.13
42.51
40.72
37.75
30.87
28.56
27.98
25.85
19.66
17.90
f1 (ppm)

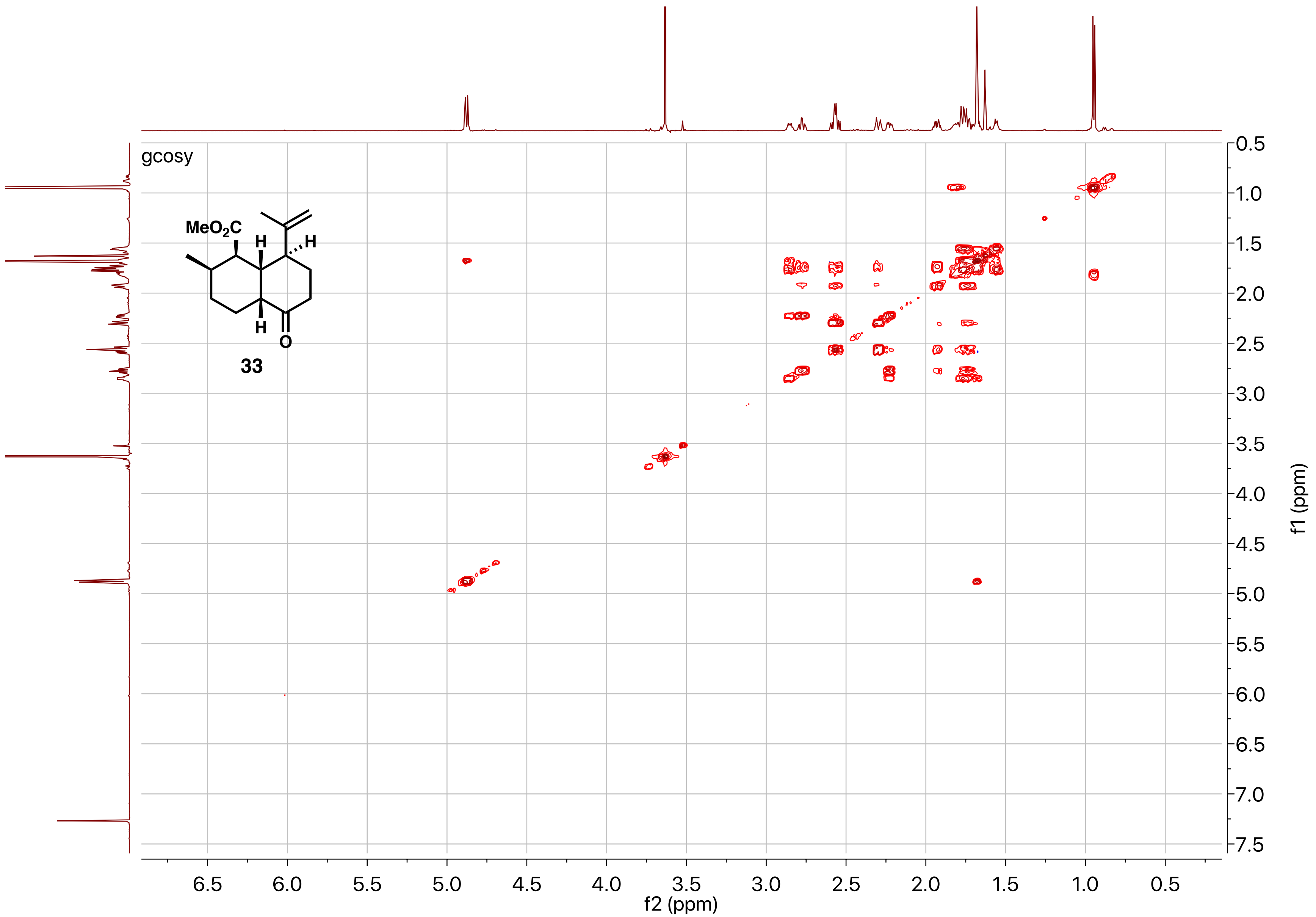
gcosy
MeO2C
H
H
H
O
33
6.5 6.0 5.5 5.0 4.5 4.0 3.5 3.0 2.5 2.0 1.5 1.0 0.5
f2 (ppm)
0.5 1.0 1.5 2.0 2.5 3.0 3.5 4.0 4.5 5.0 5.5 6.0 6.5 7.0 7.5
f1 (ppm)

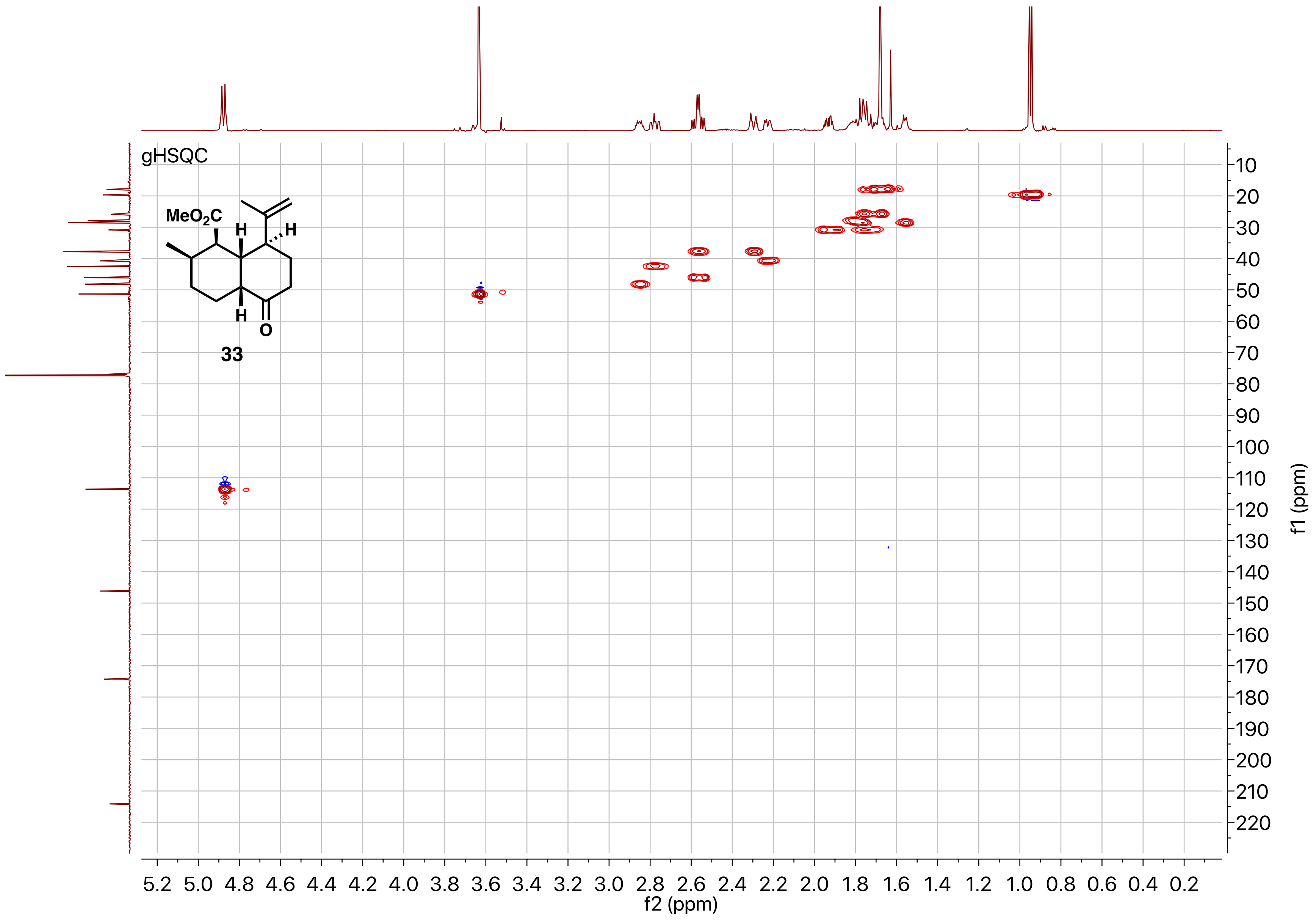
gHSQC
MeO2C
H
H
H
O
33
f2 (ppm)
f1 (ppm)

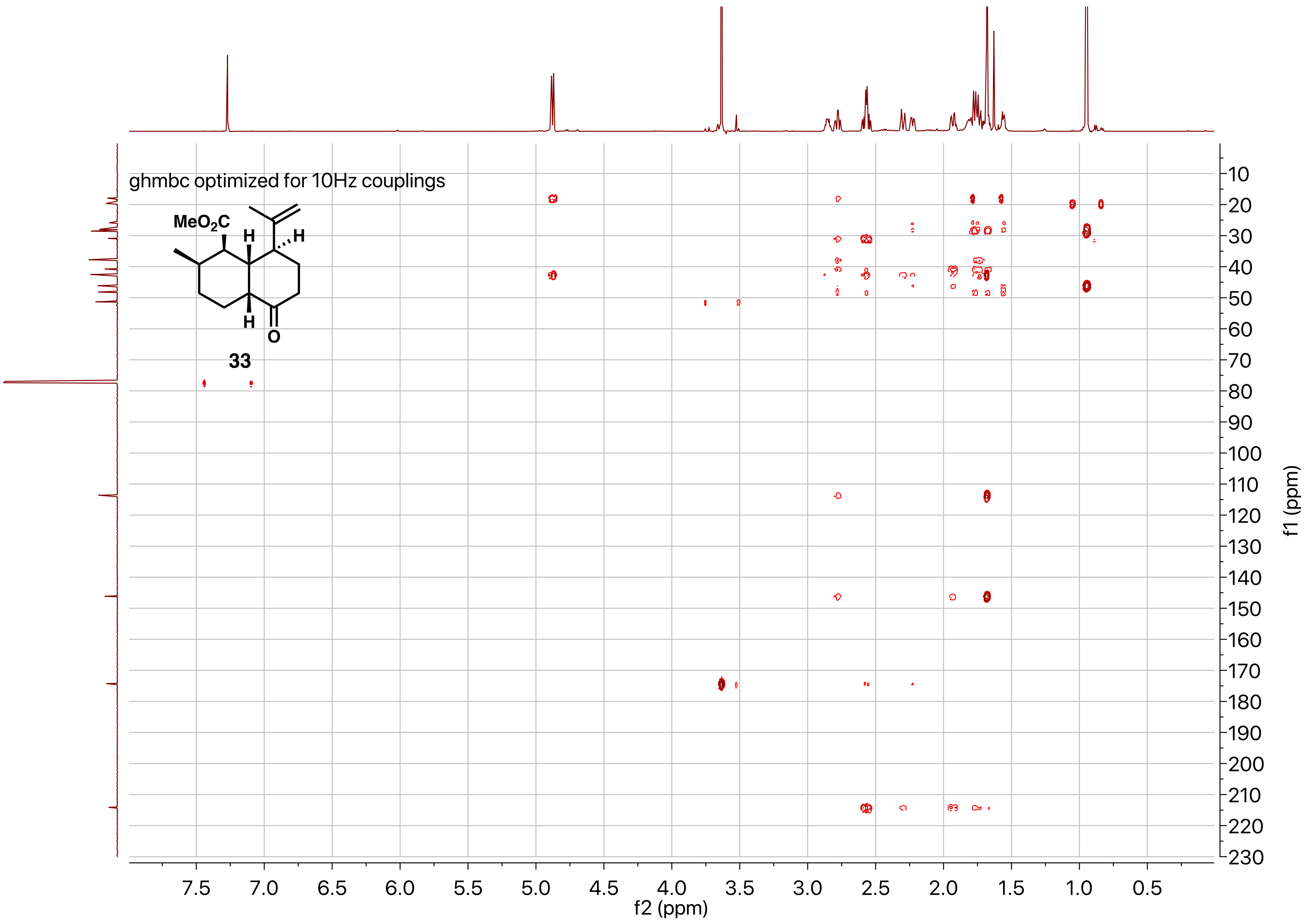

ghmbc optimized for 10Hz couplings
MeO2C
H
H
H
O
33
f2 (ppm)
f1 (ppm)

DEPTQ

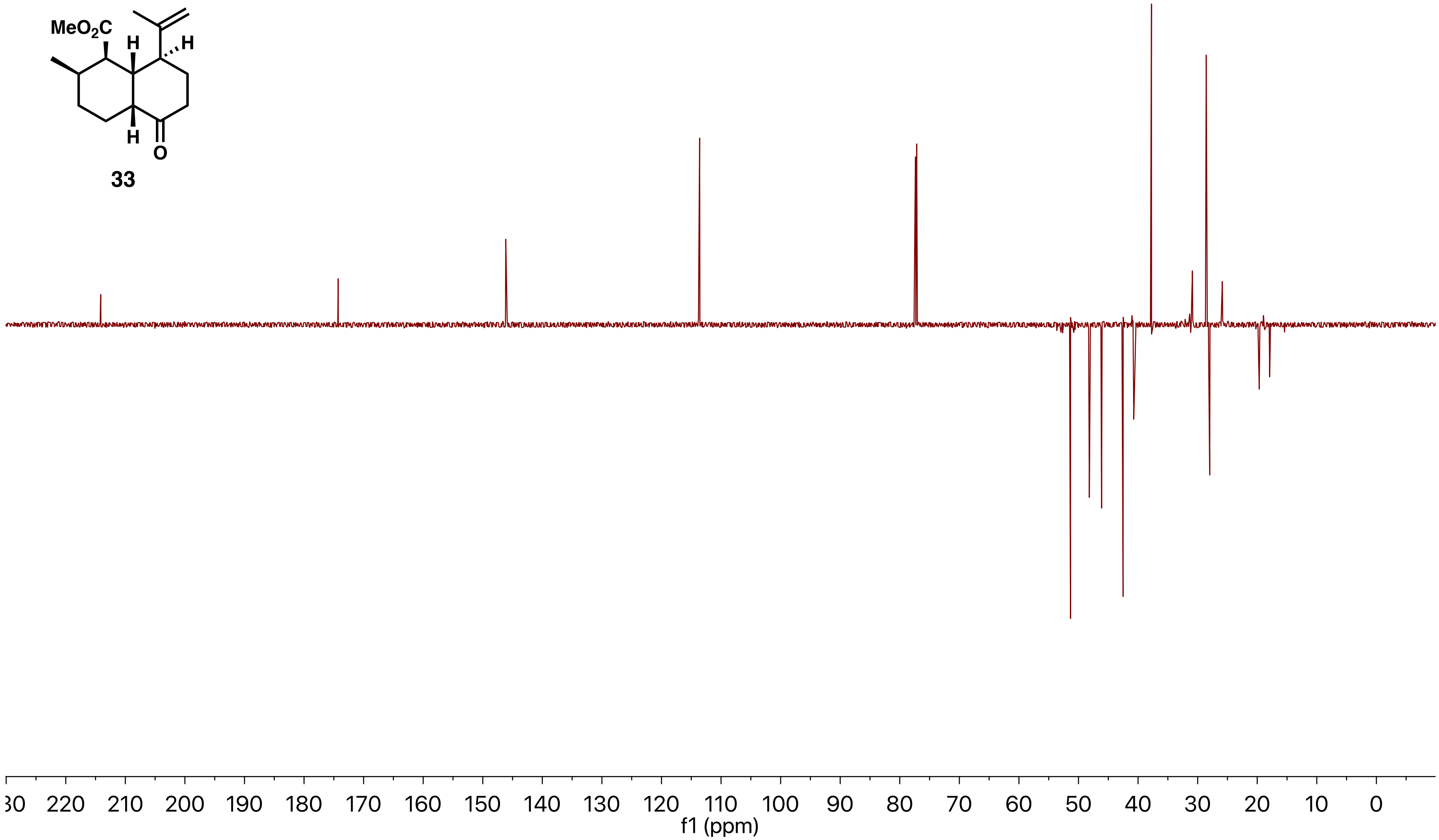
MeO2C
H
H
H
O
33
30
220
210
200
190
180
170
160
150
140
130
120
110
100
90
80
70
60
50
40
30
20
10
0
f1 (ppm)

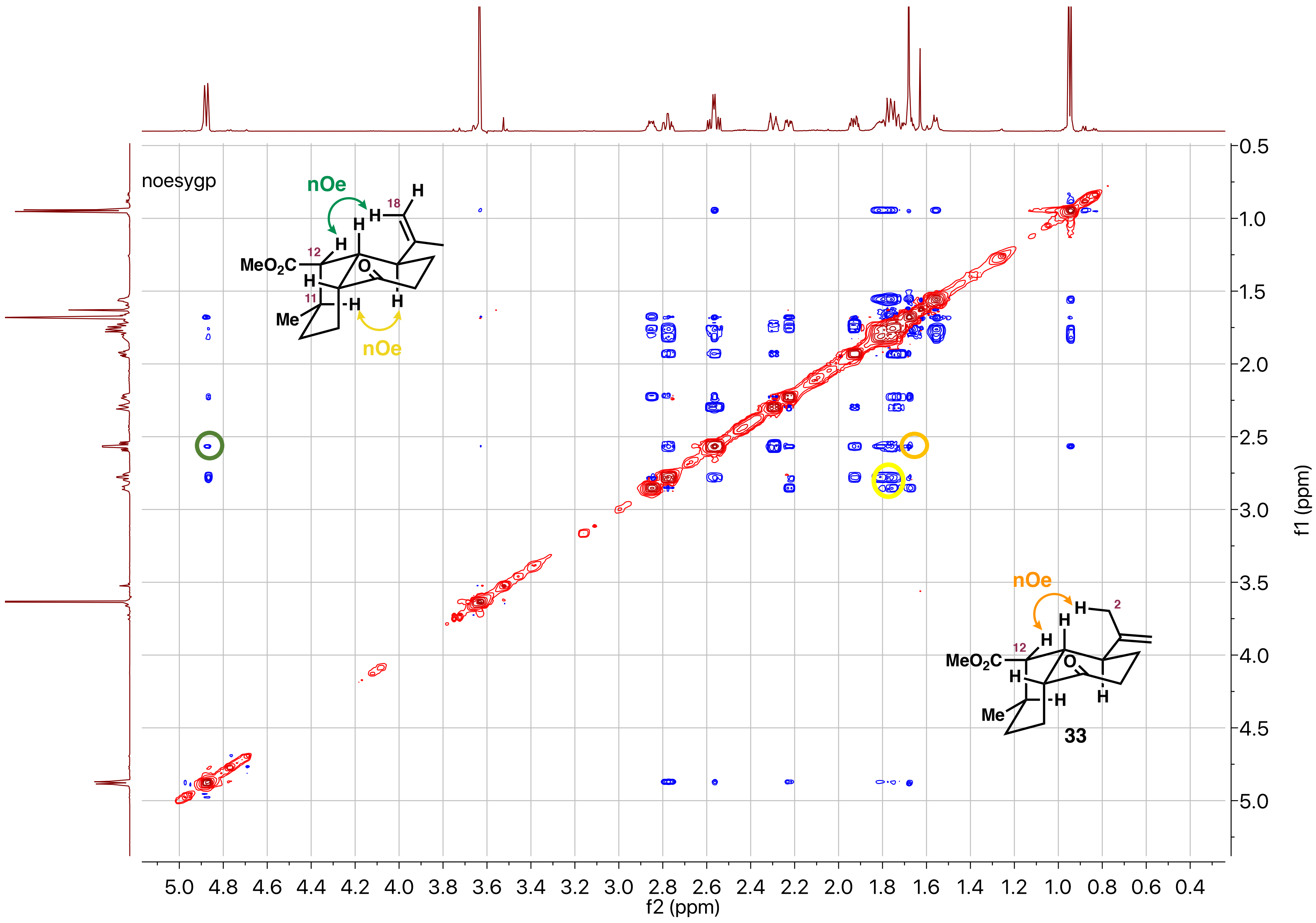
noesygp
nOe
nOe
nOe
MeO2C
Me
18
12
11
2
33
f2 (ppm)
f1 (ppm)

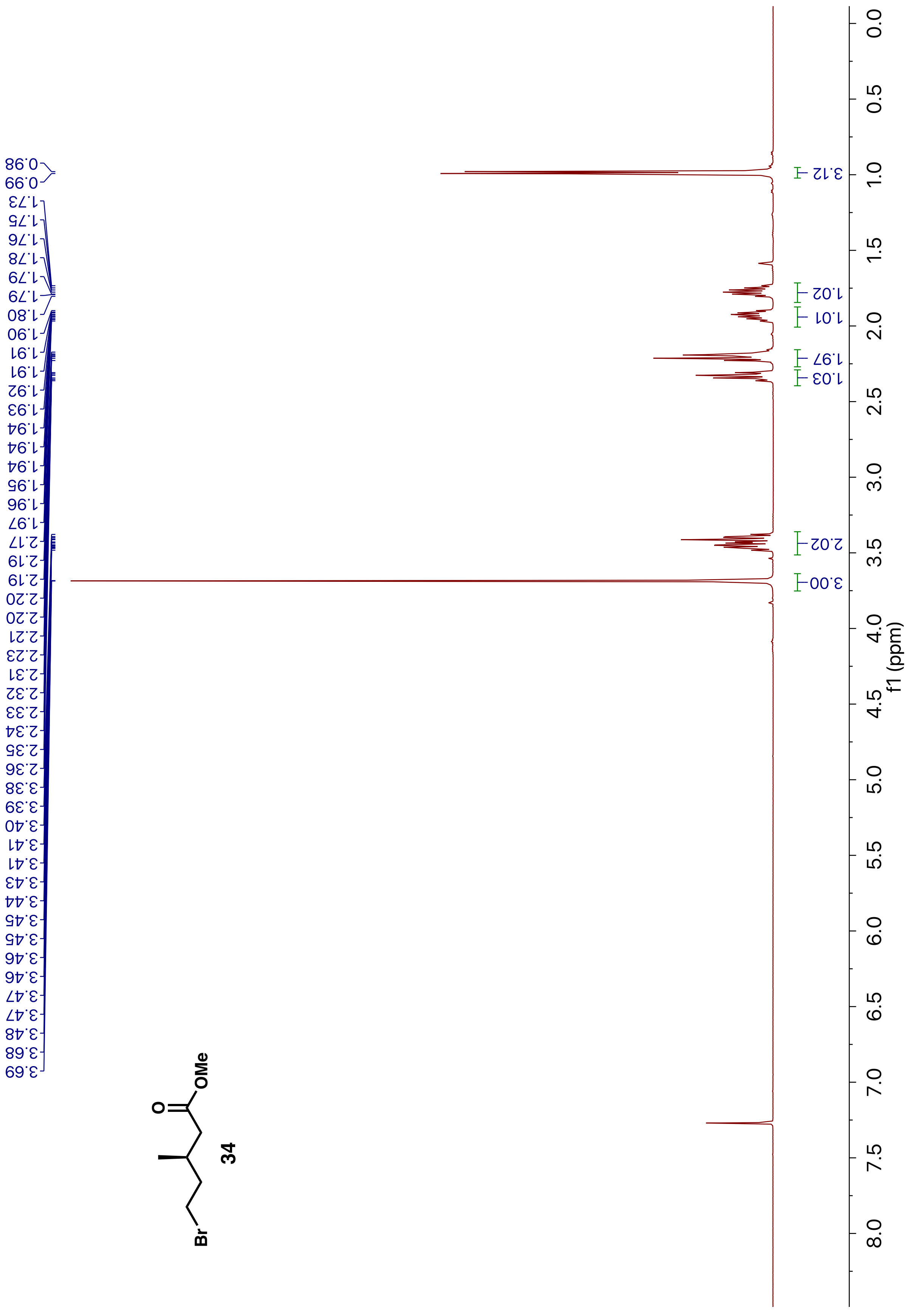
0.98
0.99
1.73
1.75
1.76
1.78
1.79
1.79
1.80
1.90
1.91
1.91
1.92
1.93
1.94
1.94
1.94
1.95
1.96
1.97
2.17
2.19
2.19
2.20
2.20
2.21
2.23
2.31
2.32
2.33
2.34
2.35
2.36
3.38
3.39
3.40
3.41
3.41
3.43
3.44
3.45
3.45
3.46
3.46
3.47
3.47
3.48
3.68
3.69
OMe
O
Br
34
3.12
1.02
1.01
1.97
1.03
2.02
3.00
f1 (ppm)

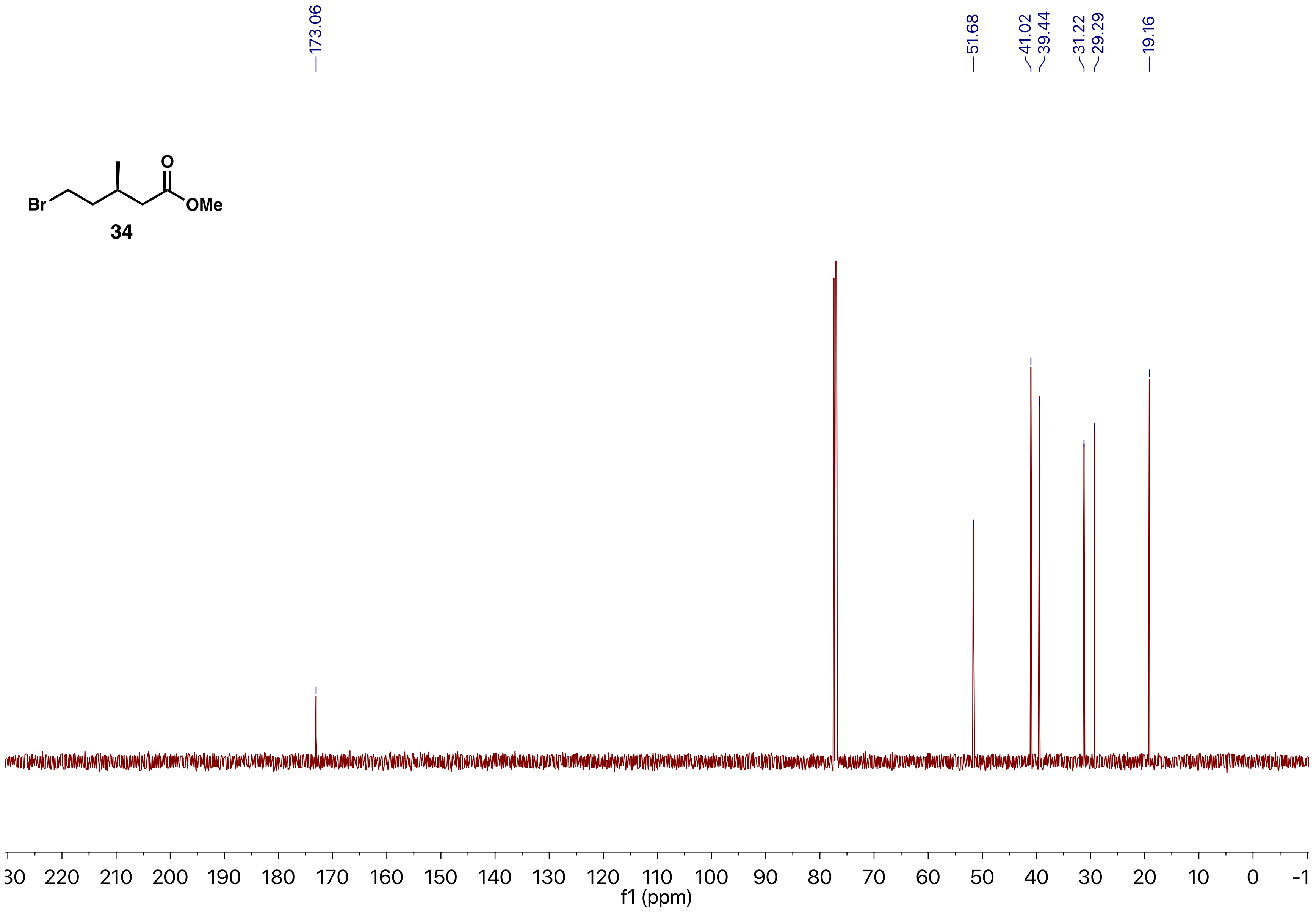

34
173.06
51.68
41.02
39.44
31.22
29.29
19.16
f1 (ppm)

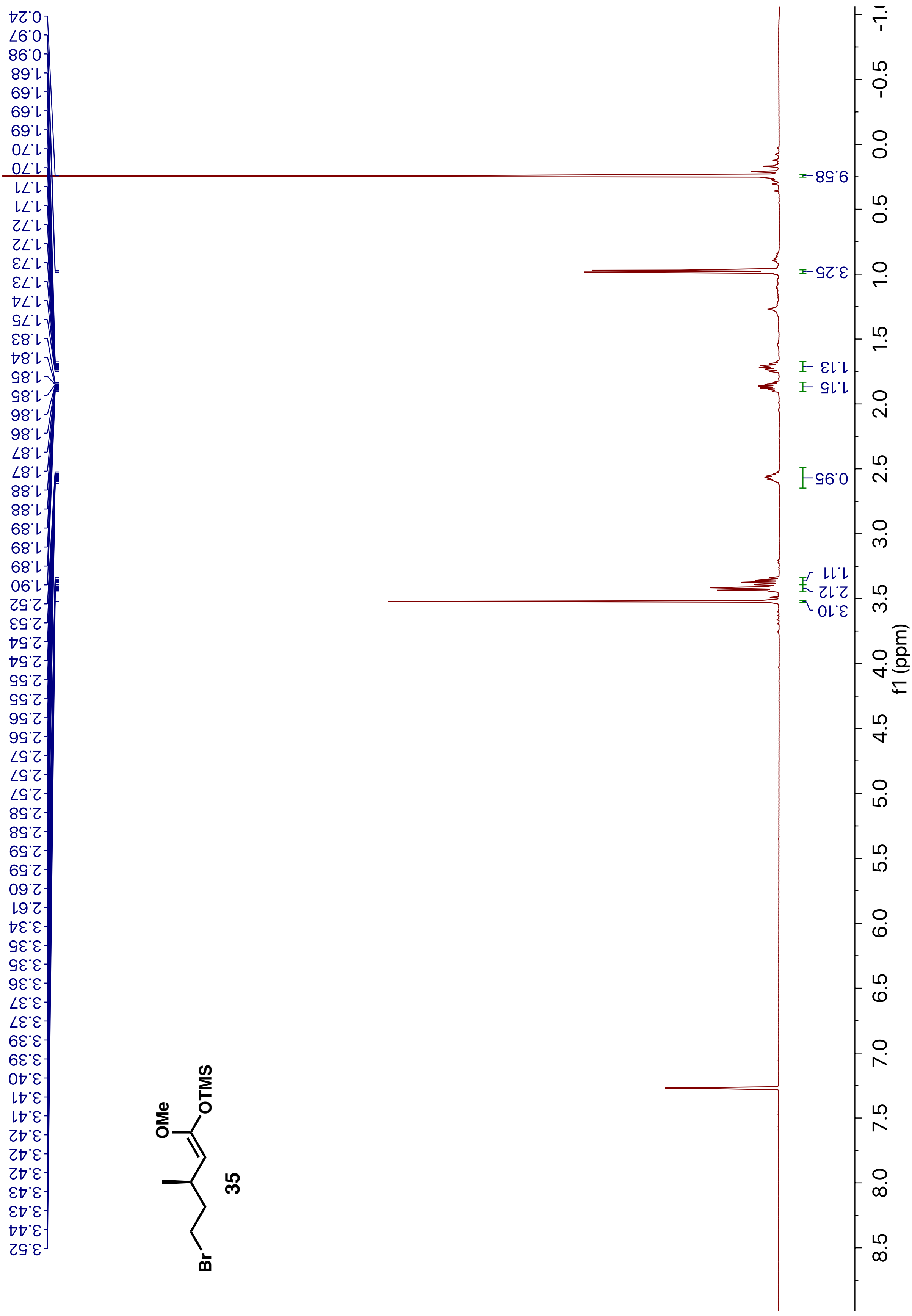

0.24
0.97
0.98
1.68
1.69
1.69
1.69
1.70
1.70
1.71
1.71
1.72
1.72
1.73
1.73
1.74
1.75
1.83
1.84
1.85
1.85
1.86
1.86
1.87
1.87
1.88
1.88
1.89
1.89
1.89
1.90
2.52
2.53
2.54
2.54
2.55
2.55
2.56
2.56
2.57
2.57
2.57
2.58
2.58
2.59
2.59
2.60
2.61
3.34
3.35
3.35
3.36
3.37
3.37
3.39
3.39
3.40
3.41
3.41
3.42
3.42
3.42
3.43
3.43
3.44
3.52
OMe
OTMS
Br
35
9.58
3.25
1.13
1.15
0.95
1.11
2.12
3.10
f1 (ppm)

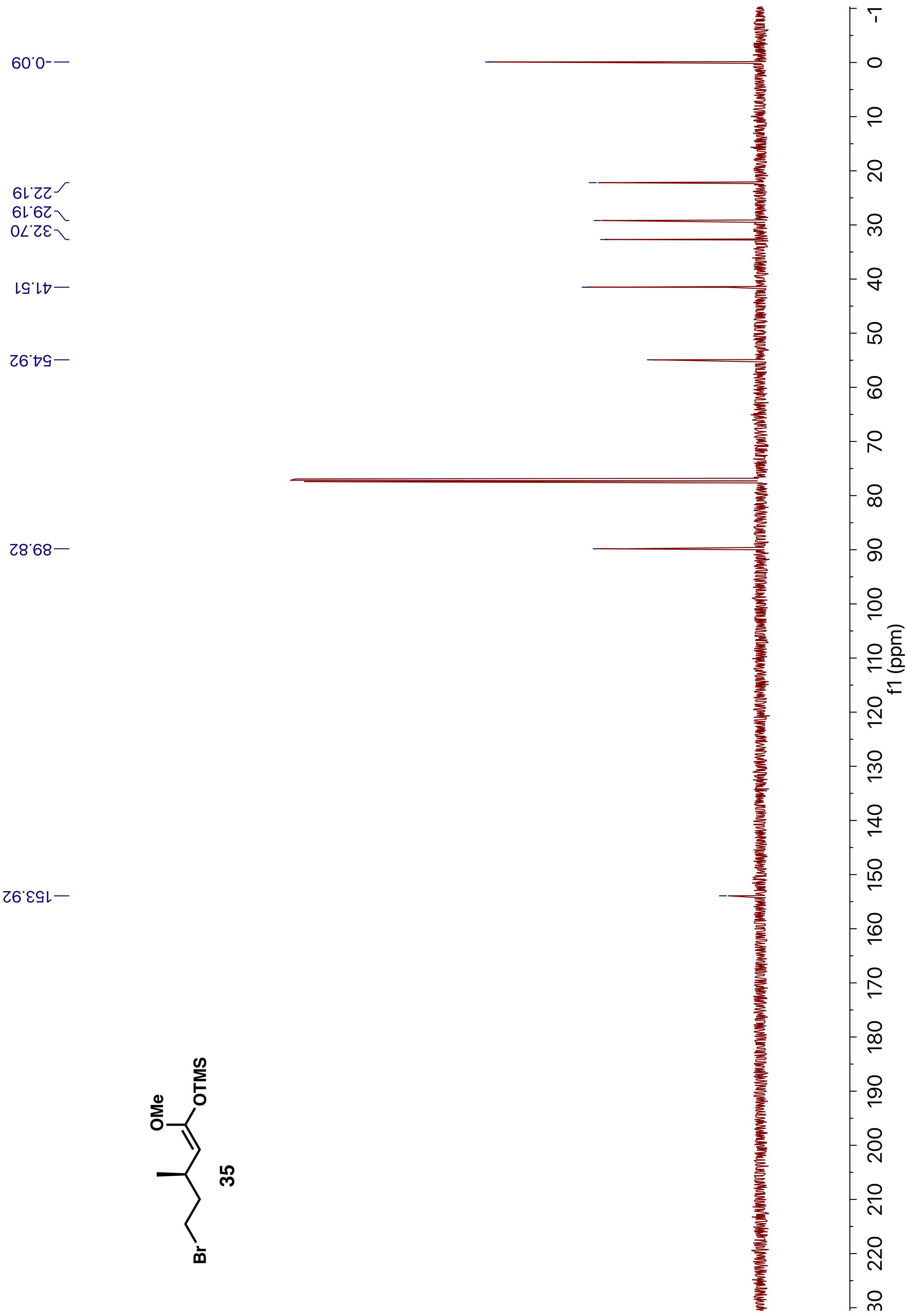

-0.09
22.19
29.19
32.70
41.51
54.92
89.82
153.92
OMe
OTMS
Br
35
f1 (ppm)

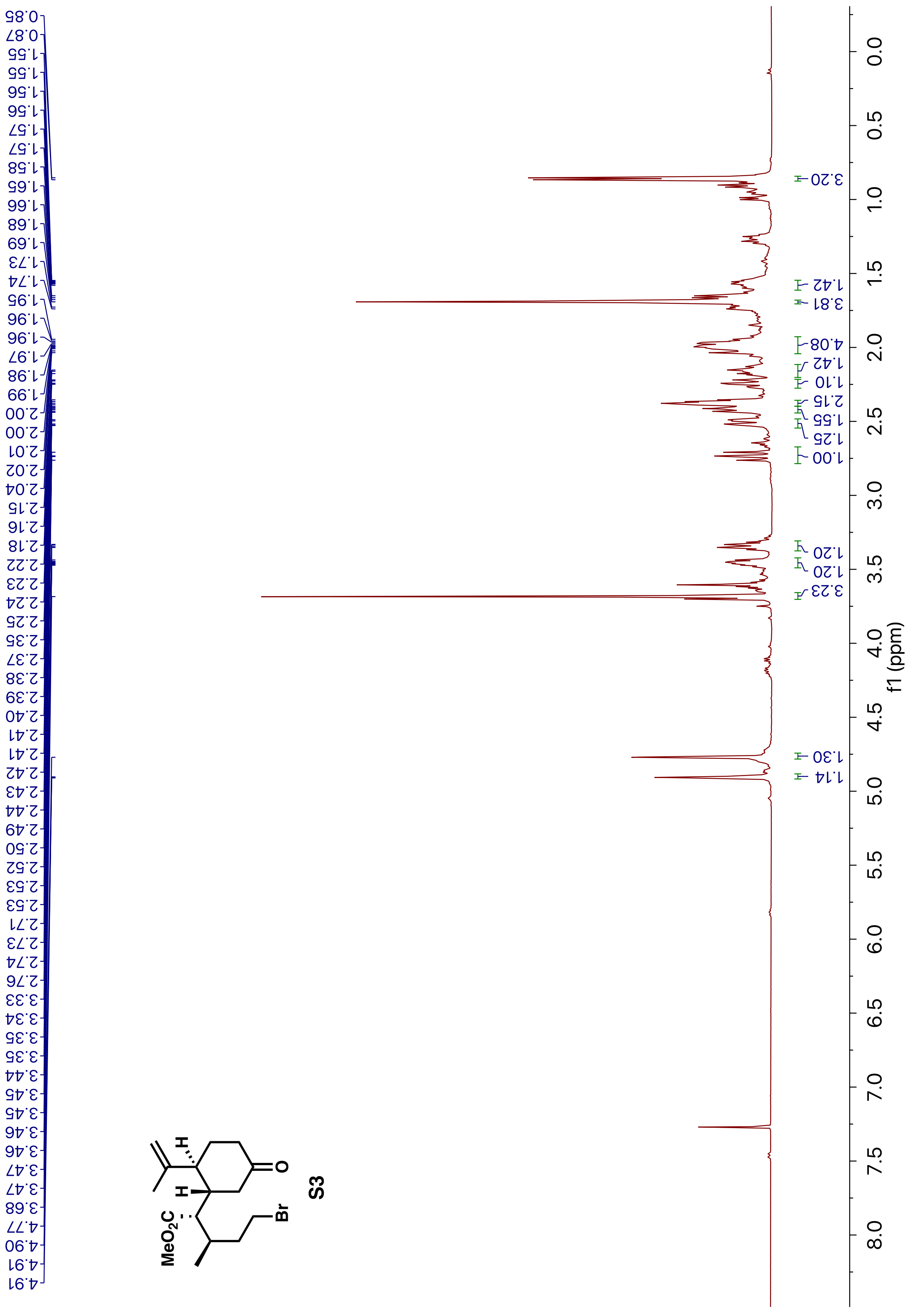
S3
MeO2C
Br
f1 (ppm)

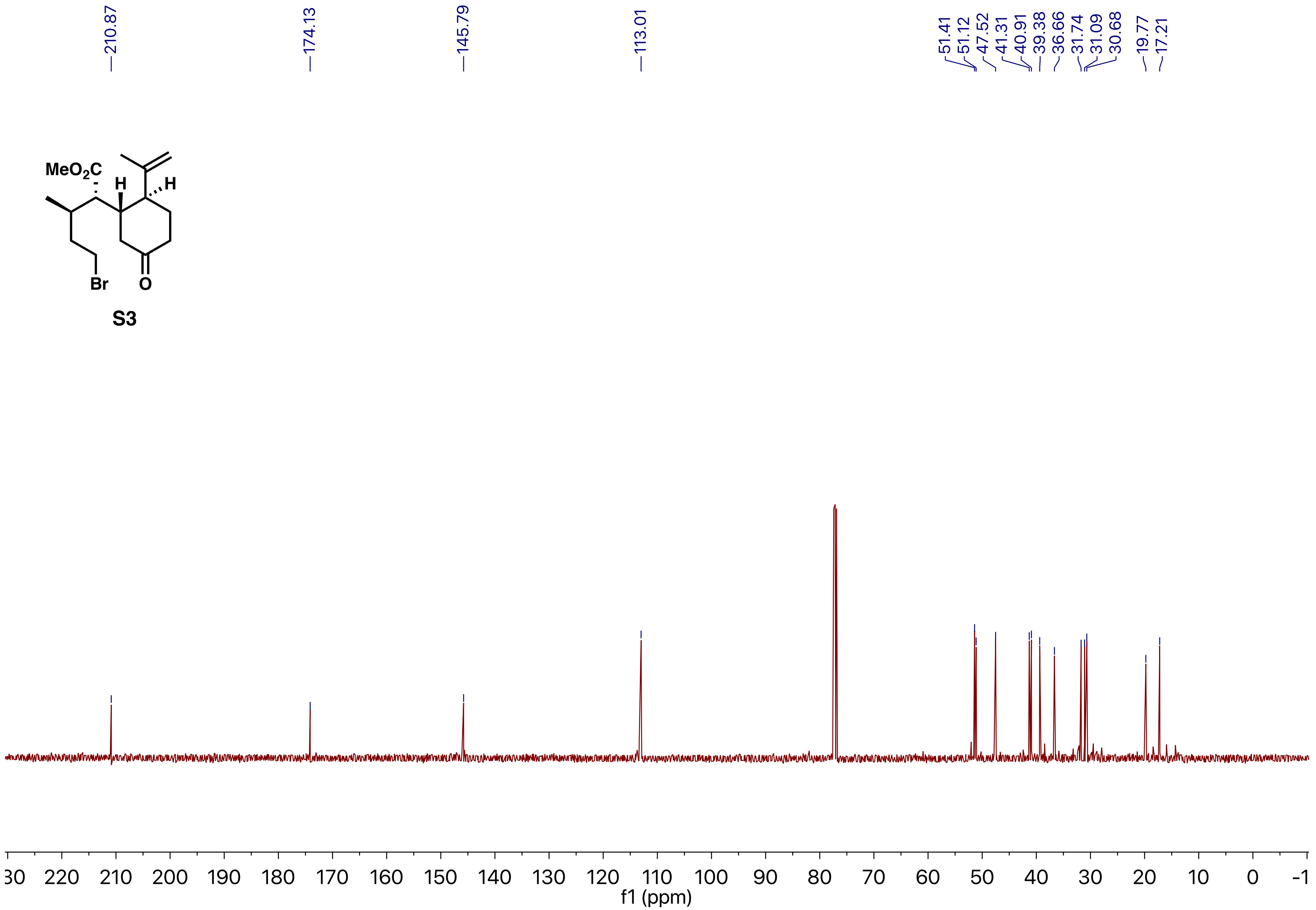
210.87
174.13
145.79
113.01
51.41
51.12
47.52
41.31
40.91
39.38
36.66
31.74
31.09
30.68
19.77
17.21
MeO2C
H
H
Br
O
S3
f1 (ppm)

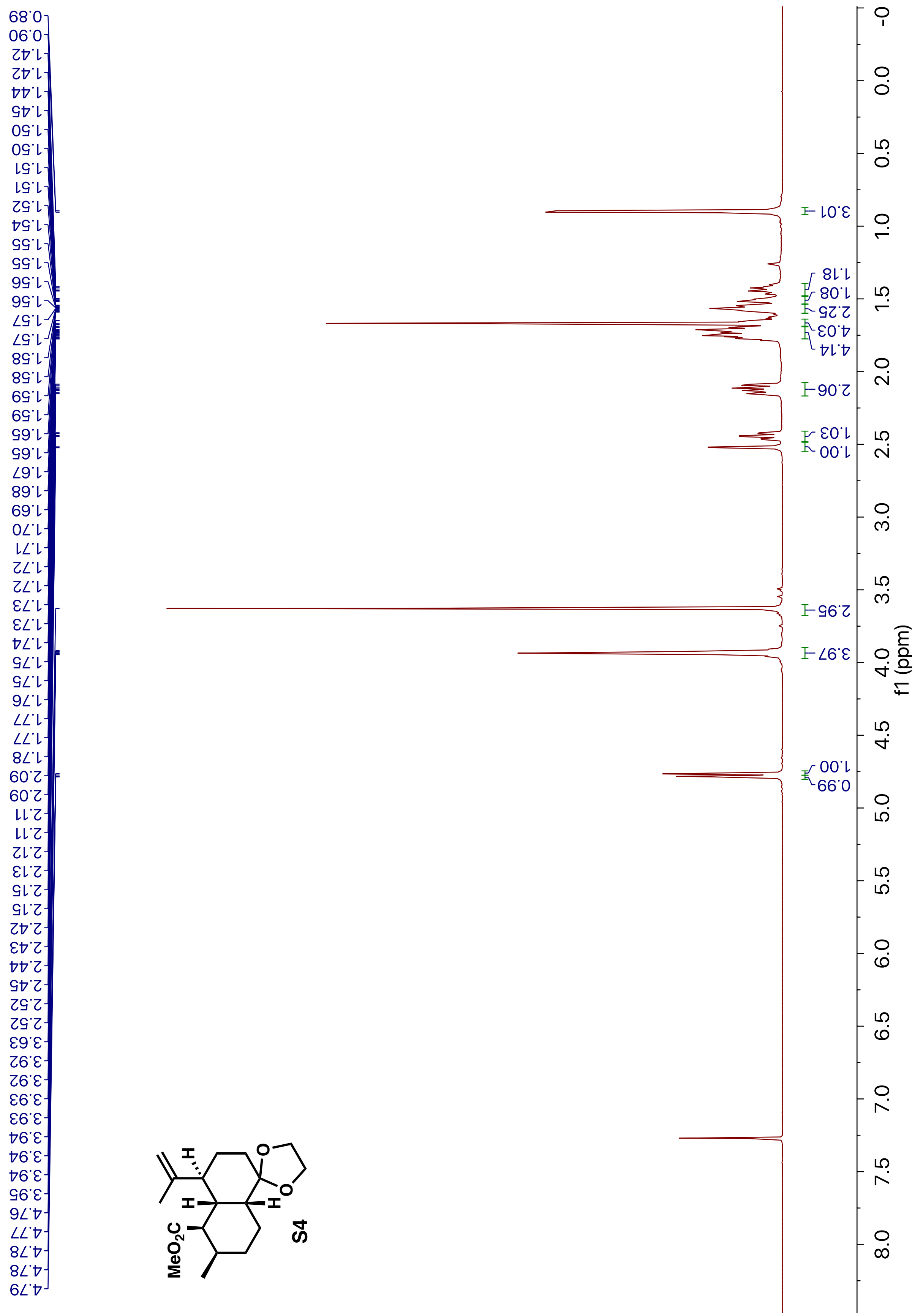
0.89
0.90
1.42
1.42
1.44
1.45
1.50
1.50
1.51
1.51
1.52
1.54
1.55
1.55
1.56
1.56
1.57
1.57
1.58
1.58
1.59
1.59
1.65
1.65
1.67
1.68
1.69
1.70
1.71
1.72
1.72
1.73
1.73
1.74
1.75
1.75
1.76
1.77
1.77
1.78
2.09
2.09
2.11
2.11
2.12
2.13
2.15
2.15
2.42
2.43
2.44
2.45
2.52
2.52
3.63
3.92
3.92
3.93
3.93
3.94
3.94
3.94
3.95
4.76
4.77
4.78
4.78
4.79
MeO2C
S4
3.01
1.18
1.08
2.25
4.03
4.14
2.06
1.03
1.00
2.95
3.97
1.00
0.99
f1 (ppm)

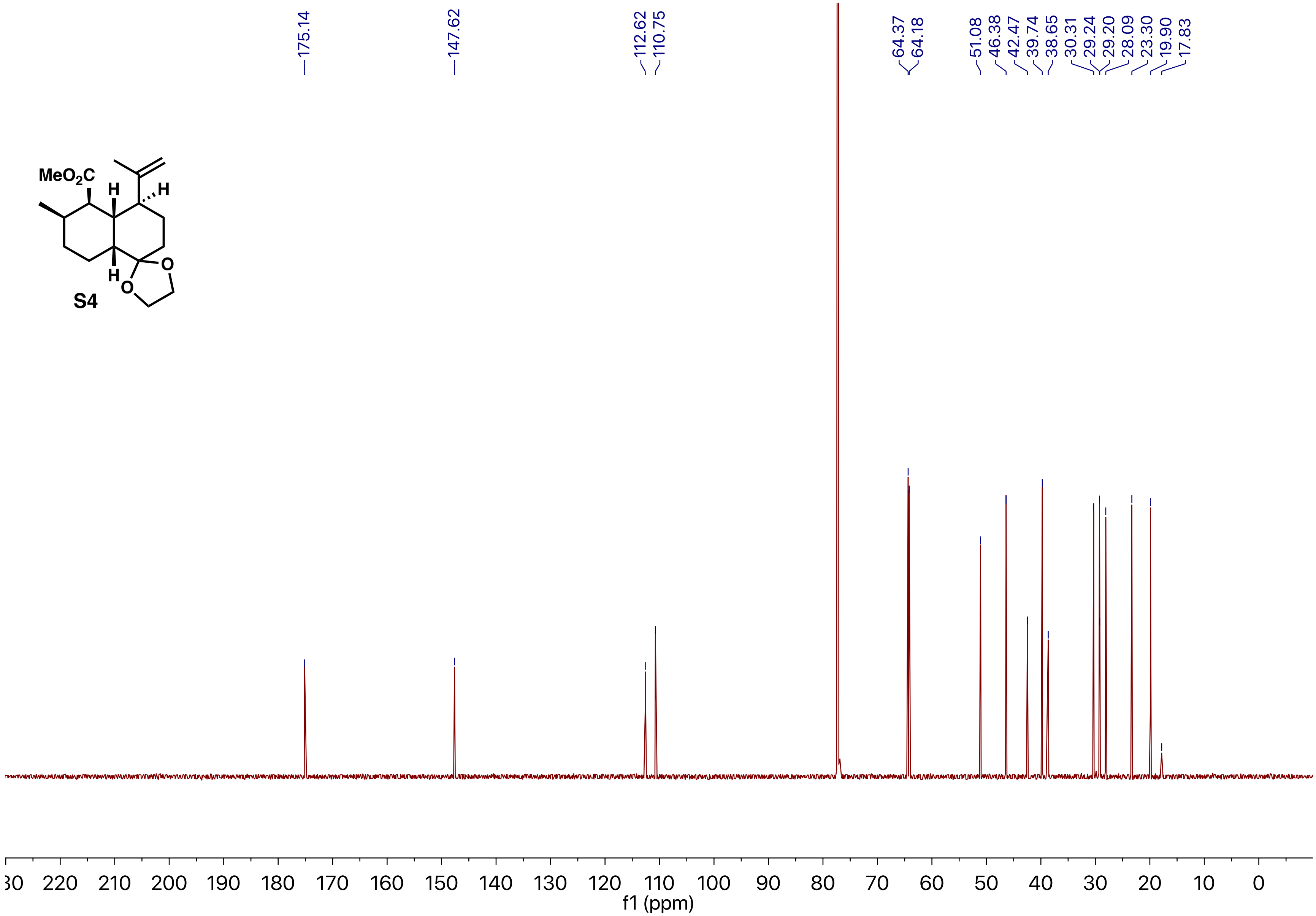
175.14
147.62
112.62
110.75
64.37
64.18
51.08
46.38
42.47
39.74
38.65
30.31
29.24
29.20
28.09
23.30
19.90
17.83
MeO2C
H
H
H
O
O
S4
f1 (ppm)

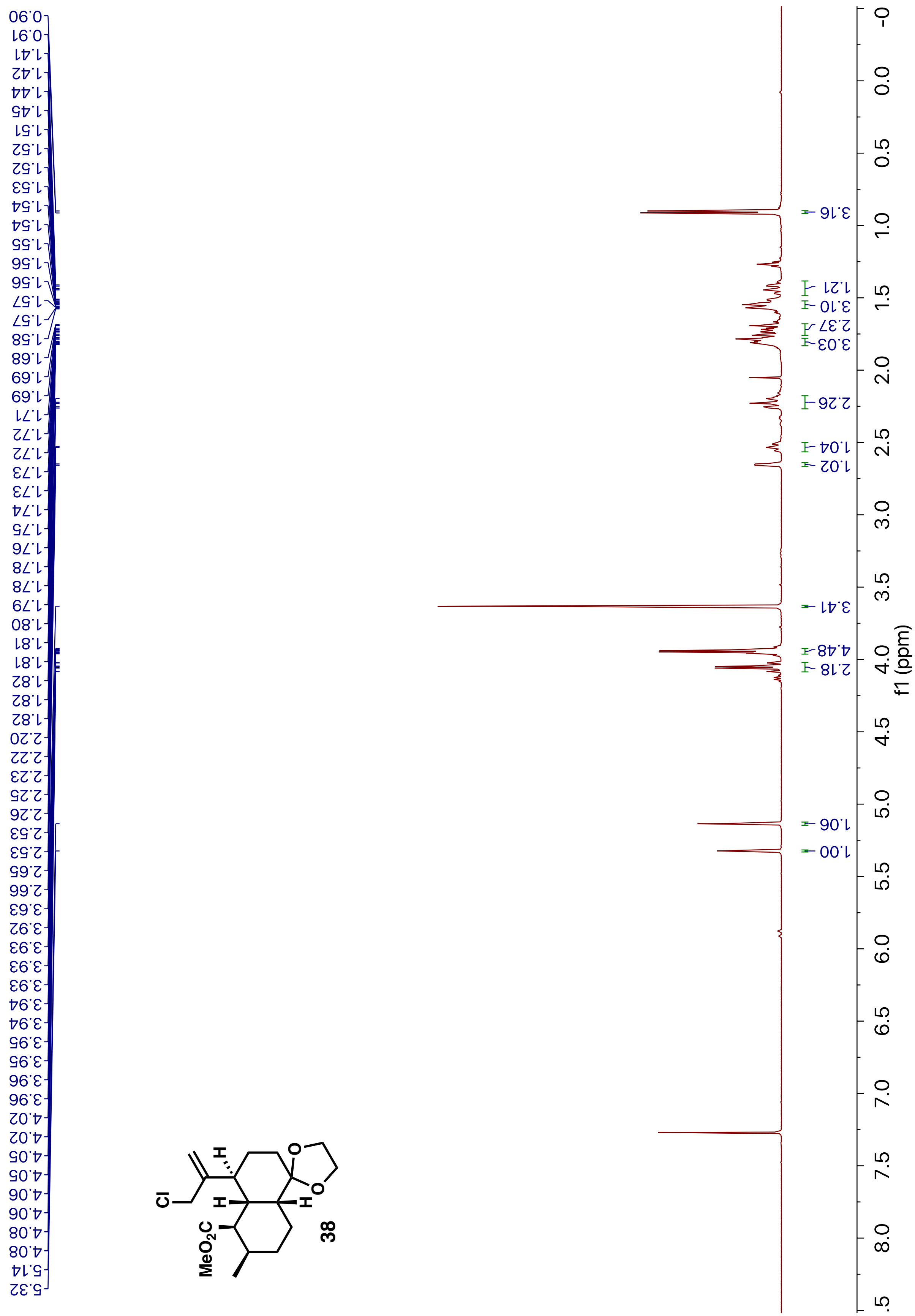

38
MeO2C
Cl
H
O
f1 (ppm)
3.16
1.21
3.10
2.37
3.03
2.26
1.04
1.02
3.41
4.48
2.18
1.06
1.00

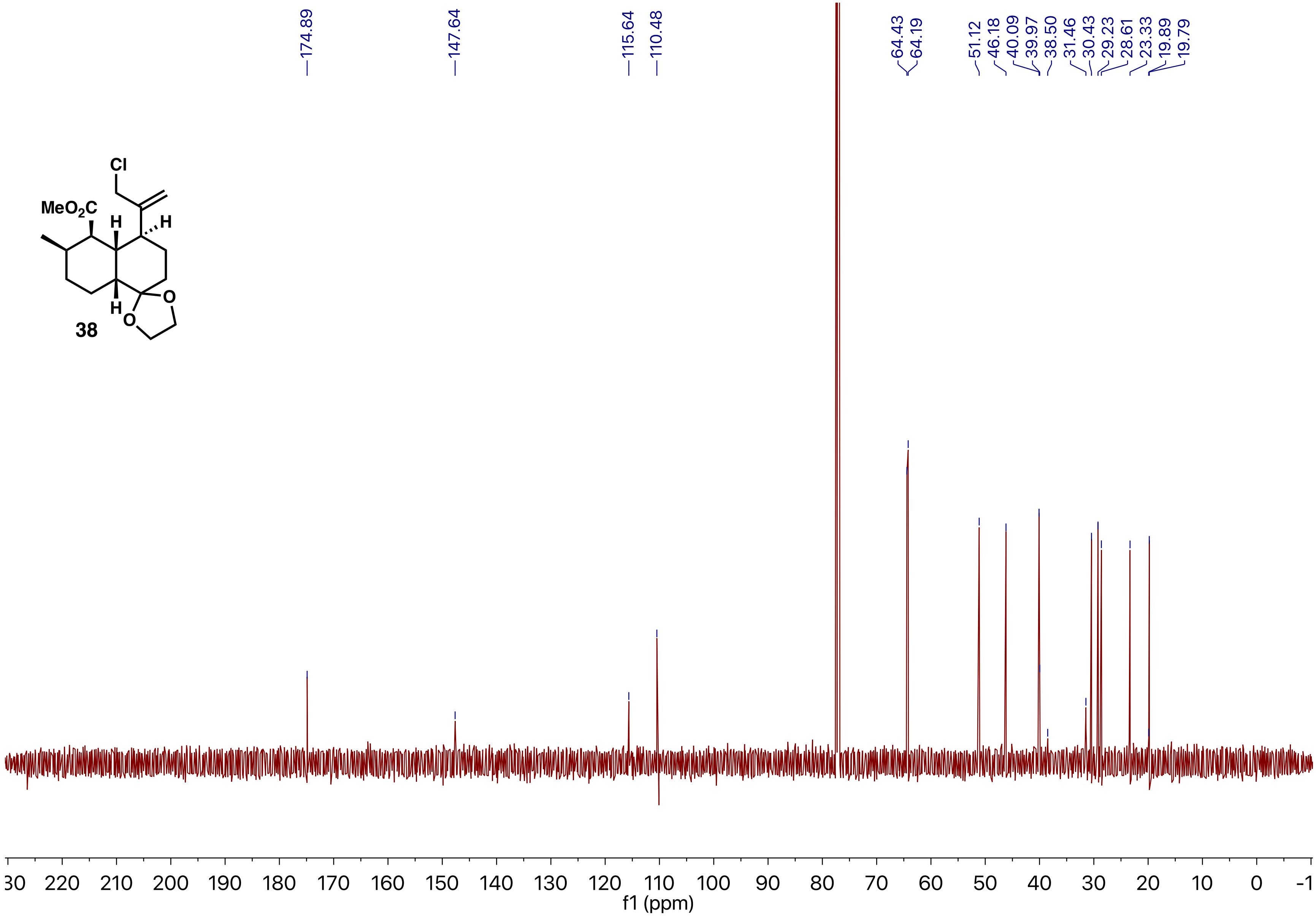

174.89
147.64
115.64
110.48
64.43
64.19
51.12
46.18
40.09
39.97
38.50
31.46
30.43
29.23
28.61
23.33
19.89
19.79
Cl
MeO2C
H
O
f1 (ppm)

38

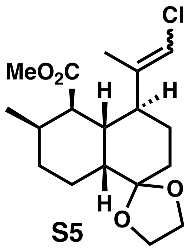
MeO2C
Cl
H
H
H
O
O

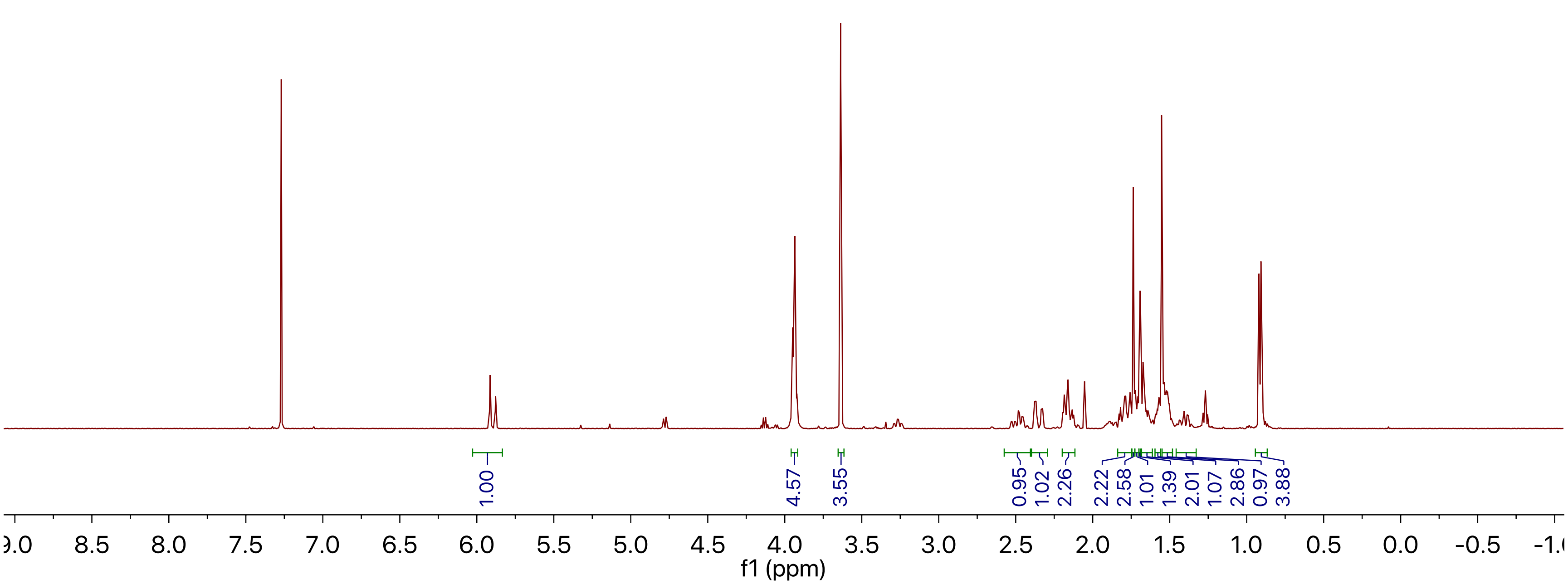
5.91 5.91 5.91 5.88 5.87 3.96 3.95 3.95 3.94 3.94 3.93 3.93 3.93 3.92 3.92 3.64 3.63 2.48 2.48 2.38 2.37 2.33 2.33 2.20 2.19 2.18 2.16 2.16 2.13 1.83 1.82 1.80 1.79 1.79 1.78 1.74 1.73 1.72 1.72 1.71 1.71 1.69 1.69 1.69 1.67 1.66 1.65 1.64 1.63 1.59 1.58 1.57 1.57 1.57 1.55 1.55 1.54 1.53 1.53 1.52 1.51 1.50 1.41 1.41 0.92 0.92 0.91 0.90
1.00
4.57
3.55
0.95
1.02
2.26
2.22
2.58
1.01
1.39
2.01
1.07
2.86
0.97
3.88
f1 (ppm)

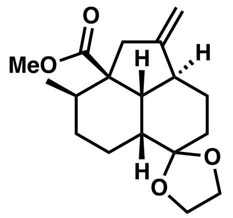

39

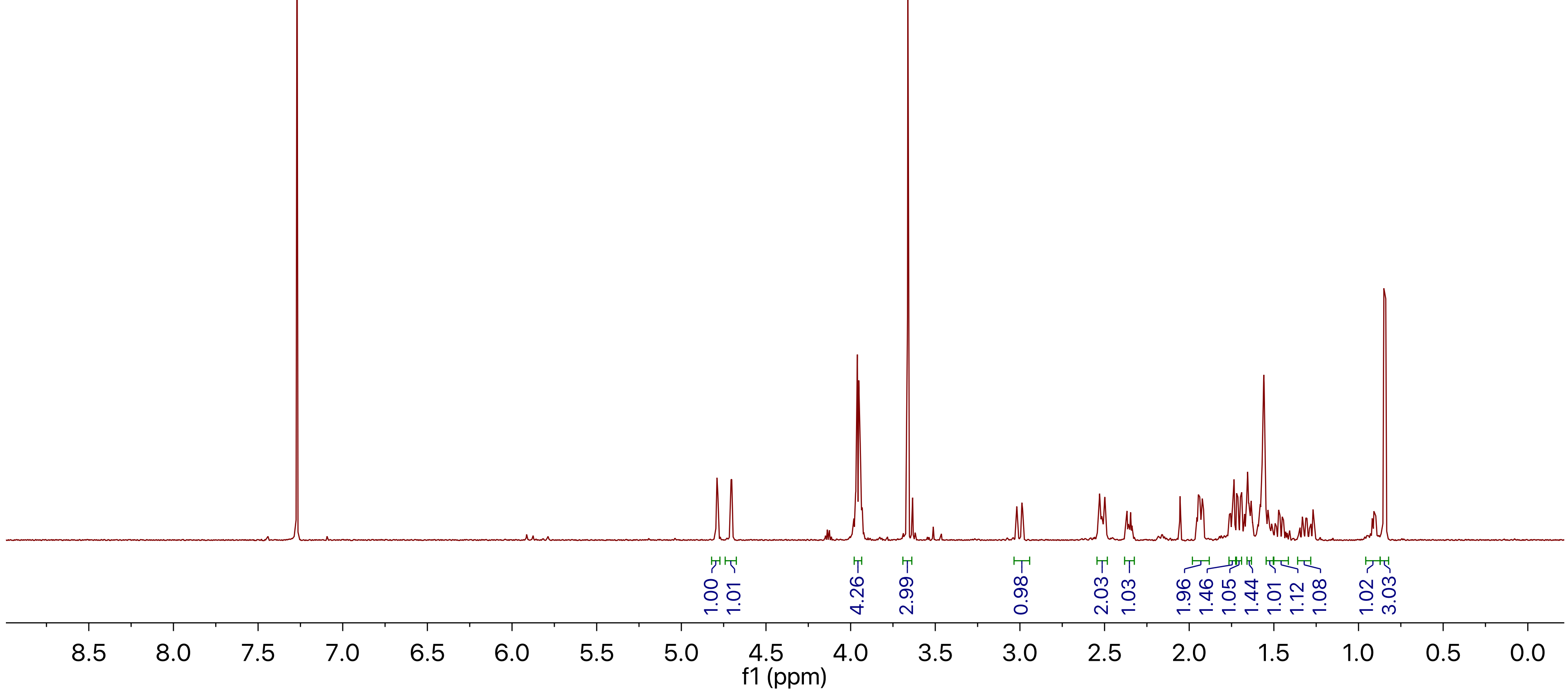
4.79 4.79 4.79 4.78 4.71 4.71 4.70 4.70 3.97 3.97 3.97 3.96 3.96 3.95 3.95 3.94 3.94 3.93 3.66 3.02 3.01 2.99 2.98 2.53 2.53 2.52 2.51 2.50 2.37 2.35 1.95 1.95 1.94 1.93 1.93 1.92 1.92 1.76 1.76 1.74 1.74 1.73 1.72 1.71 1.70 1.69 1.69 1.67 1.67 1.66 1.65 1.65 1.65 1.64 1.63 1.53 1.53 1.47 1.46 1.45 1.33 1.32 1.27 0.91 0.91 0.90 0.85 0.84
1.00 1.01 4.26 2.99 0.98 2.03 1.03 1.96 1.46 1.05 1.44 1.01 1.12 1.08 1.02 3.03
8.5 8.0 7.5 7.0 6.5 6.0 5.5 5.0 4.5 4.0 3.5 3.0 2.5 2.0 1.5 1.0 0.5 0.0
f1 (ppm)

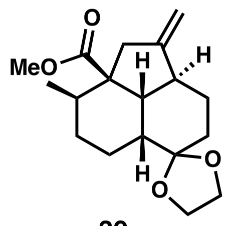

MeO
O
H
H
H
O
O

39

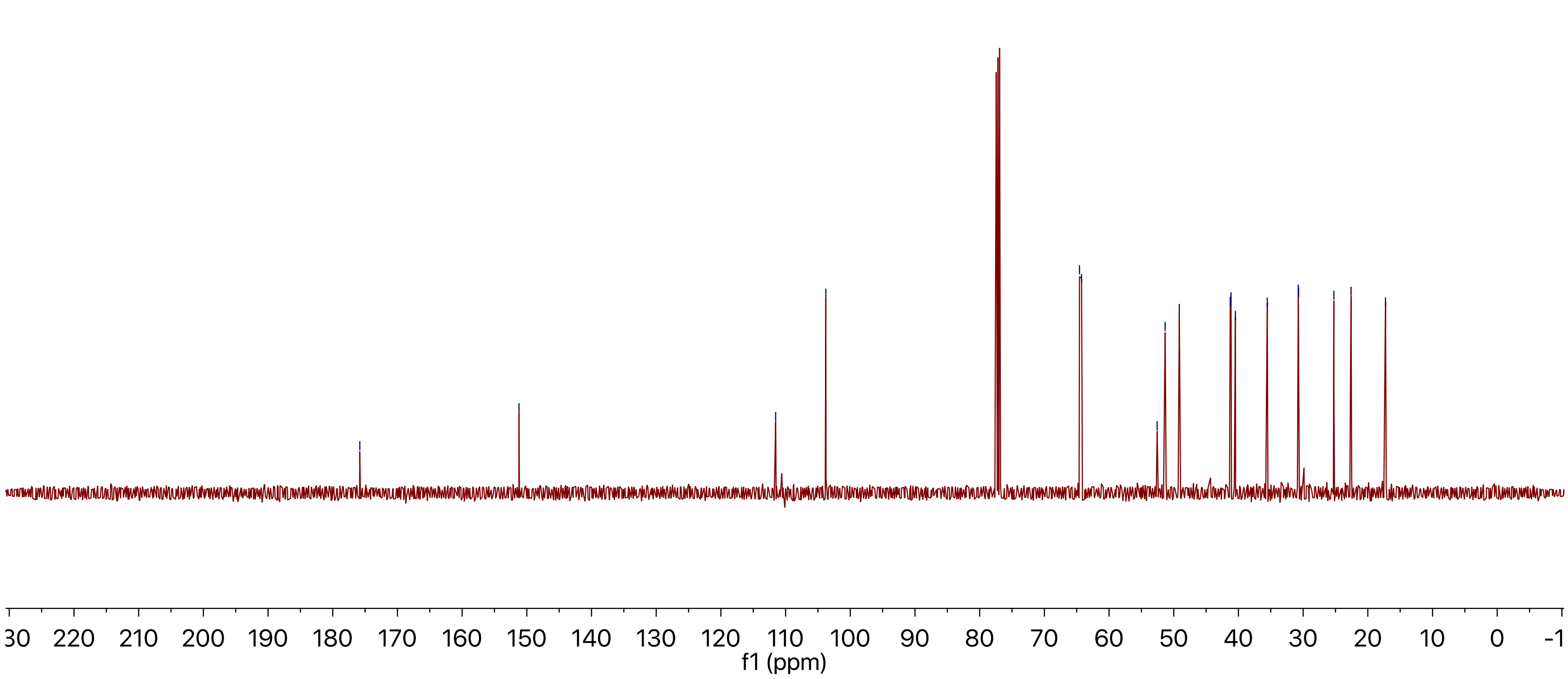

175.81
151.21
111.53
103.78
64.56
64.25
52.57
51.33
49.14
41.27
41.14
40.47
35.55
30.75
30.70
25.24
22.59
17.27
230 220 210 200 190 180 170 160 150 140 130 120 110 100 90 80 70 60 50 40 30 20 10 0 -1
f1 (ppm)

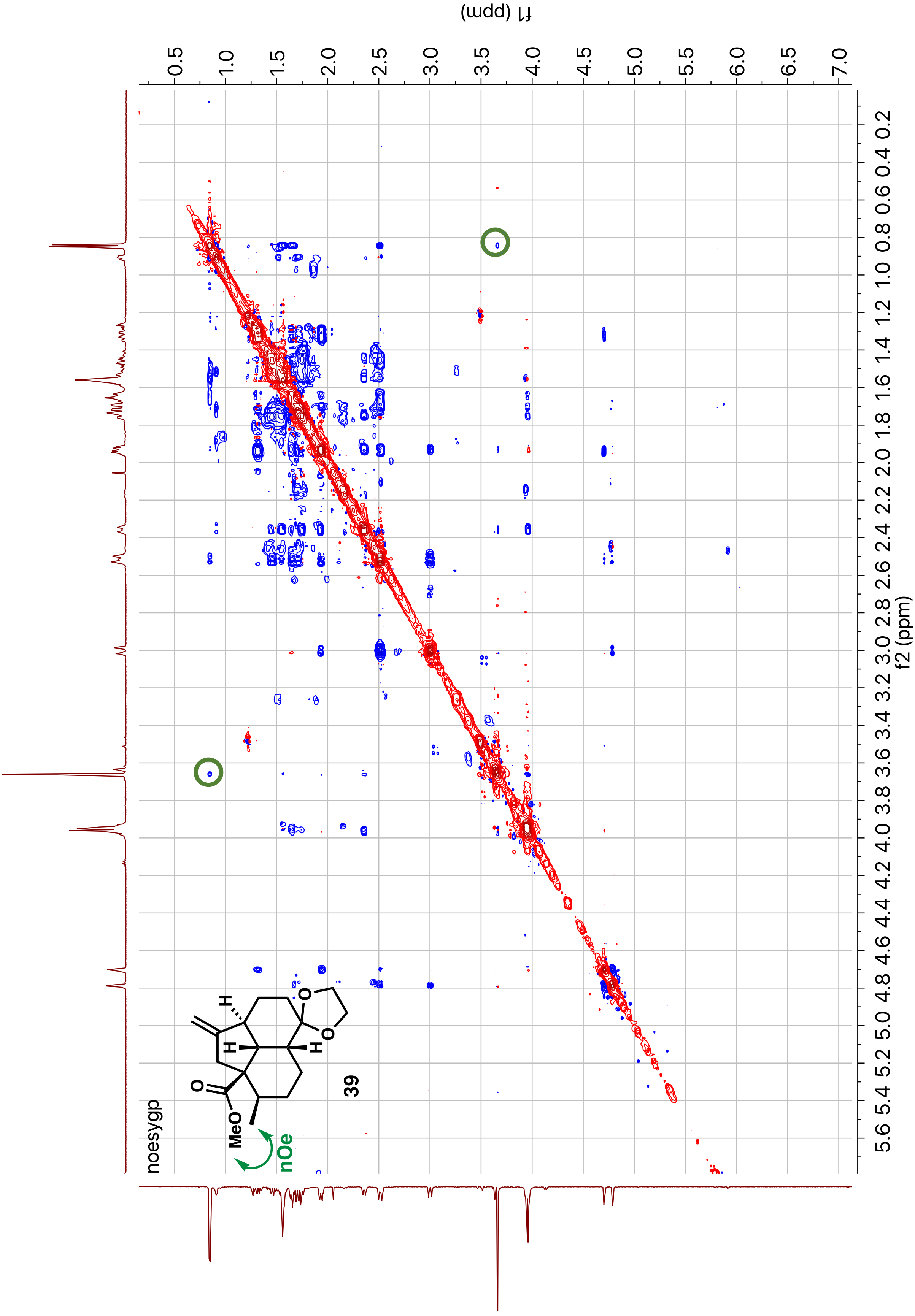
noesygp
nOe
MeO
39
f1 (ppm)
f2 (ppm)

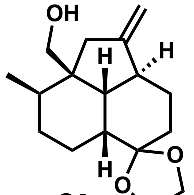
OH
H
H
H
O
O

S6

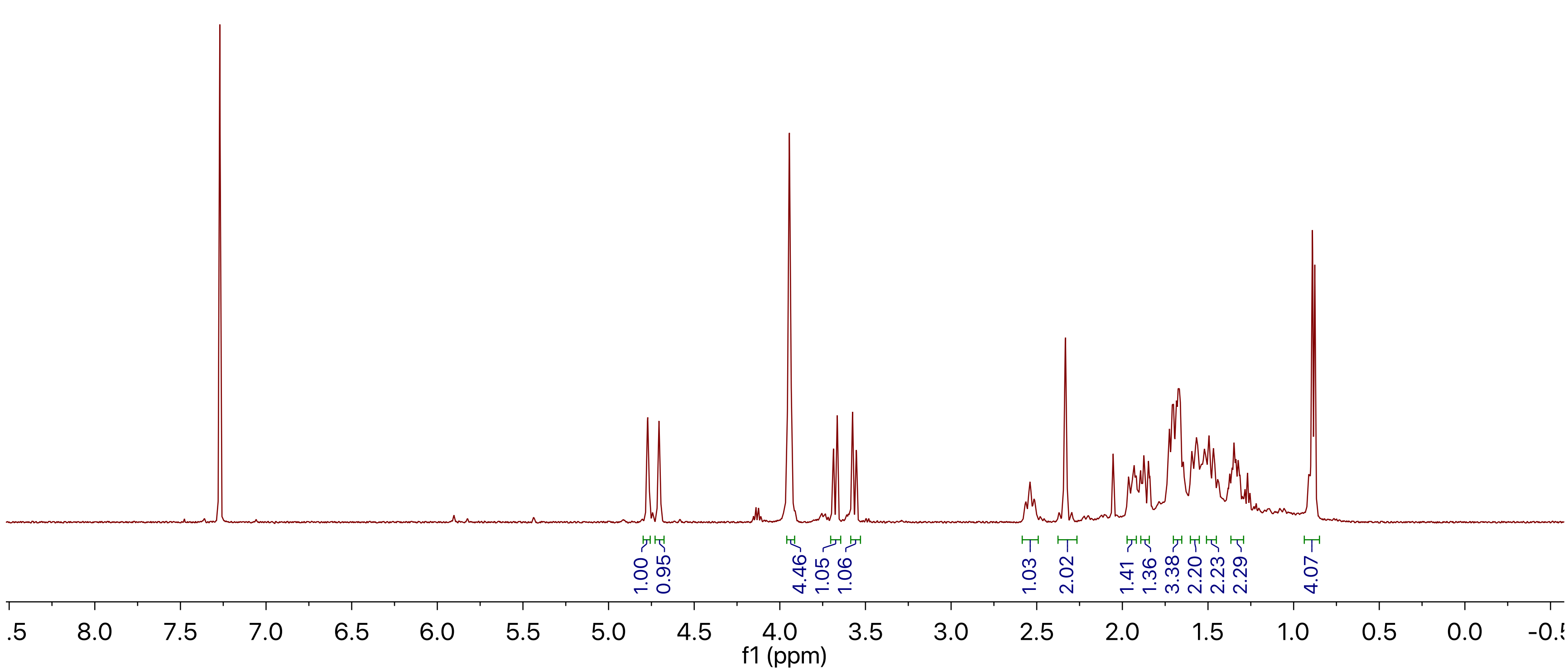
4.77
4.71
4.70
3.95
3.94
3.94
3.69
3.67
3.58
3.55
2.57
2.54
2.51
2.34
2.33
1.96
1.96
1.95
1.94
1.93
1.92
1.91
1.89
1.89
1.87
1.87
1.85
1.84
1.74
1.73
1.71
1.70
1.68
1.67
1.67
1.66
1.64
1.59
1.59
1.57
1.56
1.55
1.52
1.51
1.49
1.49
1.47
1.46
1.44
1.44
1.38
1.37
1.36
1.35
1.34
1.32
1.31
1.30
0.91
0.89
0.88
1.00
0.95
4.46
1.05
1.06
1.03
2.02
1.41
1.36
3.38
2.20
2.23
2.29
4.07
.5
8.0
7.5
7.0
6.5
6.0
5.5
5.0
4.5
4.0
3.5
3.0
2.5
2.0
1.5
1.0
0.5
0.0
-0.5
f1 (ppm)

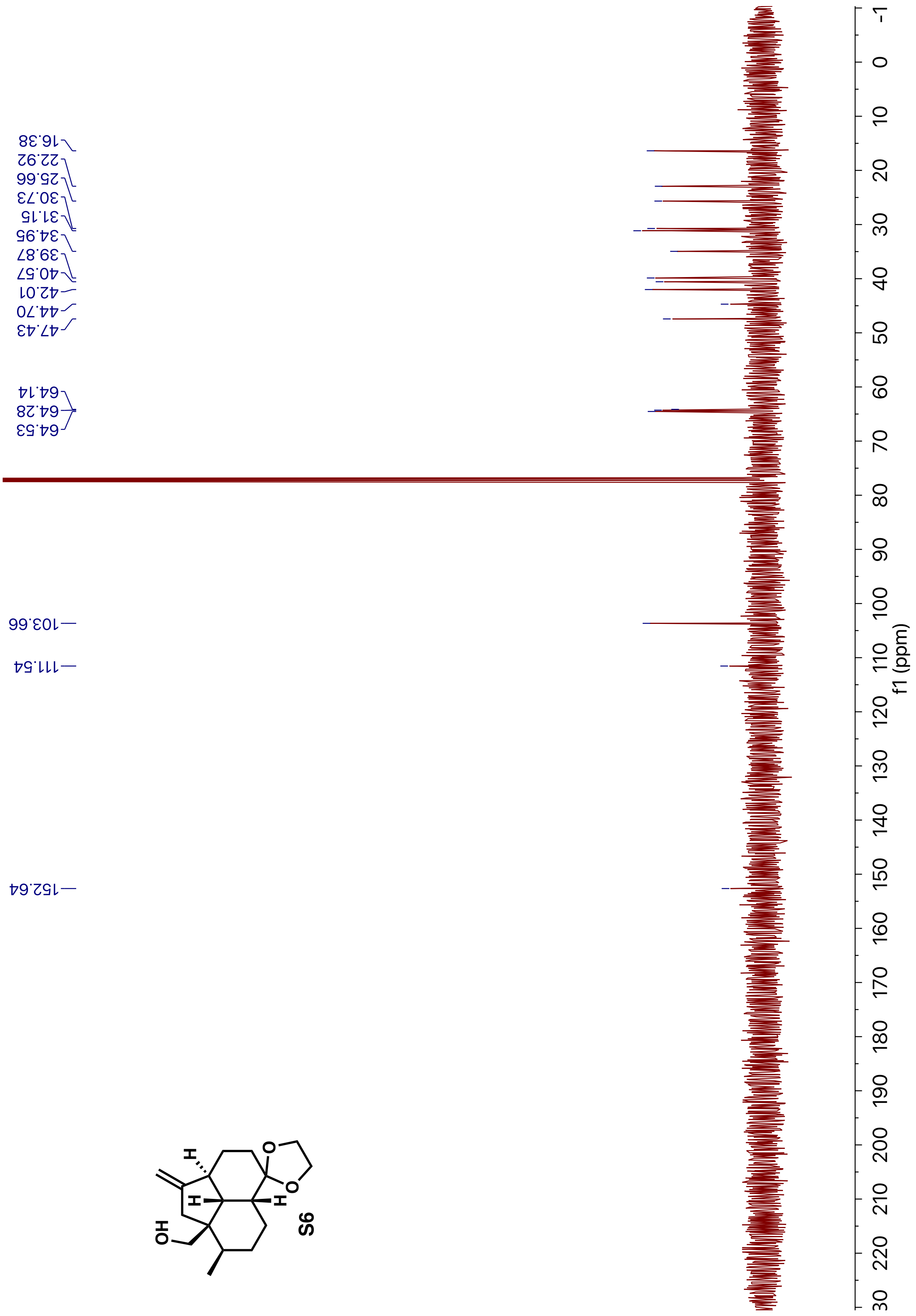

16.38
22.92
25.66
30.73
31.15
34.95
39.87
40.57
42.01
44.70
47.43
64.14
64.28
64.53
103.66
111.54
152.64
f1 (ppm)
OH
H
H
H
O
O

S6

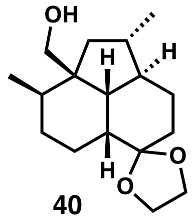

40

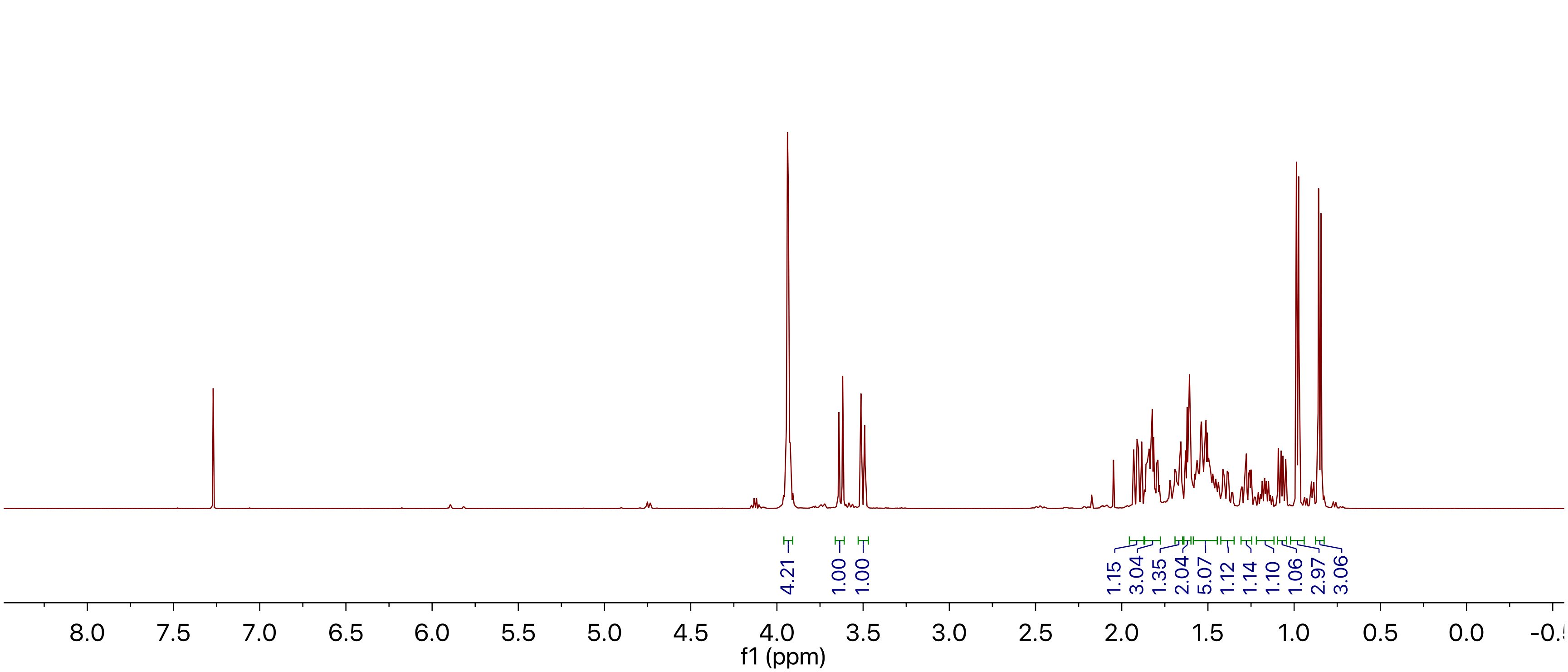
3.95
3.94
3.94
3.93
3.93
3.93
3.92
3.64
3.62
3.51
3.49
1.93
1.91
1.90
1.88
1.86
1.85
1.84
1.84
1.83
1.83
1.82
1.81
1.80
1.79
1.69
1.69
1.68
1.67
1.67
1.66
1.66
1.63
1.62
1.61
1.61
1.61
1.60
1.57
1.56
1.56
1.55
1.54
1.54
1.53
1.52
1.52
1.51
1.50
1.50
1.49
1.49
1.48
1.41
1.39
1.28
1.28
1.26
1.25
1.25
1.09
1.08
1.06
1.05
0.99
0.97
0.86
0.84
4.21
1.00
1.00
1.15
3.04
1.35
2.04
5.07
1.12
1.14
1.10
1.06
2.97
3.06
8.0
7.5
7.0
6.5
6.0
5.5
5.0
4.5
4.0
3.5
3.0
2.5
2.0
1.5
1.0
0.5
0.0
f1 (ppm)

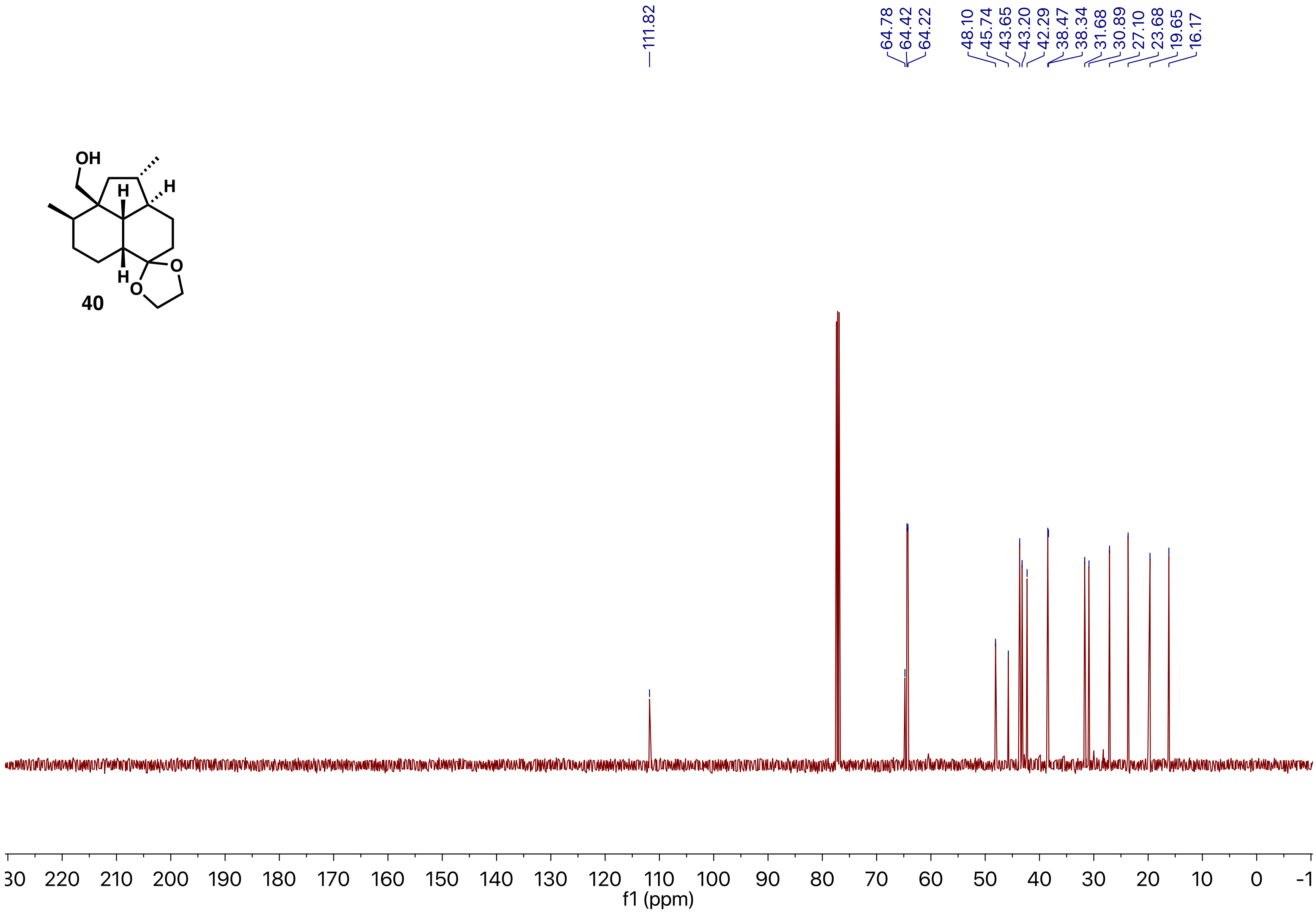

40
111.82
64.78
64.42
64.22
48.10
45.74
43.65
43.20
42.29
38.47
38.34
31.68
30.89
27.10
23.68
19.65
16.17
230 220 210 200 190 180 170 160 150 140 130 120 110 100 90 80 70 60 50 40 30 20 10 0 -1
f1 (ppm)

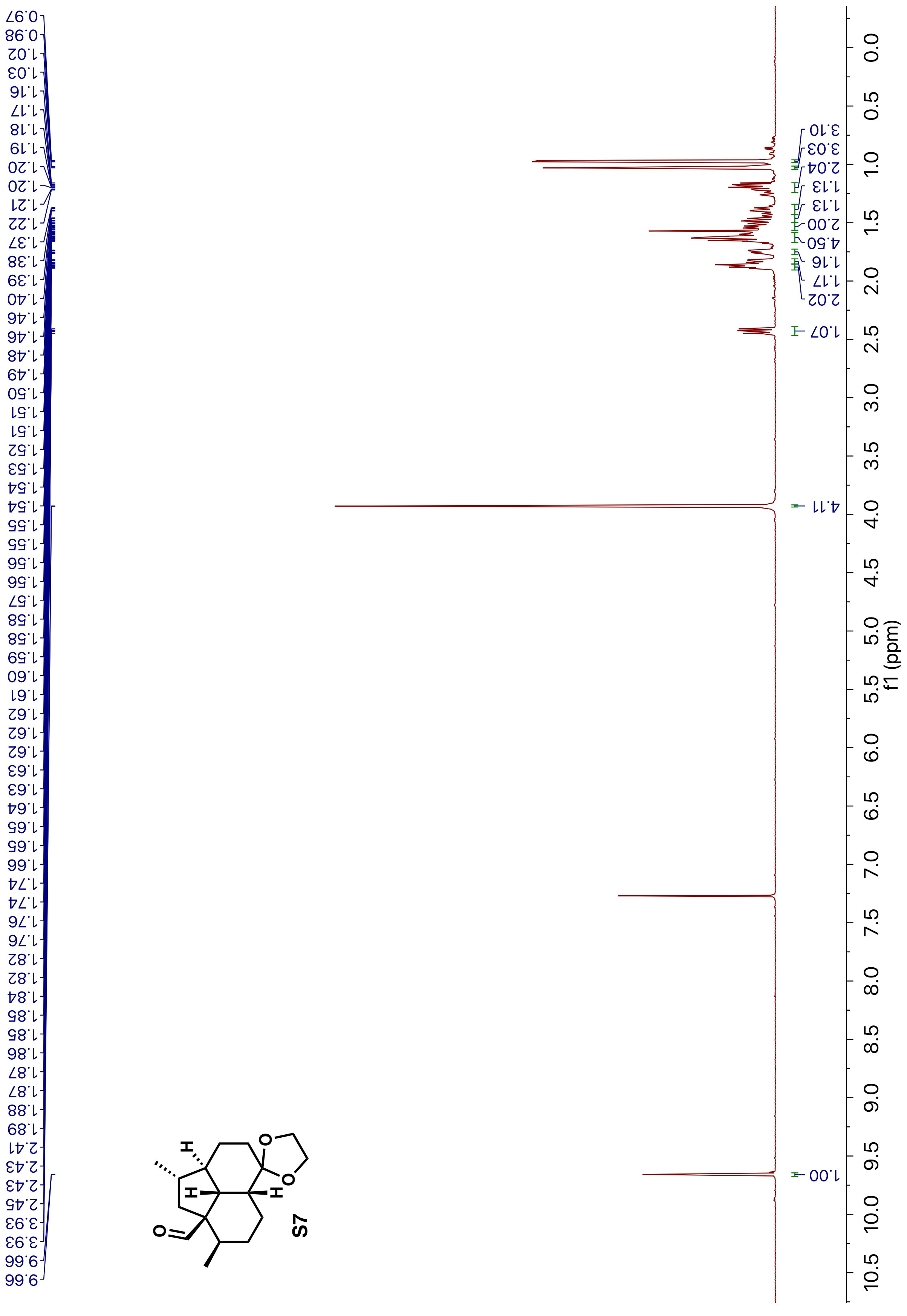
0.97
0.98
1.02
1.03
1.16
1.17
1.18
1.19
1.20
1.20
1.21
1.22
1.37
1.38
1.39
1.40
1.46
1.46
1.48
1.49
1.50
1.51
1.51
1.52
1.53
1.54
1.54
1.55
1.55
1.56
1.56
1.57
1.58
1.58
1.59
1.60
1.61
1.62
1.62
1.62
1.63
1.63
1.64
1.65
1.65
1.66
1.74
1.74
1.76
1.76
1.82
1.82
1.84
1.85
1.85
1.86
1.87
1.87
1.88
1.89
2.41
2.43
2.43
2.45
3.93
3.93
9.66
9.66
S7
3.10
3.03
2.04
1.13
1.13
2.00
4.50
1.16
1.17
2.02
1.07
4.11
1.00
f1 (ppm)

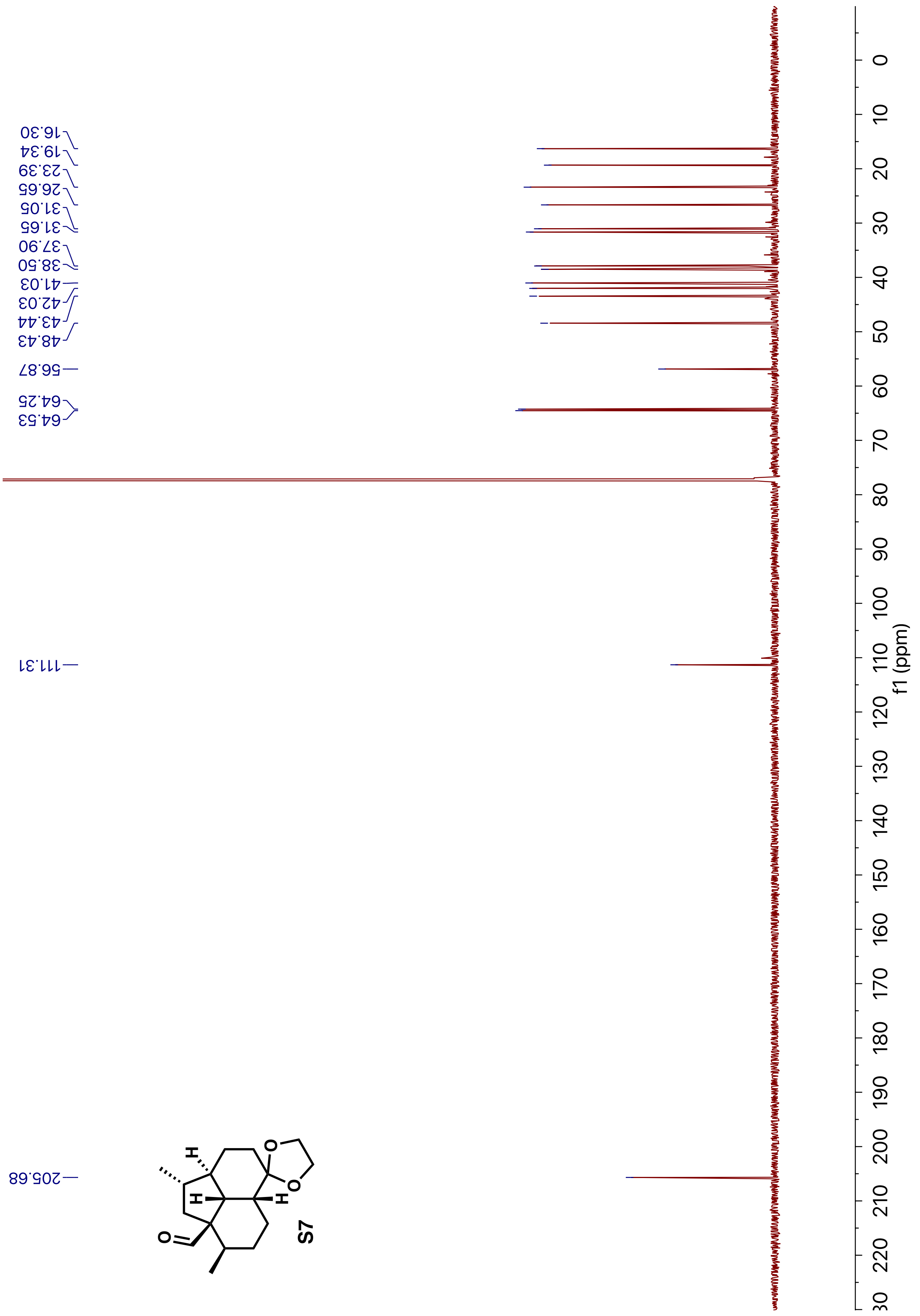
16.30
19.34
23.39
26.65
31.05
31.65
37.90
38.50
41.03
42.03
43.44
48.43
56.87
64.25
64.53
111.31
205.68
S7
f1 (ppm)

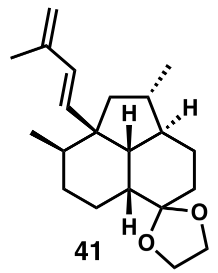

41

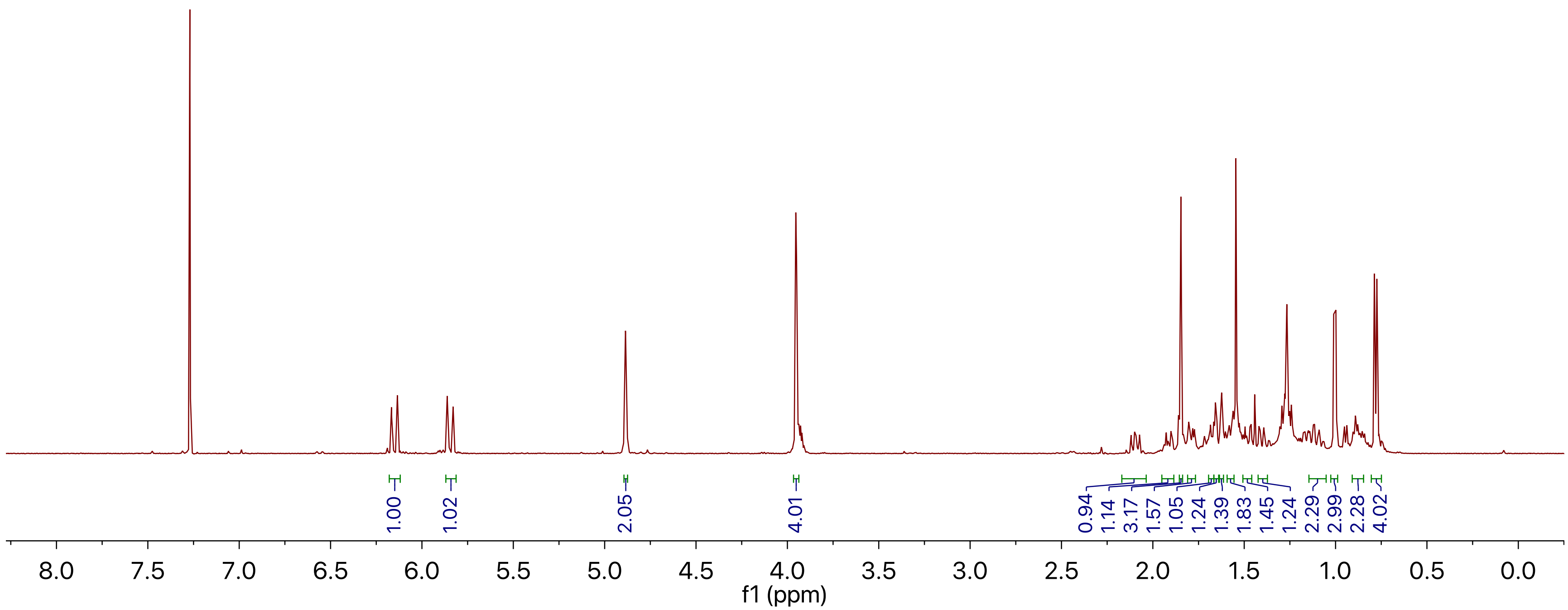
6.17 6.13 5.86 5.83 4.89 3.96 3.95 3.95 1.86 1.86 1.85 1.85 1.85 1.84 1.80 1.80 1.78 1.77 1.68 1.67 1.66 1.66 1.65 1.63 1.63 1.62 1.62 1.62 1.61 1.59 1.58 1.57 1.57 1.56 1.52 1.50 1.49 1.47 1.46 1.44 1.42 1.39 1.39 1.31 1.29 1.28 1.27 1.25 1.24 1.24 1.12 1.12 1.01 1.01 1.00 0.95 0.94 0.89 0.89 0.88 0.88 0.79 0.79 0.78 0.78 0.77 0.77
1.00
1.02
2.05
4.01
0.94 1.14 3.17 1.57 1.05 1.24 1.39 1.83 1.45 1.24 2.29 2.99 2.28 4.02
8.0 7.5 7.0 6.5 6.0 5.5 5.0 4.5 4.0 3.5 3.0 2.5 2.0 1.5 1.0 0.5 0.0
f1 (ppm)

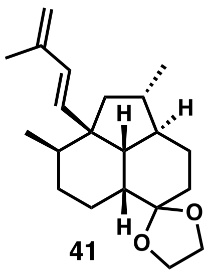

41

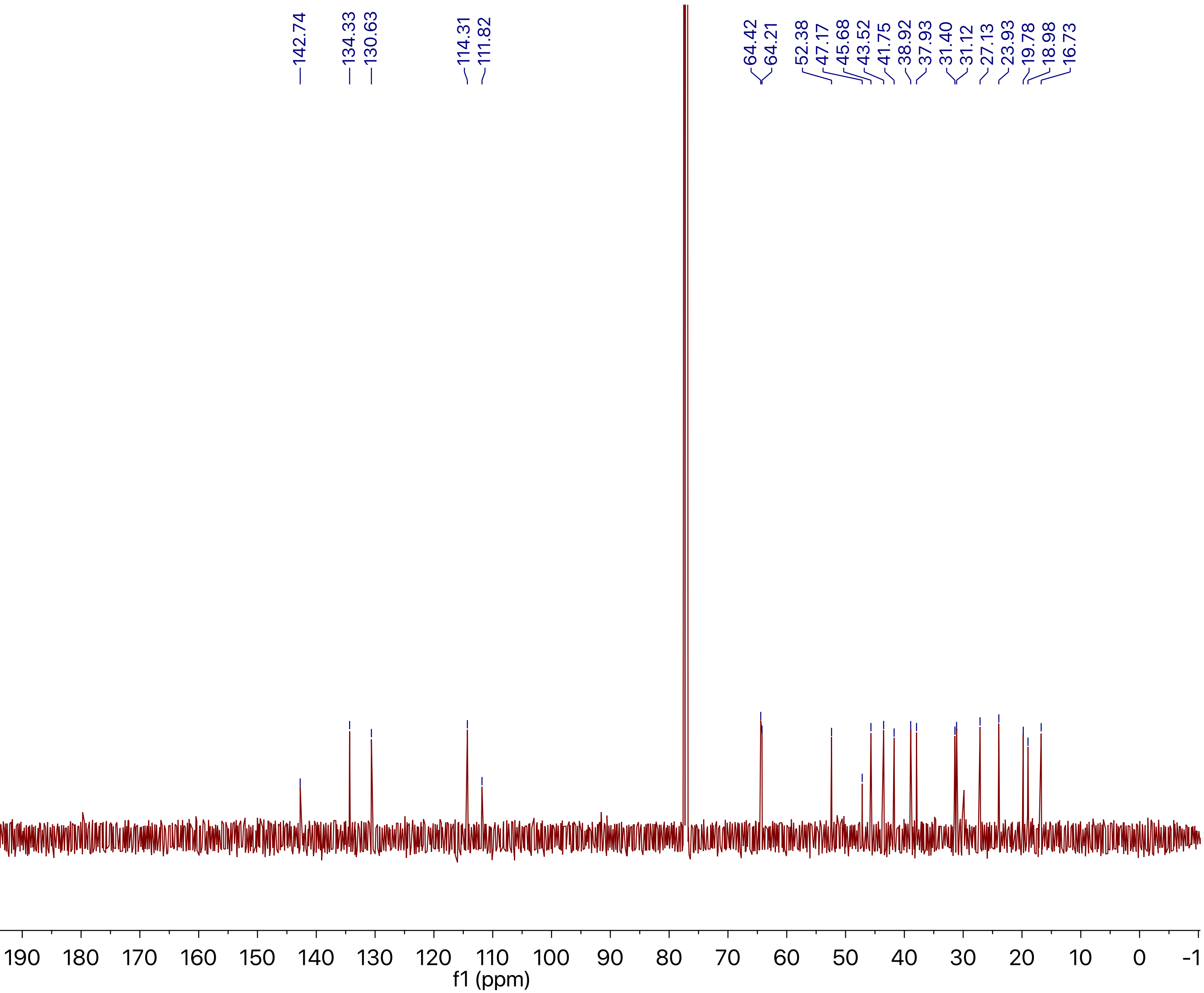

142.74
134.33
130.63
114.31
111.82
64.42
64.21
52.38
47.17
45.68
43.52
41.75
38.92
37.93
31.40
31.12
27.13
23.93
19.78
18.98
16.73
230 220 210 200 190 180 170 160 150 140 130 120 110 100 90 80 70 60 50 40 30 20 10 0 -1
f1 (ppm)

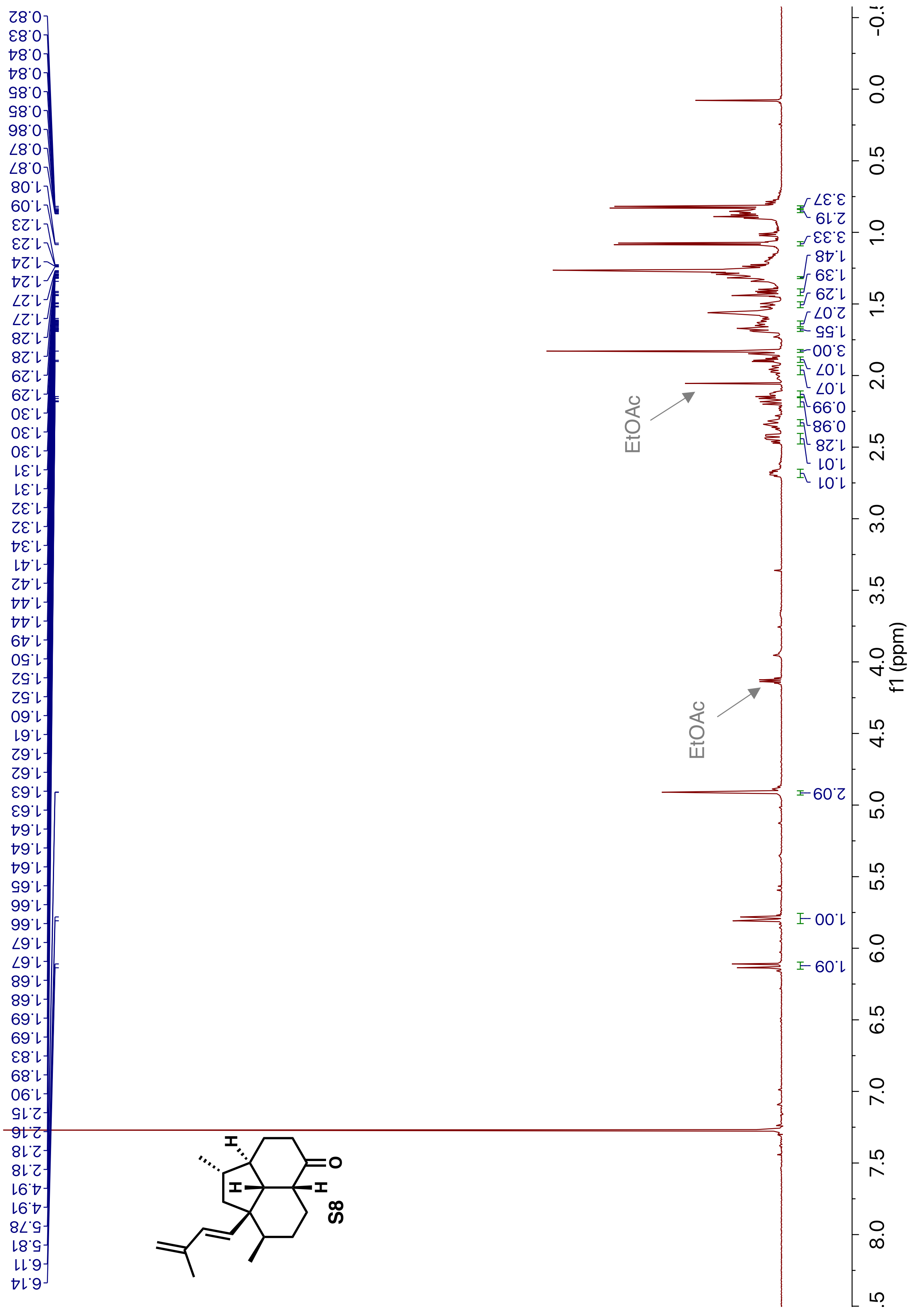
S8
EtOAc
EtOAc
f1 (ppm)

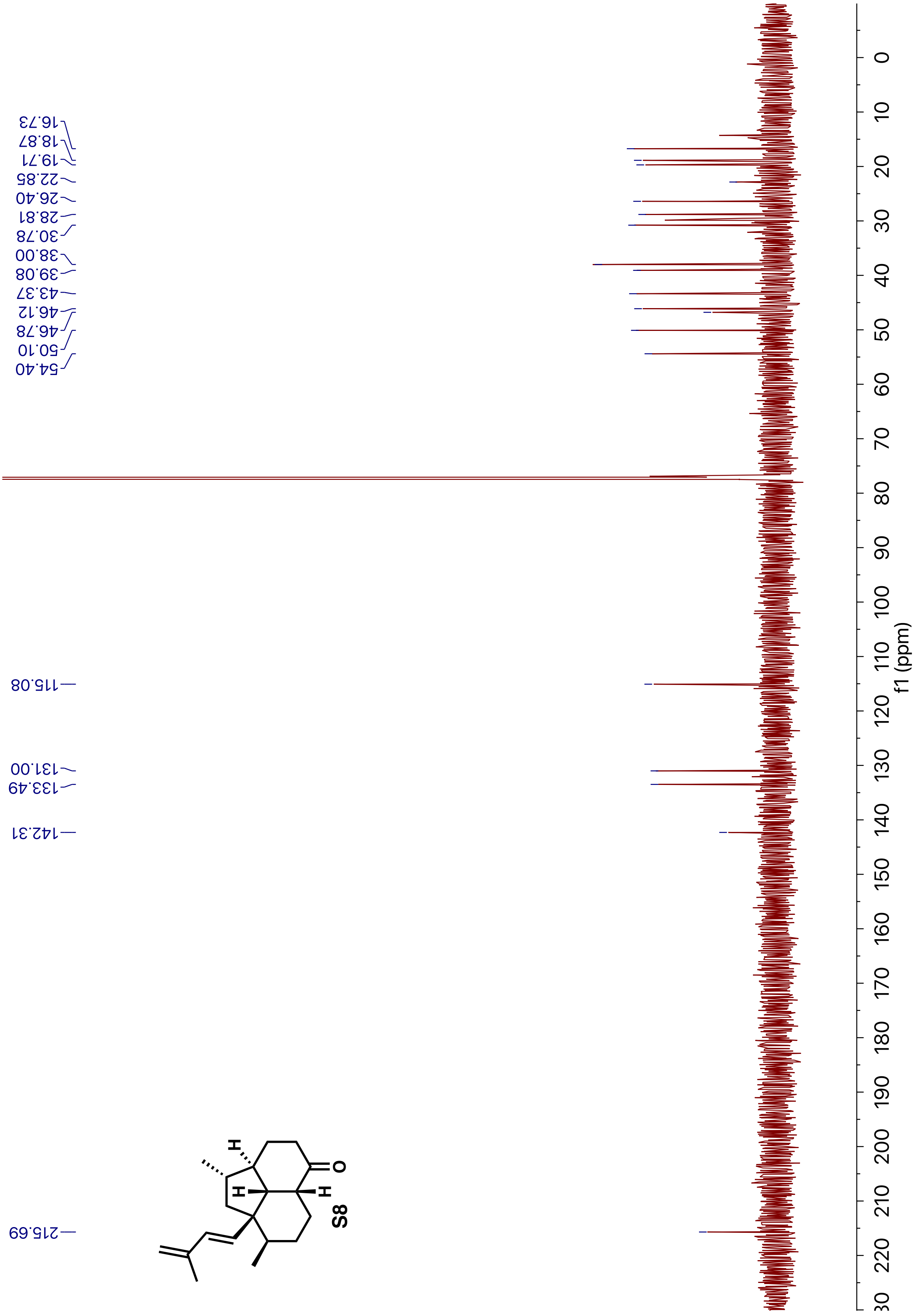
16.73
18.87
19.71
22.85
26.40
28.81
30.78
38.00
39.08
43.37
46.12
46.78
50.10
54.40
115.08
131.00
133.49
142.31
215.69
S8
H
H
H
O
f1 (ppm)

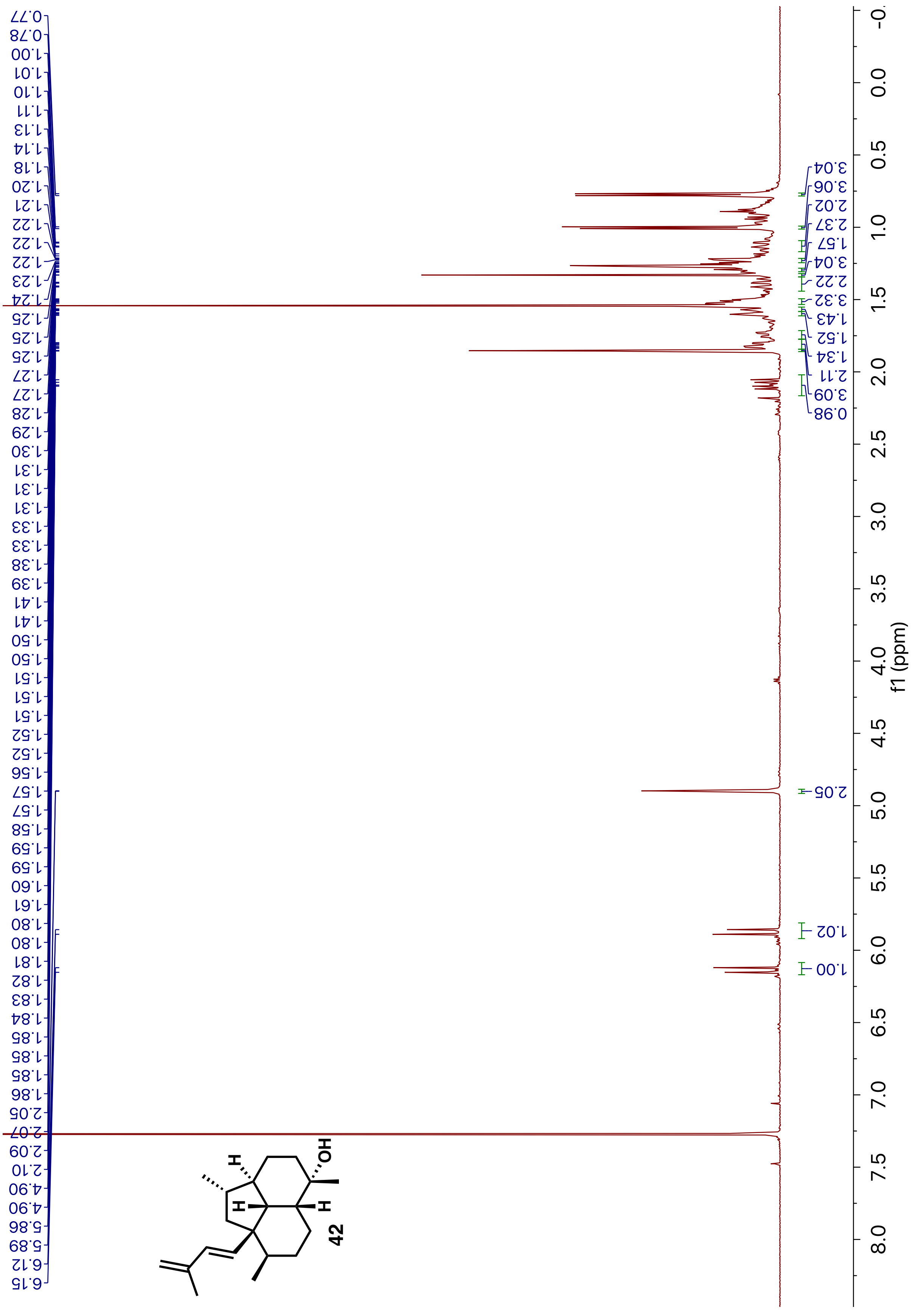

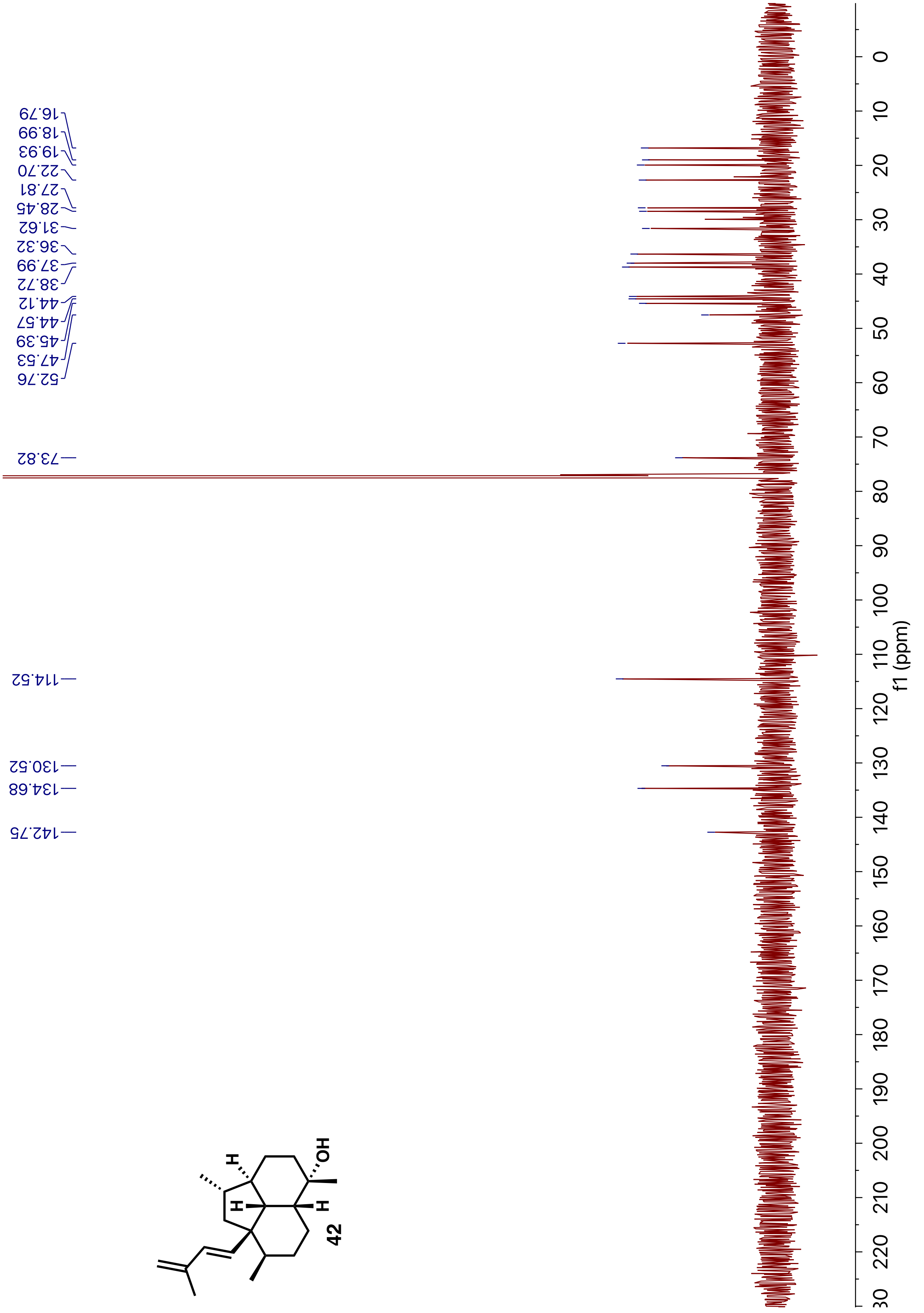

16.79
18.99
19.93
22.70
27.81
28.45
31.62
36.32
37.99
38.72
44.12
44.57
45.39
47.53
52.76
73.82
114.52
130.52
134.68
142.75
f1 (ppm)
42

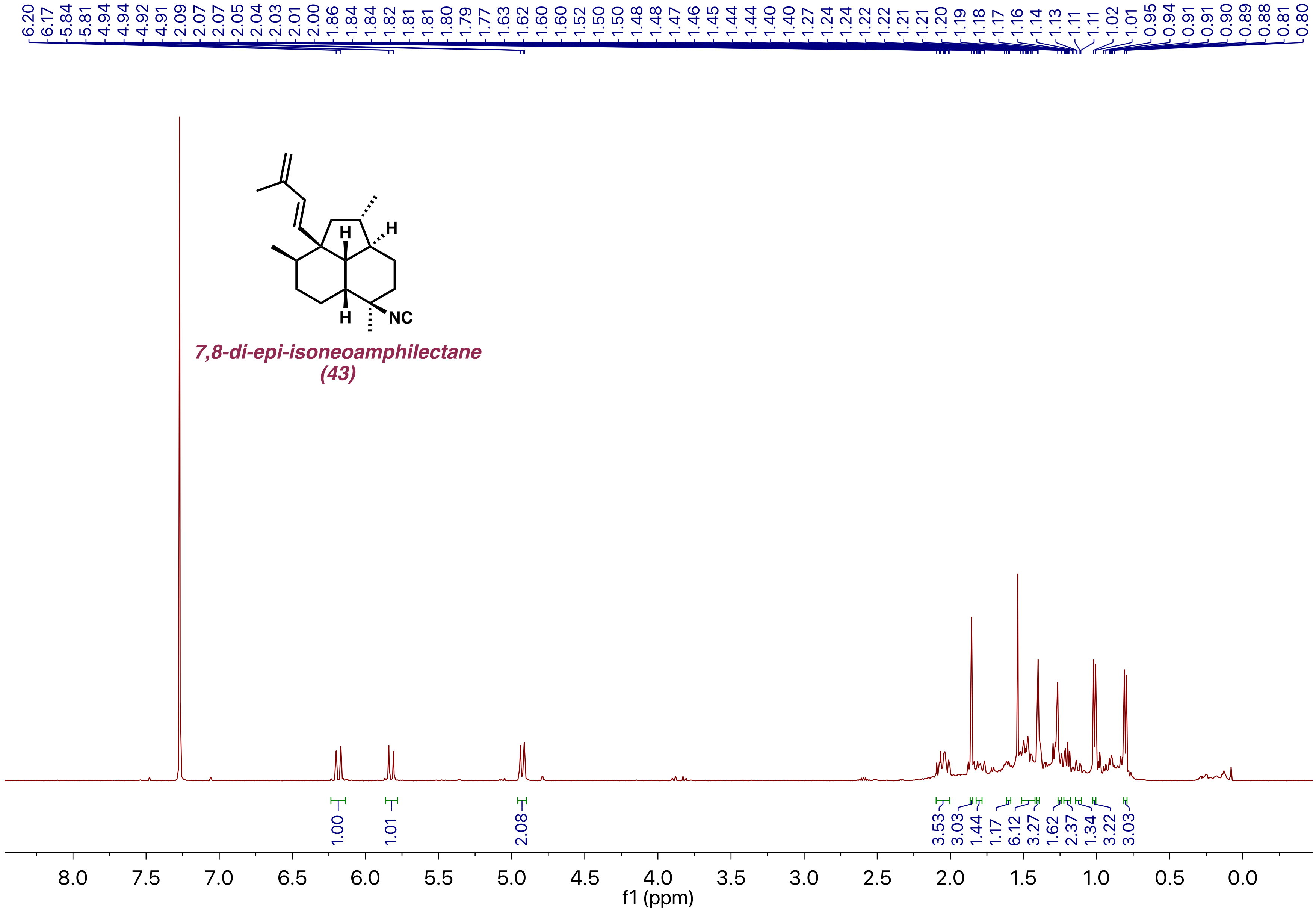
6.20 6.17 5.84 5.81 4.94 4.94 4.92 4.91 2.09 2.07 2.07 2.05 2.04 2.03 2.01 2.00 1.86 1.84 1.84 1.82 1.81 1.81 1.80 1.79 1.77 1.63 1.62 1.60 1.60 1.52 1.50 1.50 1.48 1.48 1.47 1.46 1.45 1.44 1.44 1.40 1.40 1.27 1.24 1.24 1.22 1.22 1.21 1.21 1.20 1.19 1.18 1.17 1.16 1.14 1.13 1.11 1.11 1.02 1.01 0.95 0.94 0.91 0.91 0.90 0.89 0.88 0.81 0.80
H
H
H
NC
7,8-di-epi-isoneoamphilectane
(43)
1.00
1.01
2.08
3.53
3.03
1.44
1.17
6.12
3.27
1.62
2.37
1.34
3.22
3.03
8.0 7.5 7.0 6.5 6.0 5.5 5.0 4.5 4.0 3.5 3.0 2.5 2.0 1.5 1.0 0.5 0.0
f1 (ppm)

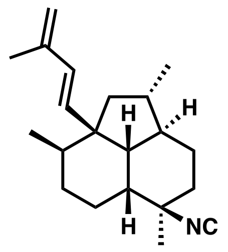

7,8-di-epi-isoneoamphilectane
(43)

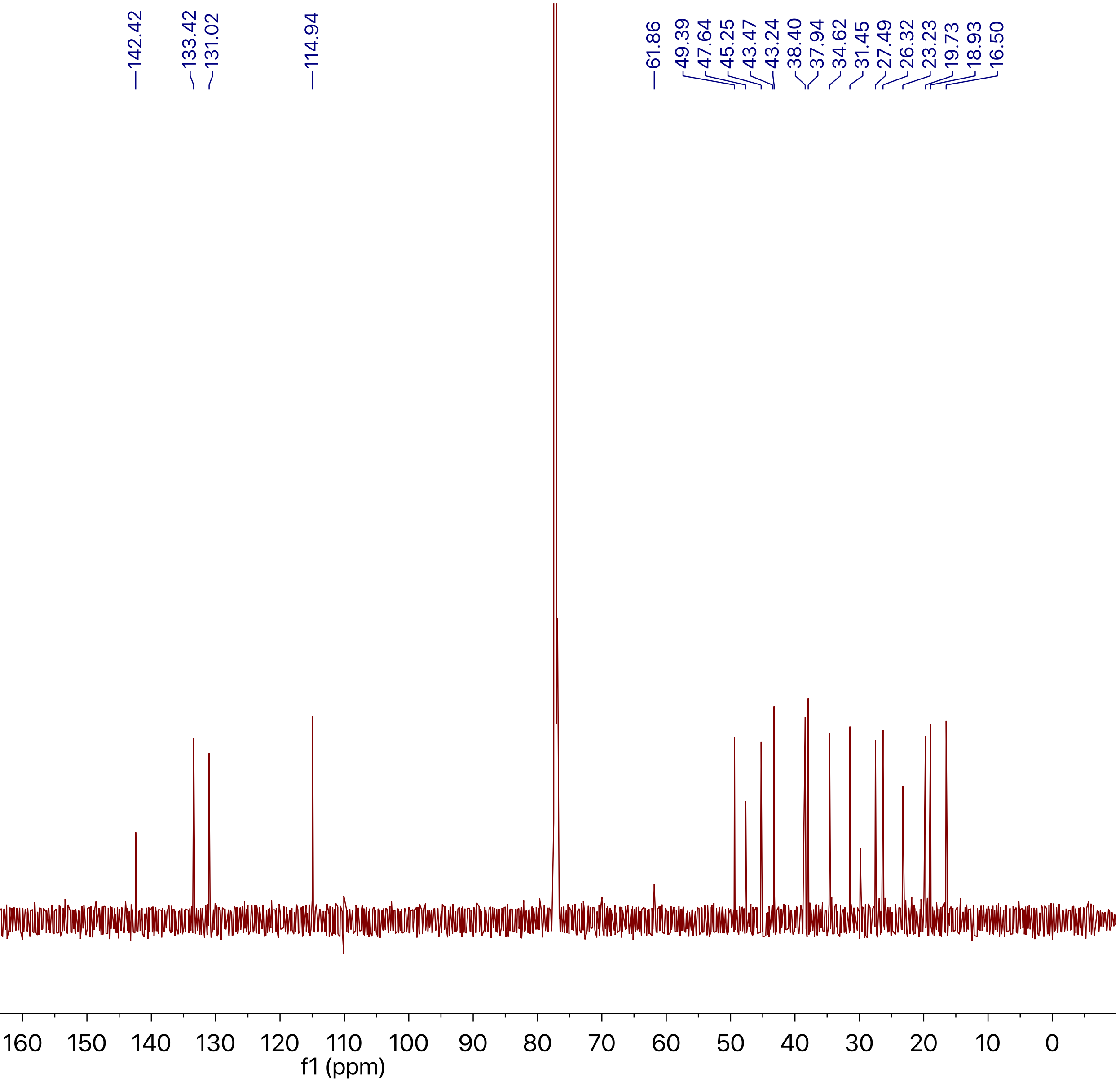

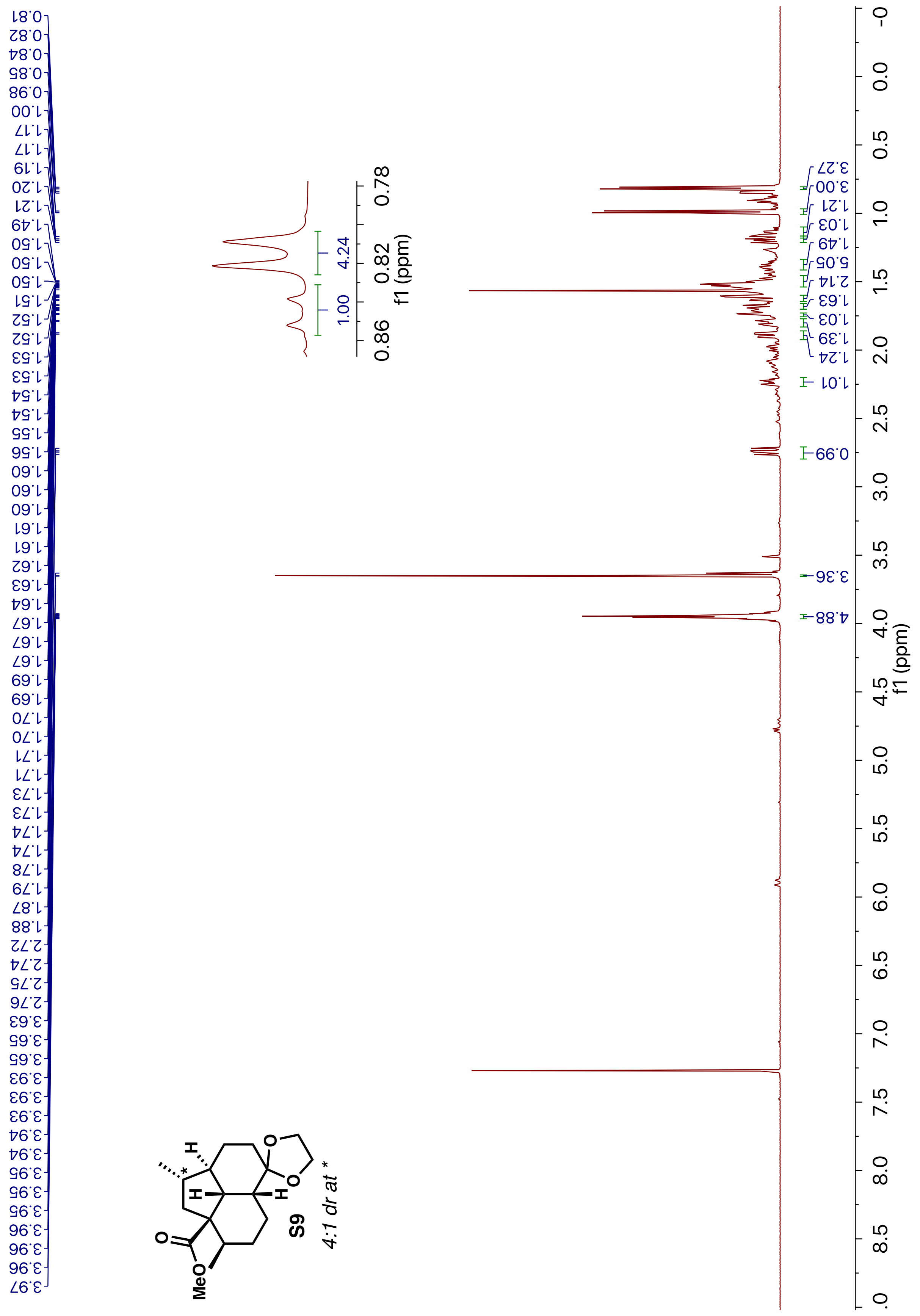
S9
4:1 dr at *
f1 (ppm)

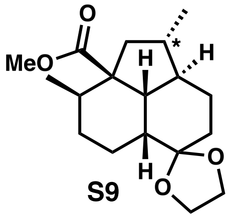

S9

*4:1 dr at **

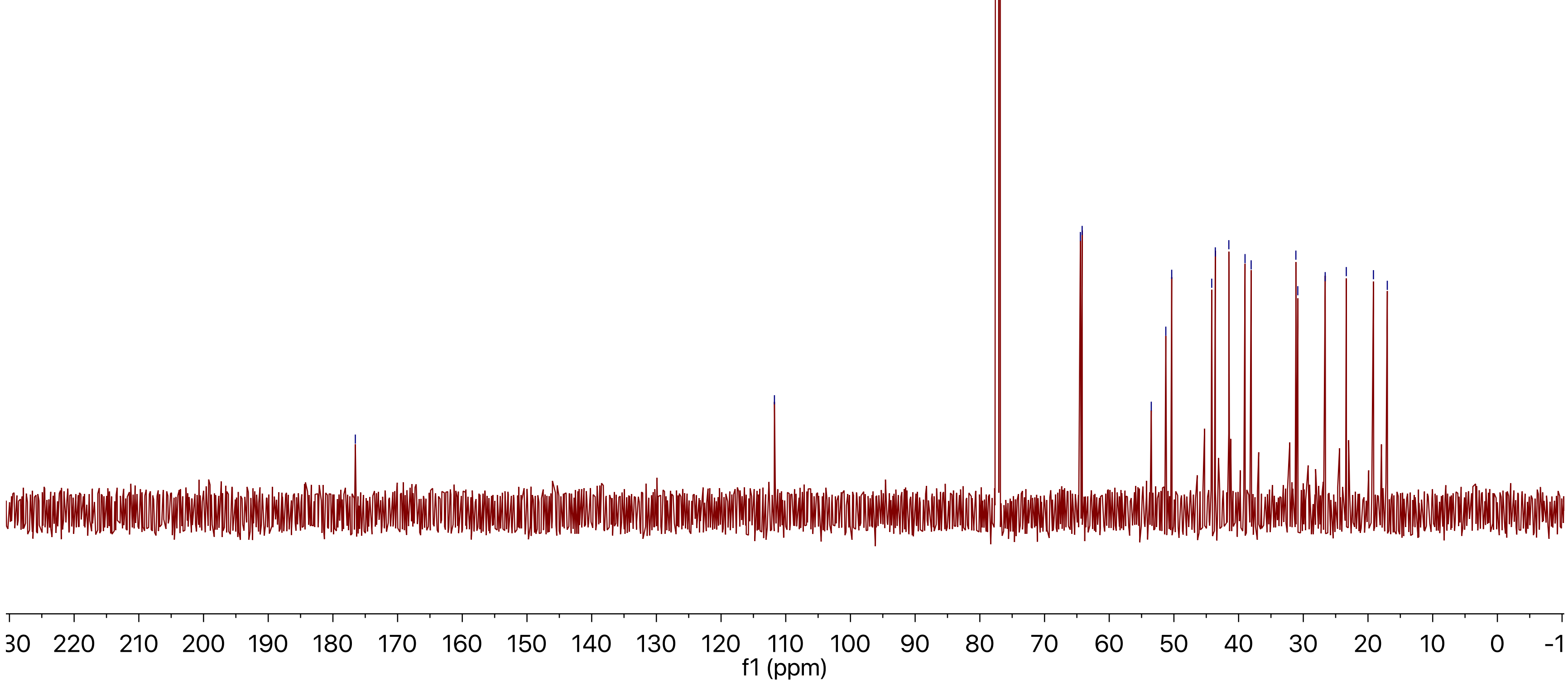
176.53
111.75
64.46
64.19
53.50
51.25
50.34
44.16
43.60
41.51
39.01
38.06
31.15
30.85
26.62
23.35
19.16
17.02
f1 (ppm)

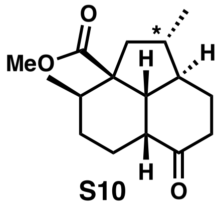
MeO
O
H
H
H
*
O

S10
*4:1 dr at **

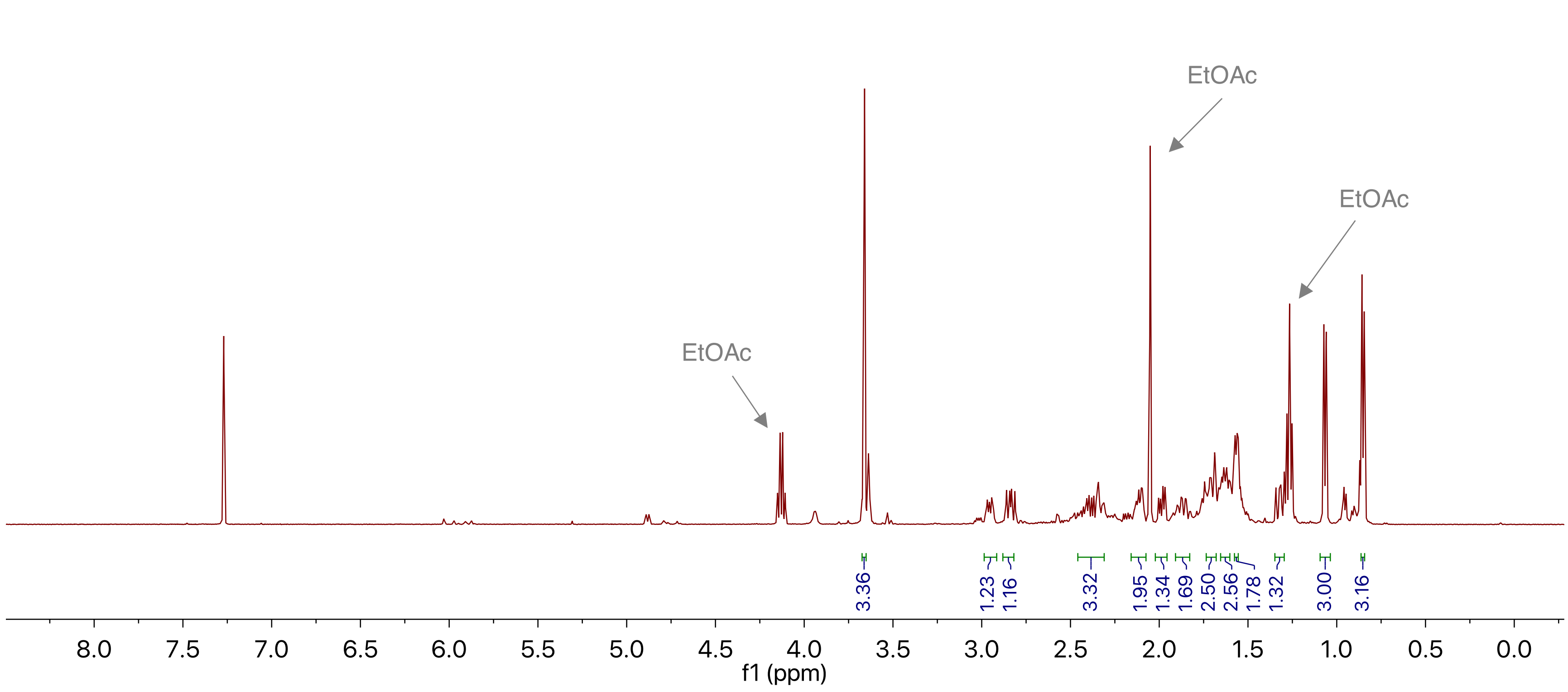
3.66
3.66
2.97
2.96
2.94
2.86
2.84
2.83
2.81
2.41
2.40
2.38
2.37
2.35
2.34
2.32
2.31
2.31
2.13
2.13
2.12
2.12
2.11
2.10
2.10
2.09
2.00
1.99
1.98
1.97
1.90
1.89
1.88
1.87
1.85
1.85
1.76
1.74
1.73
1.73
1.72
1.71
1.69
1.69
1.68
1.66
1.65
1.65
1.64
1.62
1.61
1.61
1.60
1.59
1.58
1.57
1.56
1.55
1.54
1.53
1.34
1.32
1.31
1.30
1.07
1.06
0.86
0.85
EtOAc
EtOAc
EtOAc
3.36
1.23
1.16
3.32
1.95
1.34
1.69
2.50
2.56
1.78
1.32
3.00
3.16
8.0
7.5
7.0
6.5
6.0
5.5
5.0
4.5
4.0
3.5
3.0
2.5
2.0
1.5
1.0
0.5
0.0
f1 (ppm)

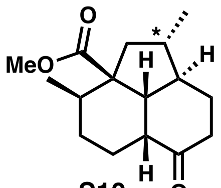

S10

*4:1 dr at **

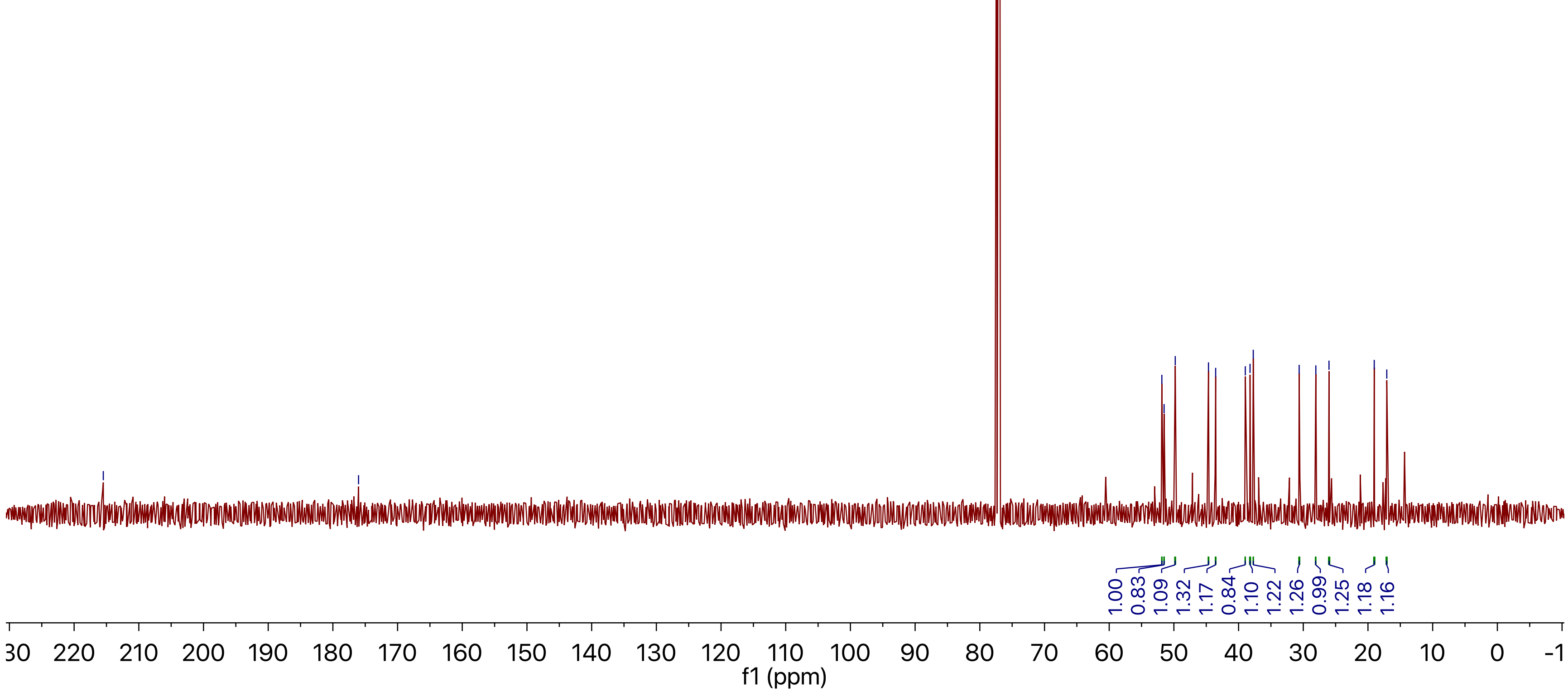
215.49
176.03
51.86
51.51
49.79
44.65
43.54
38.96
38.23
37.73
30.64
28.06
26.02
19.03
17.09
1.00
0.83
1.09
1.32
1.17
0.84
1.10
1.22
1.26
0.99
1.25
1.18
1.16
30
220
210
200
190
180
170
160
150
140
130
120
110
100
90
80
70
60
50
40
30
20
10
0
-1
f1 (ppm)

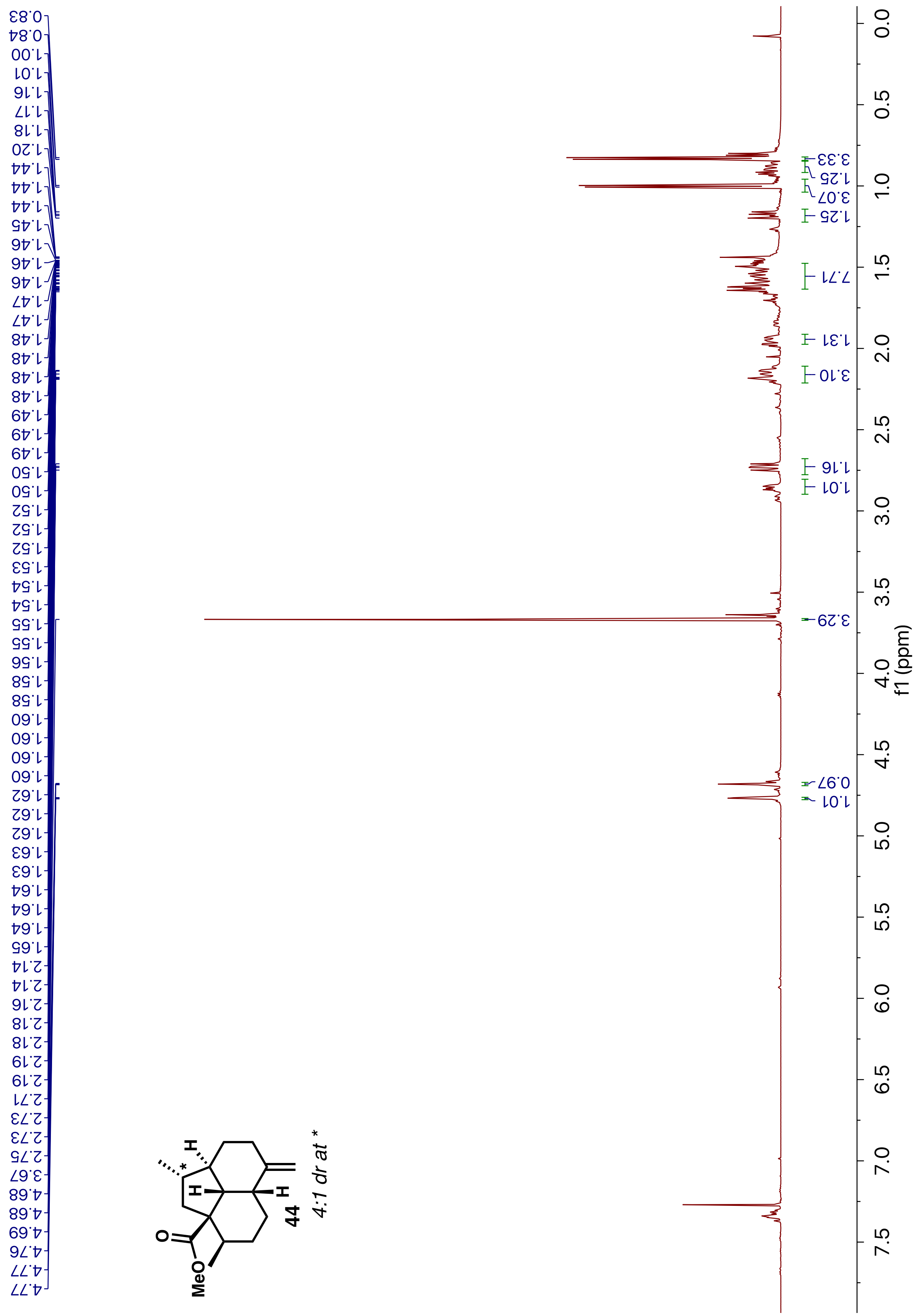
0.83
0.84
1.00
1.01
1.16
1.17
1.18
1.20
1.44
1.44
1.44
1.45
1.46
1.46
1.46
1.47
1.47
1.48
1.48
1.48
1.48
1.49
1.49
1.49
1.50
1.50
1.52
1.52
1.52
1.53
1.54
1.54
1.55
1.55
1.56
1.58
1.58
1.60
1.60
1.60
1.60
1.62
1.62
1.62
1.63
1.63
1.64
1.64
1.64
1.65
2.14
2.14
2.16
2.18
2.18
2.19
2.19
2.71
2.73
2.73
2.75
3.67
4.68
4.68
4.69
4.76
4.77
4.77
MeO
O
H
H
H
44
4:1 dr at *
3.33
1.25
3.07
1.25
7.71
1.31
3.10
1.16
1.01
3.29
0.97
1.01
f1 (ppm)

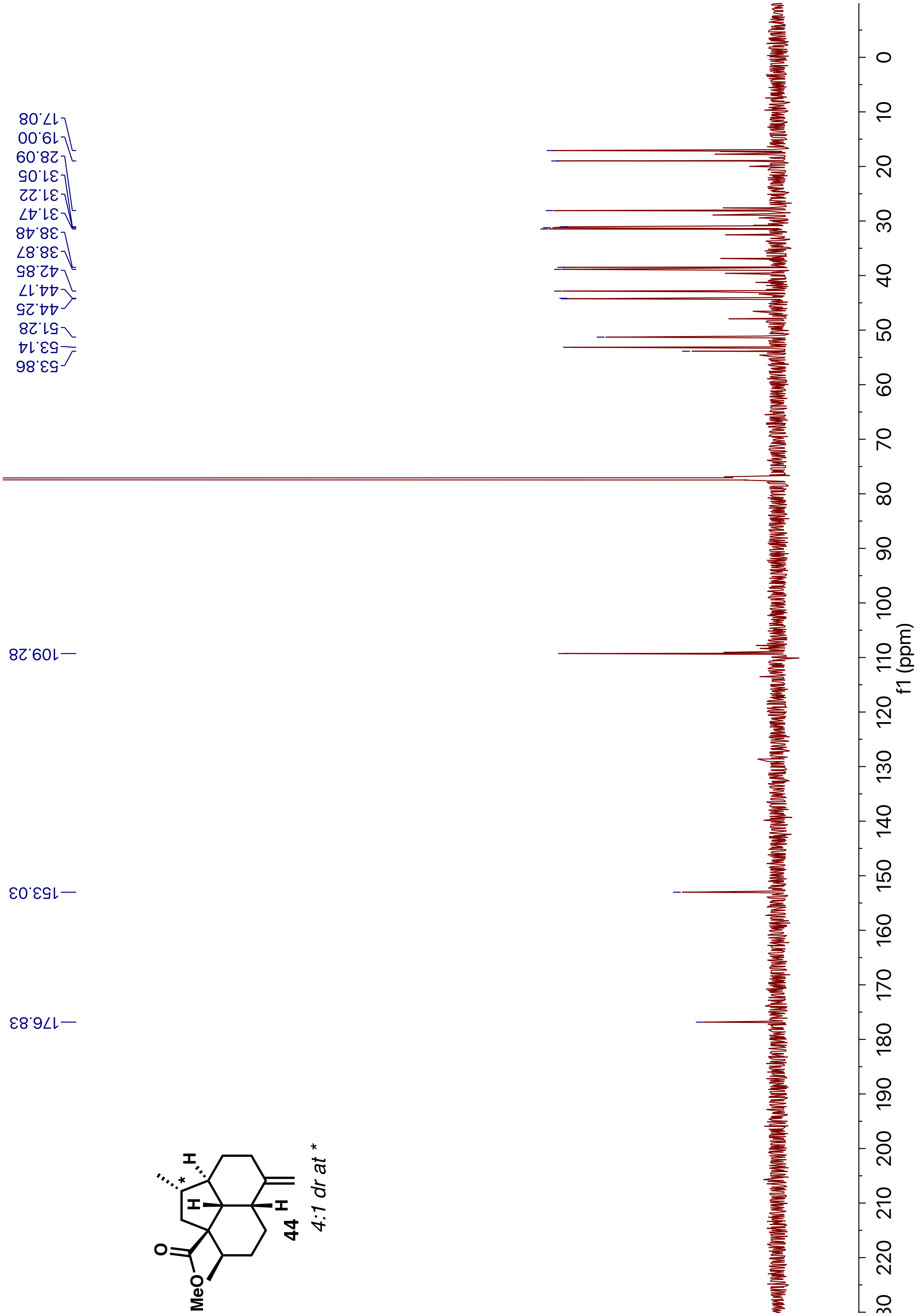
17.08
19.00
28.09
31.05
31.22
31.47
38.48
38.87
42.85
44.17
44.25
51.28
53.14
53.86
109.28
153.03
176.83
f1 (ppm)
MeO
O
H
44
4:1 dr at *

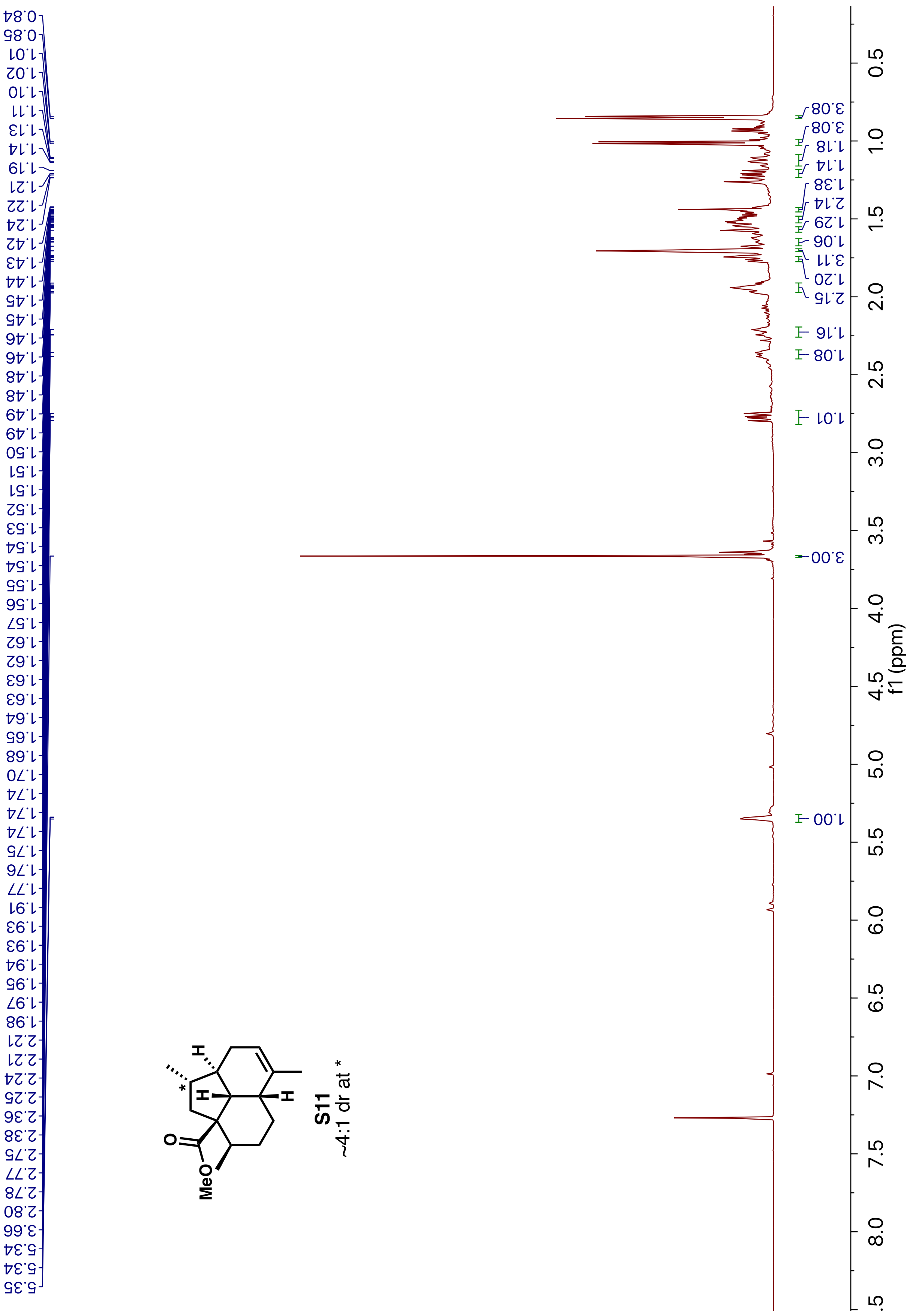

0.84
0.85
1.01
1.02
1.10
1.11
1.13
1.14
1.19
1.21
1.22
1.24
1.42
1.43
1.44
1.45
1.45
1.46
1.46
1.48
1.48
1.49
1.49
1.50
1.51
1.51
1.52
1.53
1.54
1.54
1.55
1.56
1.57
1.62
1.62
1.63
1.63
1.64
1.65
1.68
1.70
1.74
1.74
1.74
1.75
1.76
1.77
1.91
1.93
1.93
1.94
1.95
1.97
1.98
2.21
2.21
2.24
2.25
2.36
2.38
2.75
2.77
2.78
2.80
3.66
5.34
5.34
5.35
MeO
O
H
H
H
*
S11
~4:1 dr at *
3.08
3.08
1.18
1.14
1.38
2.14
1.29
1.06
3.11
1.20
2.15
1.16
1.08
1.01
3.00
1.00
0.5
1.0
1.5
2.0
2.5
3.0
3.5
4.0
4.5
5.0
5.5
6.0
6.5
7.0
7.5
8.0
f1 (ppm)

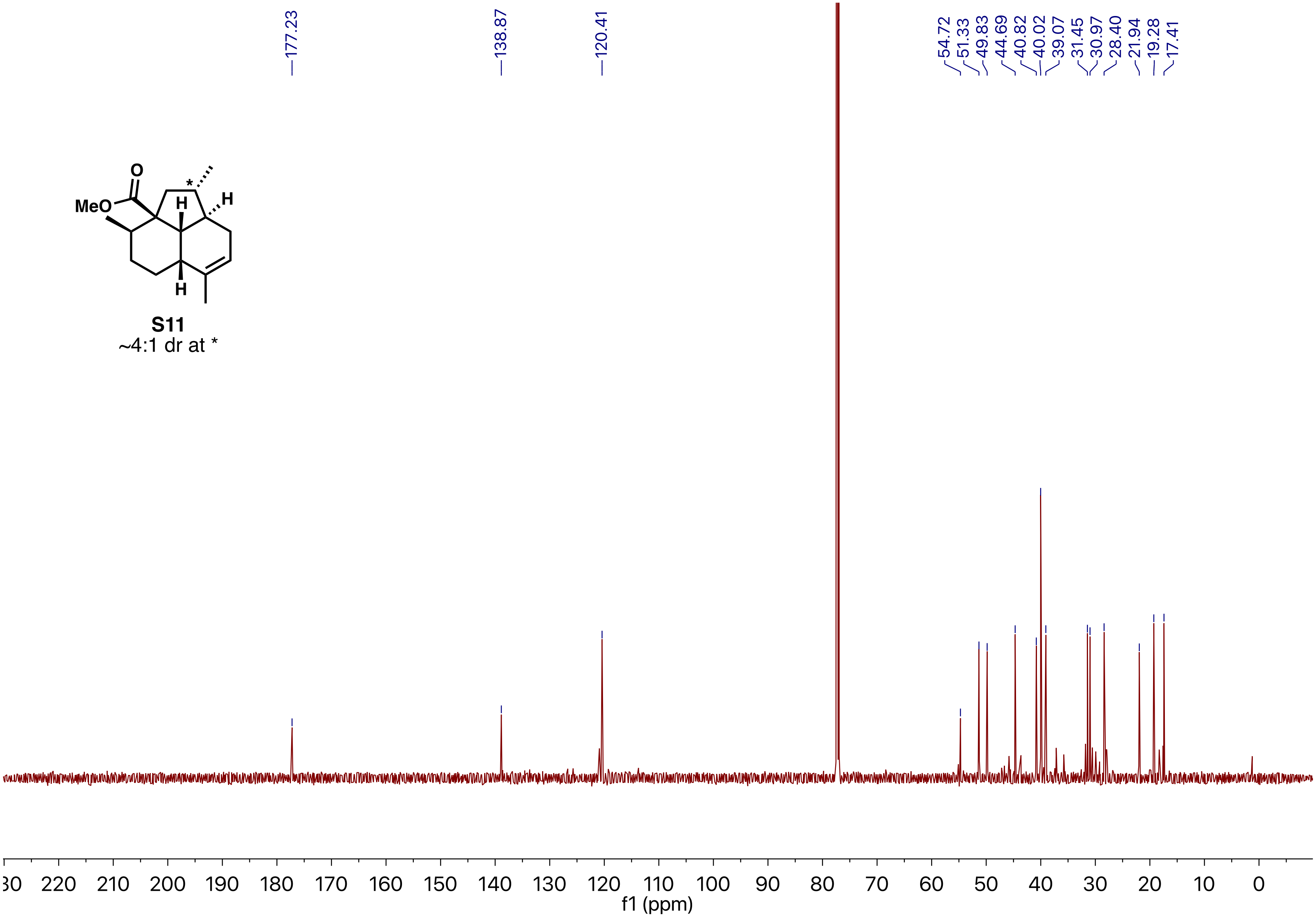
177.23
138.87
120.41
54.72
51.33
49.83
44.69
40.82
40.02
39.07
31.45
30.97
28.40
21.94
19.28
17.41
MeO
O
H
H
H
230 220 210 200 190 180 170 160 150 140 130 120 110 100 90 80 70 60 50 40 30 20 10 0
f1 (ppm)

S11
~4:1 dr at *

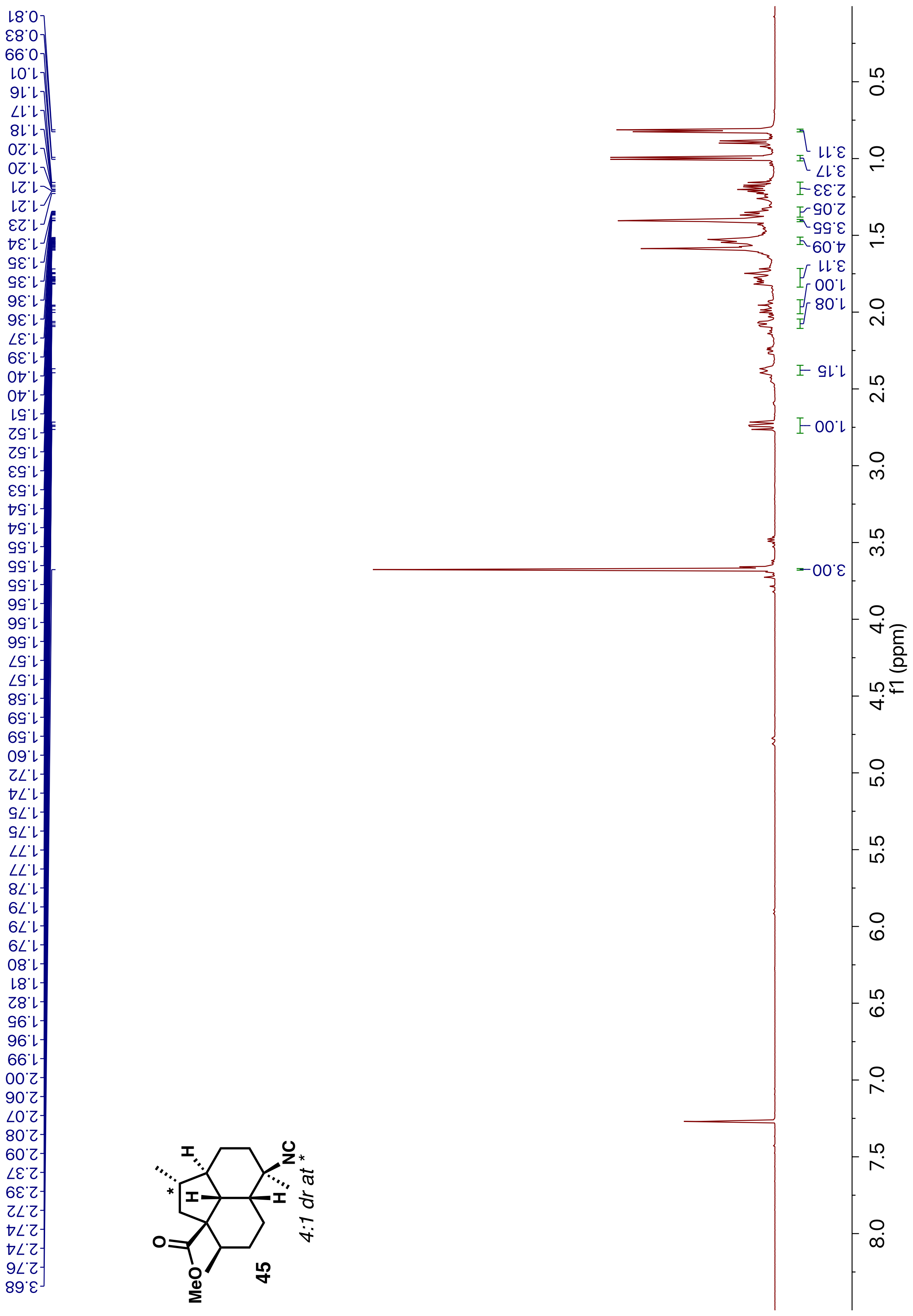

0.81
0.83
0.99
1.01
1.16
1.17
1.18
1.20
1.20
1.21
1.21
1.23
1.34
1.35
1.35
1.36
1.36
1.37
1.39
1.40
1.40
1.51
1.52
1.52
1.53
1.53
1.54
1.54
1.55
1.55
1.55
1.56
1.56
1.56
1.57
1.57
1.58
1.59
1.59
1.60
1.72
1.74
1.75
1.75
1.77
1.77
1.78
1.79
1.79
1.79
1.80
1.81
1.82
1.95
1.96
1.99
2.00
2.06
2.07
2.08
2.09
2.37
2.39
2.72
2.74
2.74
2.76
3.68
MeO
O
H
H
H
NC
*
45
4:1 dr at *
3.11
3.17
2.33
2.05
3.55
4.09
3.11
1.00
1.08
1.15
1.00
3.00
0.5
1.0
1.5
2.0
2.5
3.0
3.5
4.0
4.5
5.0
5.5
6.0
6.5
7.0
7.5
8.0
f1 (ppm)

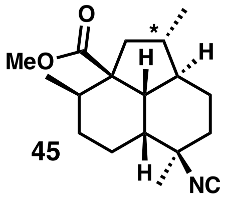
O
MeO
H
H
H
NC
*

45

*4:1 dr at **

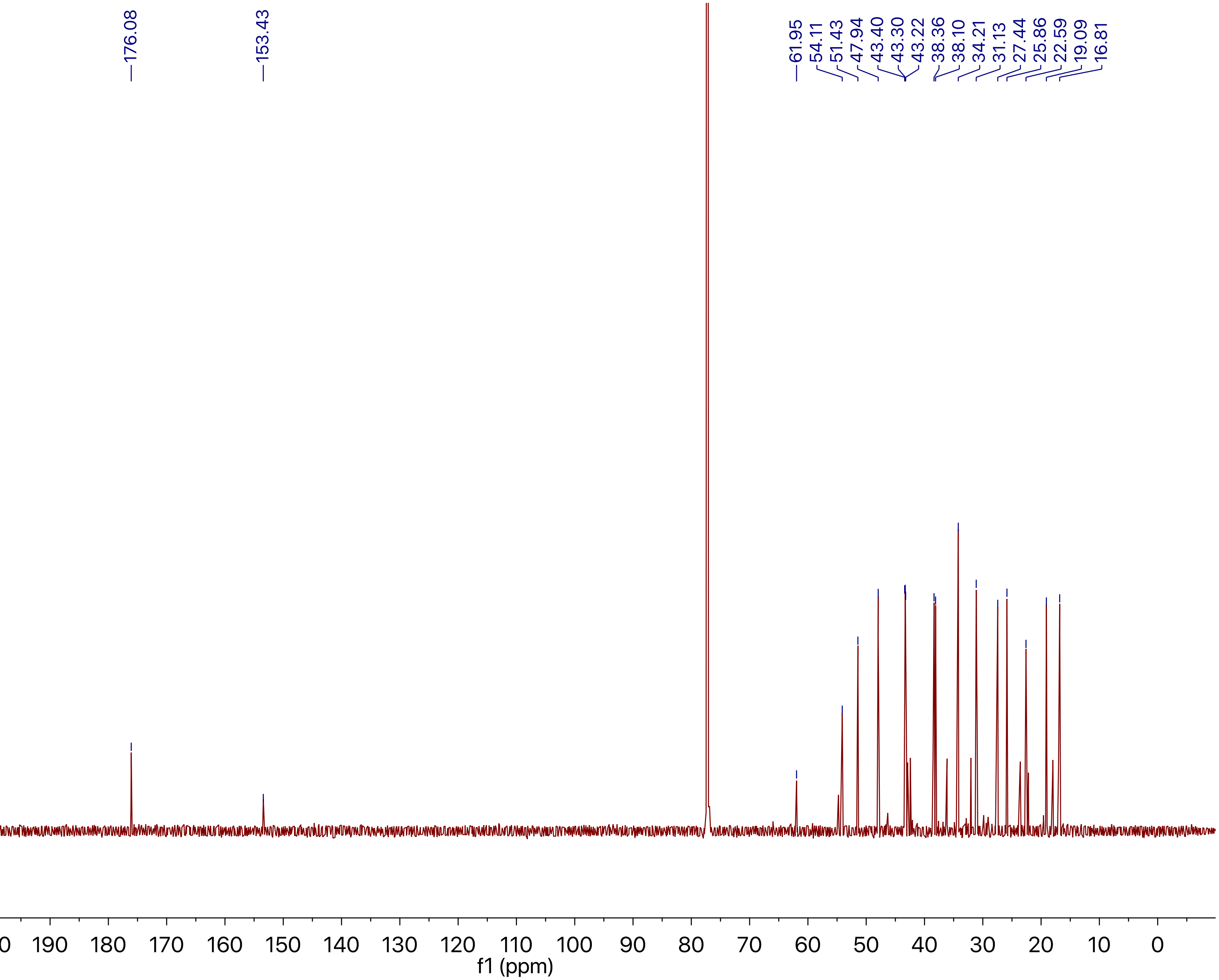
176.08
153.43
61.95
54.11
51.43
47.94
43.40
43.30
43.22
38.36
38.10
34.21
31.13
27.44
25.86
22.59
19.09
16.81
220
210
200
190
180
170
160
150
140
130
120
110
100
90
80
70
60
50
40
30
20
10
0
f1 (ppm)

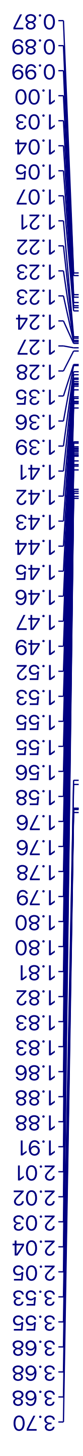
0.87
0.89
0.99
1.00
1.03
1.04
1.05
1.07
1.21
1.22
1.23
1.23
1.24
1.27
1.28
1.35
1.36
1.39
1.41
1.42
1.43
1.44
1.45
1.46
1.47
1.49
1.52
1.53
1.55
1.55
1.56
1.58
1.76
1.76
1.78
1.79
1.80
1.80
1.81
1.82
1.83
1.83
1.86
1.88
1.88
1.91
2.01
2.02
2.03
2.04
2.05
3.53
3.55
3.68
3.68
3.68
3.70

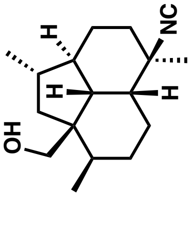
OH
H
H
H
NC

S12

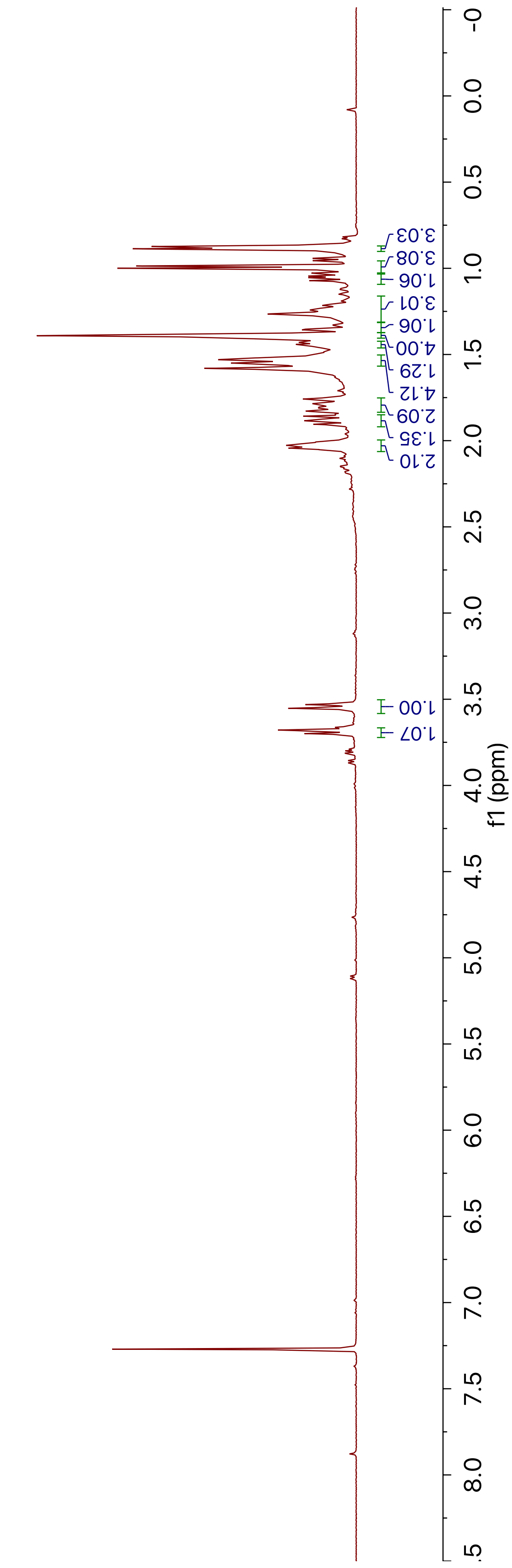
3.03
3.08
1.06
3.01
1.06
4.00
1.29
4.12
2.09
1.35
2.10
1.00
1.07
f1 (ppm)

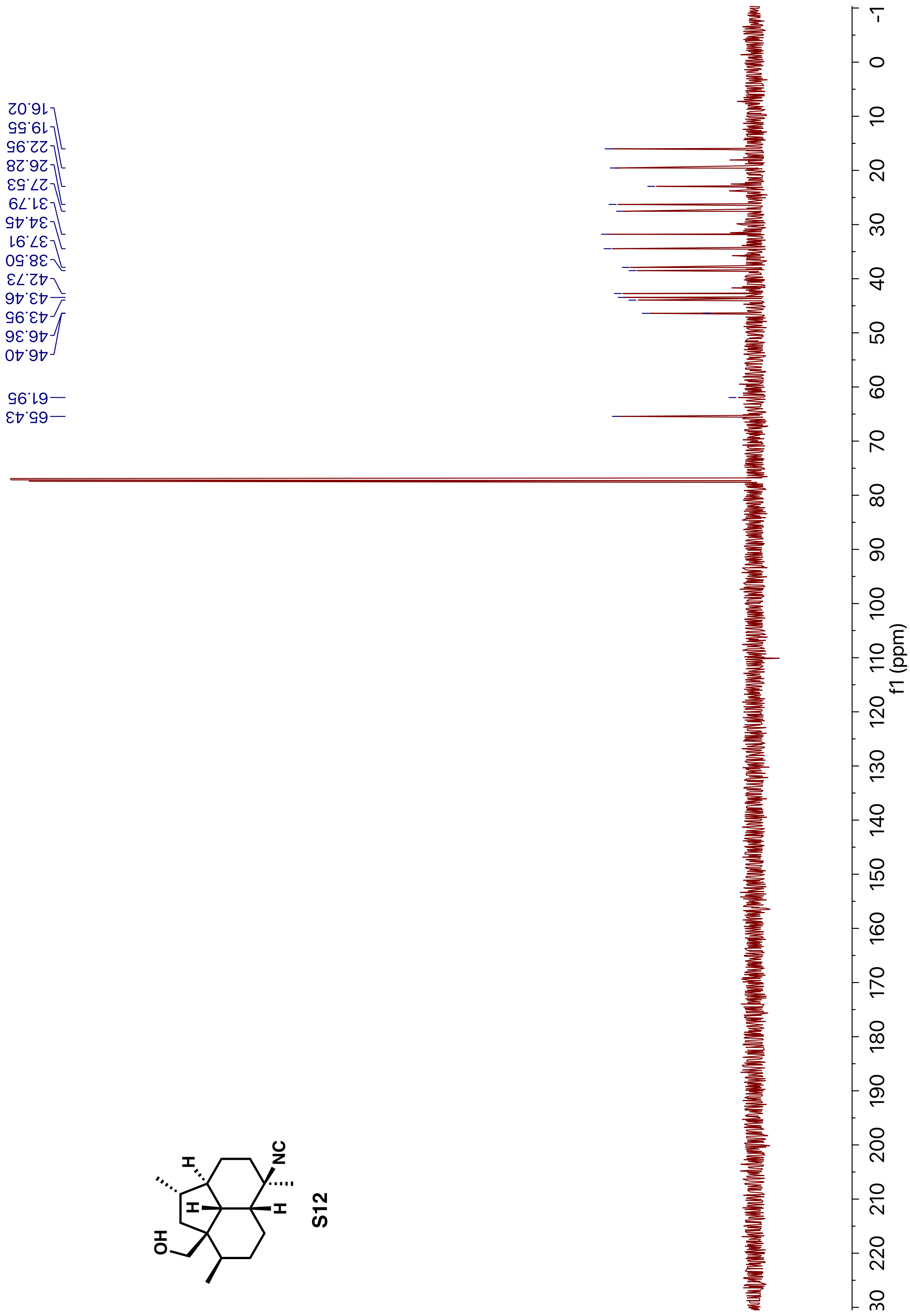
16.02
19.55
22.95
26.28
27.53
31.79
34.45
37.91
38.50
42.73
43.46
43.95
46.36
46.40
61.95
65.43
f1 (ppm)
S12

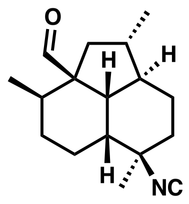

S13

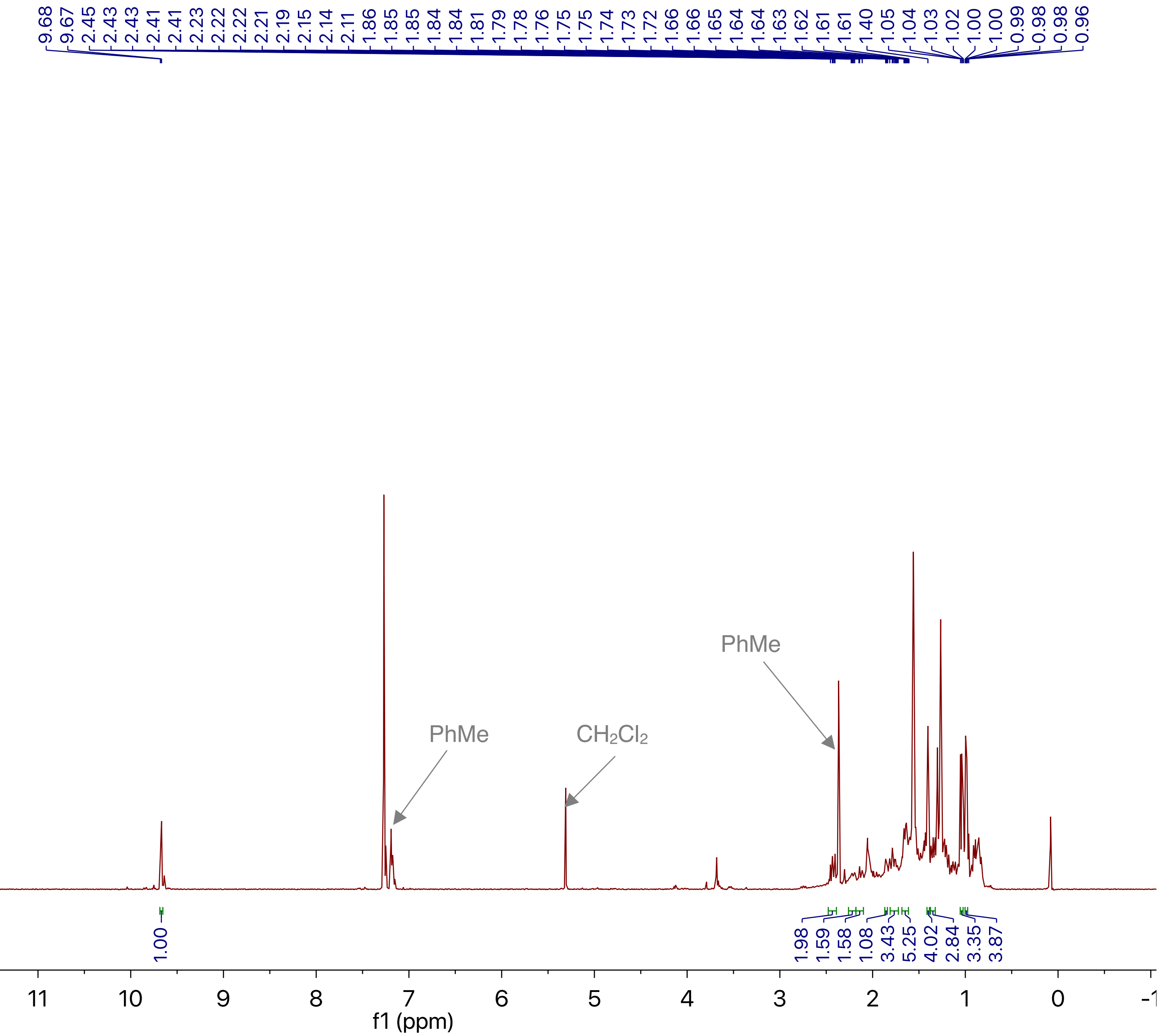
9.68 9.67 2.45 2.43 2.43 2.41 2.41 2.23 2.22 2.22 2.21 2.19 2.15 2.14 2.11 1.86 1.85 1.85 1.84 1.84 1.81 1.79 1.78 1.76 1.75 1.75 1.74 1.73 1.72 1.66 1.66 1.65 1.64 1.64 1.63 1.62 1.61 1.61 1.40 1.05 1.04 1.03 1.02 1.00 1.00 0.99 0.98 0.98 0.96
PhMe
CH2Cl2
PhMe
1.00
1.98 1.59 1.58 1.08 3.43 5.25 4.02 2.84 3.35 3.87
14 13 12 11 10 9 8 7 6 5 4 3 2 1 0 -1
f1 (ppm)

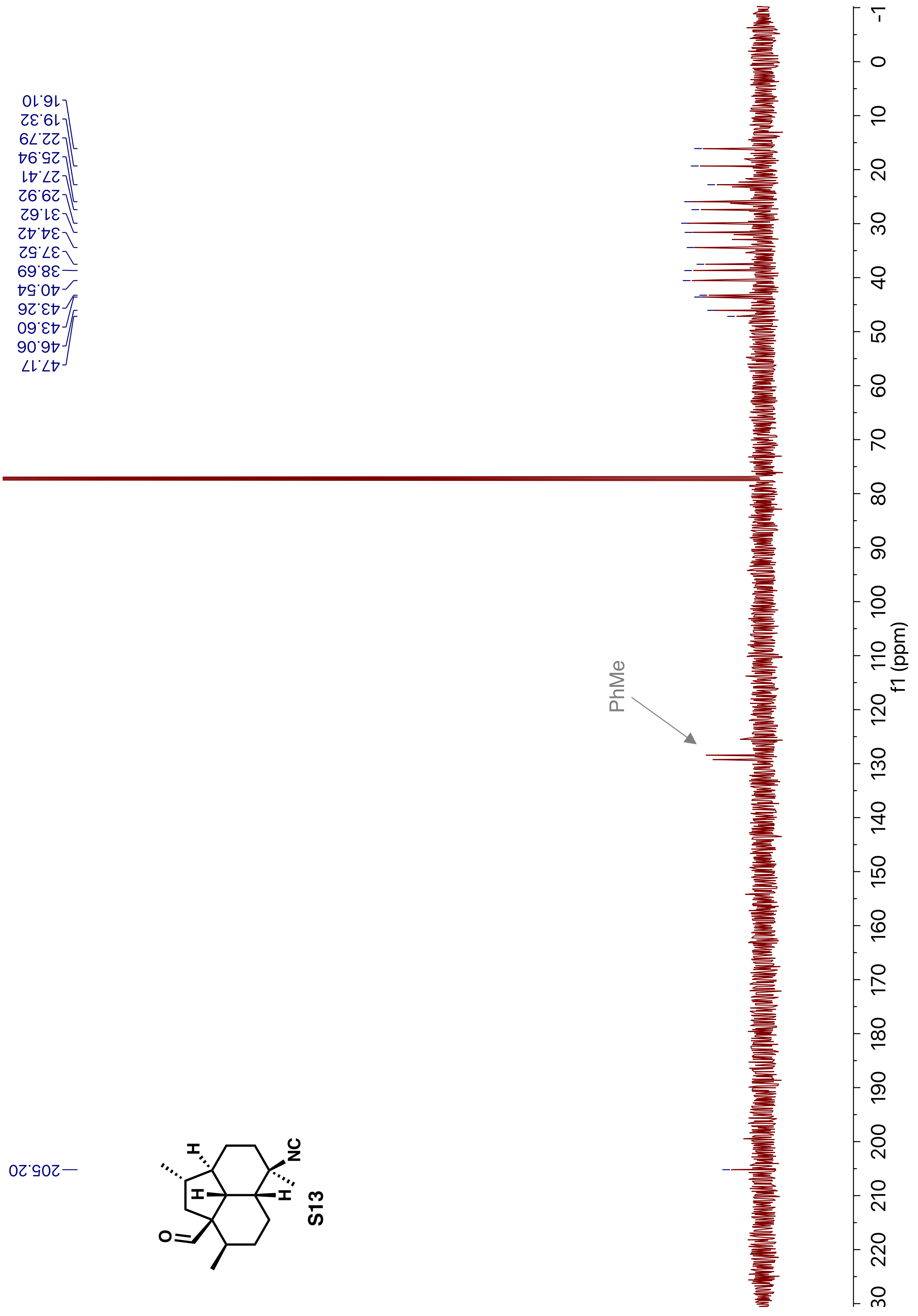

205.20
47.17
46.06
43.60
43.26
40.54
38.69
37.52
34.42
31.62
29.92
27.41
25.94
22.79
19.32
16.10
S13
PhMe
f1 (ppm)

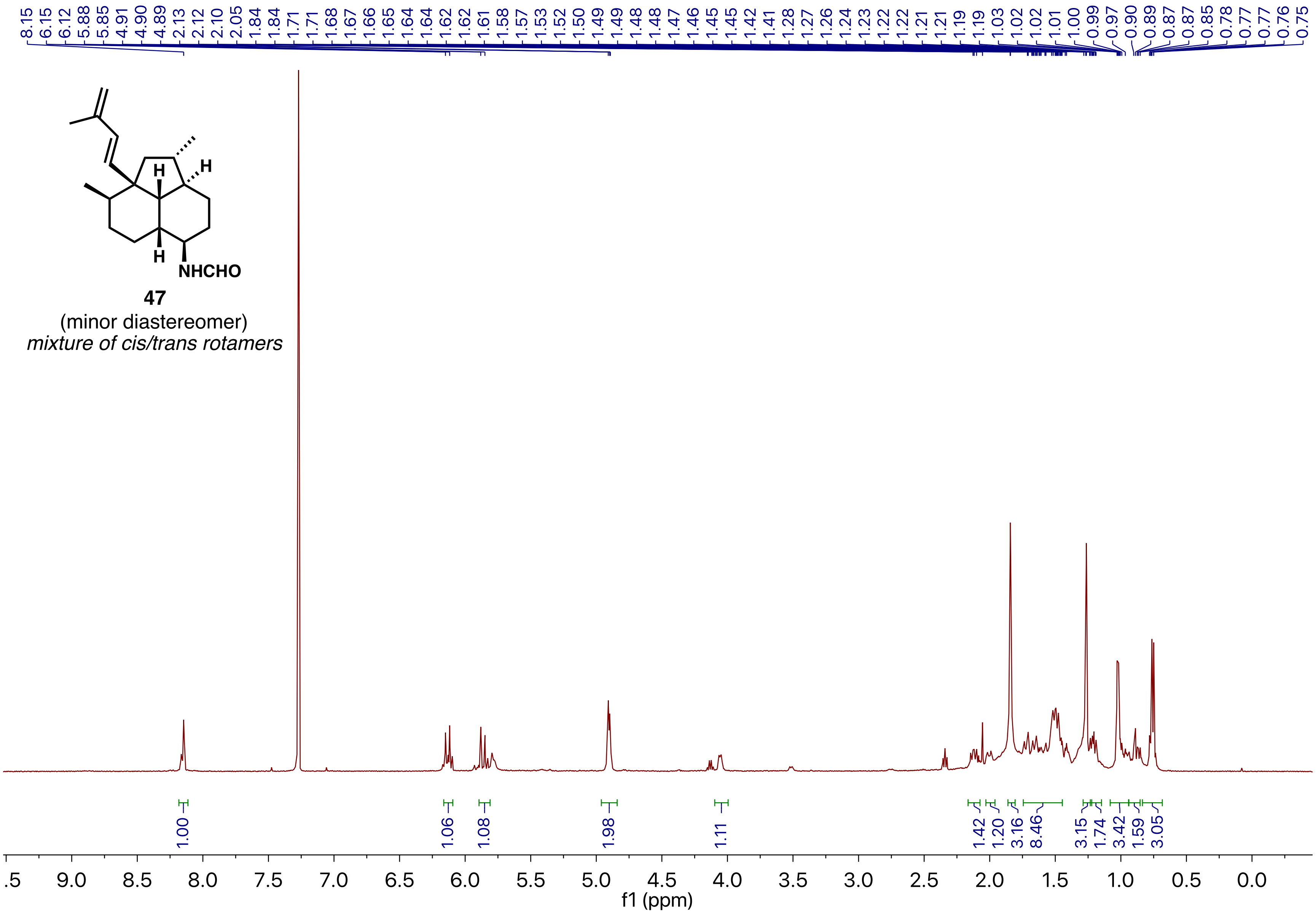

47
(minor diastereomer)
mixture of cis/trans rotamers

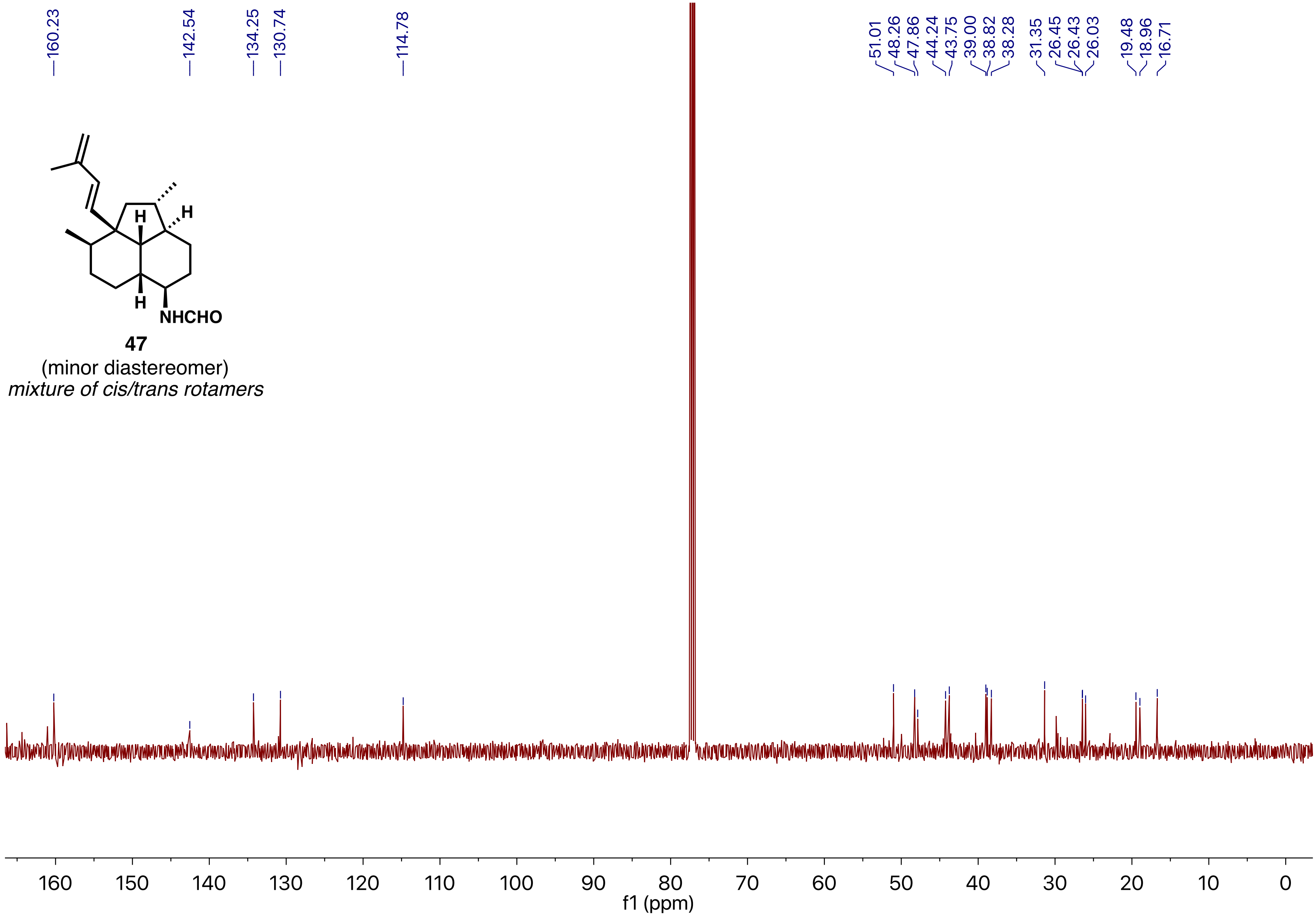

47
(minor diastereomer)
mixture of cis/trans rotamers

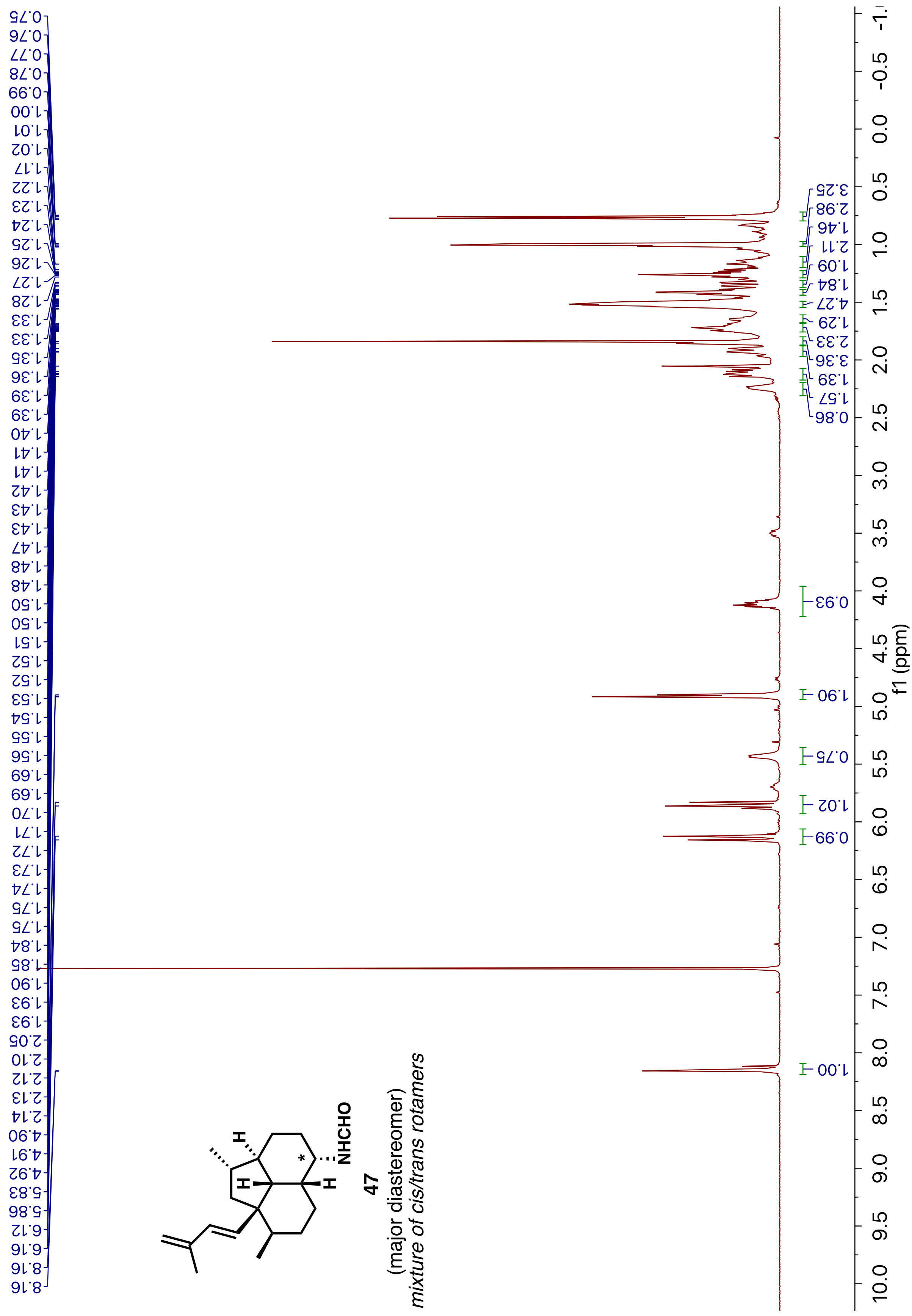

47
(major diastereomer)
mixture of cis/trans rotamers

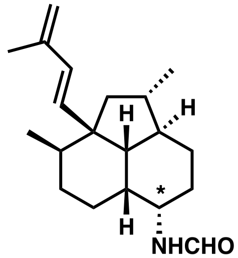

47
(major diastereomer)
mixture of cis/trans rotamers

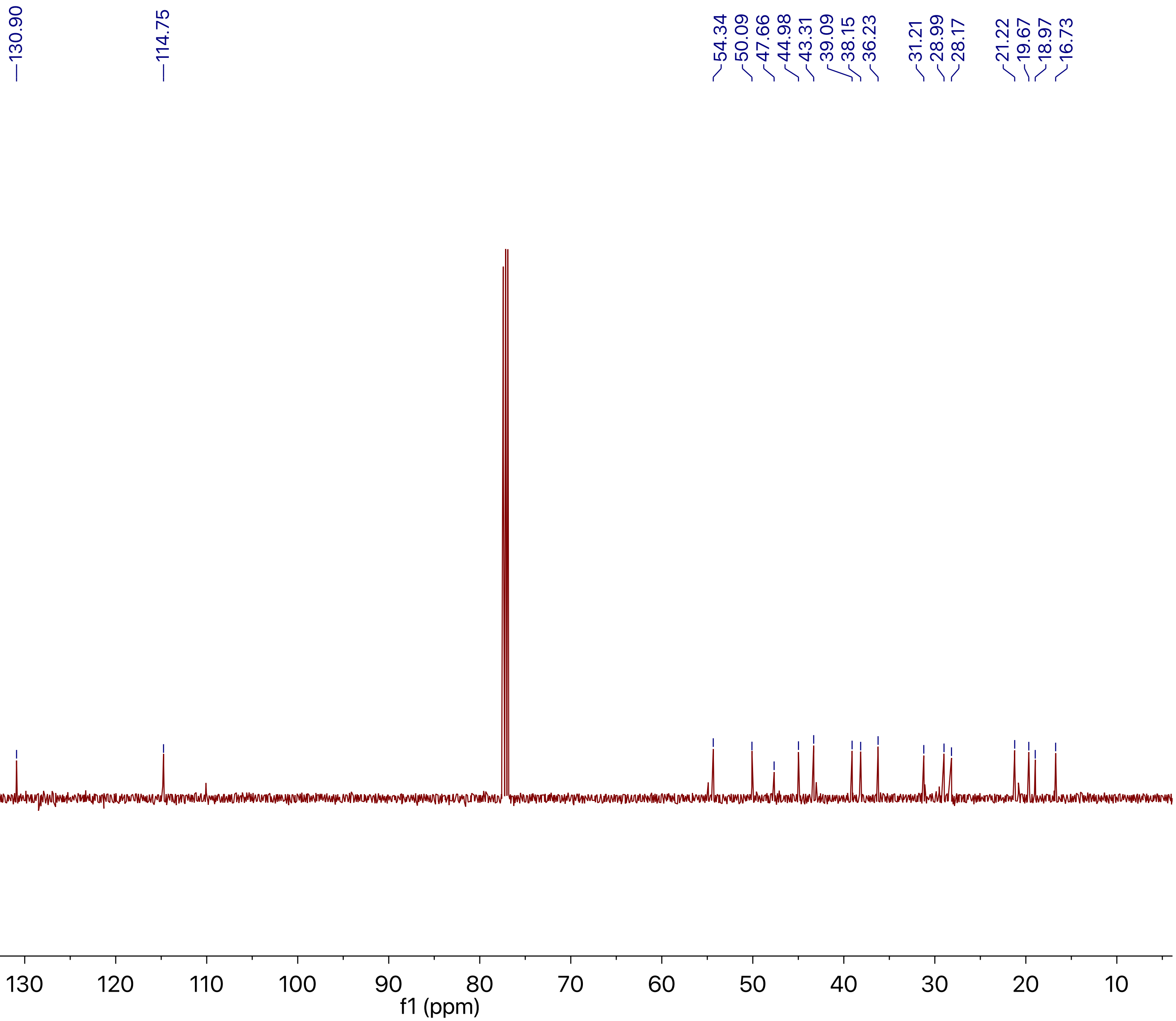

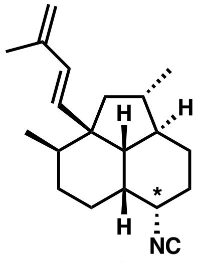

48
3:1 dr ar *

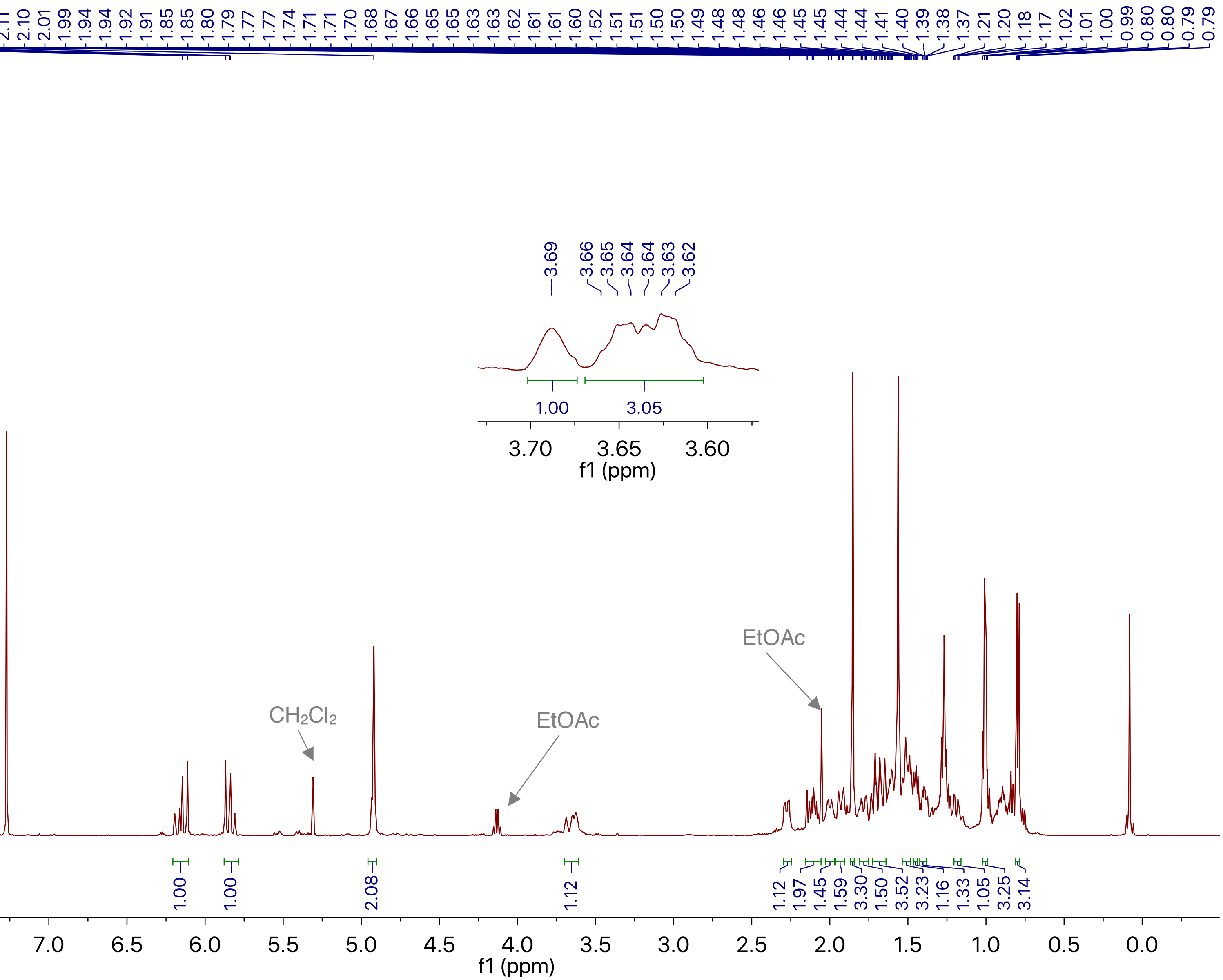
6.15 6.11 5.87 5.84 5.84 4.92 2.26 2.15 2.11 2.10 2.01 1.99 1.94 1.94 1.92 1.91 1.85 1.85 1.80 1.79 1.77 1.77 1.74 1.71 1.71 1.70 1.68 1.67 1.66 1.65 1.65 1.63 1.63 1.62 1.61 1.61 1.60 1.52 1.51 1.51 1.50 1.50 1.49 1.48 1.48 1.46 1.46 1.45 1.45 1.44 1.44 1.41 1.40 1.39 1.38 1.37 1.21 1.20 1.18 1.17 1.02 1.01 1.00 0.99 0.80 0.80 0.79 0.79
3.69 3.66 3.65 3.64 3.64 3.63 3.62
1.00
3.05
3.70 3.65 3.60
f1 (ppm)
EtOAc
CH2Cl2
EtOAc
1.00 1.00 2.08 1.12 1.12 1.97 1.45 1.59 3.30 1.50 3.52 3.23 1.16 1.33 1.05 3.25 3.14
8.0 7.5 7.0 6.5 6.0 5.5 5.0 4.5 4.0 3.5 3.0 2.5 2.0 1.5 1.0 0.5 0.0
f1 (ppm)

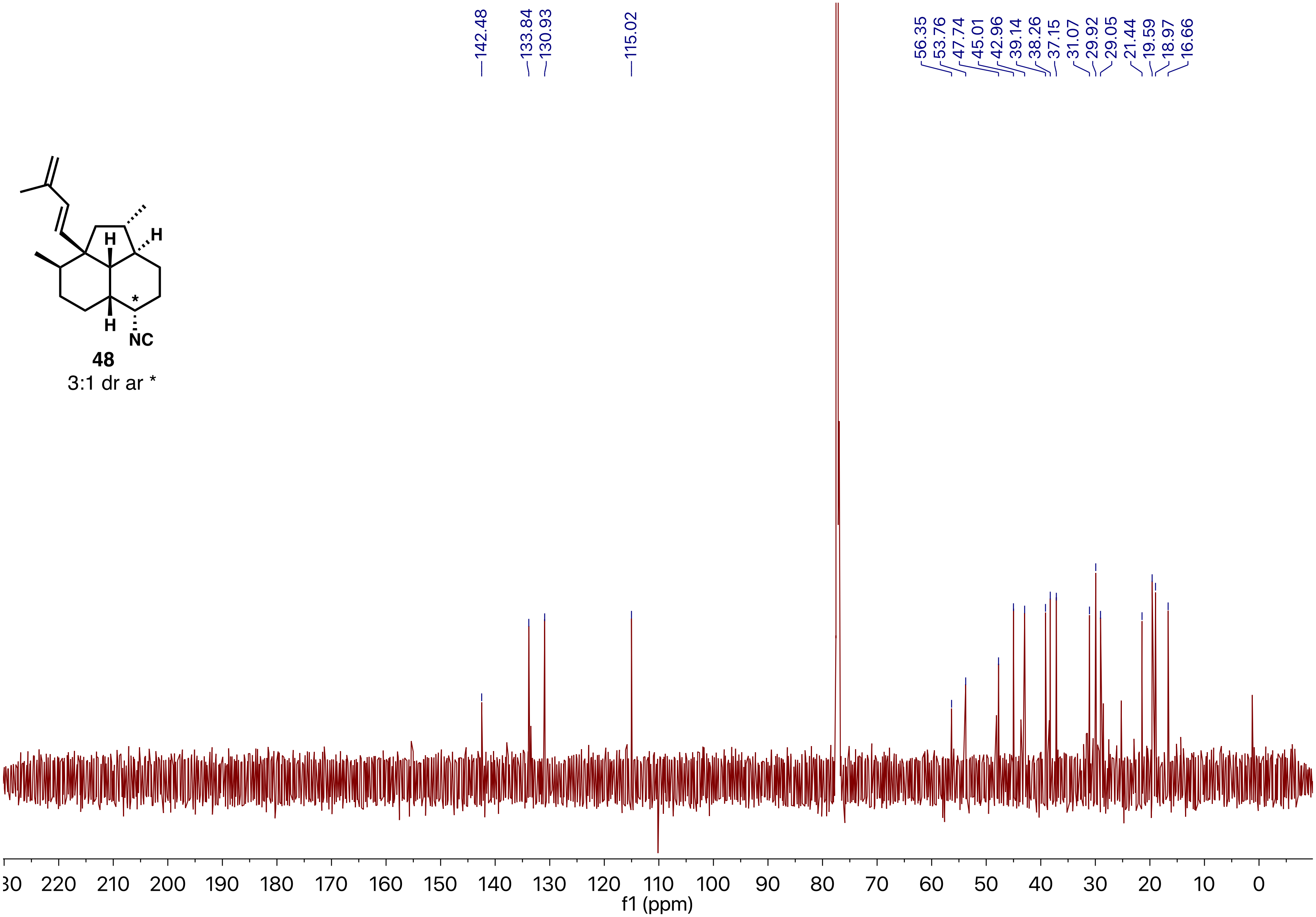

142.48
133.84
130.93
115.02
56.35
53.76
47.74
45.01
42.96
39.14
38.26
37.15
31.07
29.92
29.05
21.44
19.59
18.97
16.66
f1 (ppm)

48
3:1 dr ar *

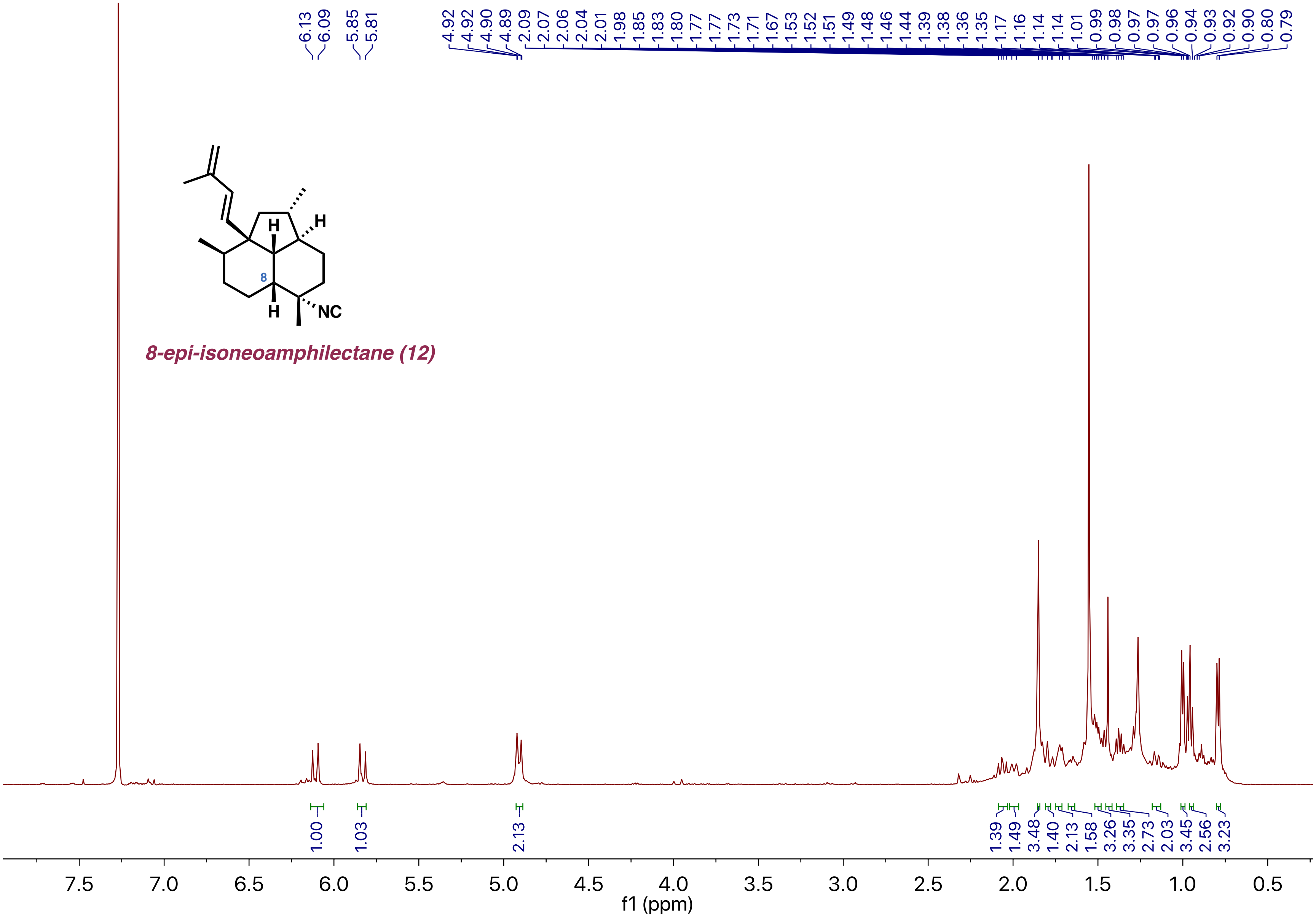

8-epi-isoneoamphilectane (12)

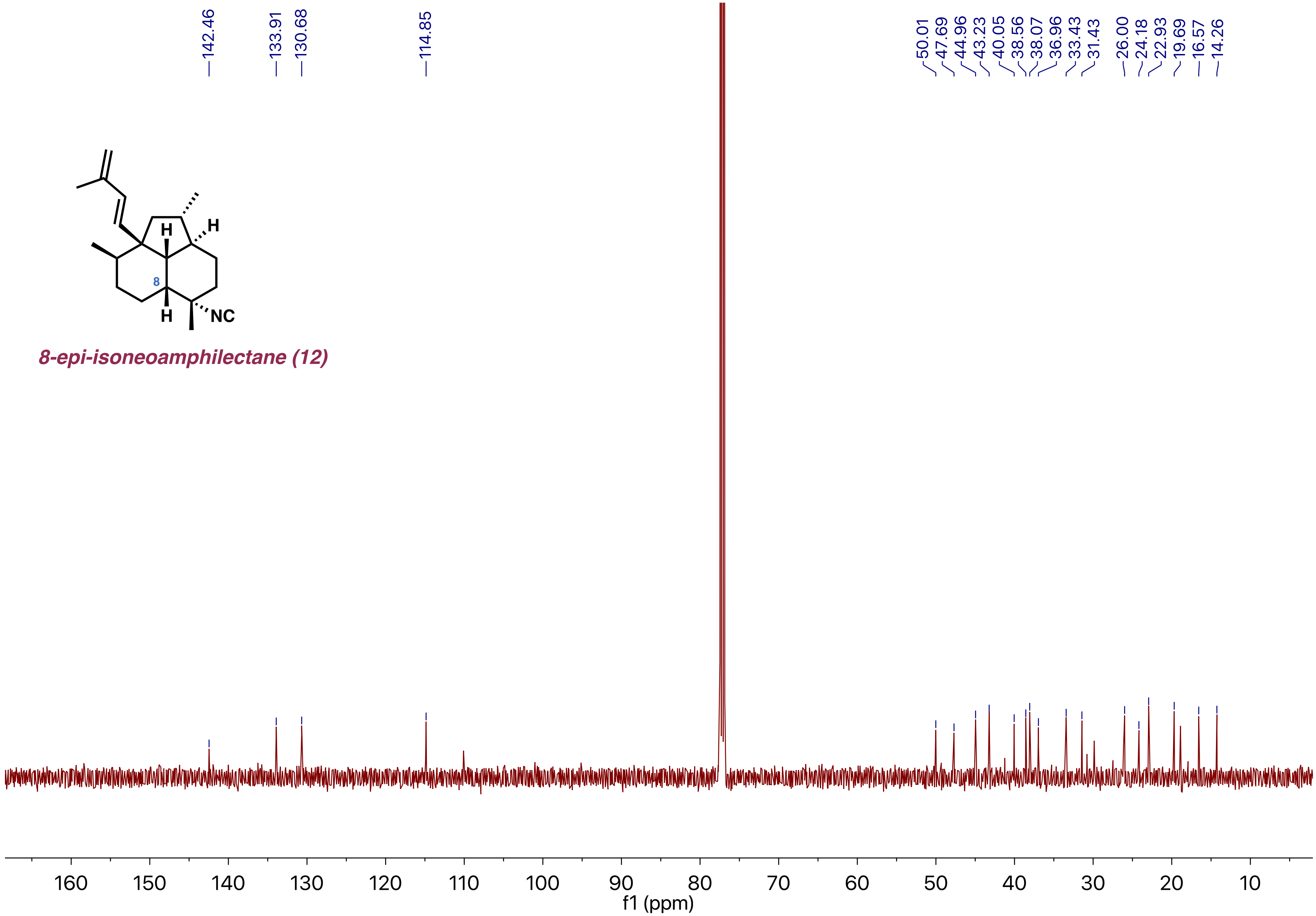

8-epi-isoneoamphilectane (12)

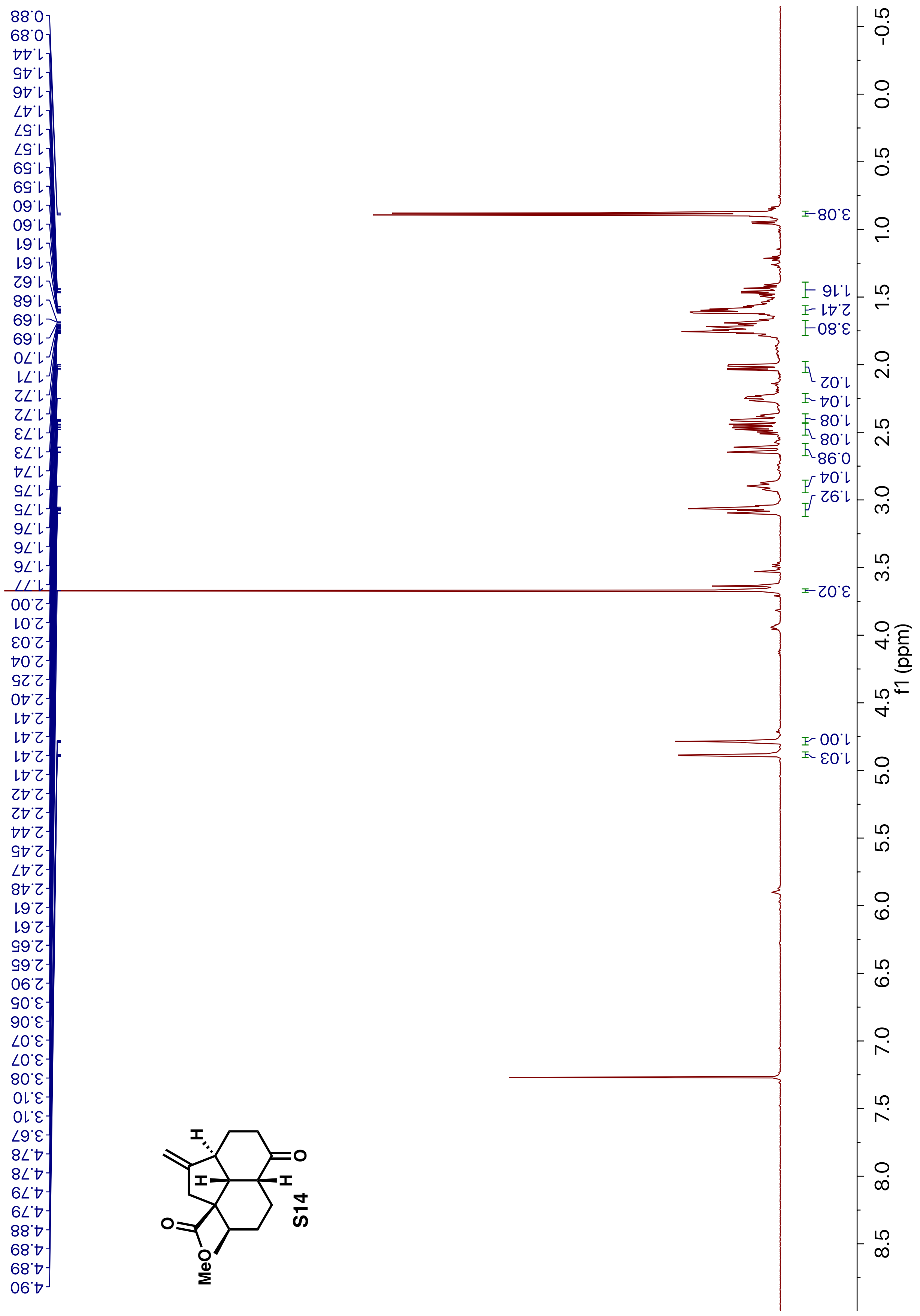
0.88 0.89 1.44 1.45 1.46 1.47 1.57 1.57 1.59 1.59 1.60 1.60 1.61 1.61 1.62 1.68 1.69 1.69 1.70 1.71 1.72 1.72 1.73 1.73 1.74 1.75 1.75 1.76 1.76 1.76 1.77 2.00 2.01 2.03 2.04 2.25 2.40 2.41 2.41 2.41 2.41 2.42 2.42 2.44 2.45 2.47 2.48 2.61 2.61 2.65 2.65 2.90 3.05 3.06 3.07 3.07 3.08 3.10 3.10 3.67 4.78 4.78 4.79 4.79 4.88 4.89 4.89 4.90
S14
MeO
f1 (ppm)

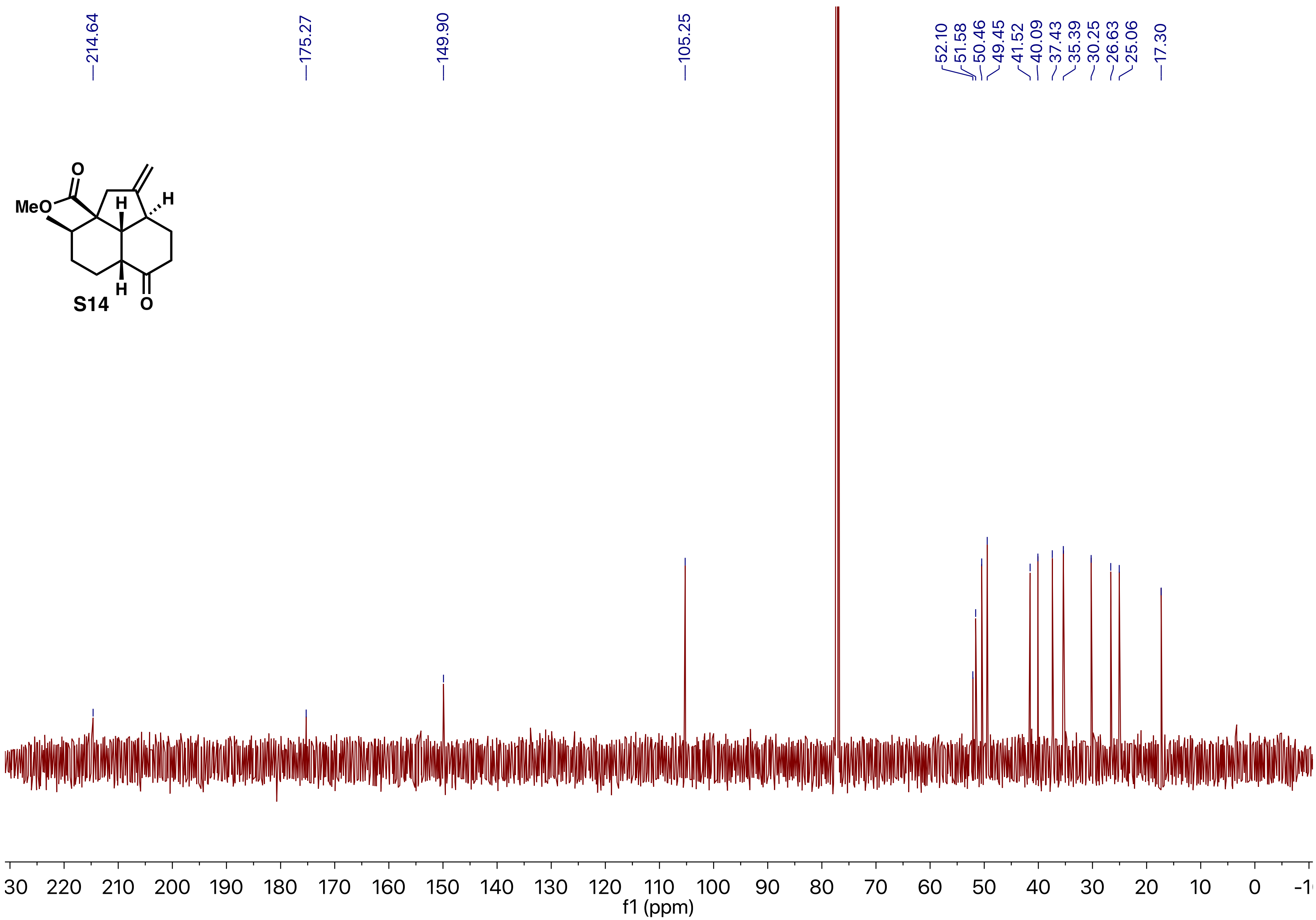
214.64
175.27
149.90
105.25
52.10
51.58
50.46
49.45
41.52
40.09
37.43
35.39
30.25
26.63
25.06
17.30
MeO
O
H
H
H
O
S14
f1 (ppm)

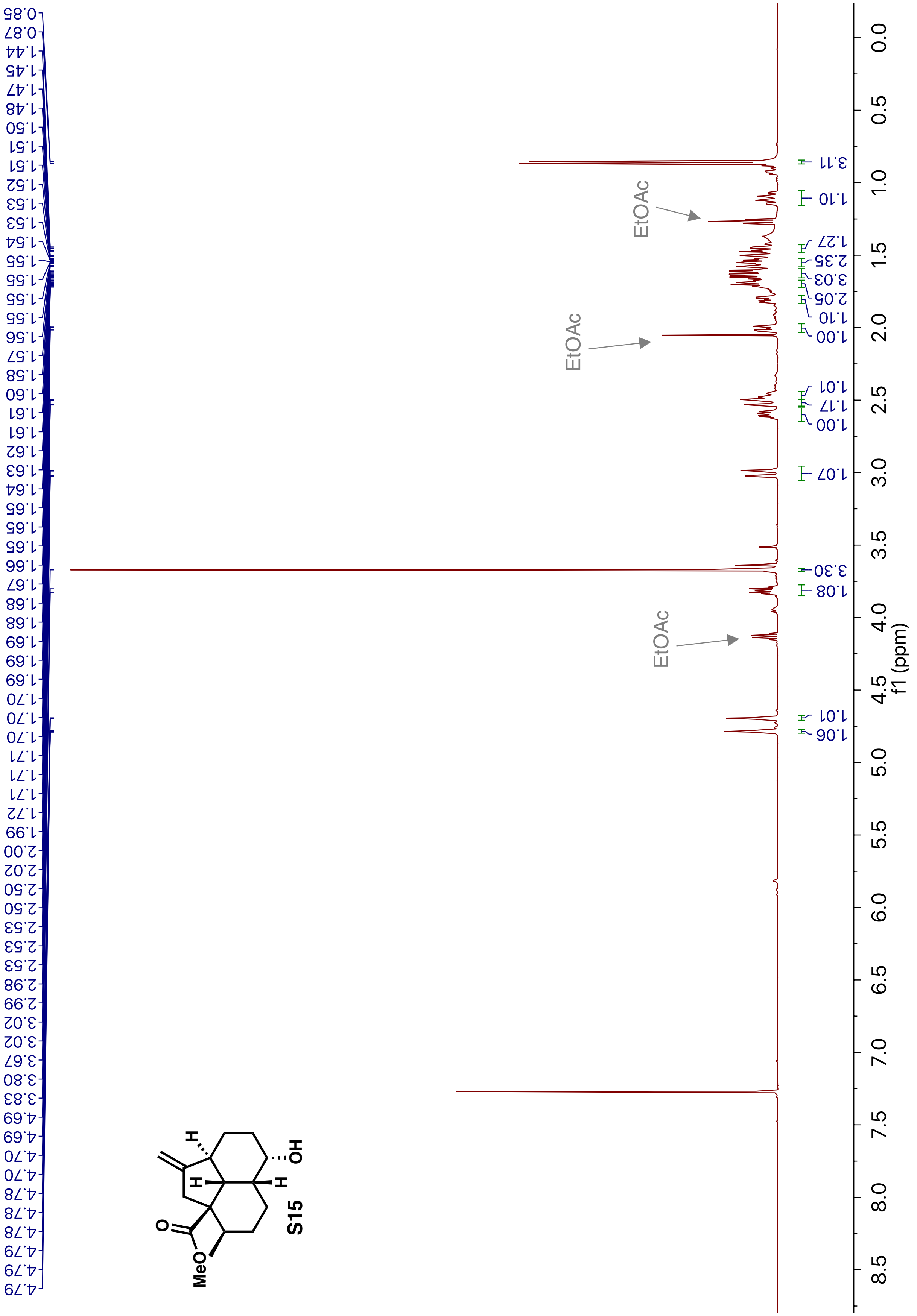

0.85
0.87
1.44
1.45
1.47
1.48
1.50
1.51
1.51
1.52
1.53
1.53
1.54
1.55
1.55
1.55
1.55
1.56
1.57
1.58
1.60
1.61
1.61
1.62
1.63
1.64
1.65
1.65
1.65
1.66
1.67
1.68
1.68
1.69
1.69
1.69
1.70
1.70
1.70
1.71
1.71
1.71
1.72
1.99
2.00
2.02
2.50
2.50
2.53
2.53
2.53
2.98
2.99
3.02
3.02
3.67
3.80
3.83
4.69
4.69
4.70
4.70
4.78
4.78
4.78
4.79
4.79
4.79
EtOAc
EtOAc
EtOAc
3.11
1.10
1.27
2.35
3.03
2.05
1.10
1.00
1.01
1.17
1.00
1.07
3.30
1.08
1.01
1.06
f1 (ppm)
MeO
O
H
H
H
OH
S15

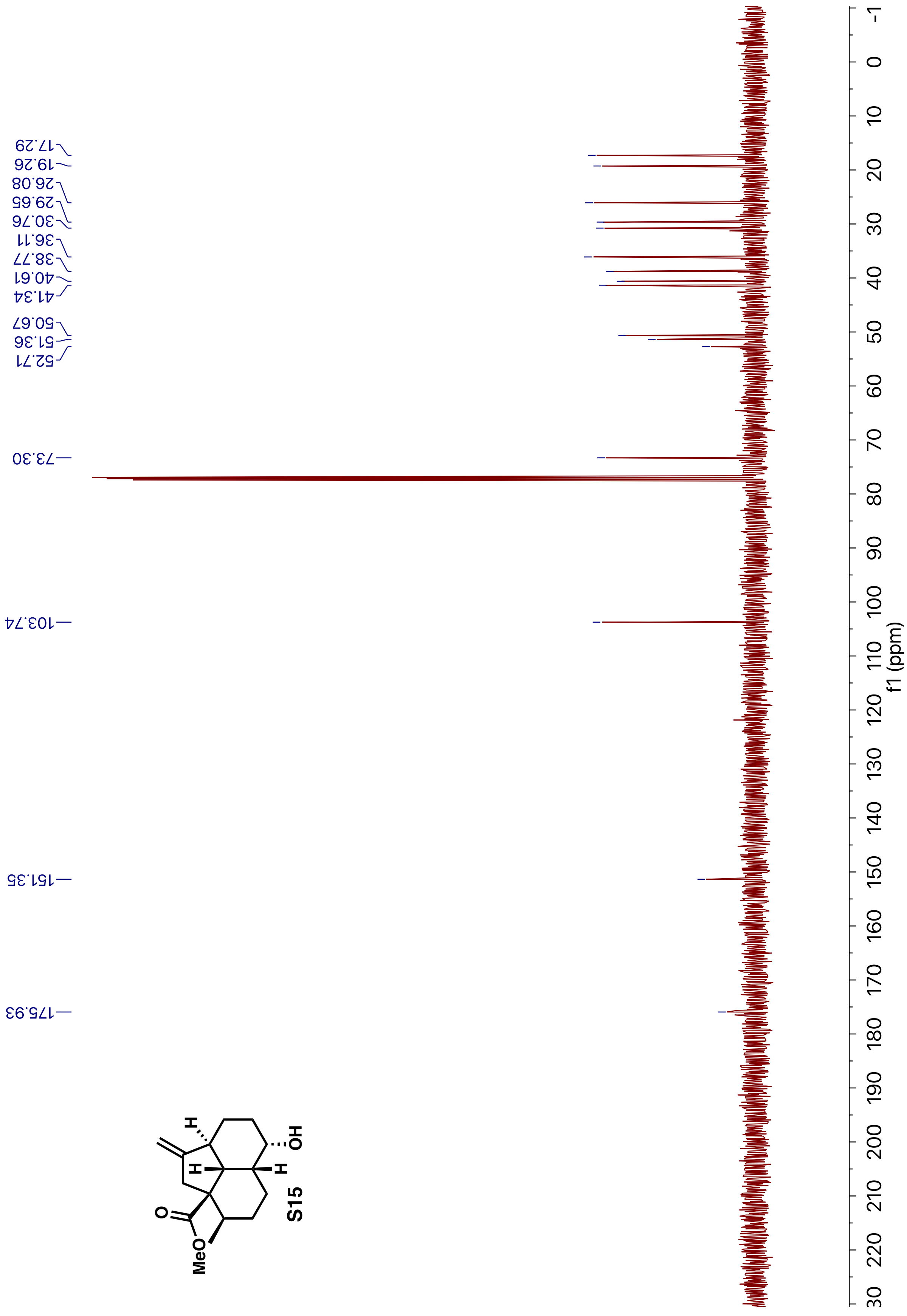
17.29
19.26
26.08
29.65
30.76
36.11
38.77
40.61
41.34
50.67
51.36
52.71
73.30
103.74
151.35
175.93
f1 (ppm)
MeO
O
H
H
OH
S15

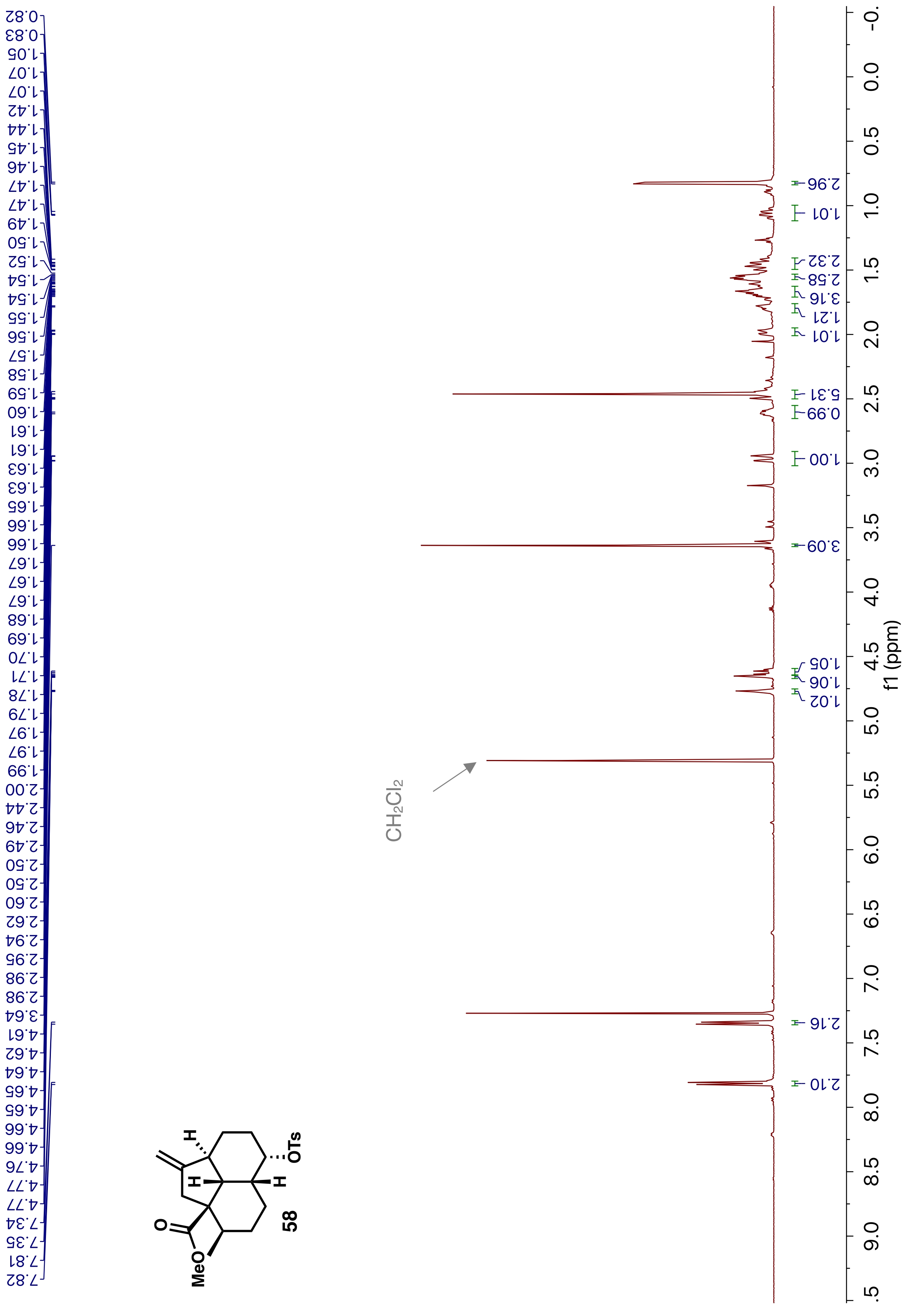

0.82
0.83
1.05
1.07
1.07
1.42
1.44
1.45
1.46
1.47
1.47
1.49
1.50
1.52
1.54
1.54
1.55
1.56
1.57
1.58
1.59
1.60
1.61
1.61
1.63
1.63
1.65
1.66
1.66
1.67
1.67
1.67
1.68
1.69
1.70
1.71
1.78
1.79
1.97
1.97
1.99
2.00
2.44
2.46
2.49
2.50
2.50
2.60
2.62
2.94
2.95
2.98
2.98
3.64
4.61
4.62
4.64
4.65
4.65
4.66
4.66
4.76
4.77
4.77
7.34
7.35
7.81
7.82
MeO
O
H
H
H
OTs
58
CH2Cl2
2.96
1.01
2.32
2.58
3.16
1.21
1.01
5.31
0.99
1.00
3.09
1.05
1.06
1.02
2.16
2.10
f1 (ppm)

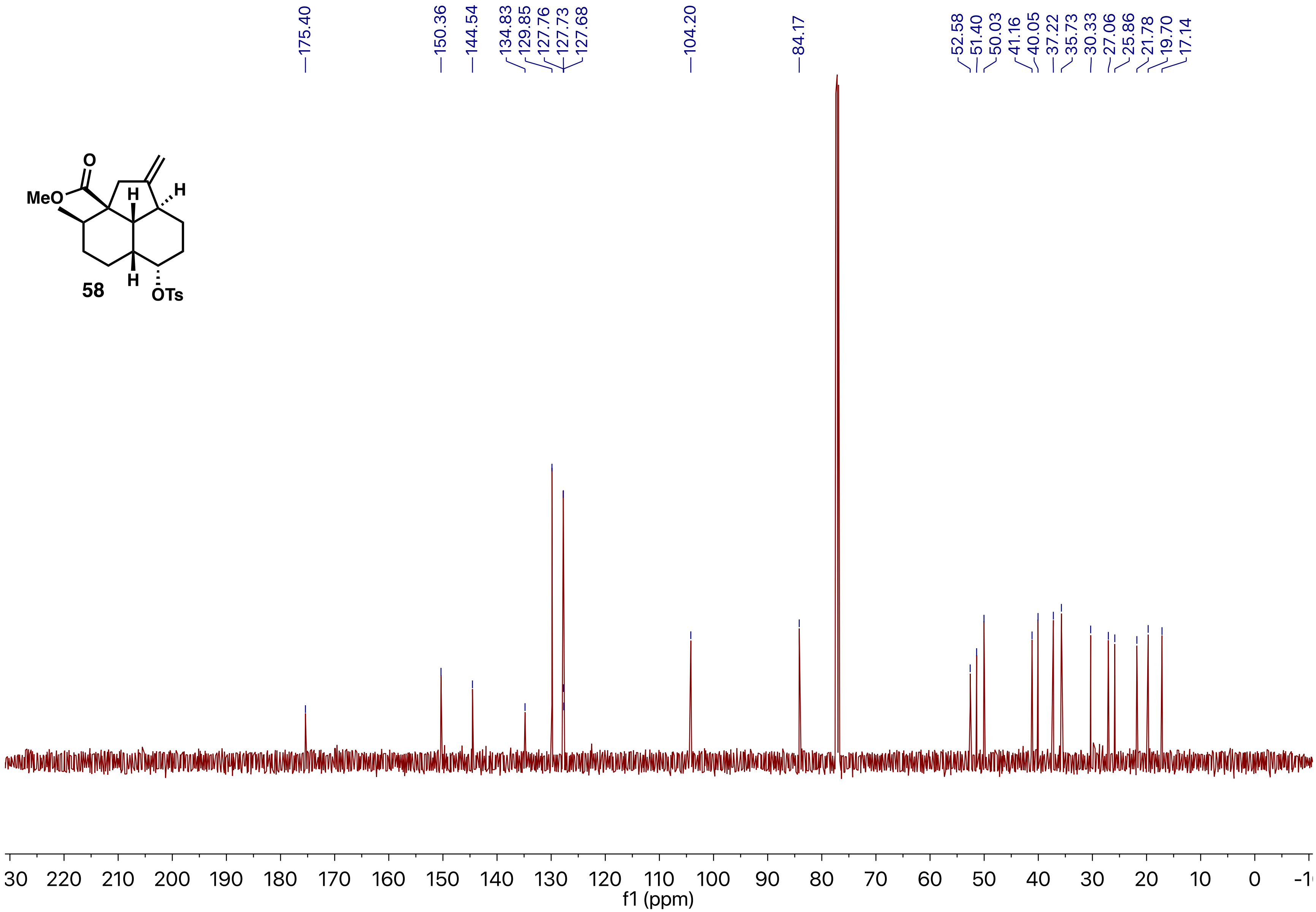

MeO
O
H
H
H
OTs
175.40
150.36
144.54
134.83
129.85
127.76
127.73
127.68
104.20
84.17
52.58
51.40
50.03
41.16
40.05
37.22
35.73
30.33
27.06
25.86
21.78
19.70
17.14
f1 (ppm)

58

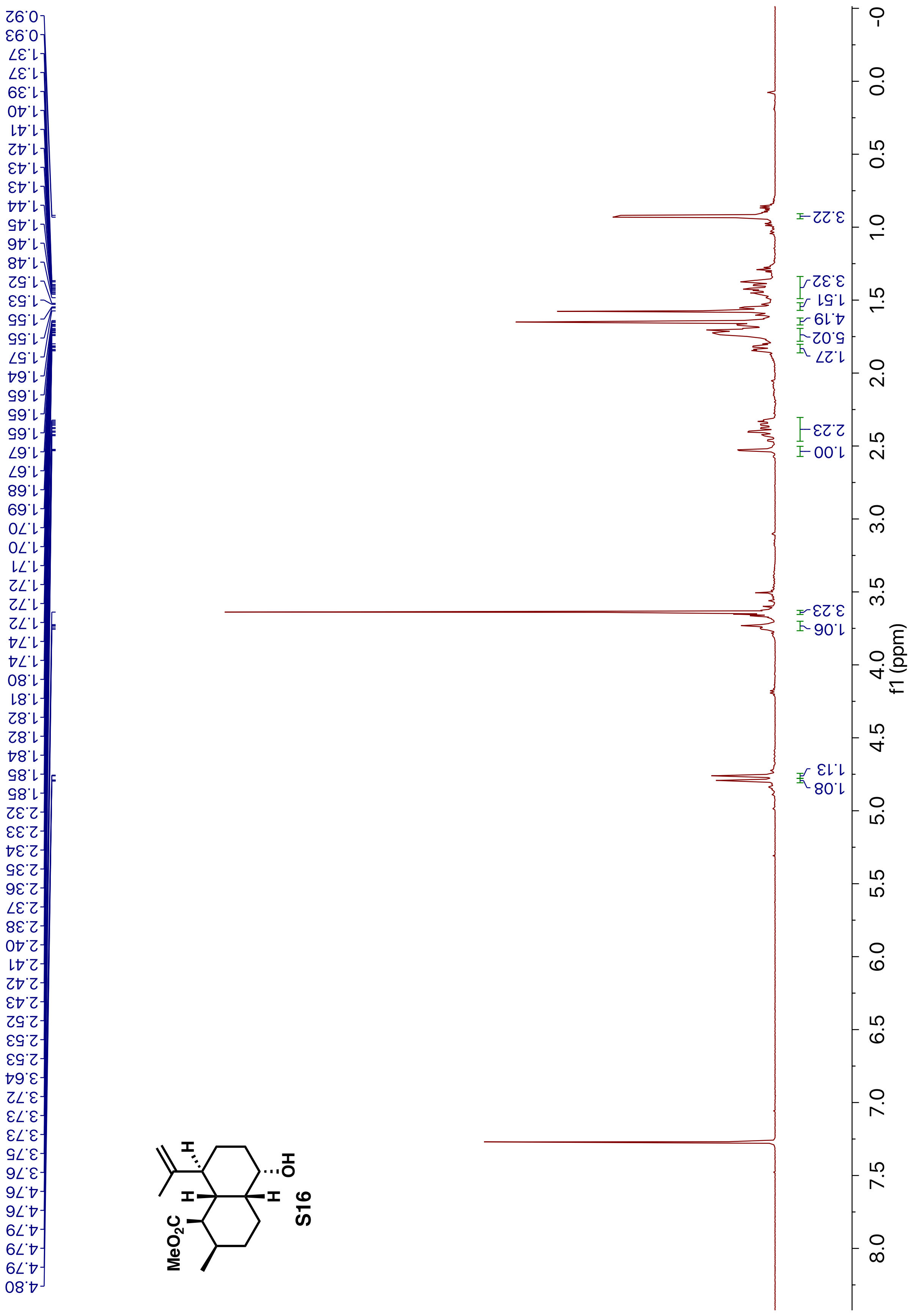
0.92
0.93
1.37
1.37
1.39
1.40
1.41
1.42
1.43
1.43
1.44
1.45
1.46
1.48
1.52
1.53
1.55
1.55
1.57
1.64
1.65
1.65
1.65
1.67
1.67
1.68
1.69
1.70
1.70
1.71
1.72
1.72
1.72
1.74
1.74
1.80
1.81
1.82
1.82
1.84
1.85
1.85
2.32
2.33
2.34
2.35
2.36
2.37
2.38
2.40
2.41
2.42
2.43
2.52
2.53
2.53
3.64
3.72
3.73
3.73
3.75
3.76
4.76
4.76
4.79
4.79
4.79
4.80
MeO2C
H
H
H
OH
S16
3.22
3.32
1.51
4.19
5.02
1.27
2.23
1.00
3.23
1.06
1.13
1.08
f1 (ppm)

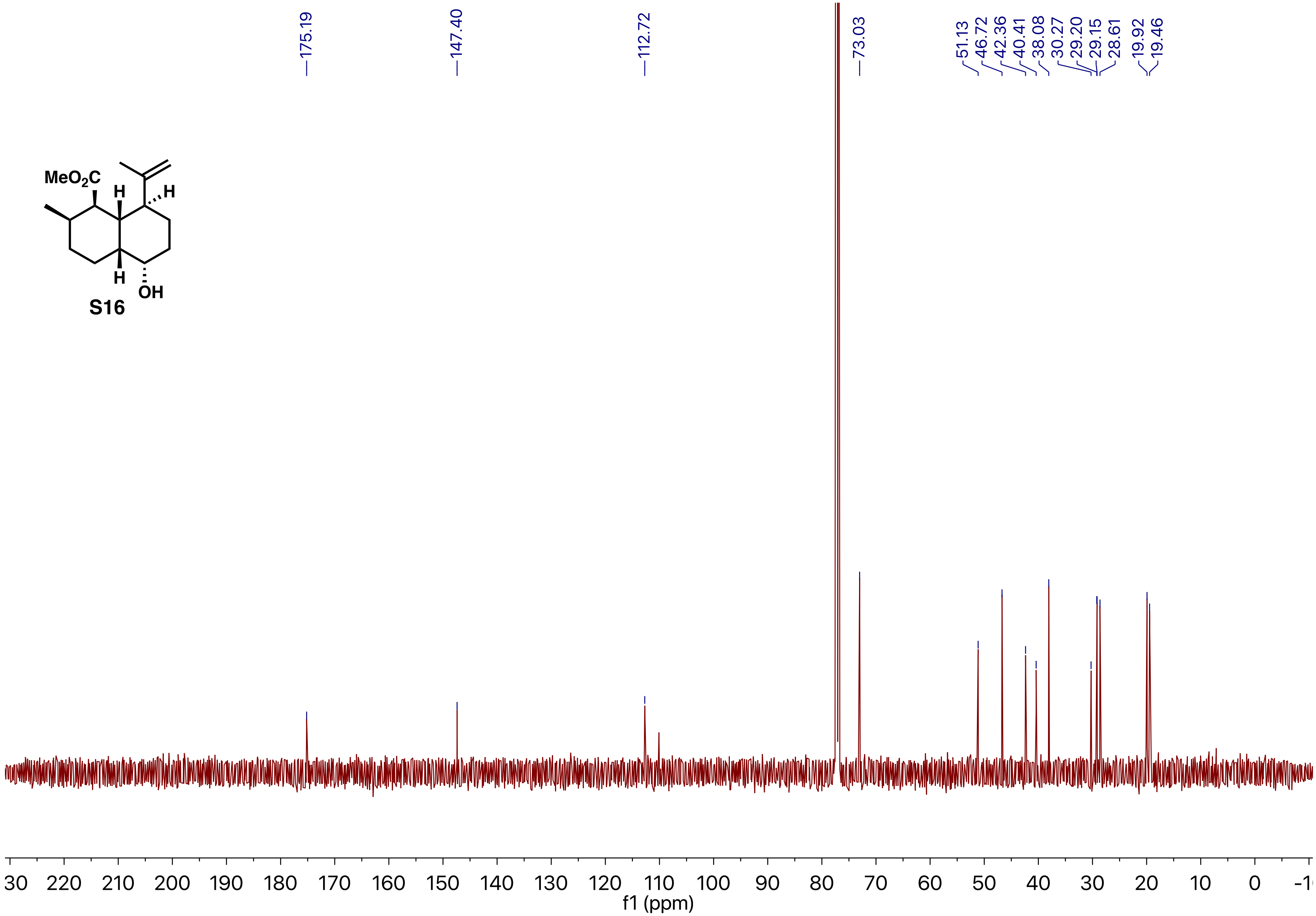
MeO2C
H
H
H
OH
S16
175.19
147.40
112.72
73.03
51.13
46.72
42.36
40.41
38.08
30.27
29.20
29.15
28.61
19.92
19.46
30 220 210 200 190 180 170 160 150 140 130 120 110 100 90 80 70 60 50 40 30 20 10 0 -1
f1 (ppm)

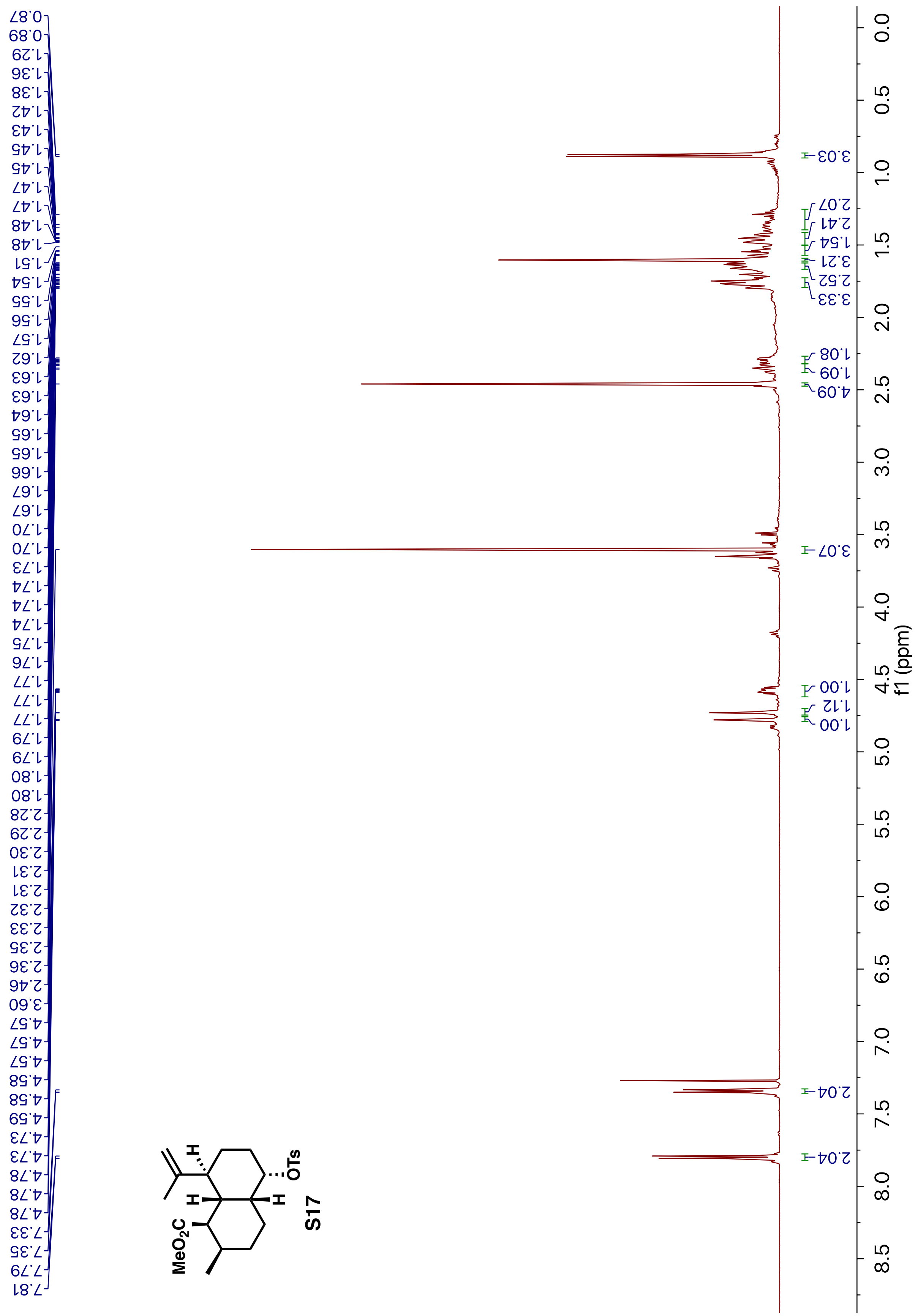
0.87
0.89
1.29
1.36
1.38
1.42
1.43
1.45
1.45
1.47
1.47
1.48
1.48
1.51
1.54
1.55
1.56
1.57
1.62
1.63
1.63
1.64
1.65
1.65
1.66
1.67
1.67
1.70
1.70
1.73
1.74
1.74
1.74
1.75
1.76
1.77
1.77
1.77
1.79
1.79
1.80
1.80
2.28
2.29
2.30
2.31
2.31
2.32
2.33
2.35
2.36
2.46
3.60
4.57
4.57
4.57
4.58
4.58
4.59
4.73
4.78
4.78
4.78
7.33
7.35
7.79
7.81
MeO2C
H
H
H
OTs
S17
3.03
2.07
2.41
1.54
3.21
2.52
3.33
1.08
1.09
4.09
3.07
1.00
1.12
1.00
2.04
2.04
0.0
0.5
1.0
1.5
2.0
2.5
3.0
3.5
4.0
4.5
5.0
5.5
6.0
6.5
7.0
7.5
8.0
8.5
f1 (ppm)

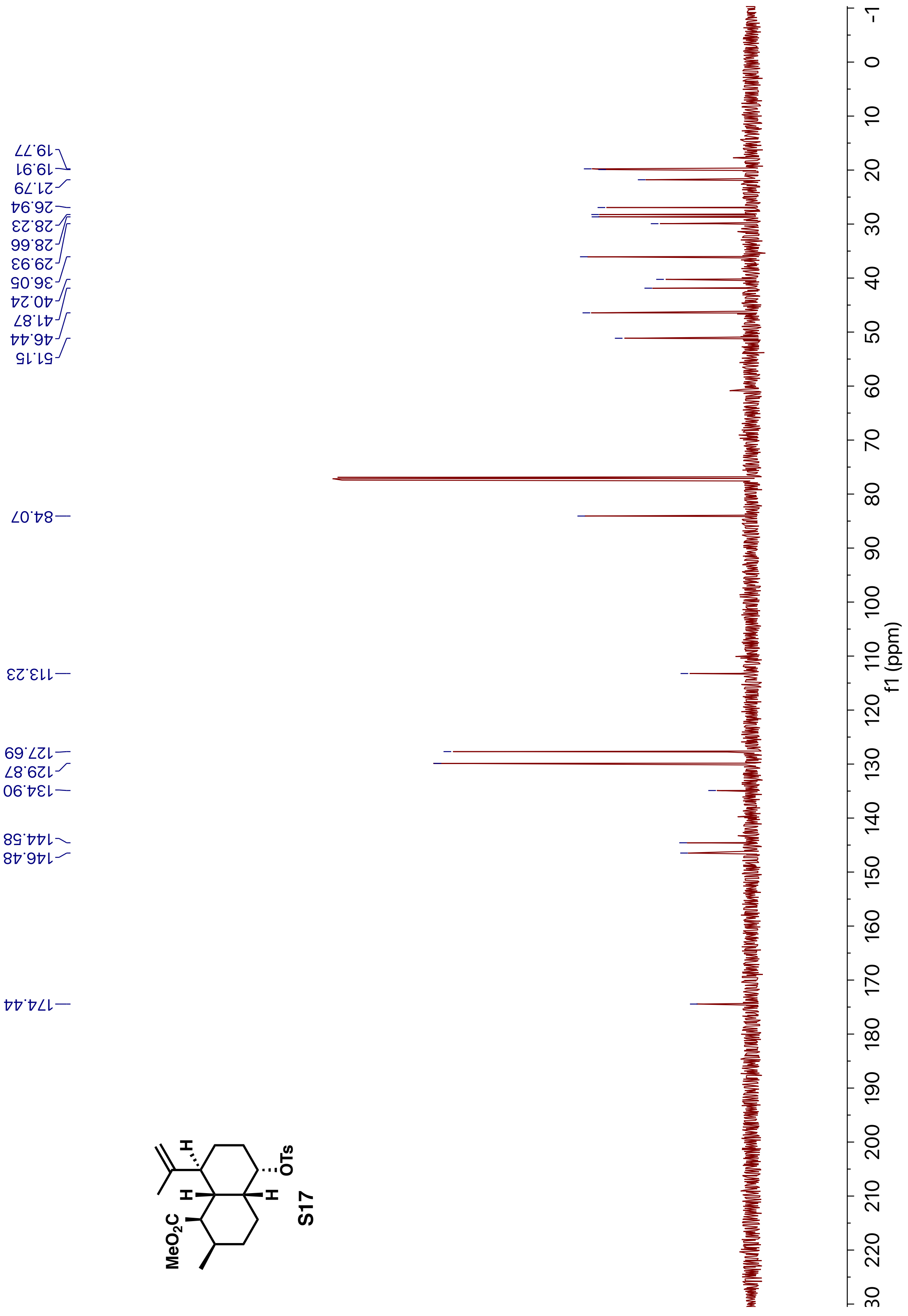

19.77
19.91
21.79
26.94
28.23
28.66
29.93
36.05
40.24
41.87
46.44
51.15
84.07
113.23
127.69
129.87
134.90
144.58
146.48
174.44
f1 (ppm)
MeO2C
OTs
S17

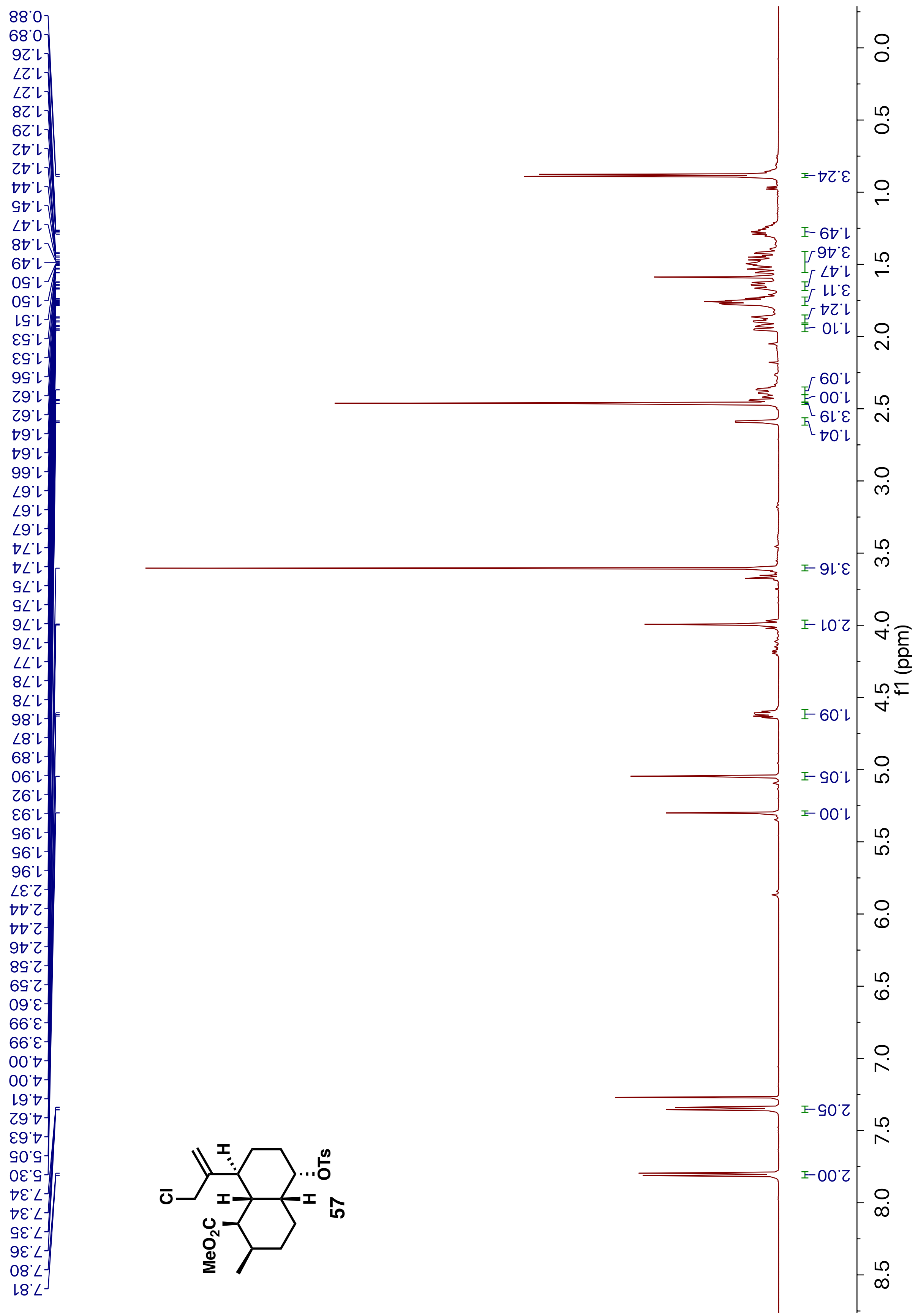
0.88
0.89
1.26
1.27
1.27
1.28
1.29
1.42
1.42
1.44
1.45
1.47
1.48
1.49
1.50
1.50
1.51
1.53
1.53
1.56
1.62
1.62
1.64
1.64
1.66
1.67
1.67
1.67
1.74
1.74
1.75
1.75
1.76
1.76
1.77
1.78
1.78
1.86
1.87
1.89
1.90
1.92
1.93
1.95
1.95
1.96
2.37
2.44
2.44
2.46
2.58
2.59
3.60
3.99
3.99
4.00
4.00
4.61
4.62
4.63
5.05
5.30
7.34
7.34
7.35
7.36
7.80
7.81
Cl
MeO2C
H
H
H
OTs
57
3.24
1.49
3.46
1.47
3.11
1.24
1.10
1.09
1.00
3.19
1.04
3.16
2.01
1.09
1.05
1.00
2.05
2.00
f1 (ppm)

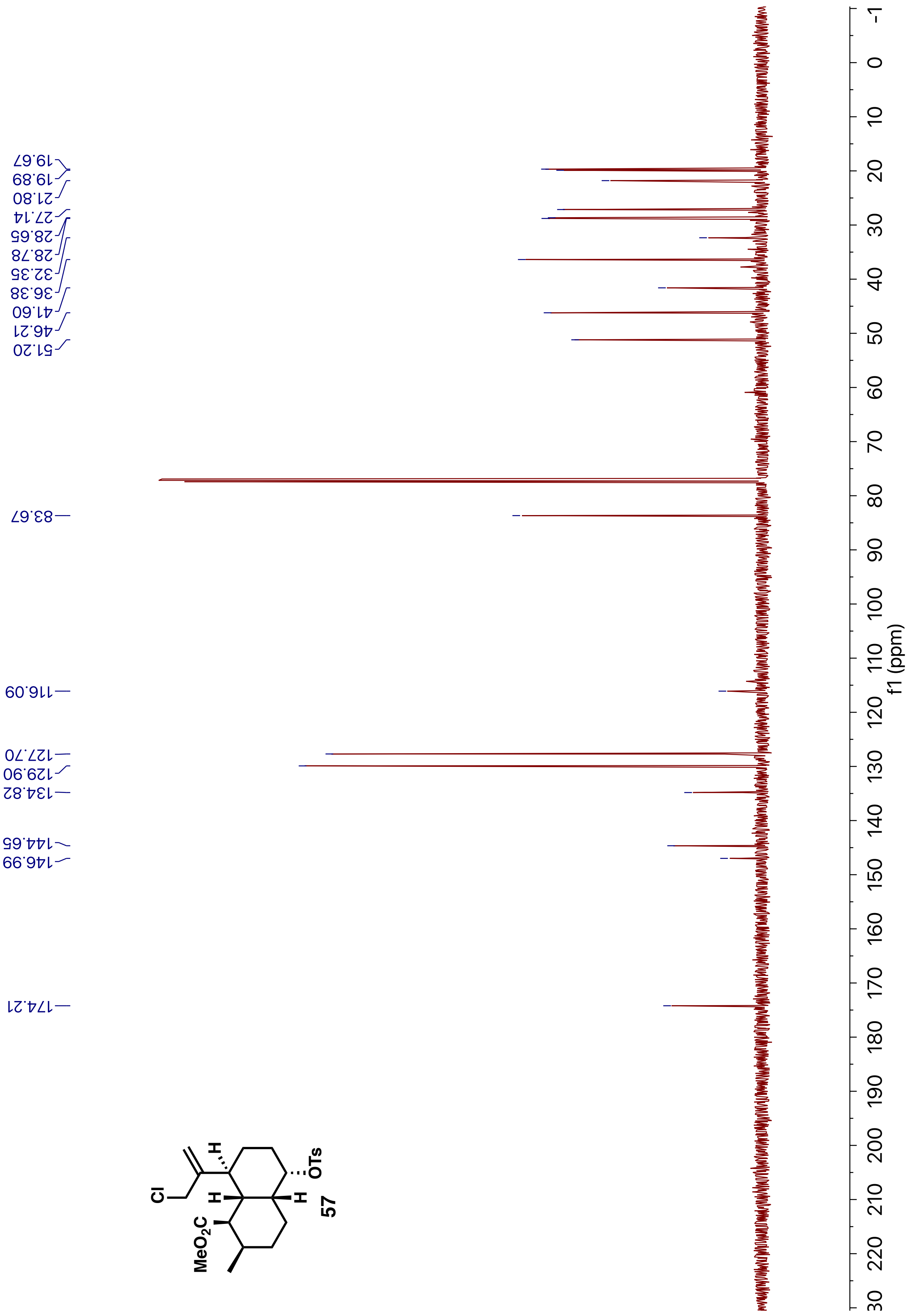
19.67
19.89
21.80
27.14
28.65
28.78
32.35
36.38
41.60
46.21
51.20
83.67
116.09
127.70
129.90
134.82
144.65
146.99
174.21
f1 (ppm)
MeO2C
Cl
OTs
57

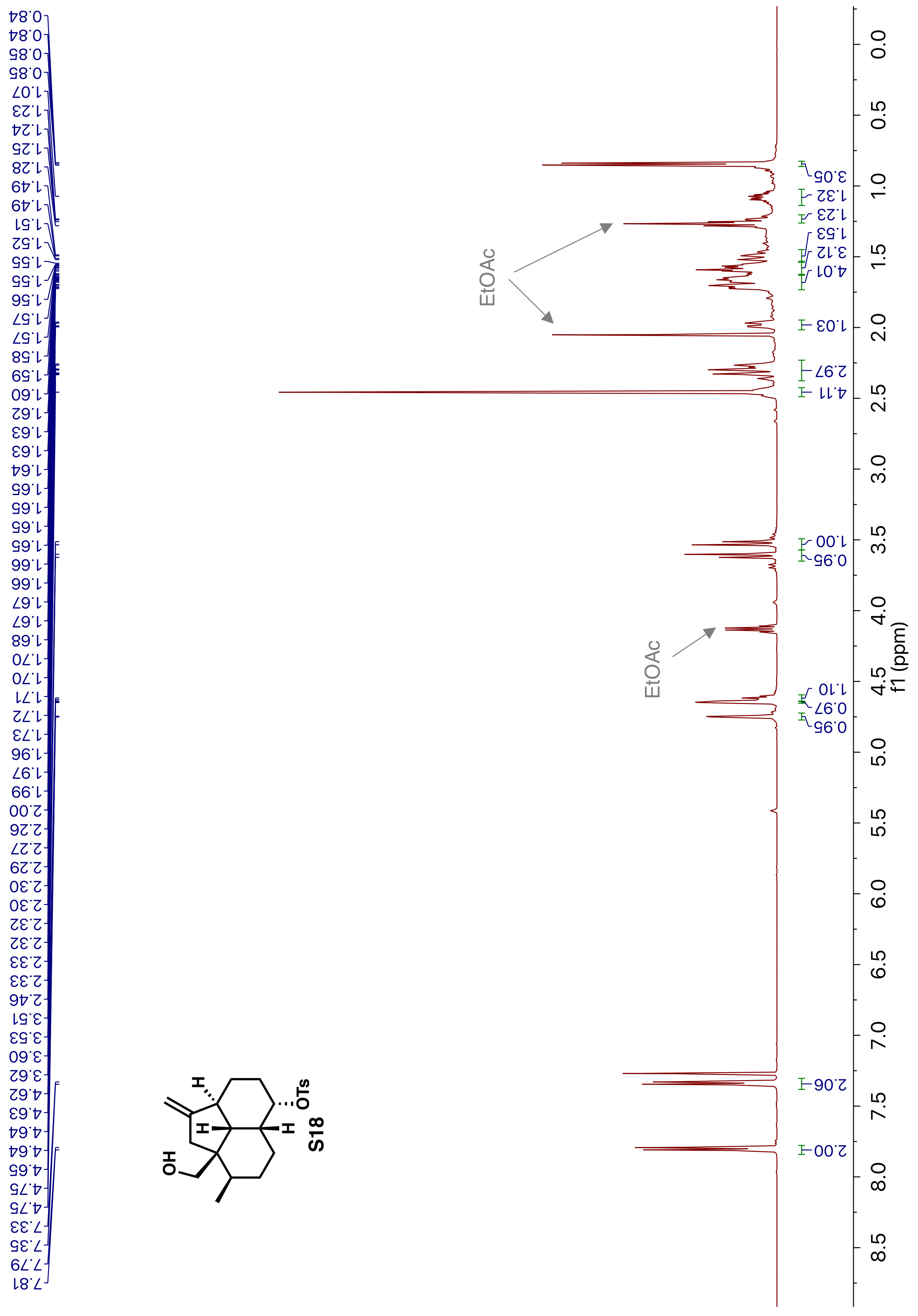

0.84
0.84
0.85
0.85
1.07
1.23
1.24
1.25
1.28
1.49
1.49
1.51
1.52
1.55
1.55
1.56
1.57
1.57
1.58
1.59
1.60
1.62
1.63
1.63
1.64
1.65
1.65
1.65
1.65
1.66
1.66
1.67
1.67
1.68
1.70
1.70
1.71
1.72
1.73
1.96
1.97
1.99
2.00
2.26
2.27
2.29
2.30
2.30
2.32
2.32
2.33
2.33
2.46
3.51
3.53
3.60
3.62
4.62
4.63
4.64
4.64
4.65
4.75
4.75
7.33
7.35
7.79
7.81
OH
H
H
H
OTs
S18
EtOAc
EtOAc
3.05
1.32
1.23
1.53
3.12
4.01
1.03
2.97
4.11
1.00
0.95
1.10
0.97
0.95
2.06
2.00
0.0
0.5
1.0
1.5
2.0
2.5
3.0
3.5
4.0
4.5
5.0
5.5
6.0
6.5
7.0
7.5
8.0
8.5
f1 (ppm)

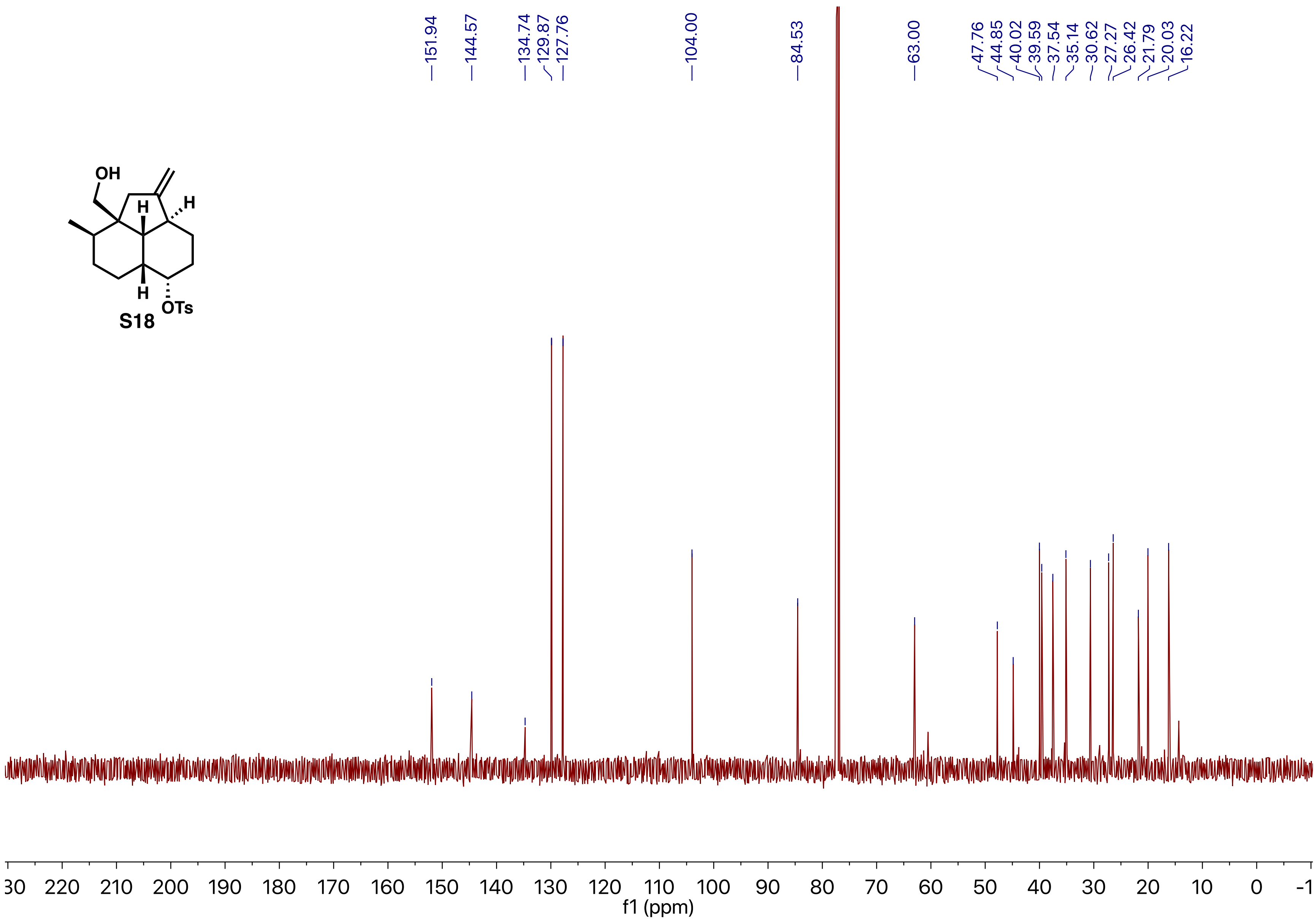
S18
151.94
144.57
134.74
129.87
127.76
104.00
84.53
63.00
47.76
44.85
40.02
39.59
37.54
35.14
30.62
27.27
26.42
21.79
20.03
16.22
230 220 210 200 190 180 170 160 150 140 130 120 110 100 90 80 70 60 50 40 30 20 10 0 -1
f1 (ppm)

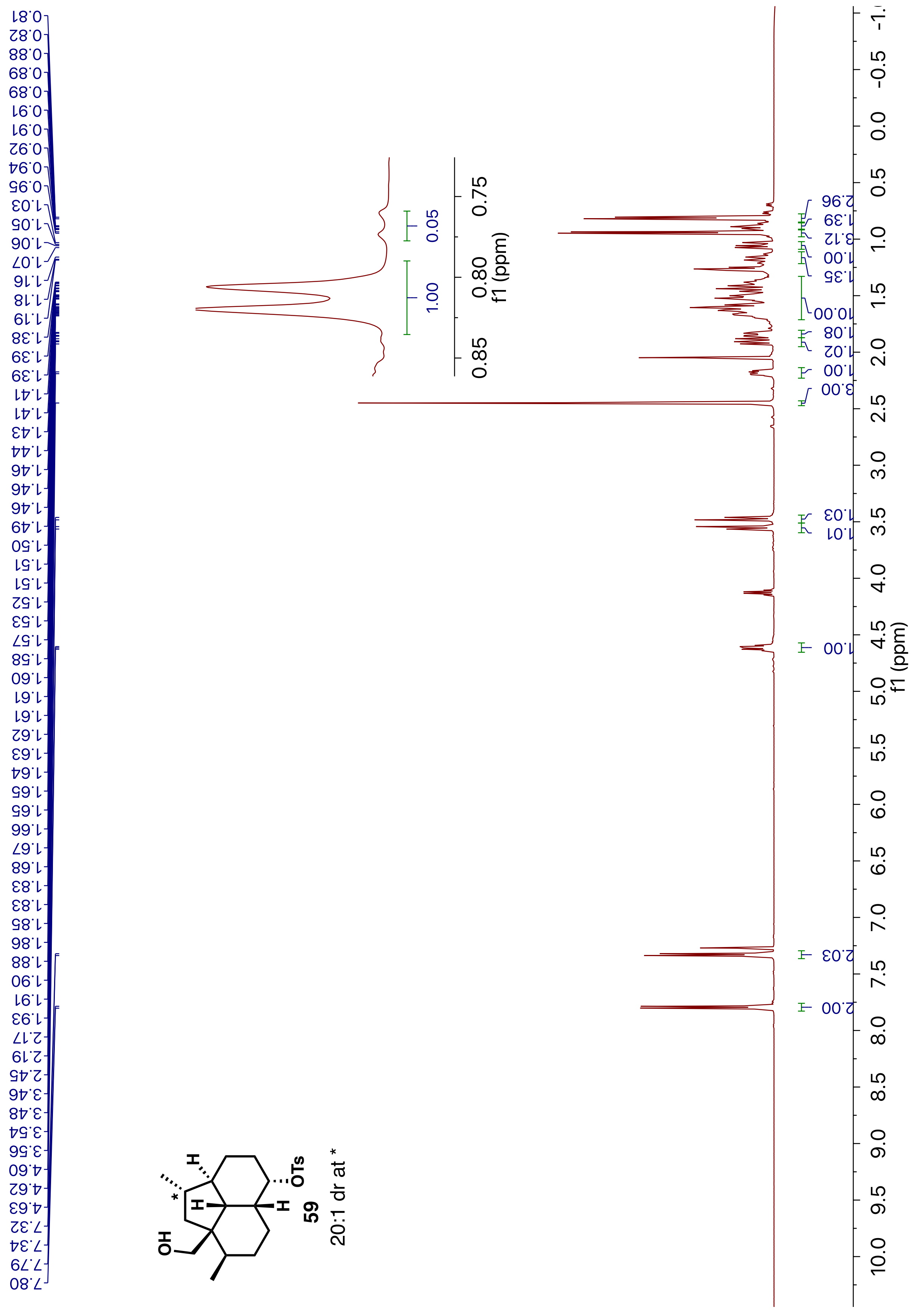
0.81 0.82 0.88 0.89 0.89 0.91 0.91 0.92 0.94 0.95 1.03 1.05 1.06 1.07 1.16 1.18 1.19 1.38 1.39 1.39 1.41 1.41 1.43 1.44 1.46 1.46 1.46 1.49 1.50 1.51 1.51 1.52 1.53 1.57 1.58 1.60 1.61 1.61 1.62 1.63 1.64 1.65 1.65 1.66 1.67 1.68 1.83 1.83 1.85 1.86 1.88 1.90 1.91 1.93 2.17 2.19 2.45 3.46 3.48 3.54 3.56 4.60 4.62 4.63 7.32 7.34 7.79 7.80
OH
H
H
H
OTs
*
59
20:1 dr at *
0.05
1.00
0.75
0.80
0.85
f1 (ppm)
2.96 1.39 3.12 1.00 1.35 10.00 1.08 1.02 1.00 3.00 1.03 1.01 1.00 2.03 2.00
10.0 9.5 9.0 8.5 8.0 7.5 7.0 6.5 6.0 5.5 5.0 4.5 4.0 3.5 3.0 2.5 2.0 1.5 1.0 0.5 0.0 -0.5
f1 (ppm)

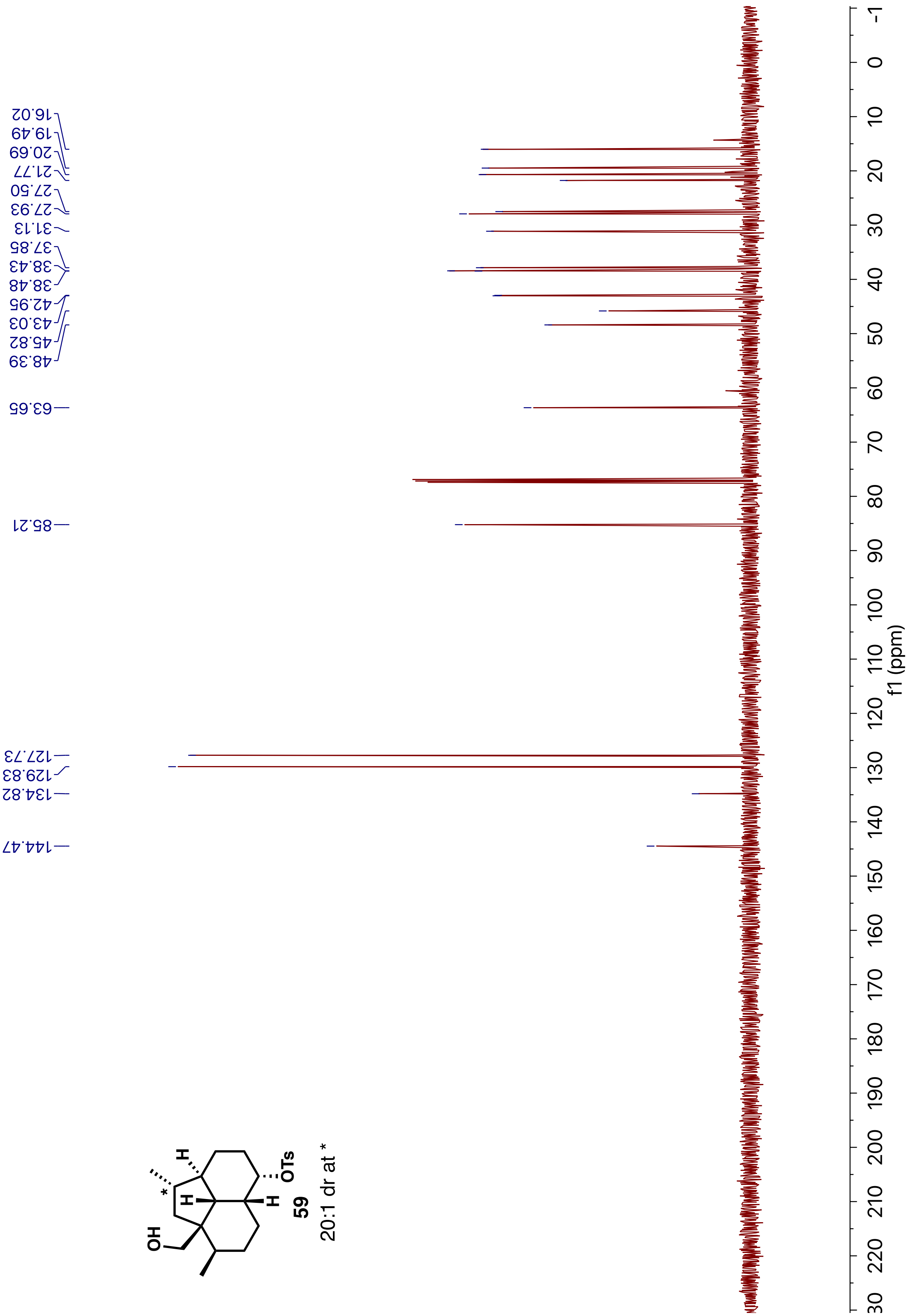
16.02
19.49
20.69
21.77
27.50
27.93
31.13
37.85
38.43
38.48
42.95
43.03
45.82
48.39
63.65
85.21
127.73
129.83
134.82
144.47
f1 (ppm)
OH
H
H
H
OTs
59
20:1 dr at *

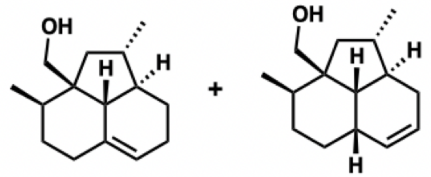

53 + **S19**

4:1 mixture of isomers)
(53 to S19)

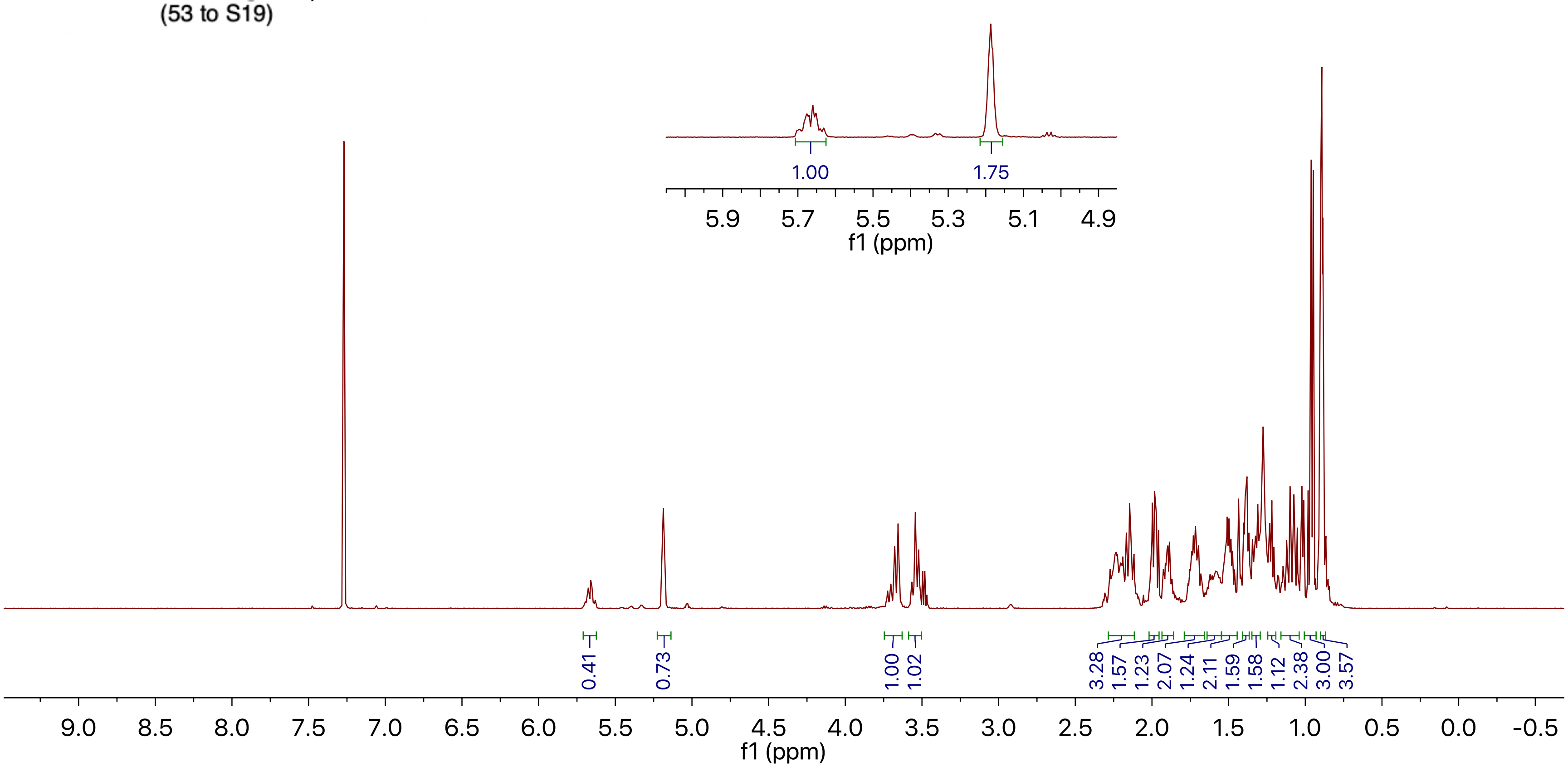

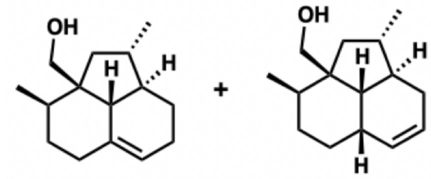

53　　**S19**

4:1 mixture of isomers)
(53 to S19)

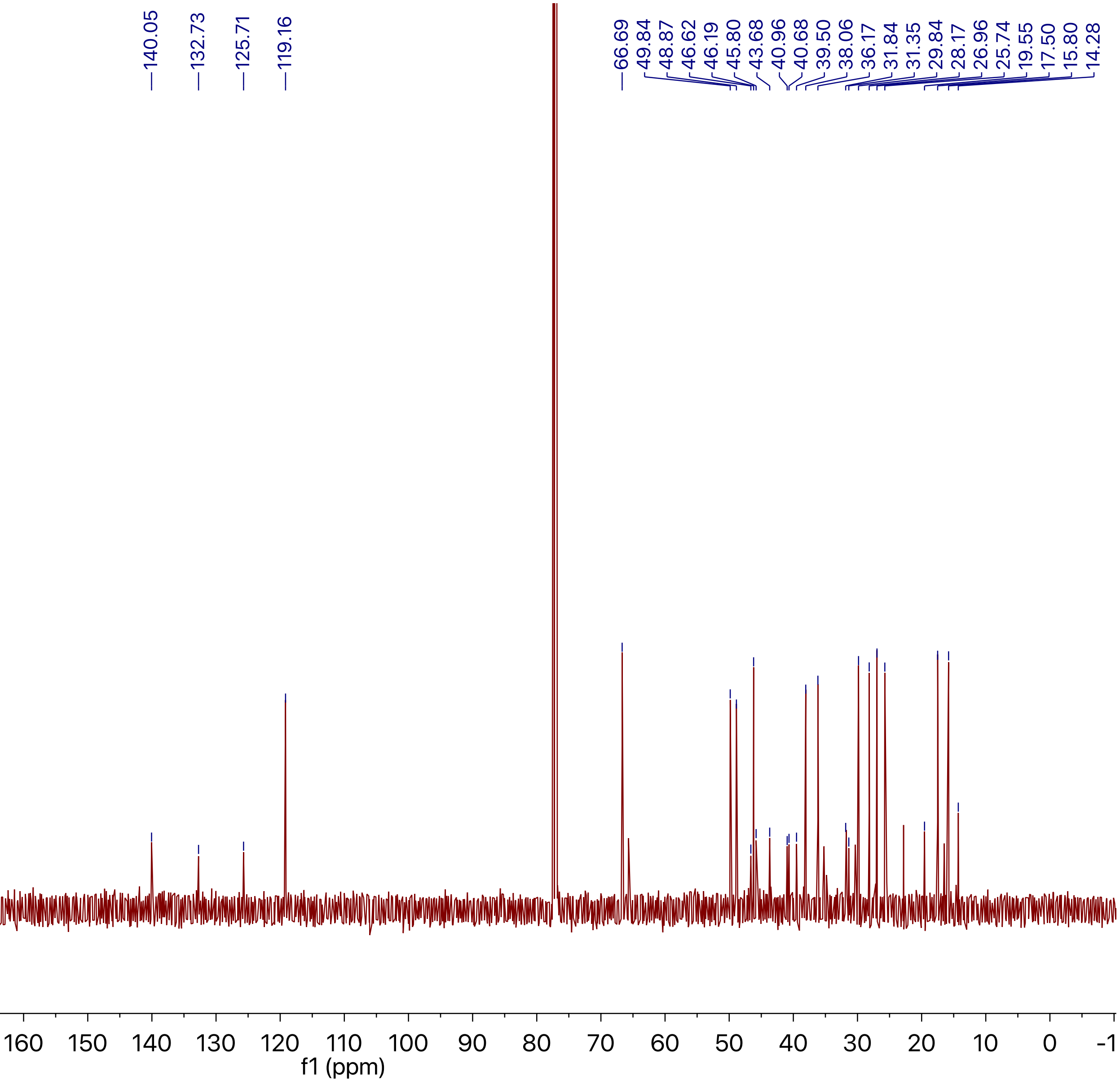

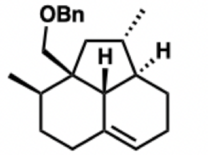

S20
(containing 20%
other alkene isomer)

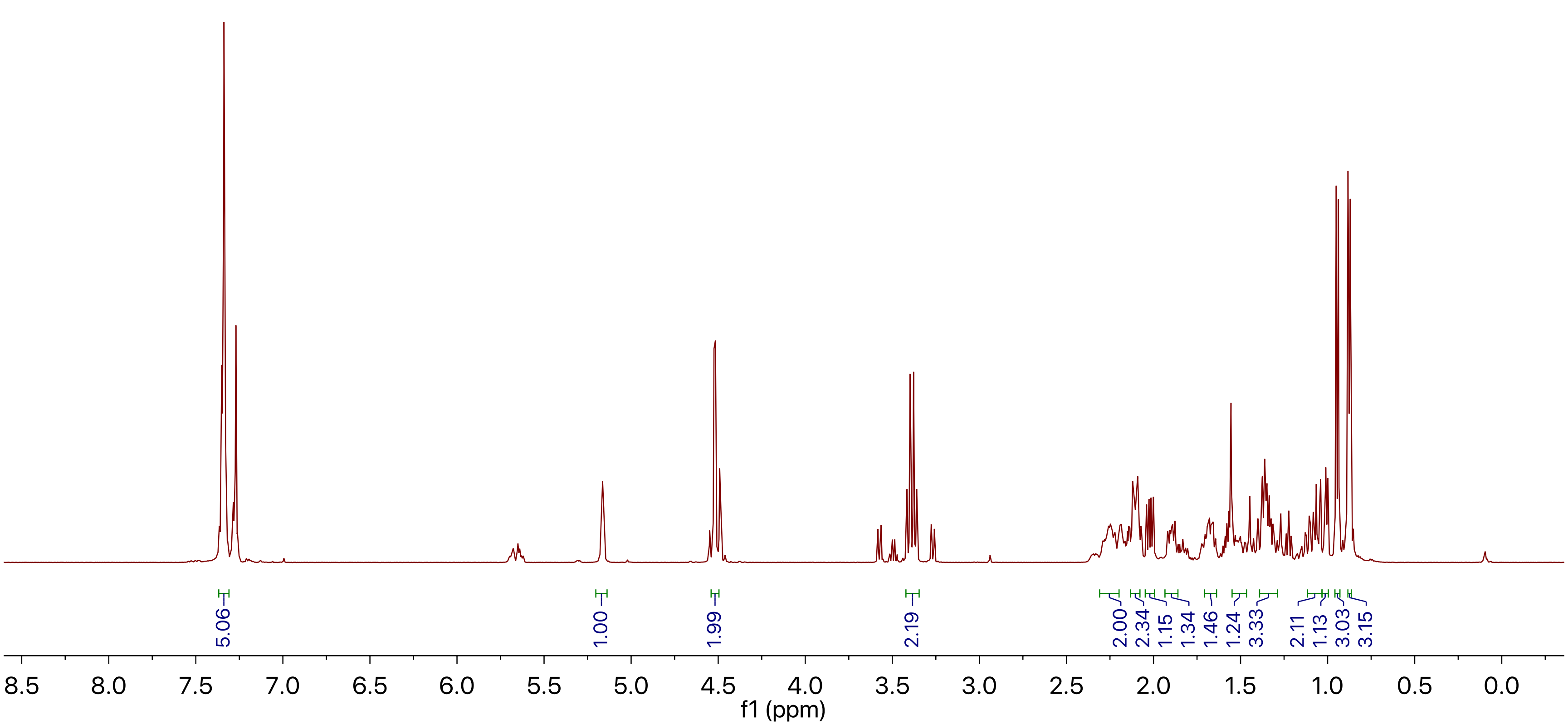

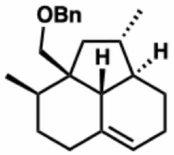

S20
(containing 20%
other alkene isomer)

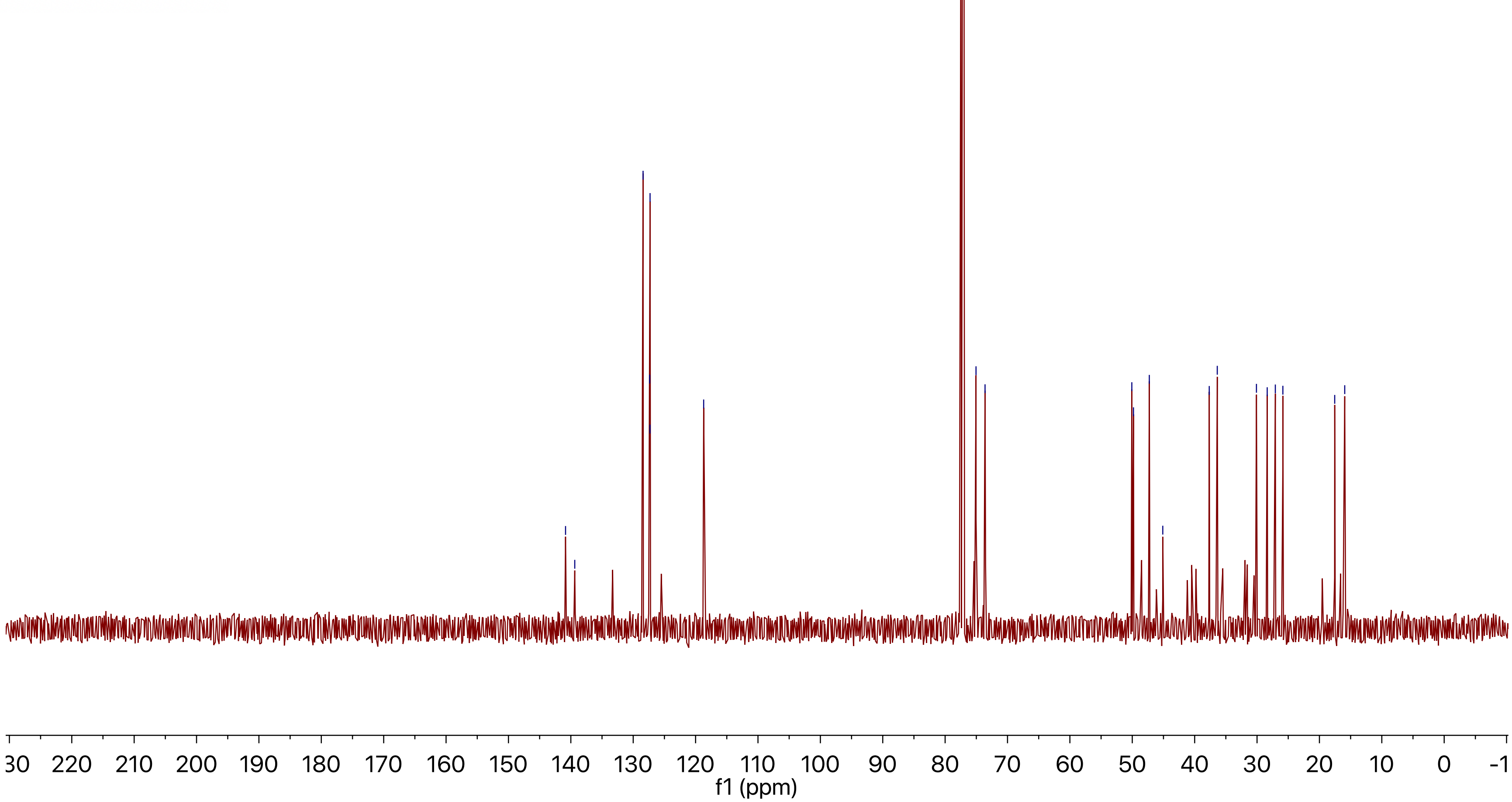

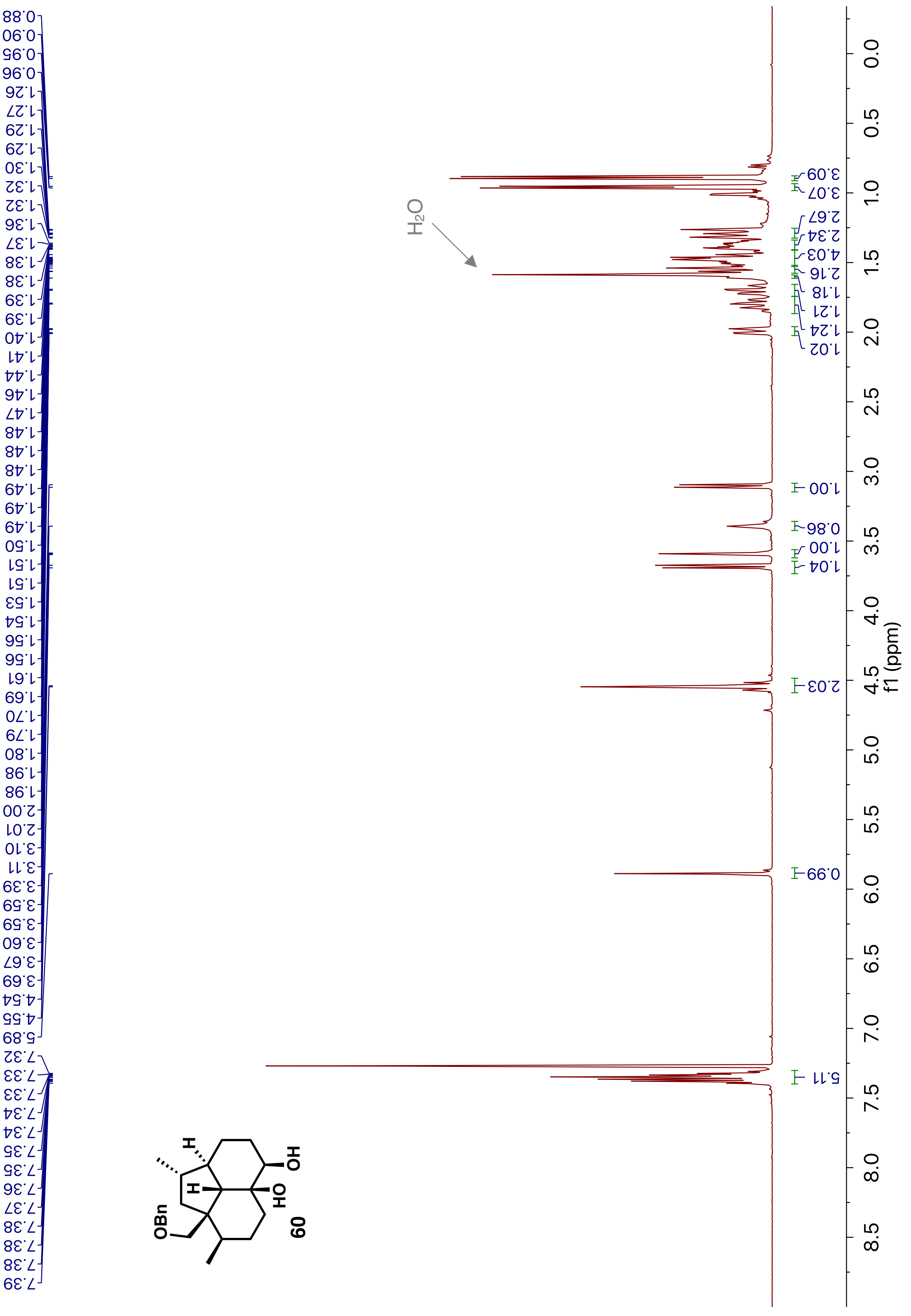

0.88
0.90
0.95
0.96
1.26
1.27
1.29
1.29
1.30
1.32
1.32
1.36
1.37
1.38
1.38
1.39
1.39
1.40
1.41
1.44
1.46
1.47
1.48
1.48
1.48
1.49
1.49
1.49
1.50
1.51
1.51
1.53
1.54
1.56
1.56
1.61
1.69
1.70
1.76
1.79
1.80
1.98
1.98
2.00
2.01
3.10
3.11
3.39
3.59
3.59
3.60
3.67
3.69
4.54
4.55
5.89
7.32
7.33
7.33
7.34
7.34
7.35
7.36
7.36
7.37
7.38
7.38
7.38
7.39
OBn
H
H
HO
OH
60
H2O
3.09
3.07
2.67
2.34
4.03
2.16
1.18
1.21
1.24
1.02
1.00
0.86
1.00
1.04
2.03
0.99
5.11
0.0
0.5
1.0
1.5
2.0
2.5
3.0
3.5
4.0
4.5
5.0
5.5
6.0
6.5
7.0
7.5
8.0
8.5
f1 (ppm)

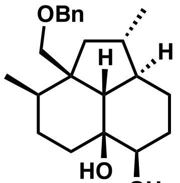
OBn
H
H
HO
OH

60

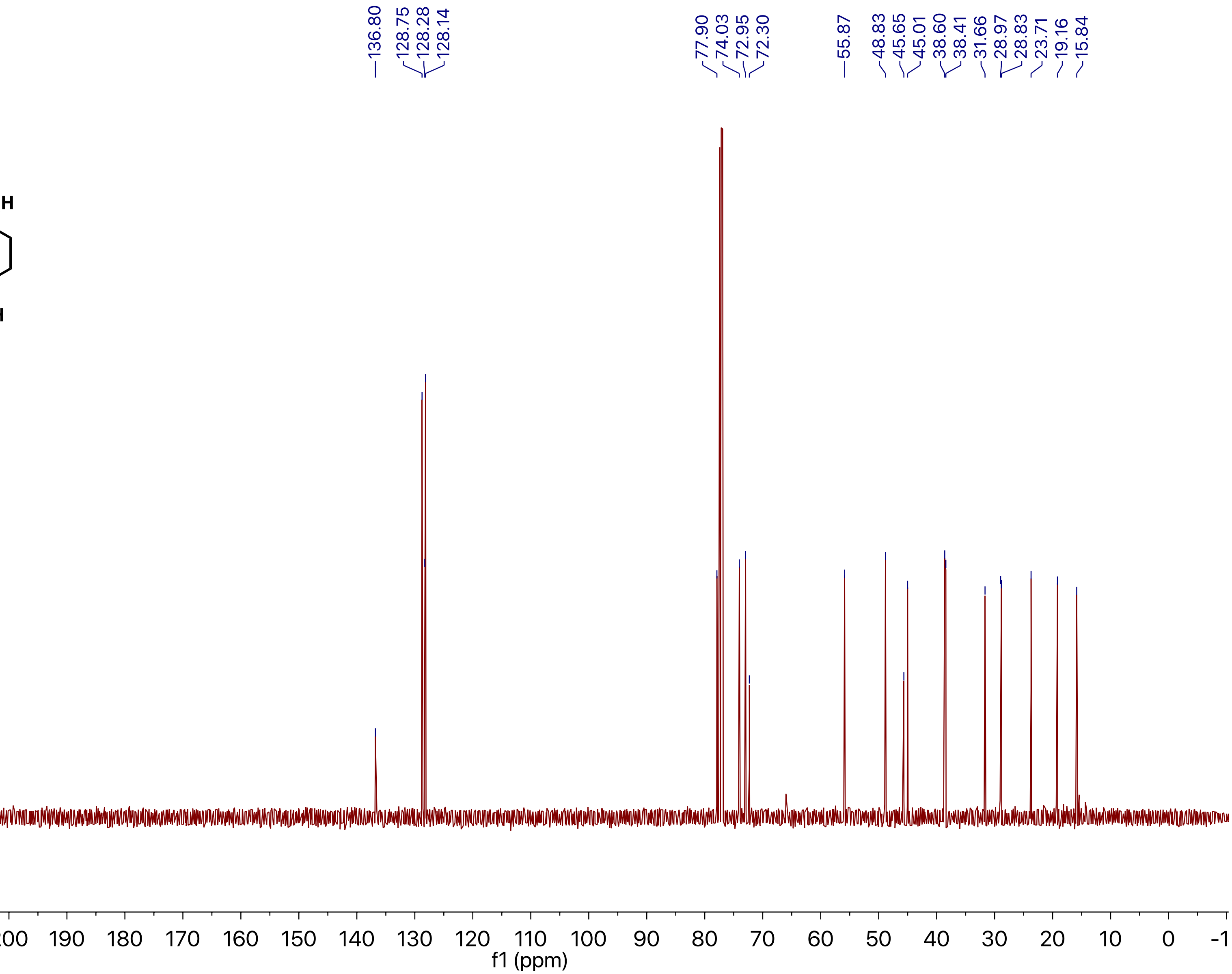
136.80
128.75
128.28
128.14
77.90
74.03
72.95
72.30
55.87
48.83
45.65
45.01
38.60
38.41
31.66
28.97
28.83
23.71
19.16
15.84
230 220 210 200 190 180 170 160 150 140 130 120 110 100 90 80 70 60 50 40 30 20 10 0 -1
f1 (ppm)

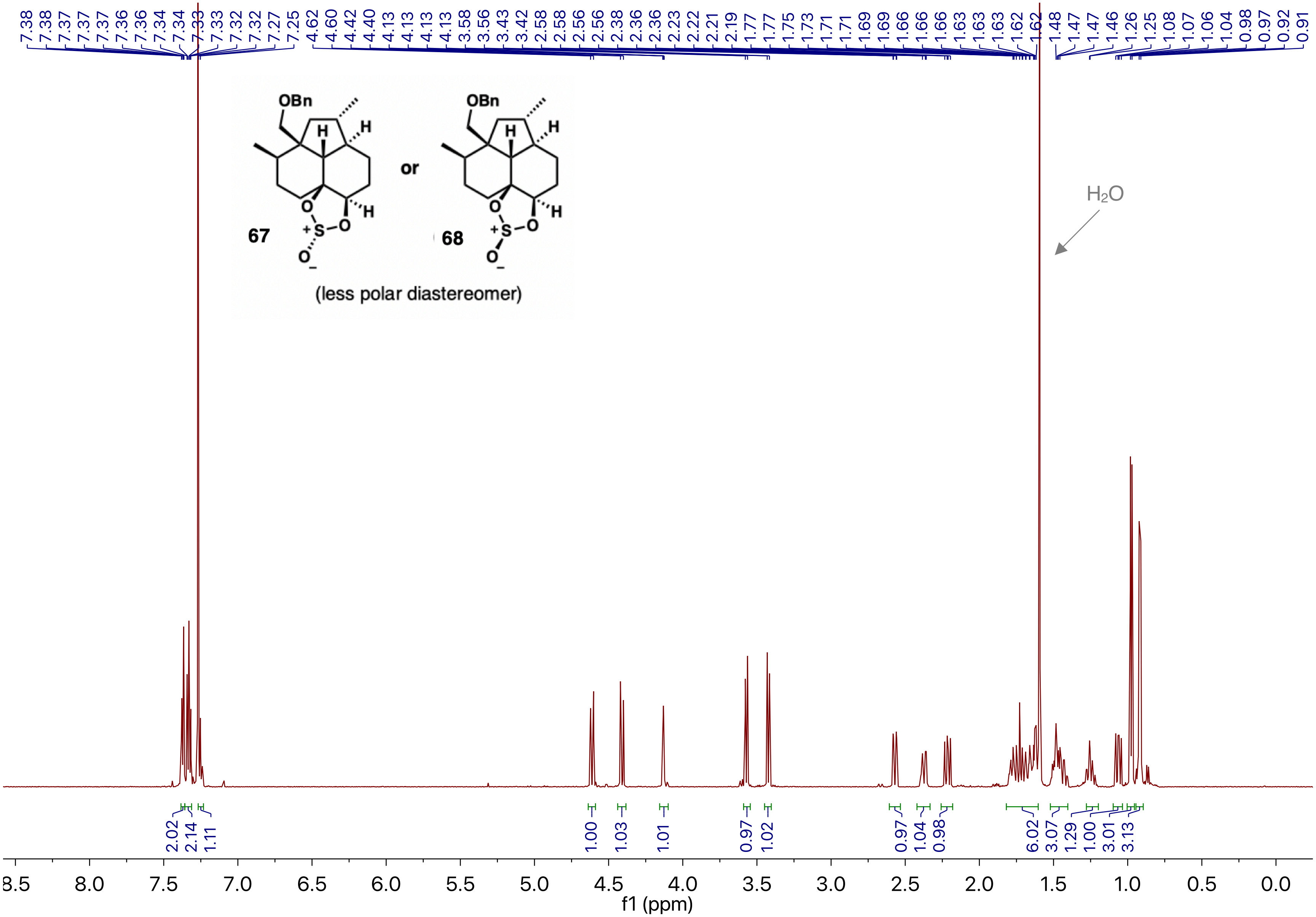
7.38 7.38 7.37 7.37 7.37 7.36 7.36 7.34 7.34 7.33 7.33 7.32 7.32 7.27 7.25 4.62 4.60 4.42 4.40 4.13 4.13 4.13 4.13 3.58 3.56 3.43 3.42 2.58 2.58 2.56 2.56 2.38 2.36 2.36 2.23 2.22 2.21 2.19 1.77 1.77 1.75 1.73 1.71 1.71 1.69 1.69 1.66 1.66 1.66 1.63 1.63 1.63 1.62 1.62 1.48 1.47 1.47 1.46 1.26 1.25 1.08 1.07 1.06 1.04 0.98 0.97 0.92 0.91
OBn
67
or
68
(less polar diastereomer)
H2O
2.02 2.14 1.11 1.00 1.03 1.01 0.97 1.02 0.97 1.04 0.98 6.02 3.07 1.29 1.00 3.01 3.13
8.5 8.0 7.5 7.0 6.5 6.0 5.5 5.0 4.5 4.0 3.5 3.0 2.5 2.0 1.5 1.0 0.5 0.0
f1 (ppm)

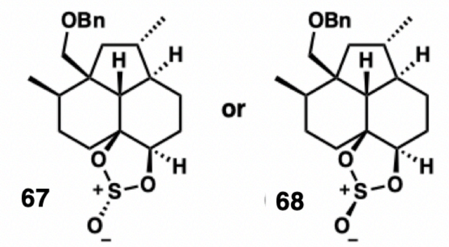

(less polar diastereomer)

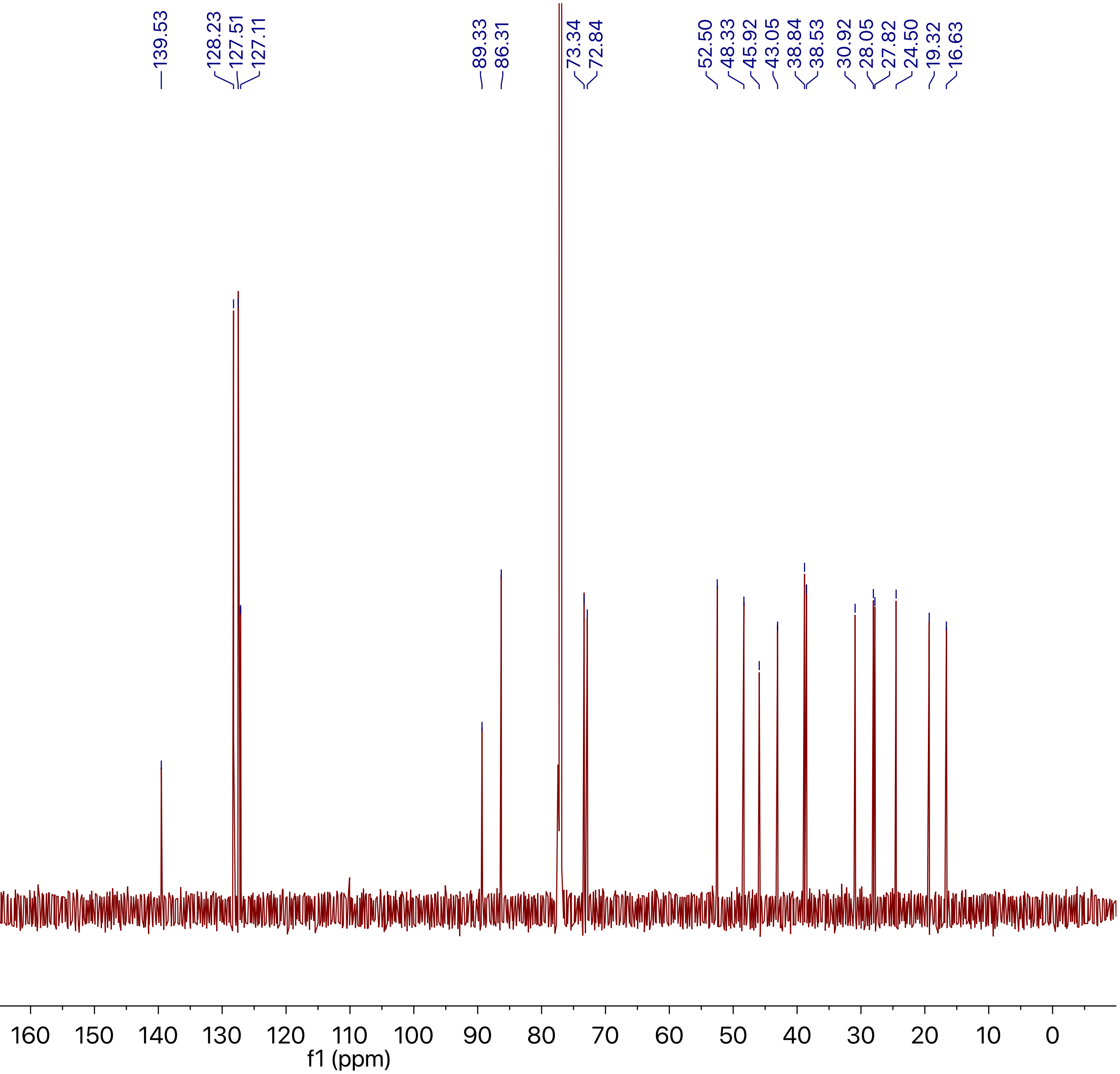

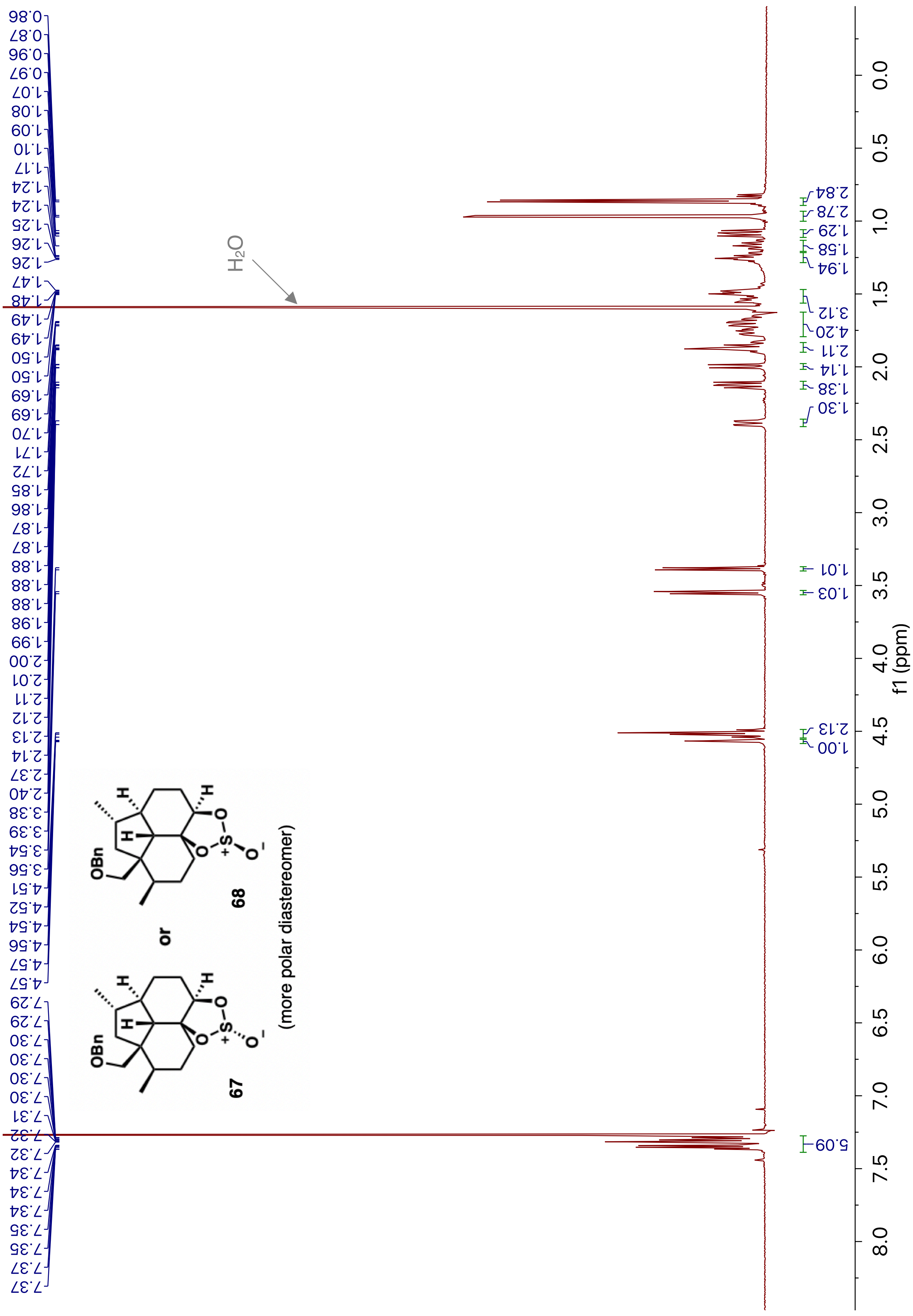

OBn
67
or
OBn
68
(more polar diastereomer)
H2O
f1 (ppm)

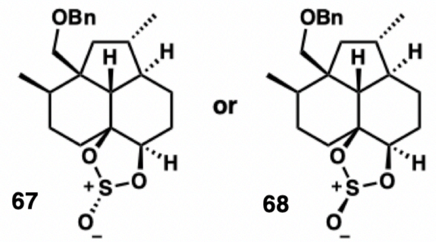

67 68

(more polar diastereomer)

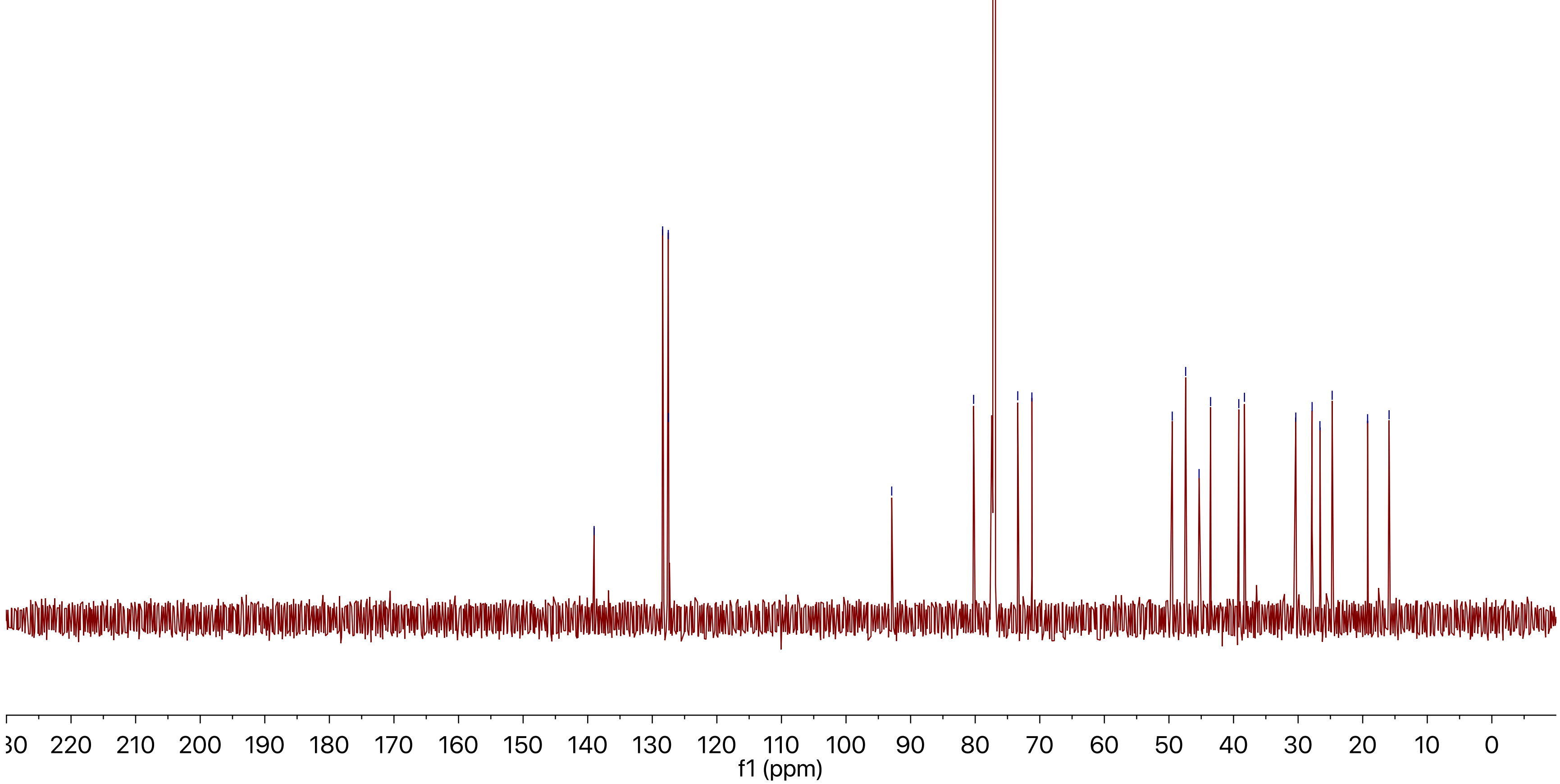

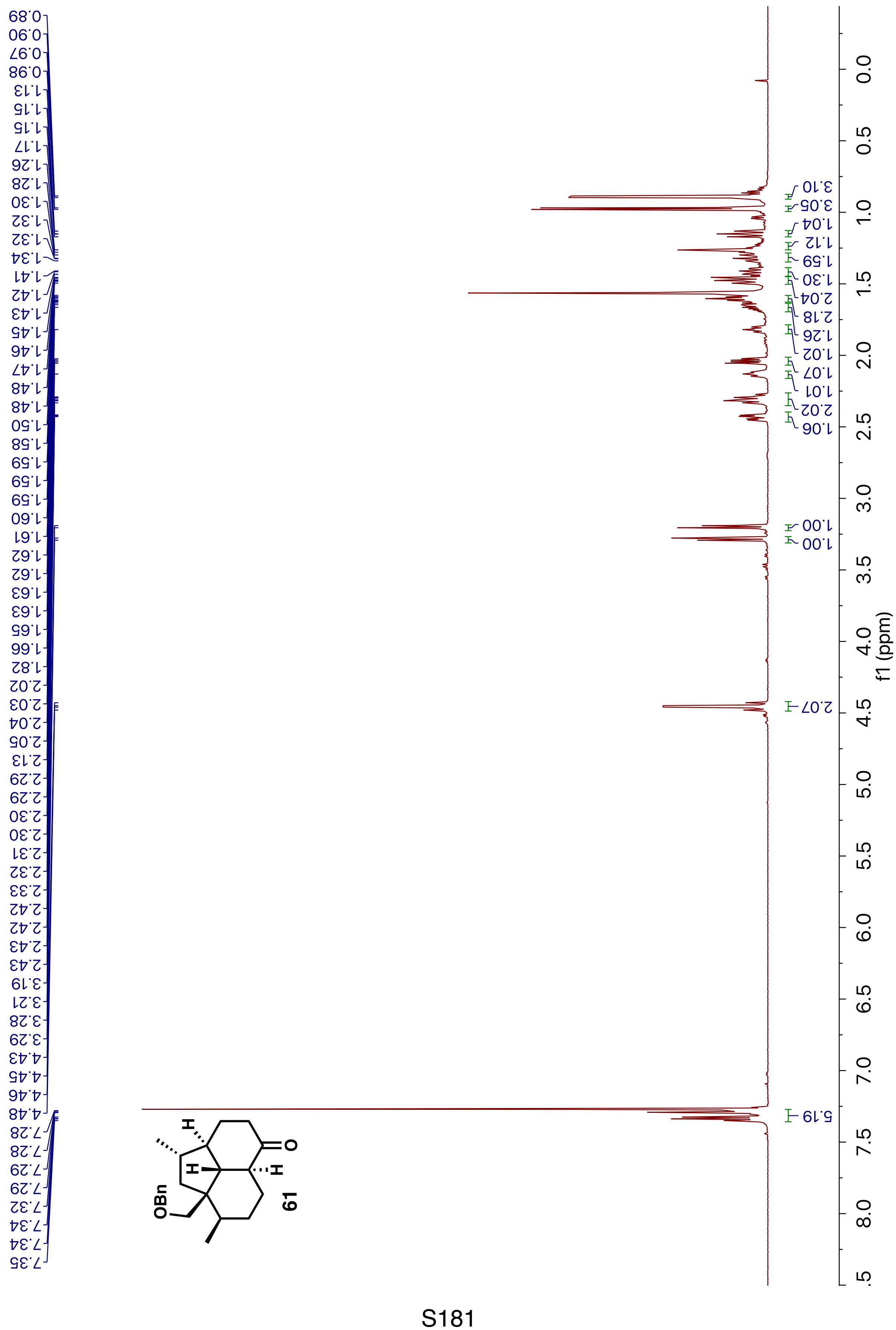
0.89
0.90
0.97
0.98
1.13
1.15
1.15
1.17
1.26
1.28
1.30
1.32
1.32
1.34
1.41
1.42
1.43
1.45
1.46
1.47
1.48
1.48
1.50
1.58
1.59
1.59
1.59
1.60
1.61
1.62
1.62
1.63
1.63
1.65
1.66
1.82
2.02
2.03
2.04
2.05
2.13
2.29
2.29
2.30
2.30
2.31
2.32
2.33
2.42
2.42
2.43
2.43
3.19
3.21
3.28
3.29
4.43
4.45
4.46
4.48
7.28
7.28
7.29
7.29
7.32
7.34
7.34
7.35
OBn
H
H
H
O
61
3.10
3.05
1.04
1.12
1.59
1.30
2.04
2.18
1.26
1.02
1.07
1.01
2.02
1.06
1.00
1.00
2.07
5.19
f1 (ppm)

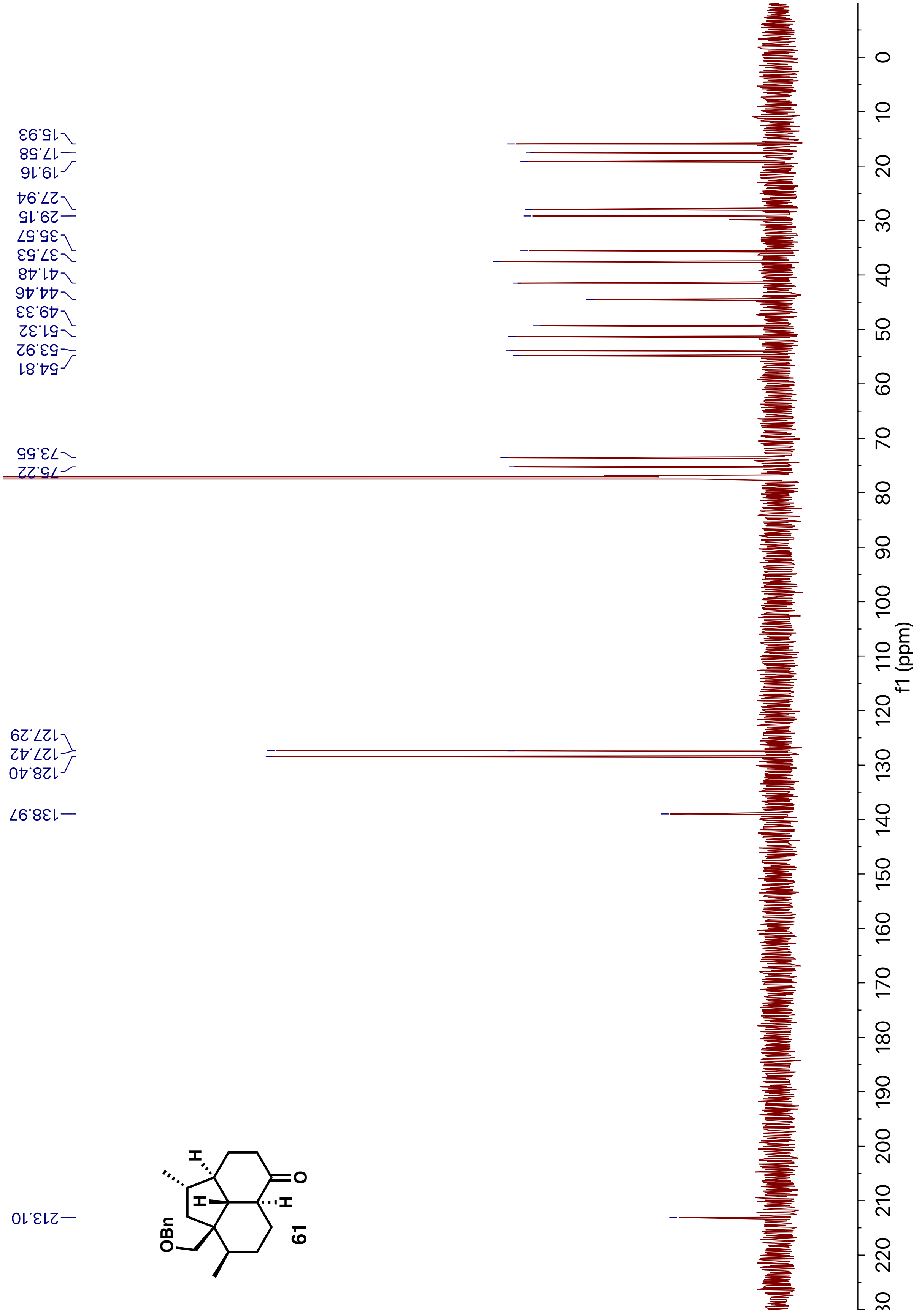

15.93
17.58
19.16
27.94
29.15
35.57
37.53
41.48
44.46
49.33
51.32
53.92
54.81
73.55
75.22
127.29
127.42
128.40
138.97
213.10
OBn
61
f1 (ppm)

DEPTQ

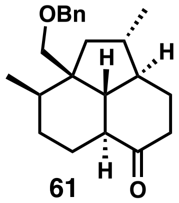
OBn
H
H
H
O
61

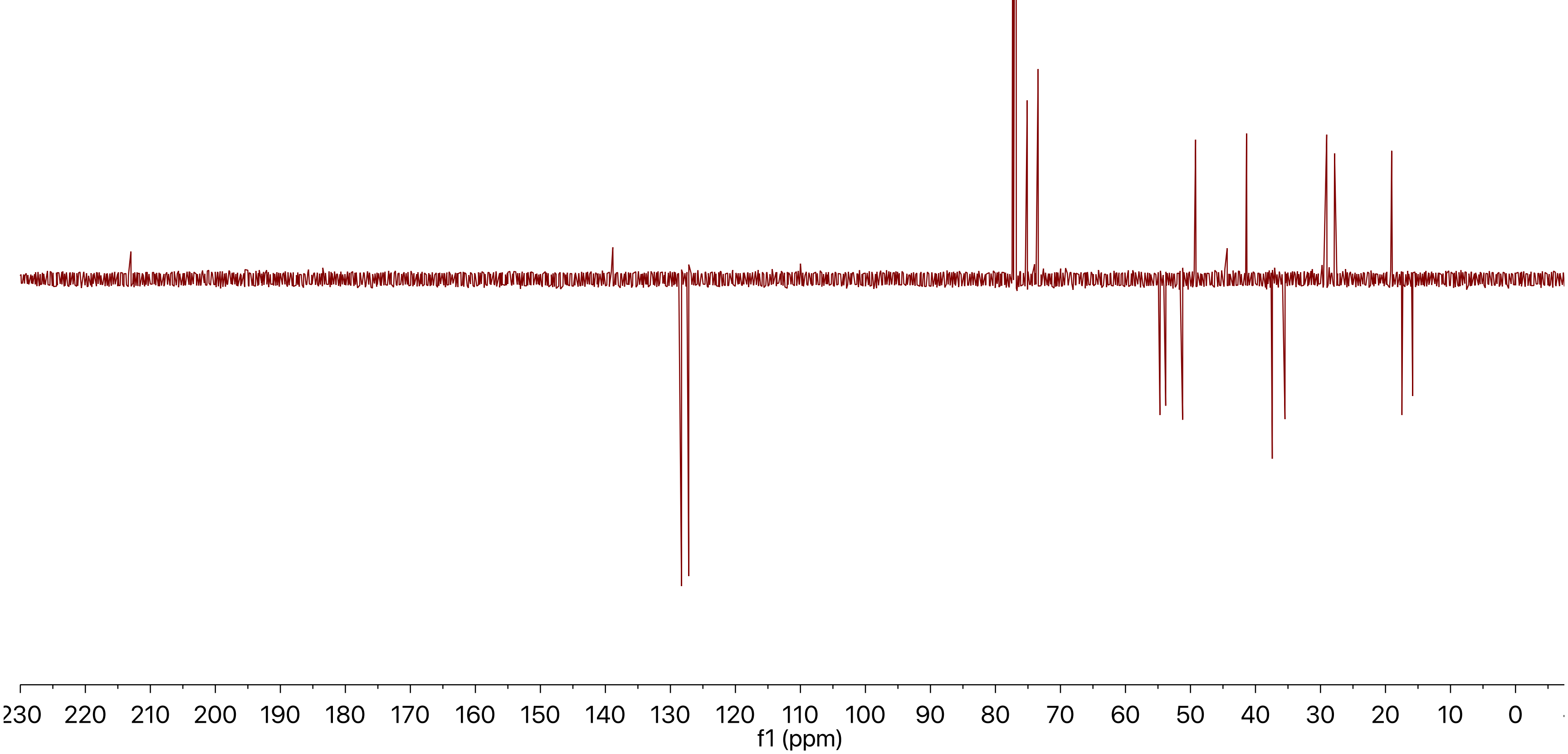
230
220
210
200
190
180
170
160
150
140
130
120
110
100
90
80
70
60
50
40
30
20
10
0
f1 (ppm)

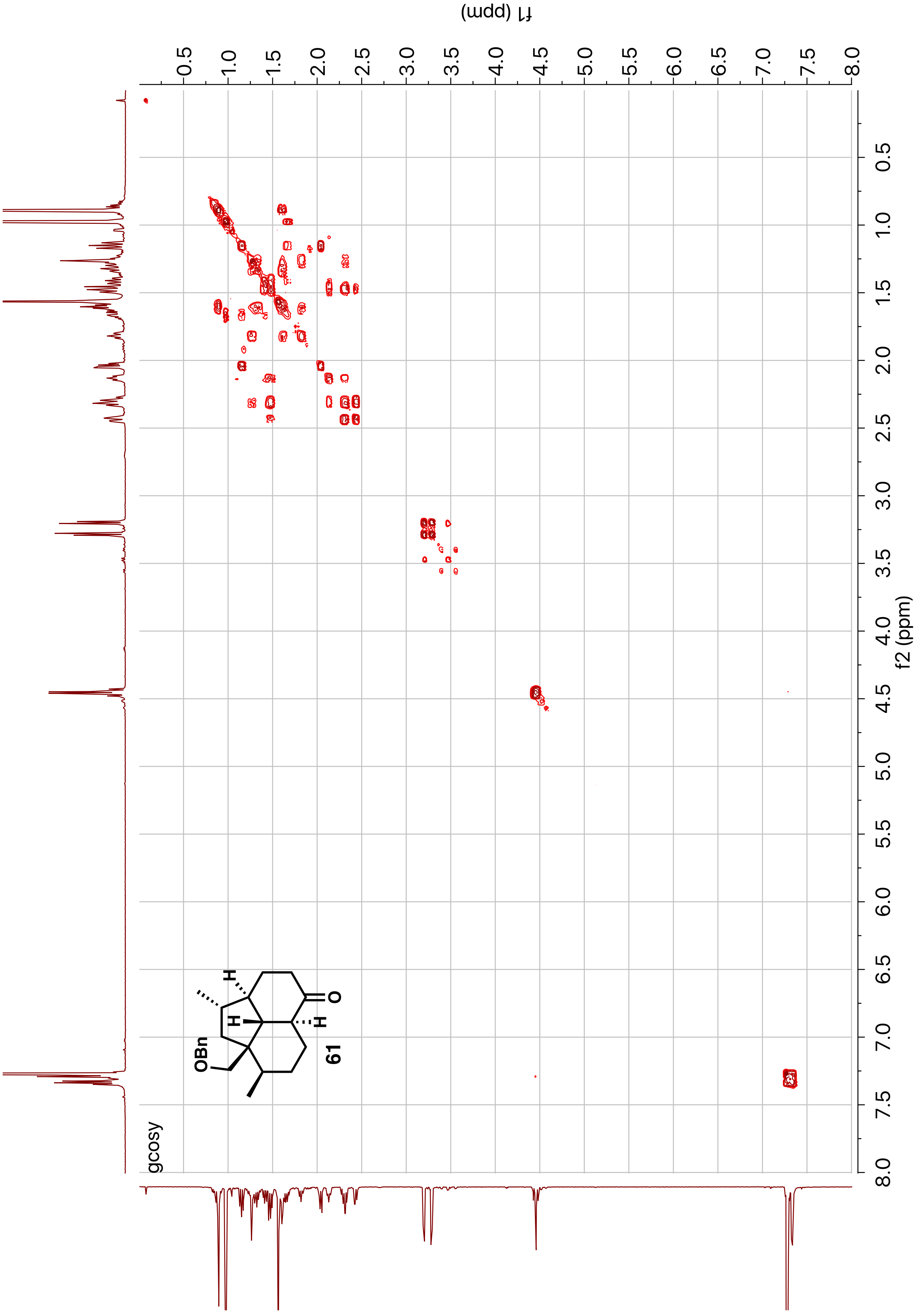
gcosy
f1 (ppm)
f2 (ppm)
OBn
H
H
H
O
61

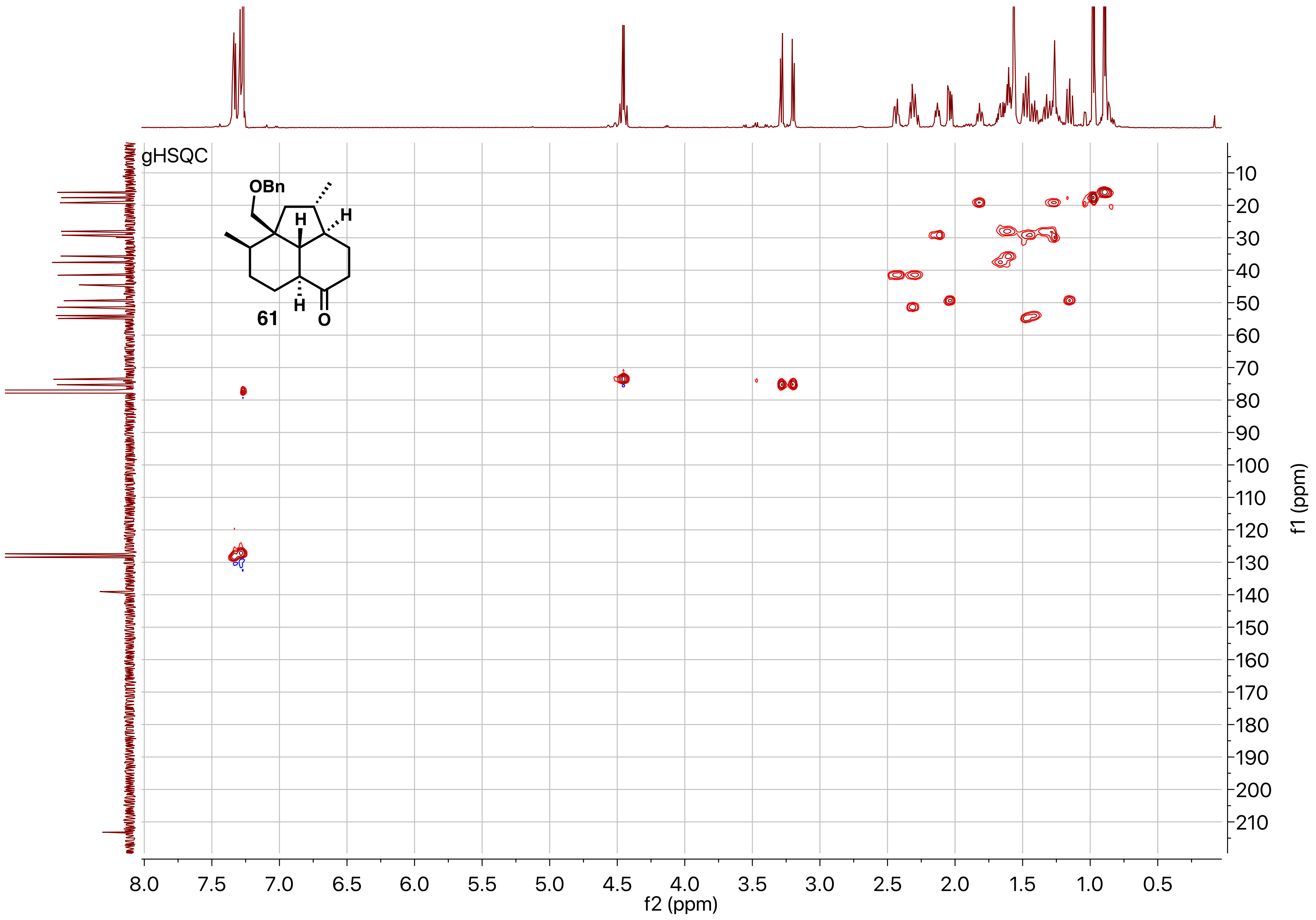
gHSQC
OBn
H
H
H
O
61
f2 (ppm)
f1 (ppm)
8.0 7.5 7.0 6.5 6.0 5.5 5.0 4.5 4.0 3.5 3.0 2.5 2.0 1.5 1.0 0.5
10 20 30 40 50 60 70 80 90 100 110 120 130 140 150 160 170 180 190 200 210

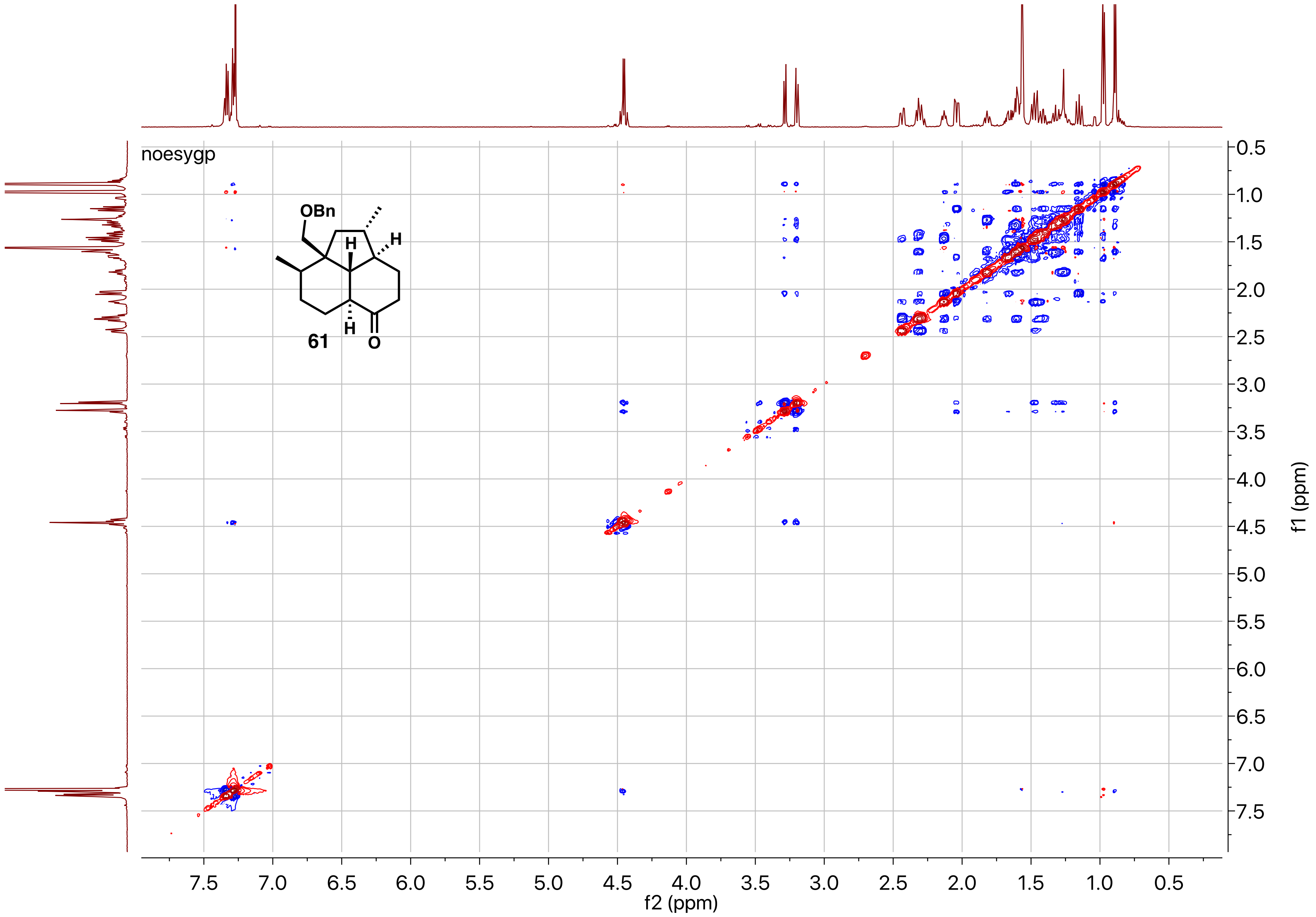
noesygp
OBn
H
H
H
O
61
f2 (ppm)
f1 (ppm)

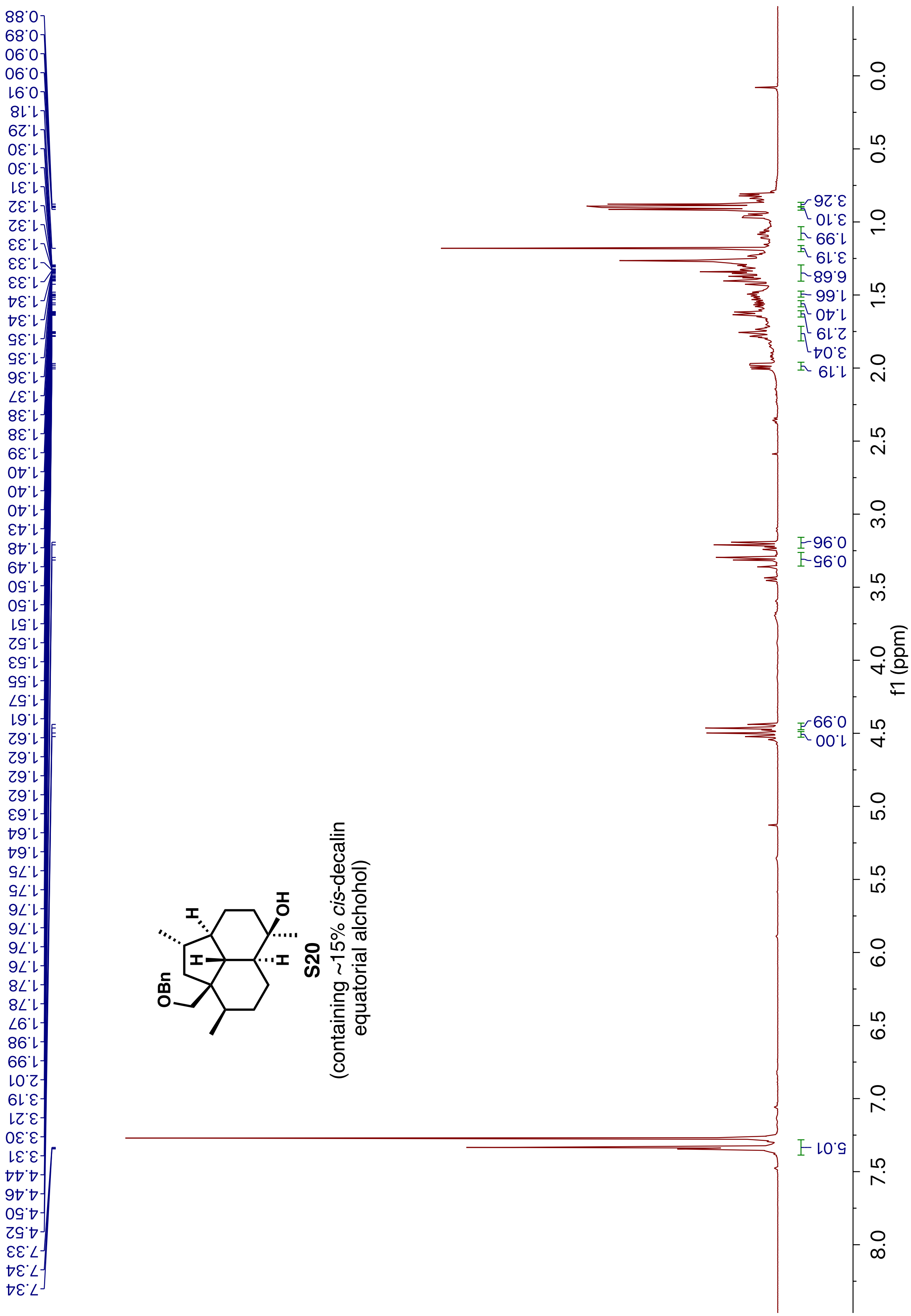

0.88
0.89
0.90
0.90
0.91
1.18
1.29
1.30
1.30
1.31
1.32
1.32
1.33
1.33
1.33
1.34
1.34
1.35
1.35
1.36
1.37
1.38
1.38
1.39
1.40
1.40
1.40
1.43
1.48
1.49
1.50
1.50
1.51
1.52
1.53
1.55
1.57
1.61
1.62
1.62
1.62
1.62
1.63
1.64
1.64
1.75
1.75
1.76
1.76
1.76
1.78
1.78
1.97
1.98
1.99
2.01
3.19
3.21
3.30
3.31
4.44
4.46
4.50
4.52
7.33
7.34
7.34
OBn
H
H
H
OH
S20
(containing ~15% *cis*-decalin
equatorial alchohol)
3.26
3.10
1.99
3.19
6.68
1.66
1.40
2.19
3.04
1.19
0.96
0.95
0.99
1.00
5.01
f1 (ppm)

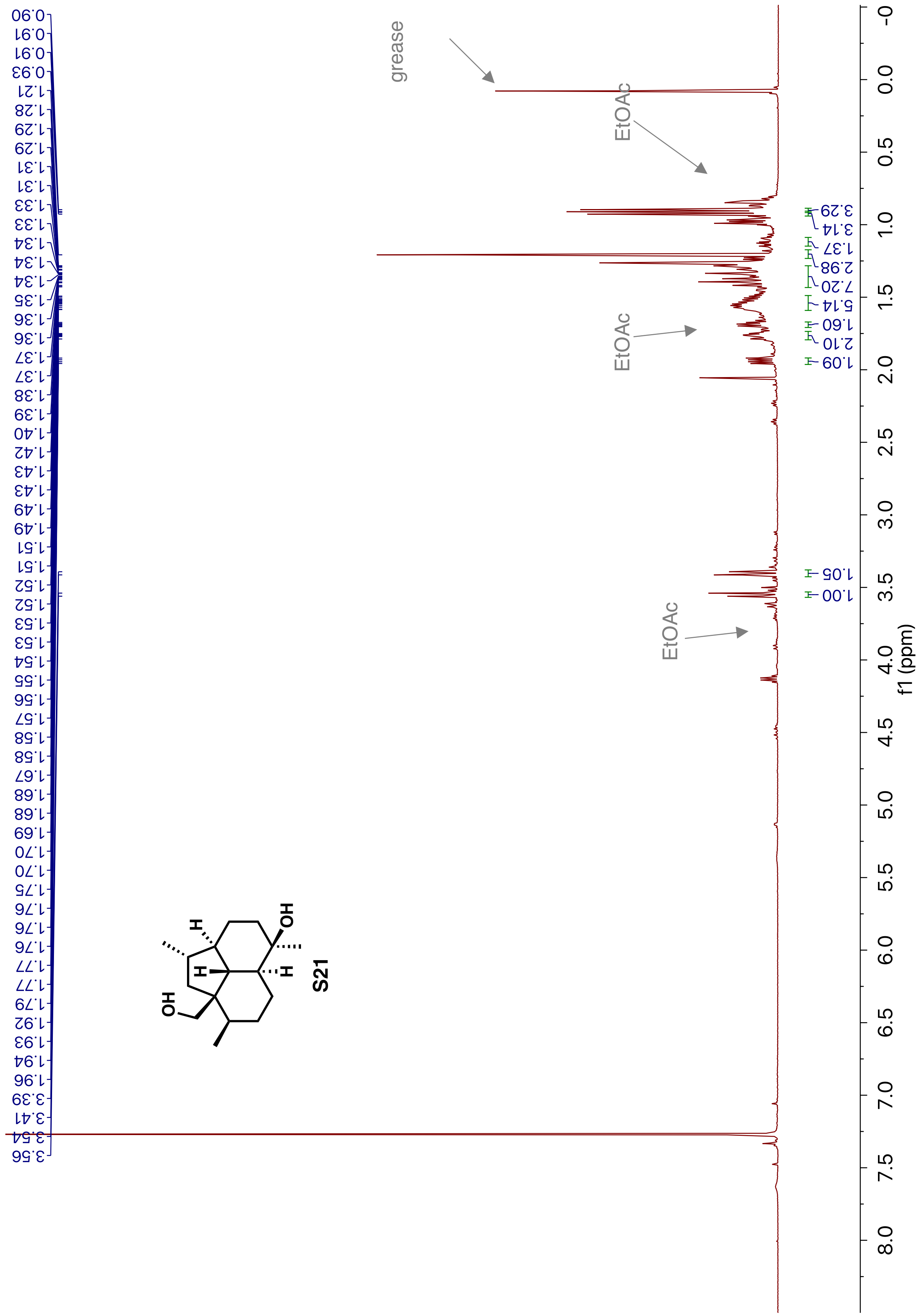
grease
EtOAc
EtOAc
EtOAc
S21
f1 (ppm)

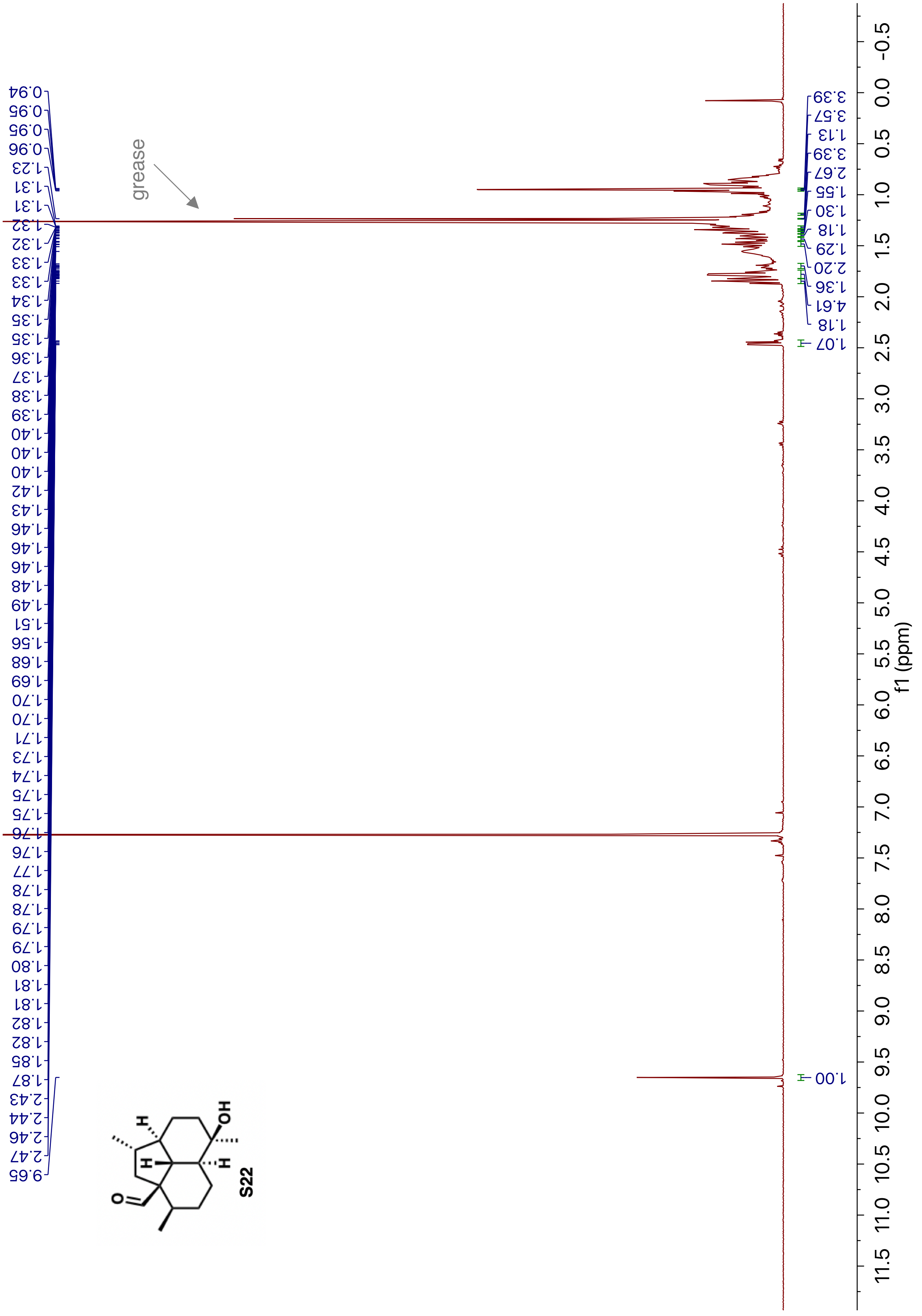
0.94
0.95
0.95
0.96
1.23
1.31
1.31
1.32
1.32
1.33
1.33
1.34
1.35
1.35
1.36
1.37
1.38
1.39
1.40
1.40
1.40
1.42
1.43
1.46
1.46
1.46
1.48
1.49
1.51
1.56
1.68
1.69
1.70
1.70
1.71
1.73
1.74
1.75
1.75
1.76
1.76
1.77
1.78
1.78
1.79
1.79
1.80
1.81
1.81
1.82
1.82
1.85
1.87
2.43
2.44
2.46
2.47
9.65
grease
3.39
3.57
1.13
3.39
2.67
1.55
1.30
1.18
1.29
2.20
1.36
4.61
1.18
1.07
1.00
f1 (ppm)
S22

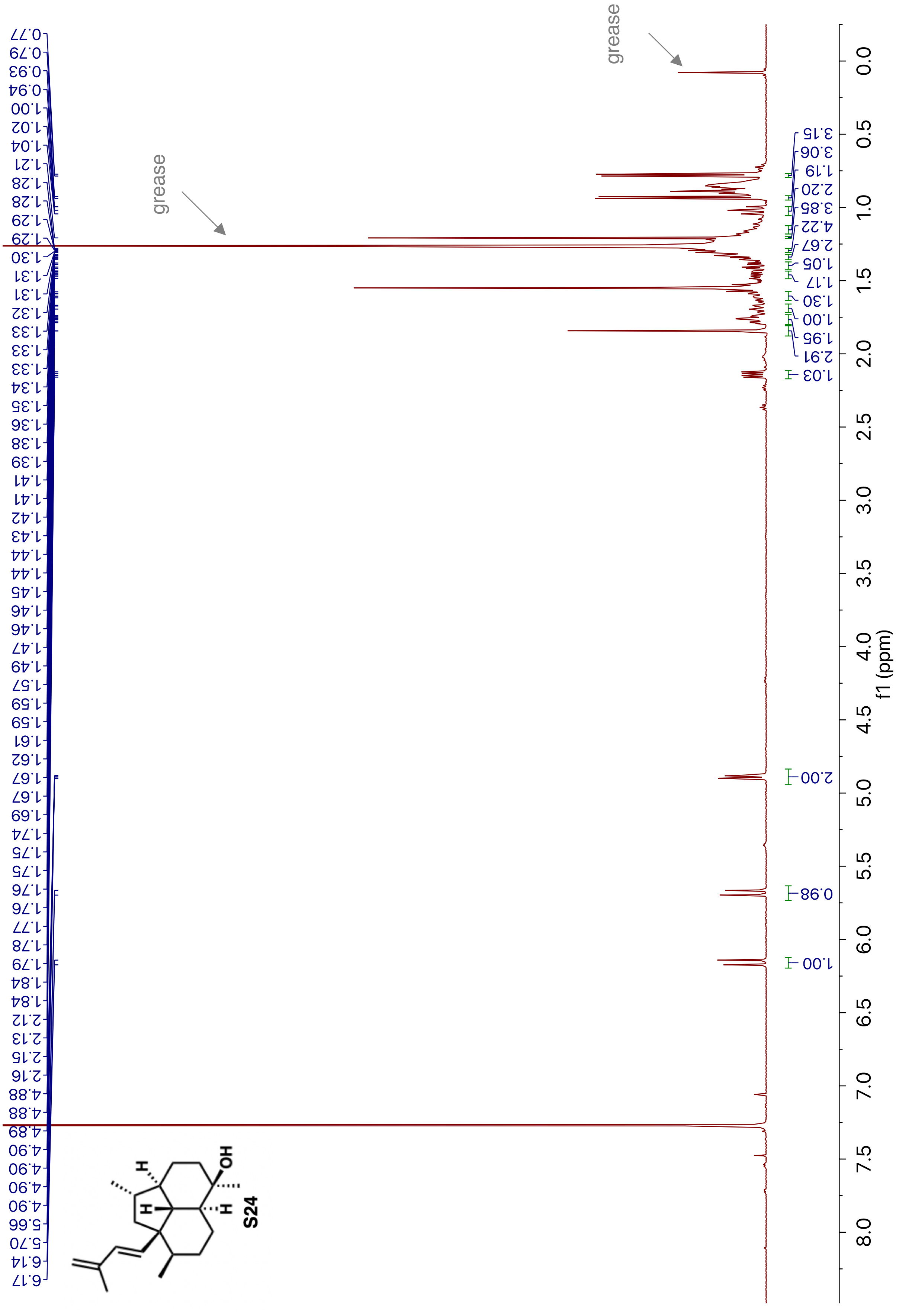
0.77
0.79
0.93
0.94
1.00
1.02
1.04
1.21
1.28
1.28
1.29
1.29
1.30
1.31
1.31
1.32
1.33
1.33
1.33
1.34
1.35
1.36
1.38
1.39
1.41
1.41
1.42
1.43
1.44
1.44
1.45
1.46
1.46
1.47
1.49
1.57
1.59
1.59
1.61
1.62
1.67
1.67
1.69
1.74
1.75
1.75
1.76
1.76
1.77
1.78
1.79
1.84
1.84
2.12
2.13
2.15
2.16
4.88
4.88
4.89
4.90
4.90
4.90
4.90
5.66
5.70
6.14
6.17
grease
grease
H
H
H
OH
S24
3.15
3.06
1.19
2.20
3.85
4.22
2.67
1.05
1.17
1.30
1.00
1.95
2.91
1.03
2.00
0.98
1.00
f1 (ppm)

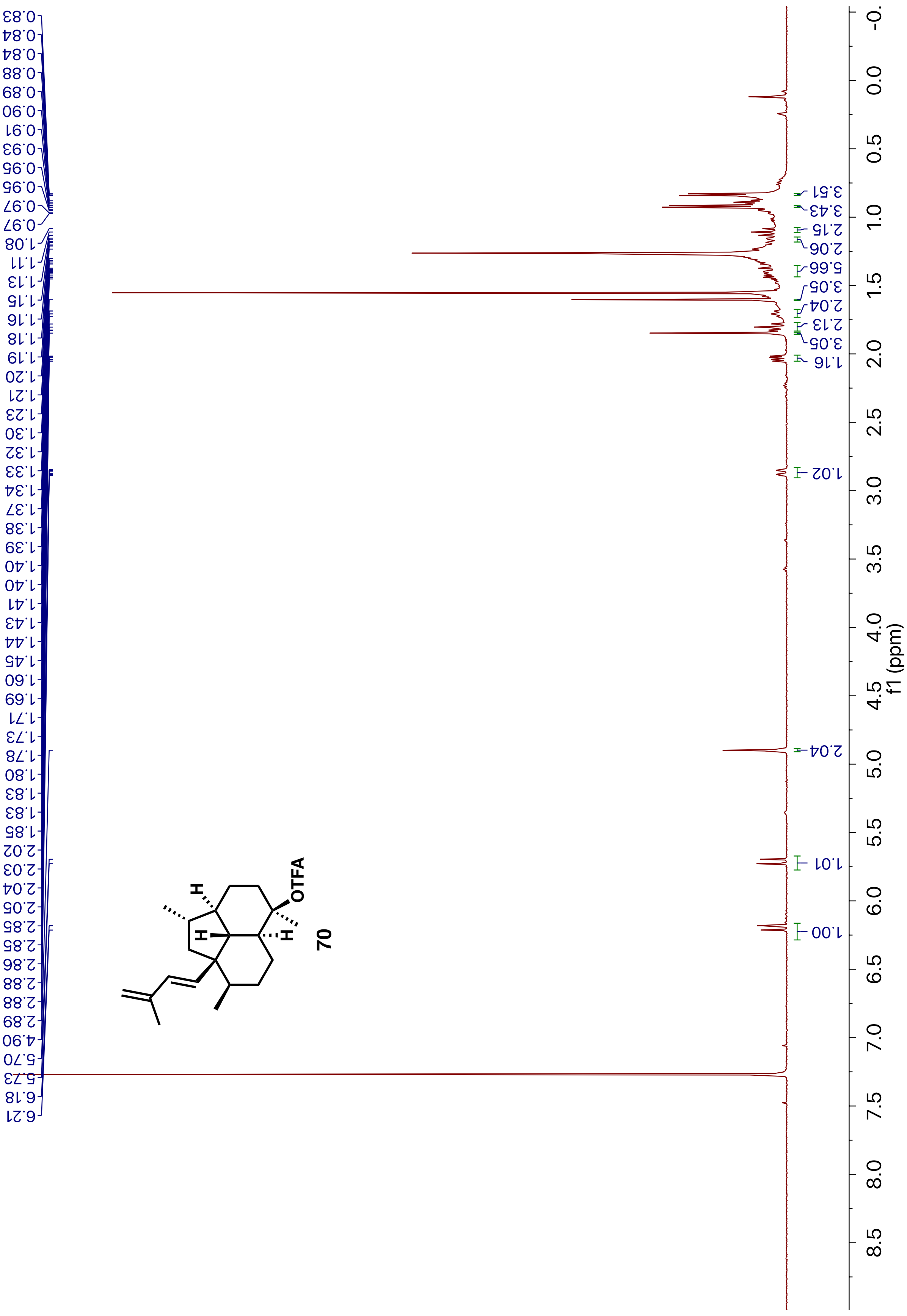
0.83
0.84
0.84
0.88
0.89
0.90
0.91
0.93
0.95
0.95
0.97
0.97
1.08
1.11
1.13
1.15
1.16
1.18
1.19
1.20
1.21
1.23
1.30
1.32
1.33
1.34
1.37
1.38
1.39
1.40
1.40
1.41
1.43
1.44
1.45
1.60
1.69
1.71
1.73
1.78
1.80
1.83
1.83
1.85
2.02
2.03
2.04
2.05
2.85
2.85
2.86
2.88
2.88
2.89
4.90
5.70
5.73
6.18
6.21
3.51
3.43
2.15
2.06
5.66
3.05
2.04
2.13
3.05
1.16
1.02
2.04
1.01
1.00
OTFA
H
H
H
70
f1 (ppm)

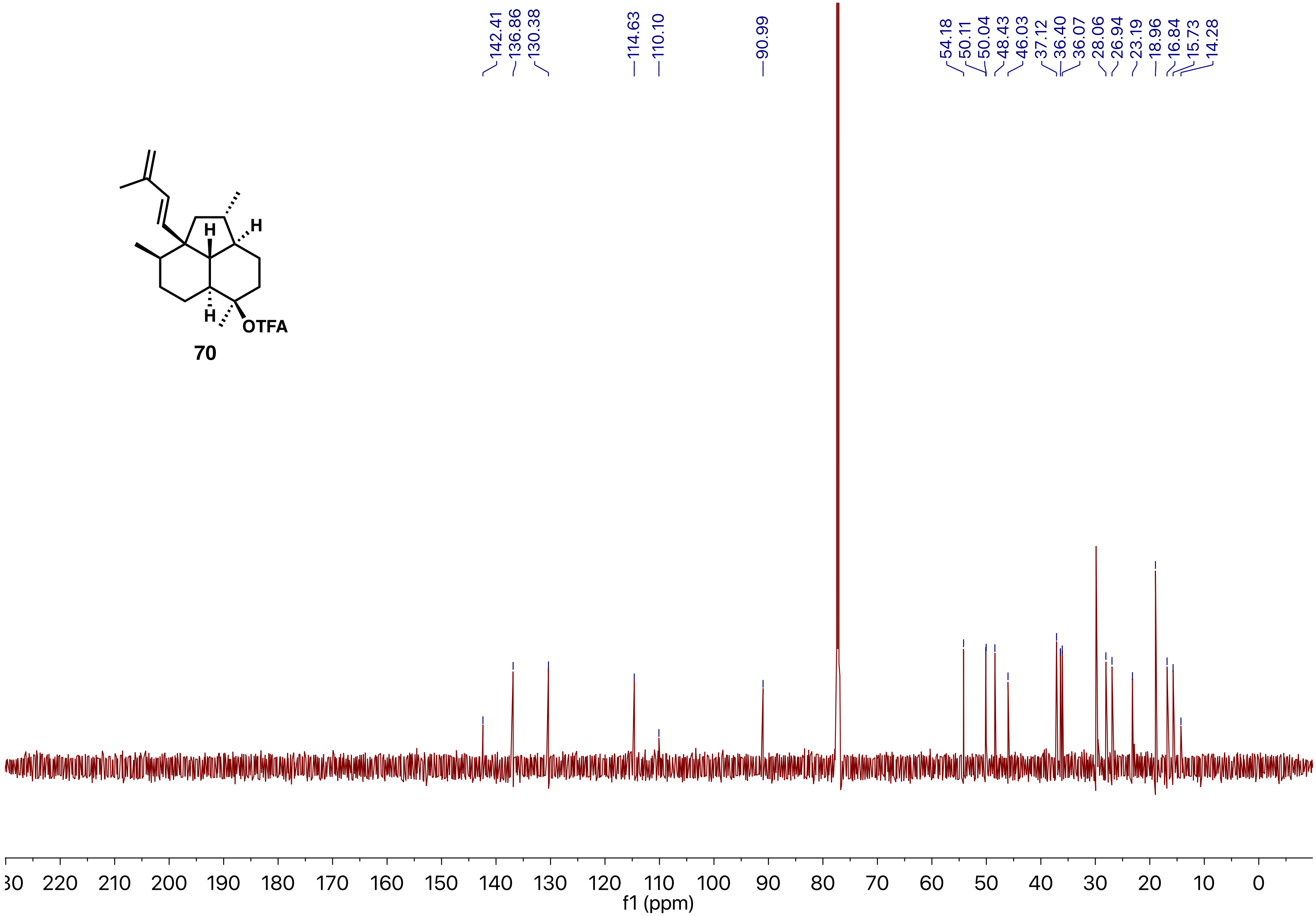
H
H
H
OTFA
70
142.41
136.86
130.38
114.63
110.10
90.99
54.18
50.11
50.04
48.43
46.03
37.12
36.40
36.07
28.06
26.94
23.19
18.96
16.84
15.73
14.28
f1 (ppm)

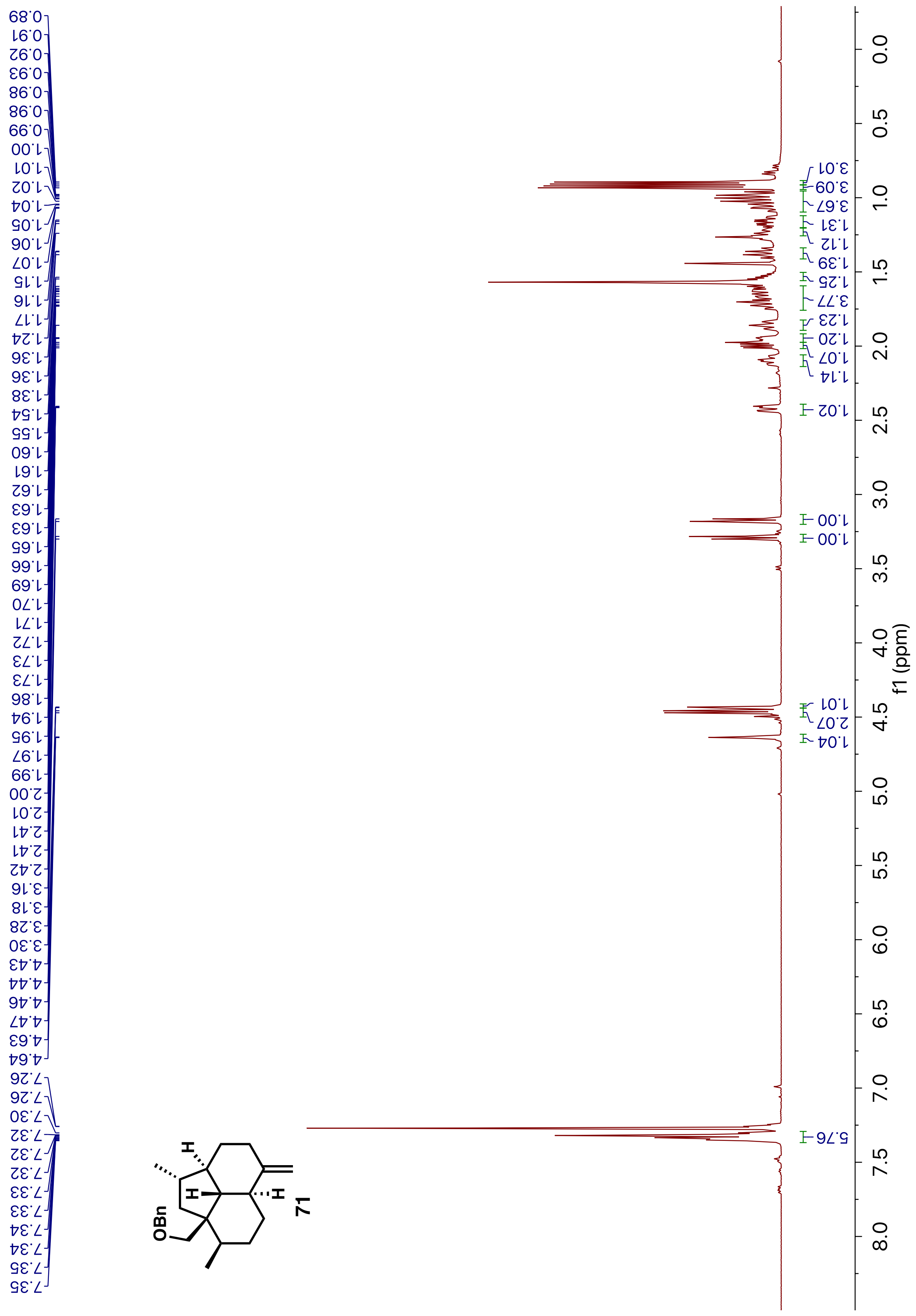
0.89 0.91 0.92 0.93 0.98 0.98 0.99 1.00 1.01 1.02 1.04 1.05 1.06 1.07 1.15 1.16 1.17 1.24 1.36 1.36 1.38 1.54 1.55 1.60 1.61 1.62 1.63 1.63 1.65 1.66 1.69 1.70 1.71 1.72 1.73 1.73 1.86 1.94 1.95 1.97 1.99 2.00 2.01 2.41 2.41 2.42 3.16 3.18 3.28 3.30 4.43 4.44 4.46 4.47 4.63 4.64 7.26 7.26 7.30 7.32 7.32 7.32 7.33 7.33 7.34 7.34 7.35 7.35
OBn
H
H
H
71
3.01 3.09 3.67 1.31 1.12 1.39 1.25 3.77 1.23 1.20 1.07 1.14 1.02 1.00 1.00 1.01 2.07 1.04 5.76
f1 (ppm)

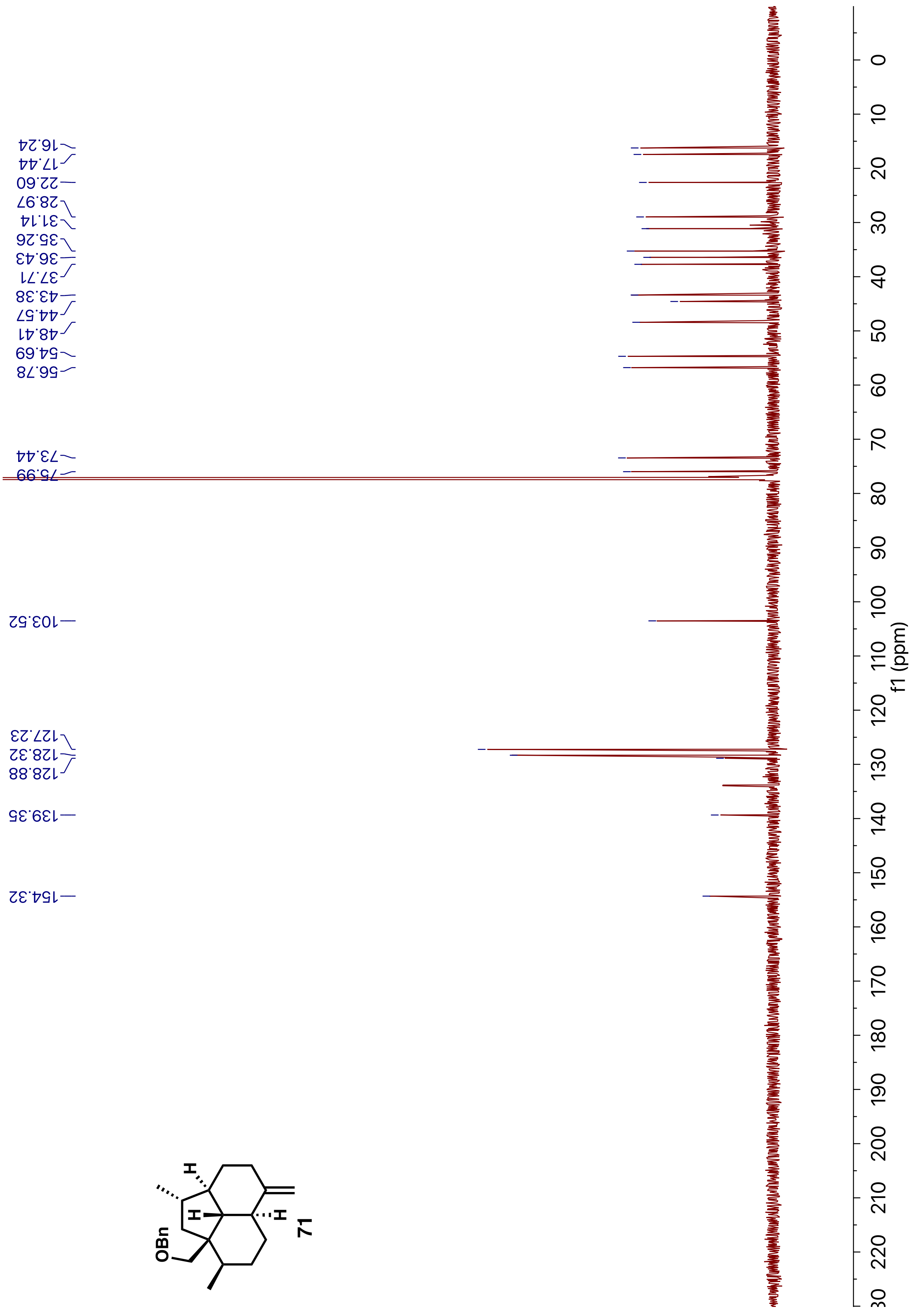
16.24
17.44
22.60
28.97
31.14
35.26
36.43
37.71
43.38
44.57
48.41
54.69
56.78
73.44
75.99
103.52
127.23
128.32
128.88
139.35
154.32
f1 (ppm)
OBn
71

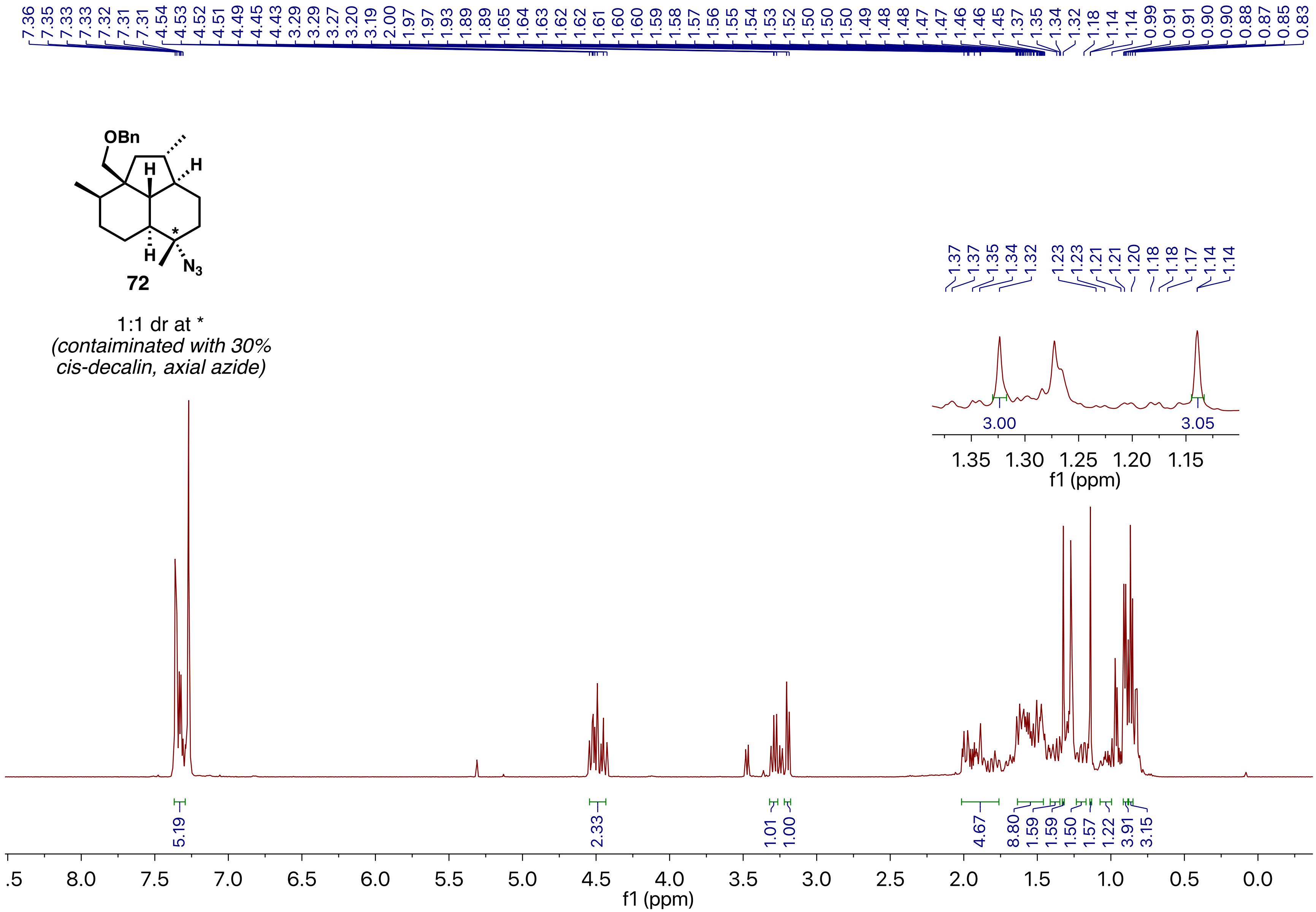

72

1:1 dr at *
(contaiminated with 30% cis-decalin, axial azide)

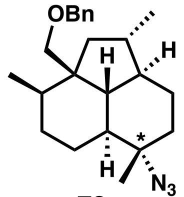

72

1:1 dr at *
*(contaiminated with 30%
cis-decalin, axial azide)*

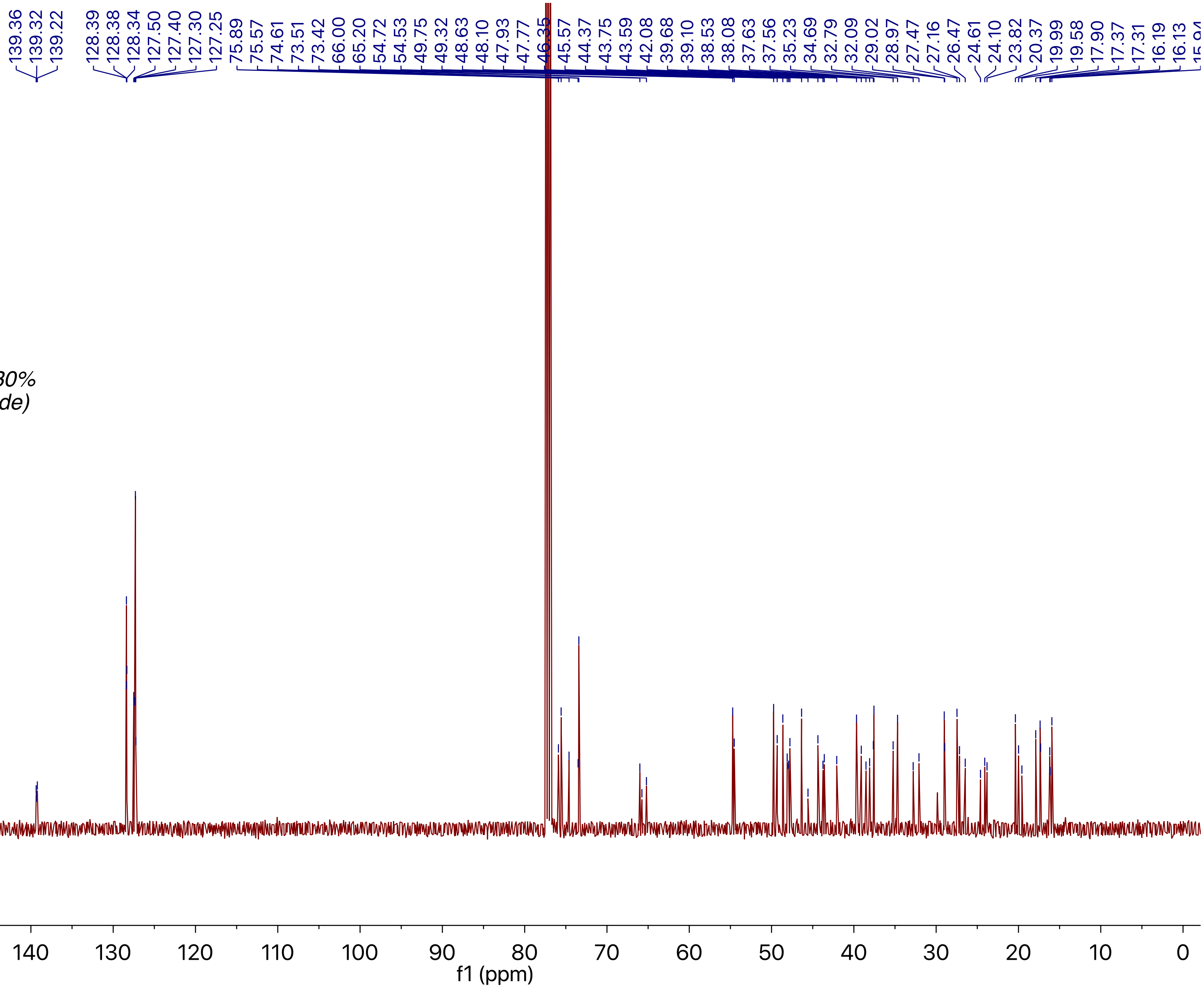

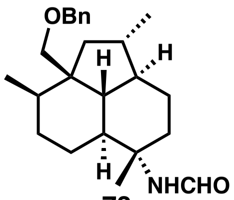

73

(1.7:1 mixture of cis/trans formamide rotamers)

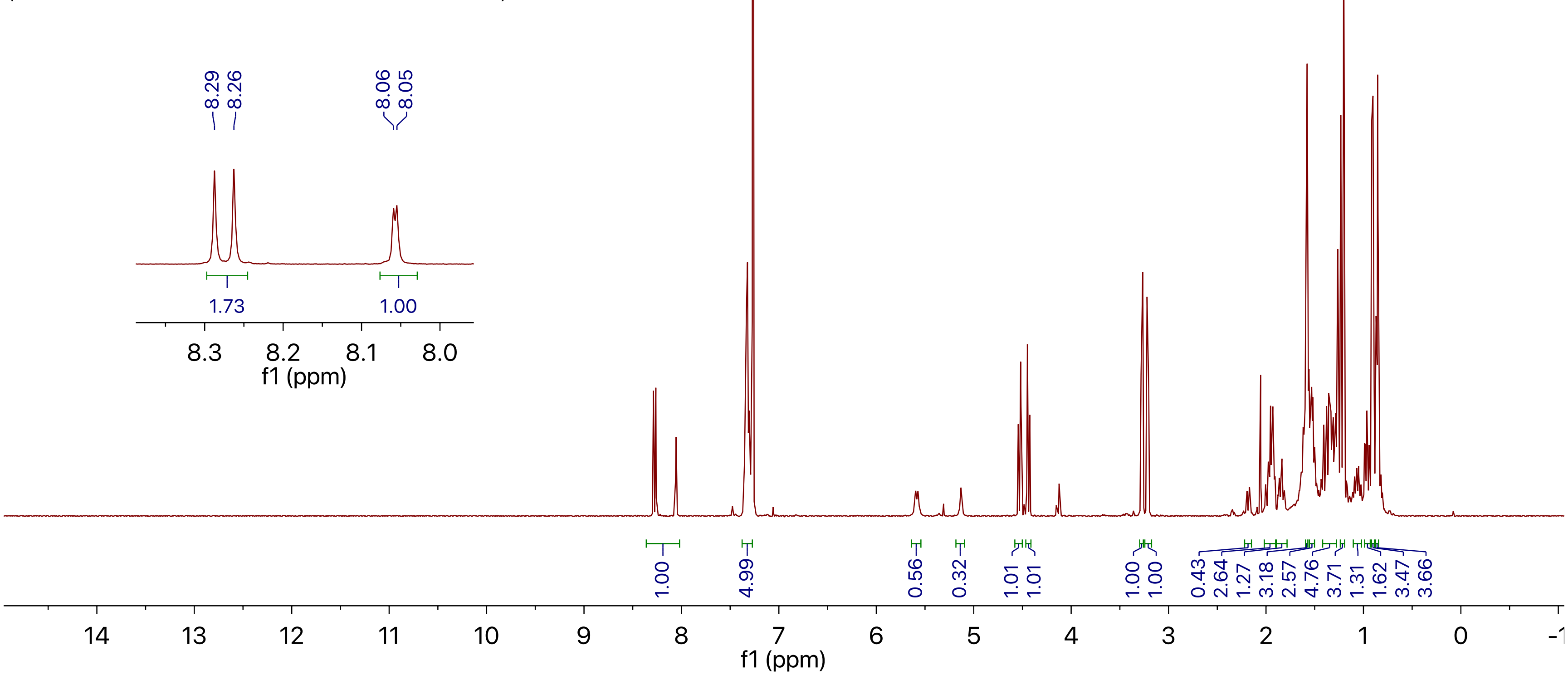

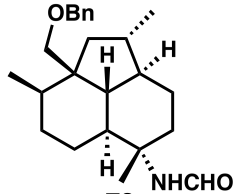

73

(1.7:1 mixture of cis/trans formamide rotamers)

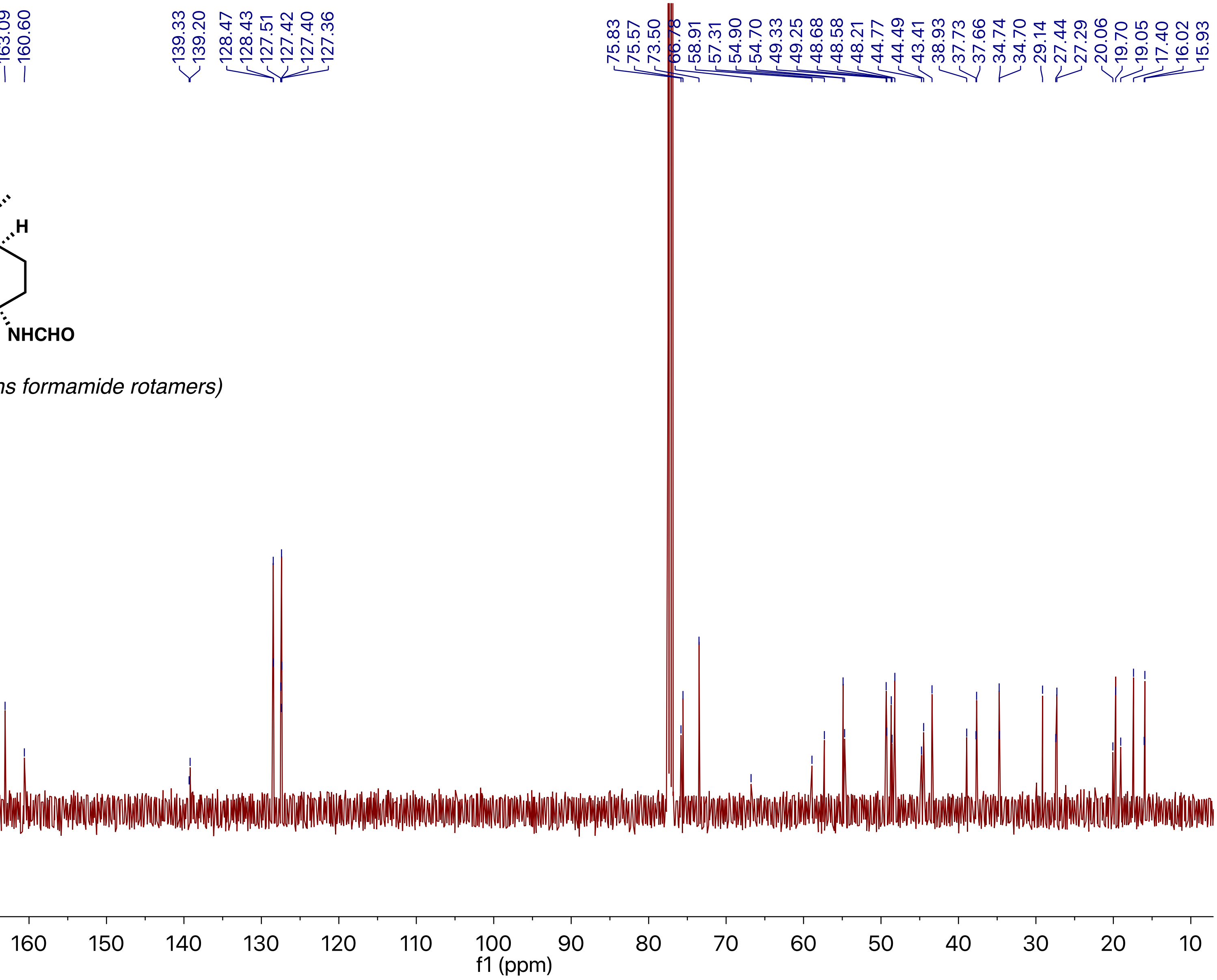

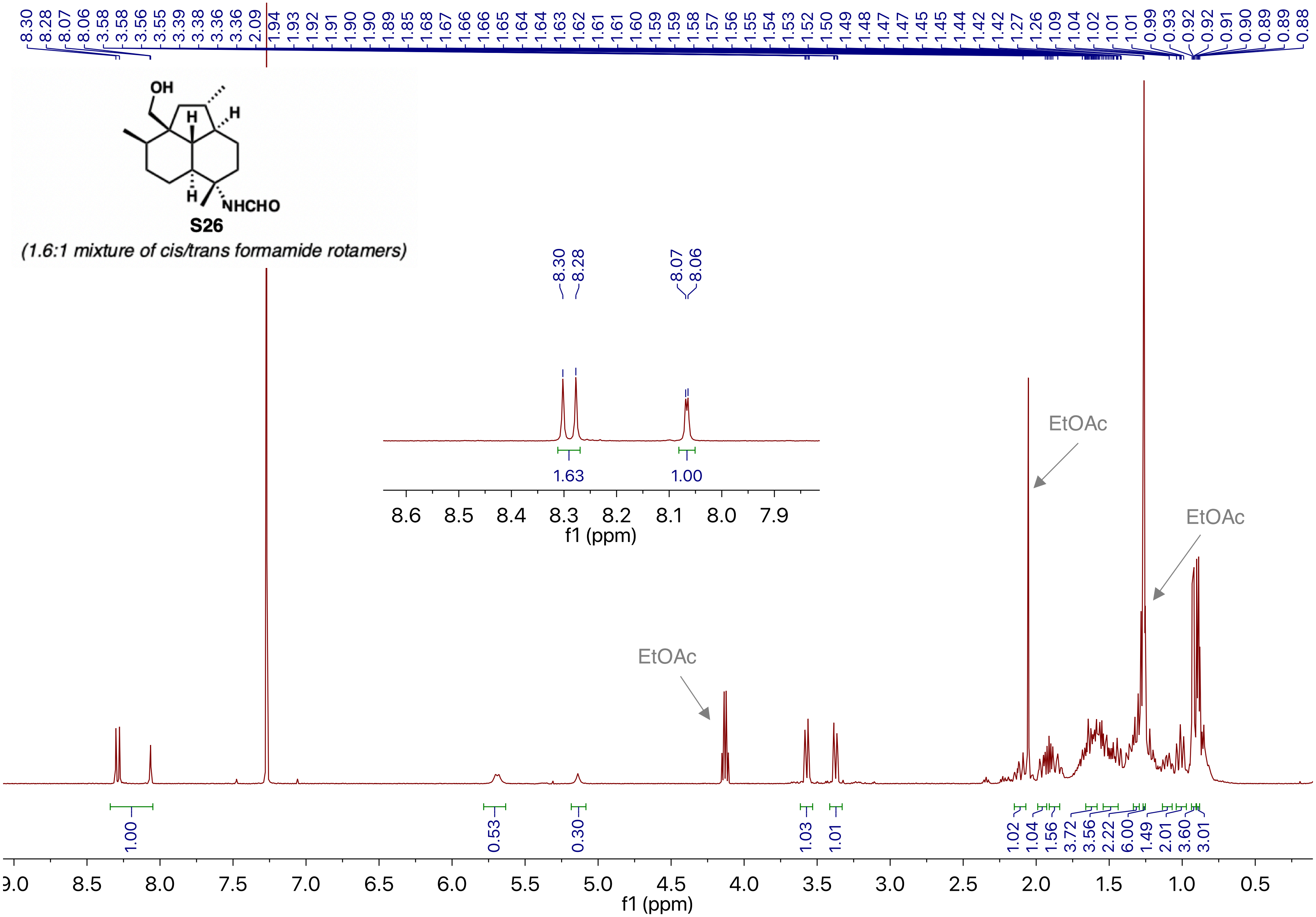

S26

(1.6:1 mixture of cis/trans formamide rotamers)

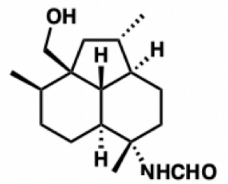

S26

(1.6:1 mixture of cis/trans formamide rotamers)

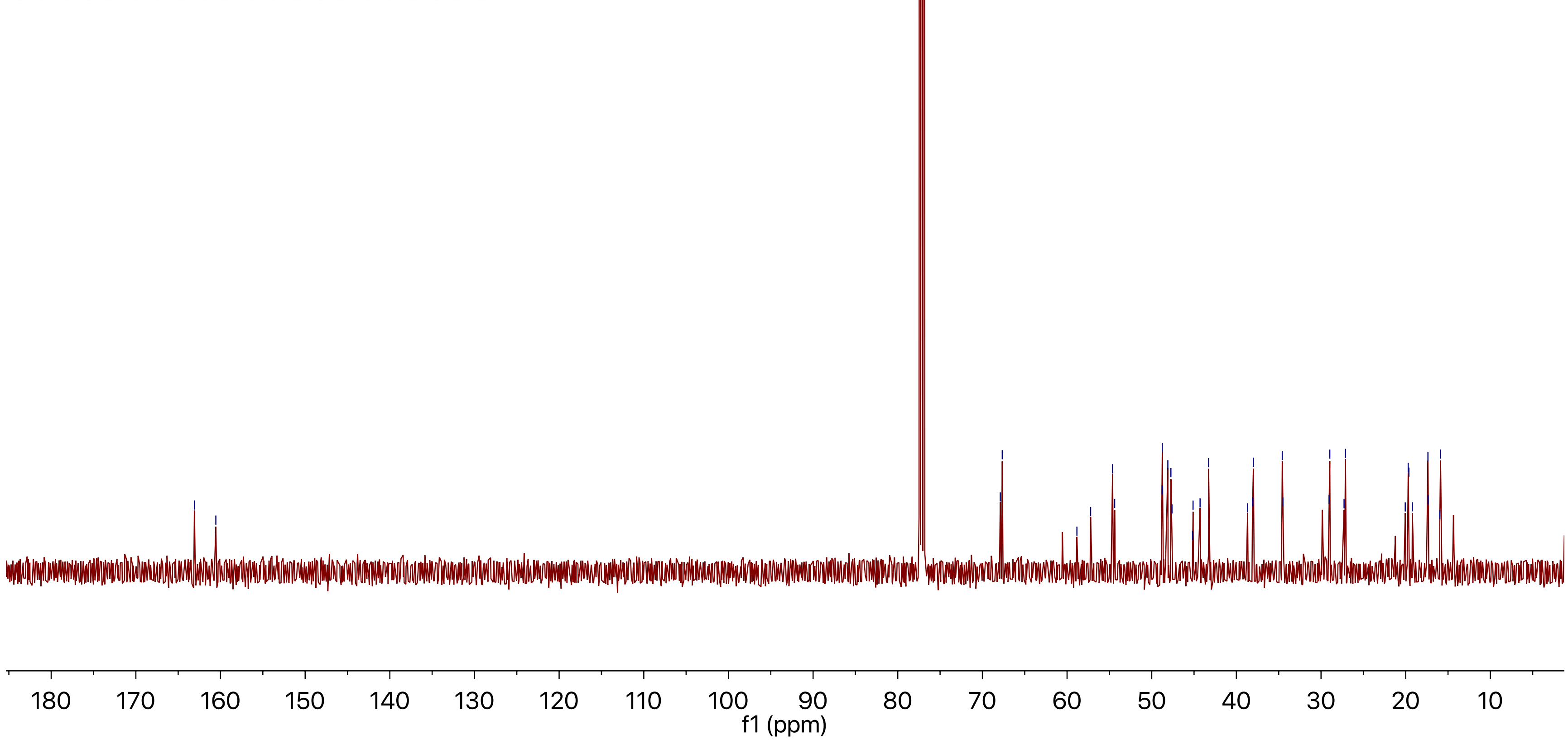

74

(1.2:1 mixture of cis/trans formamide rotamers)

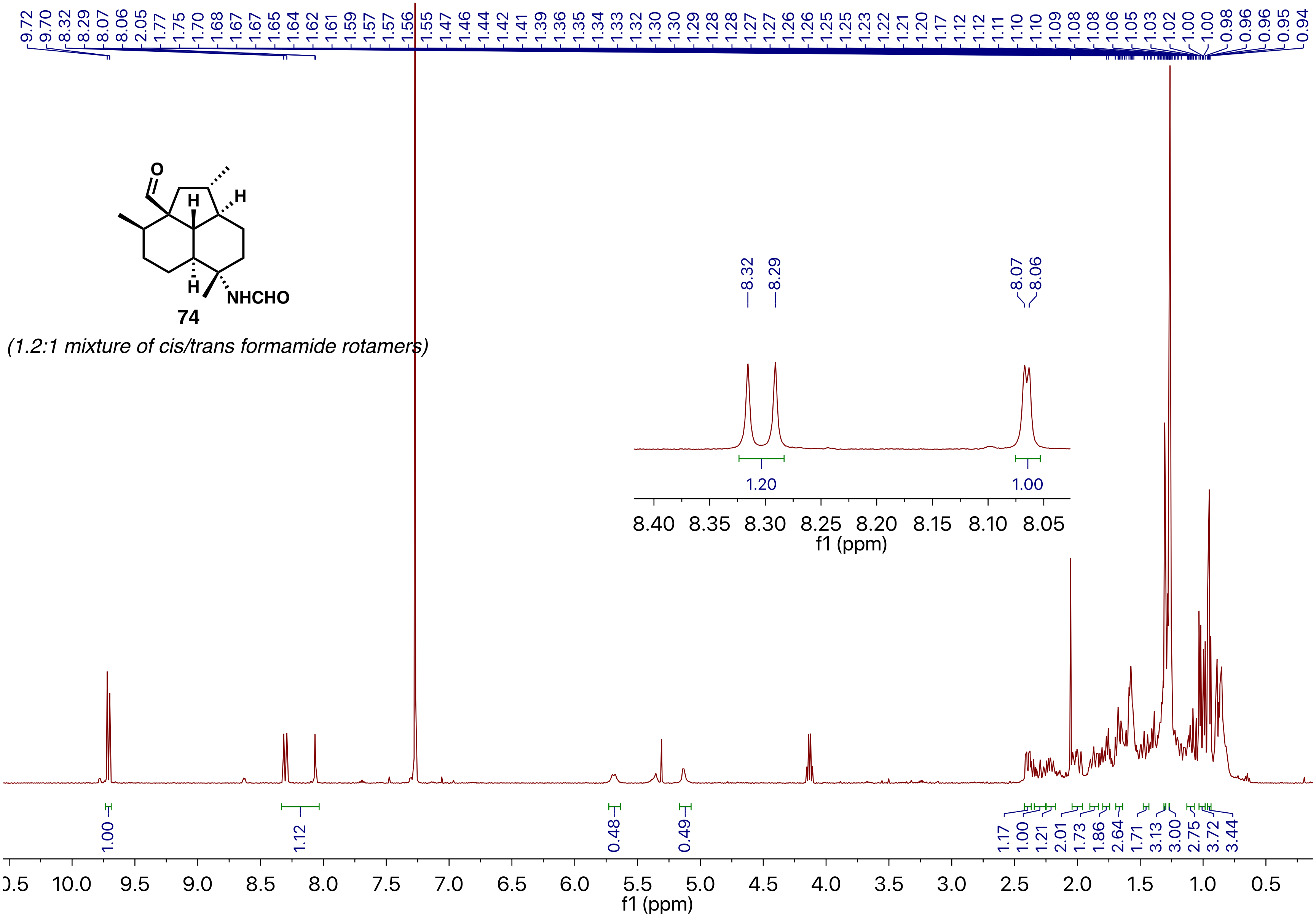

Literature ¹H NMR of 7-formamidoisoneoamphilectane: 2:1 mixture of rotamers

Reprinted with permission from Ref. 7. Copyright 2013
American Chemical Society

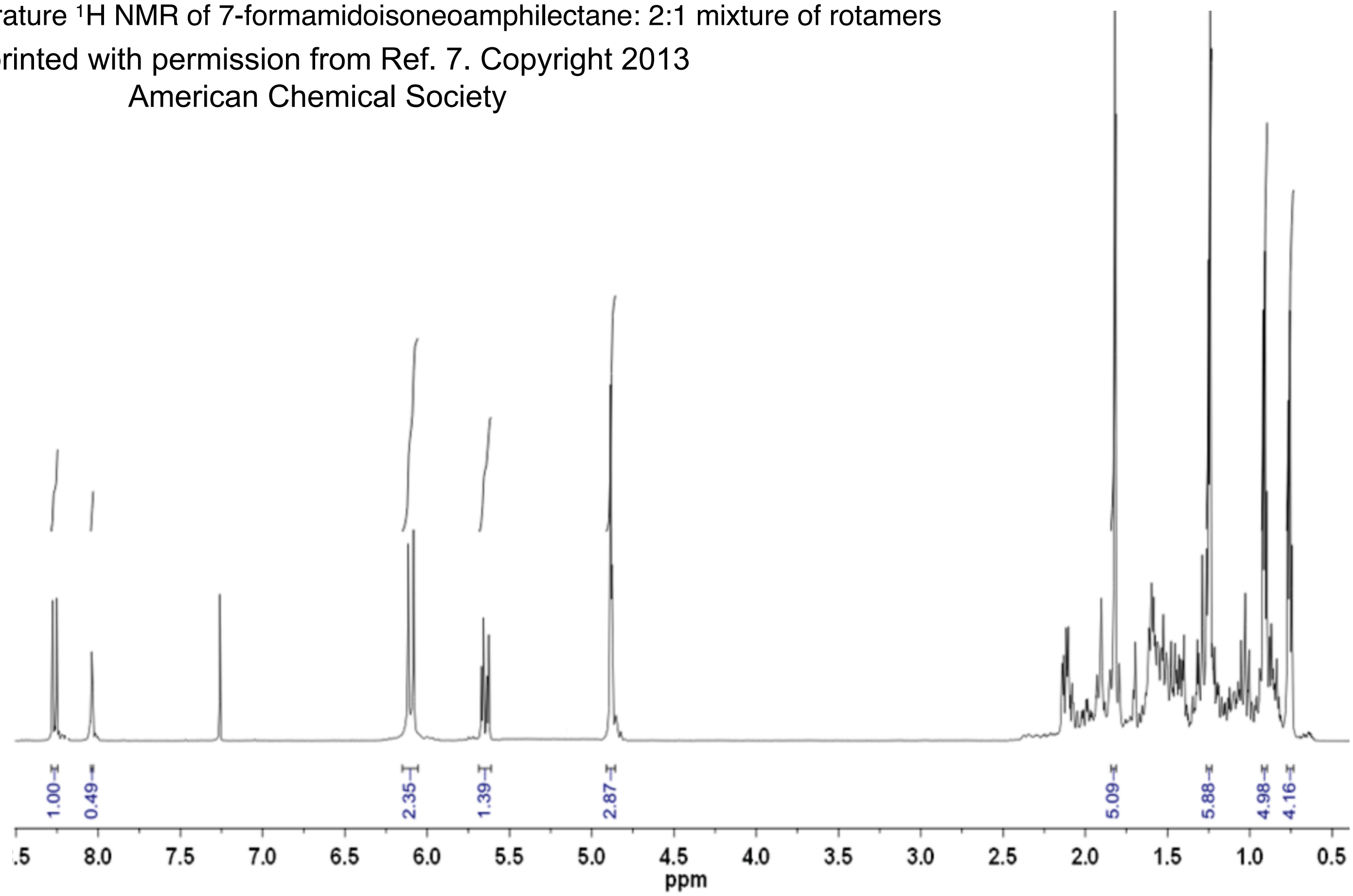

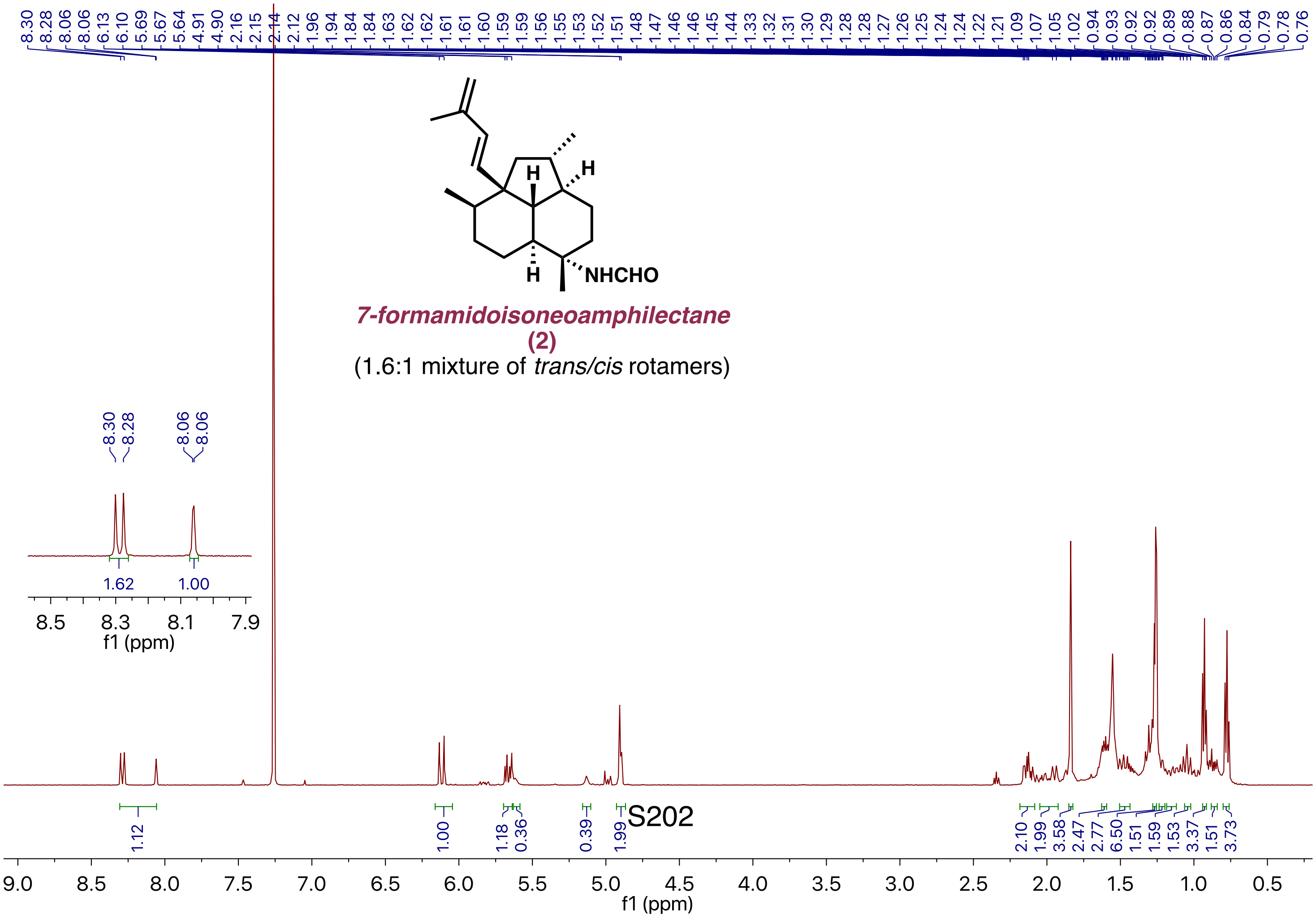

7-formamidoisoneoamphilectane
(2)
(1.6:1 mixture of *trans/cis* rotamers)

Literature ^{13}C NMR of 7-formamidoisoneoamphilectane:
2:1 mixture of rotamers
Reprinted with permission from Ref. 7. Copyright 2013 American Chemical Society

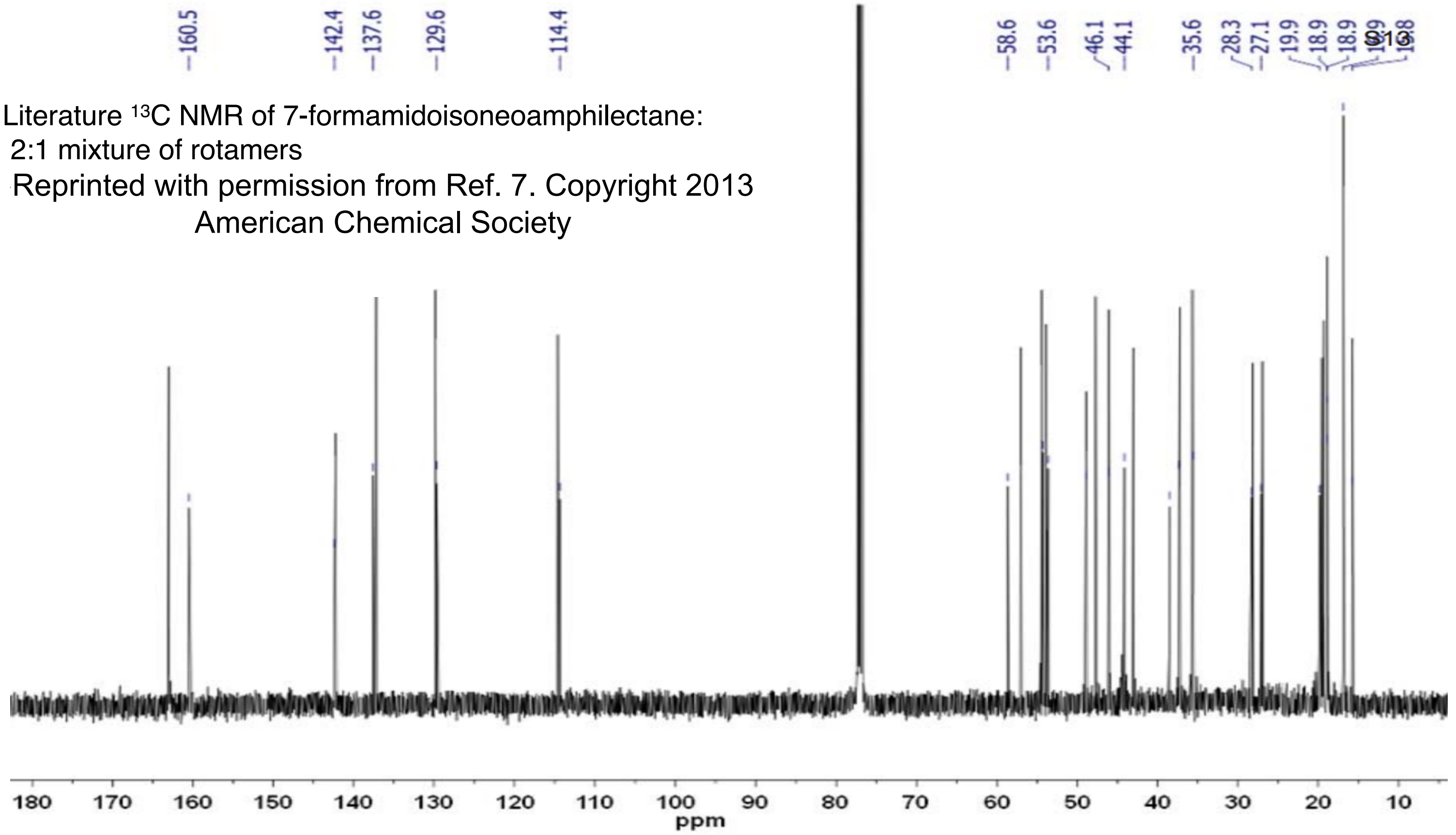

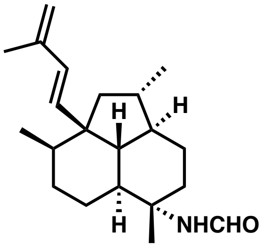

7-formamidoisoneoamphilectane
(2)
(1.6:1 mixture of *trans/cis* rotamers)

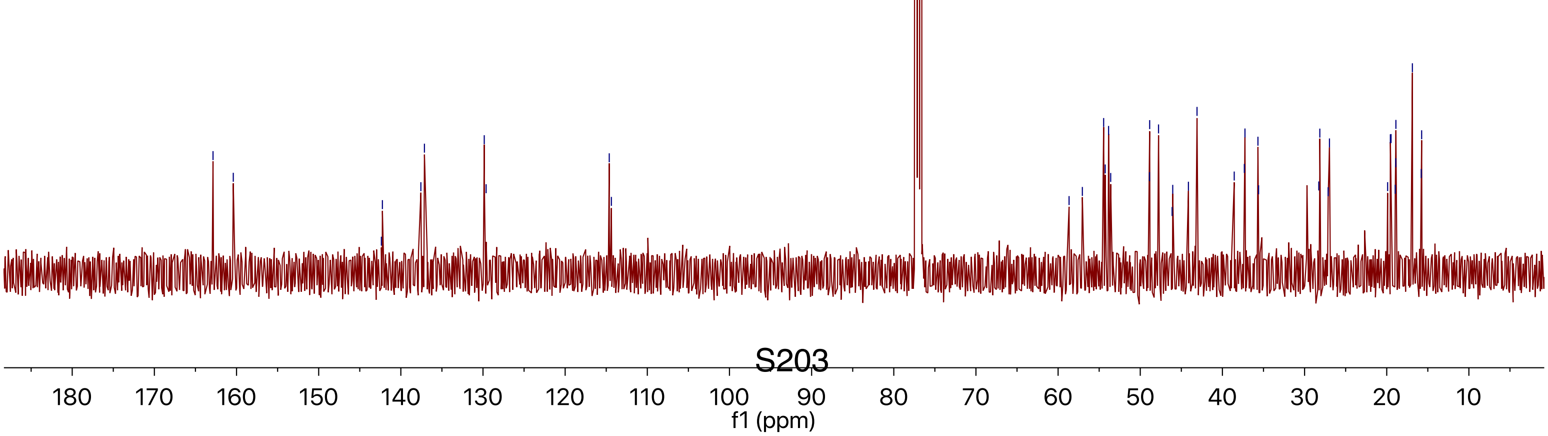

Literature ^{1}H NMR of isoneoamphilectane:

Reprinted with permission from Ref. 8. Copyright 1996
American Chemical Society

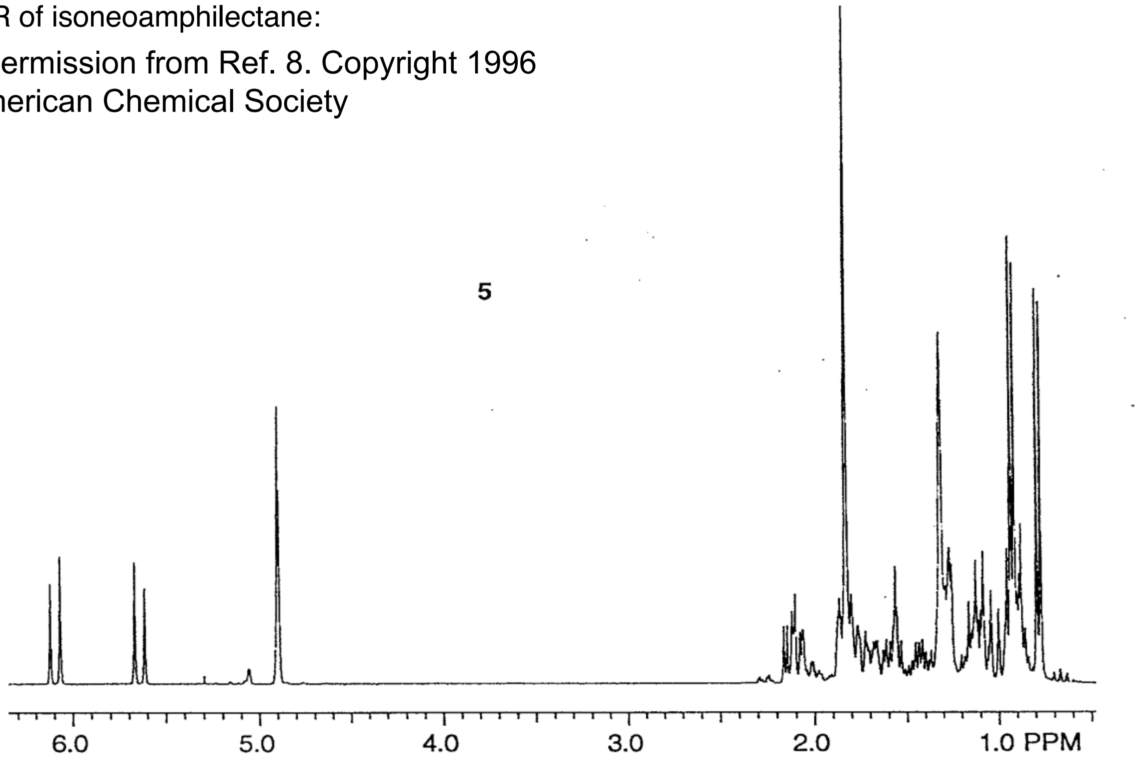

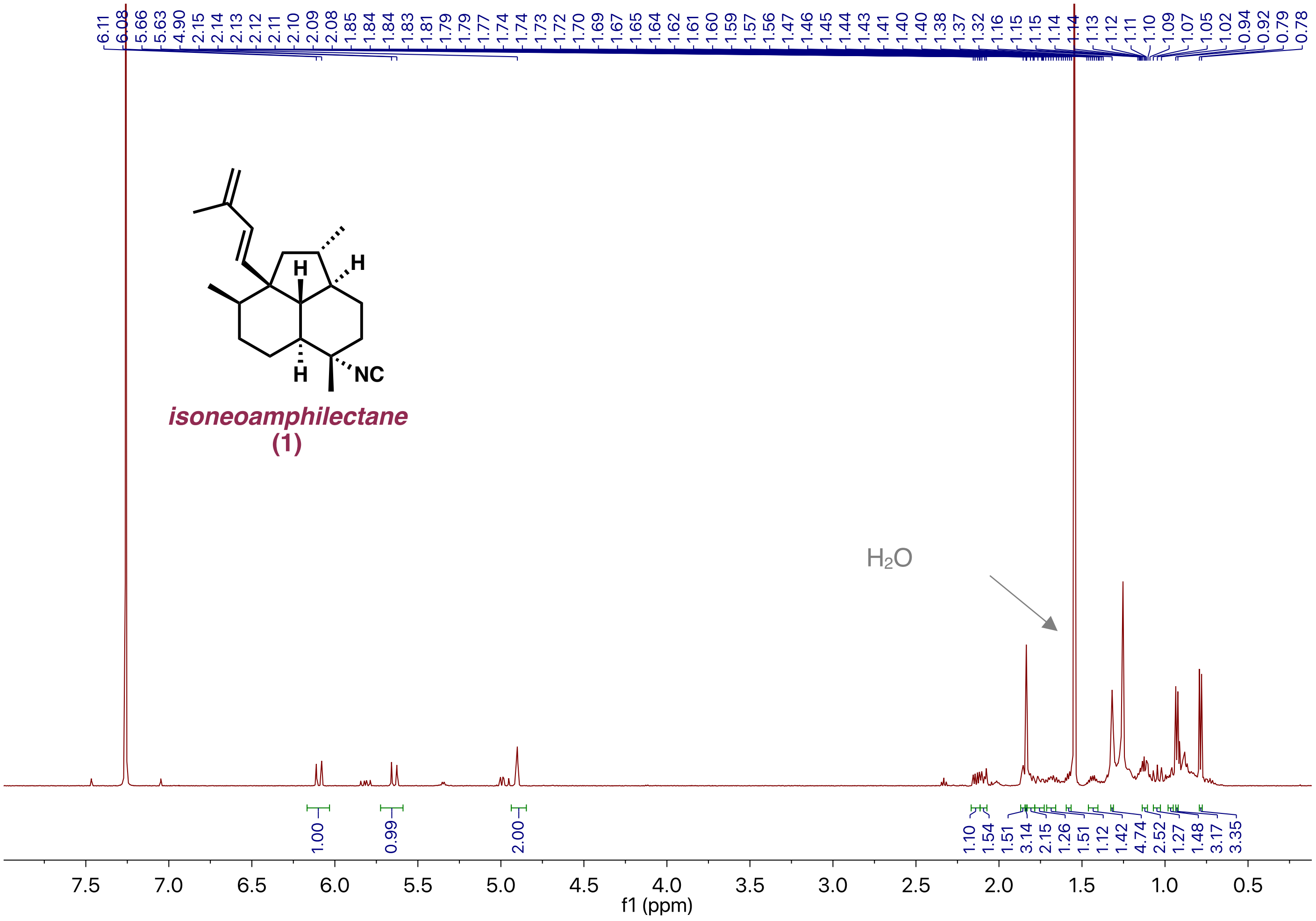

Literature ^{13}C NMR of isoneoamphilectane:

Reprinted with permission from Ref. 8. Copyright 1996 American Chemical Society

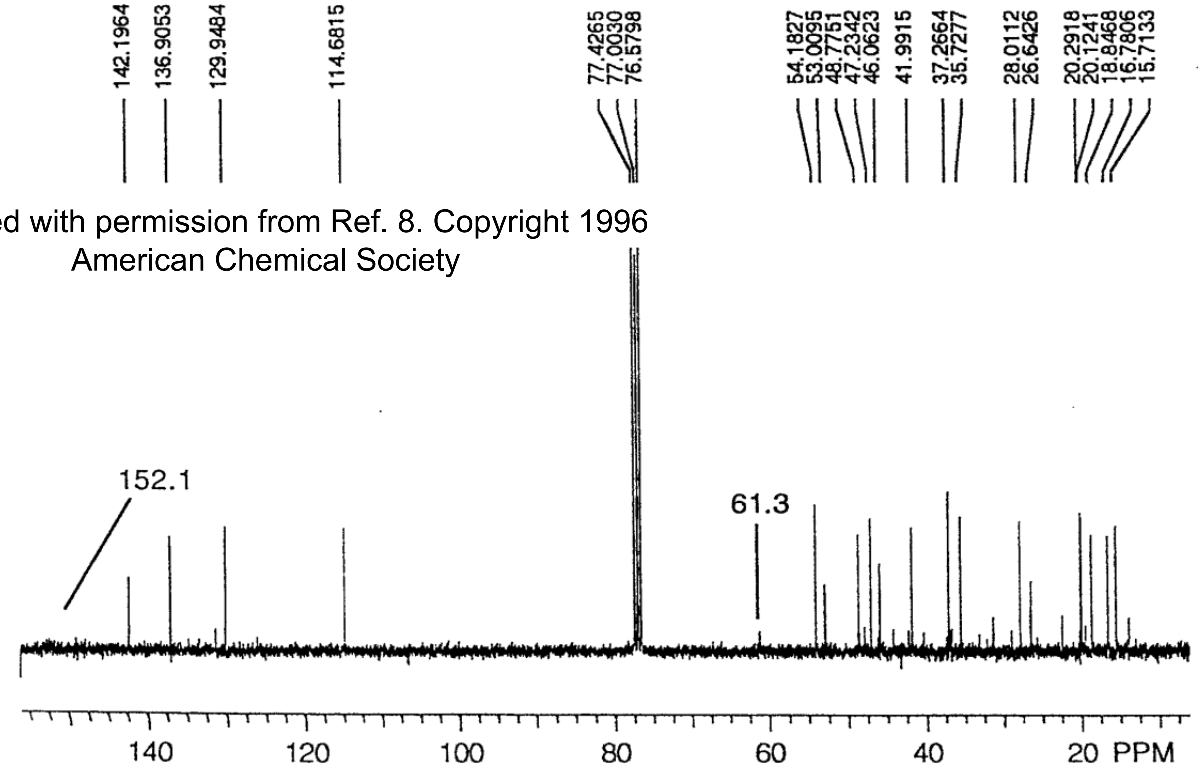

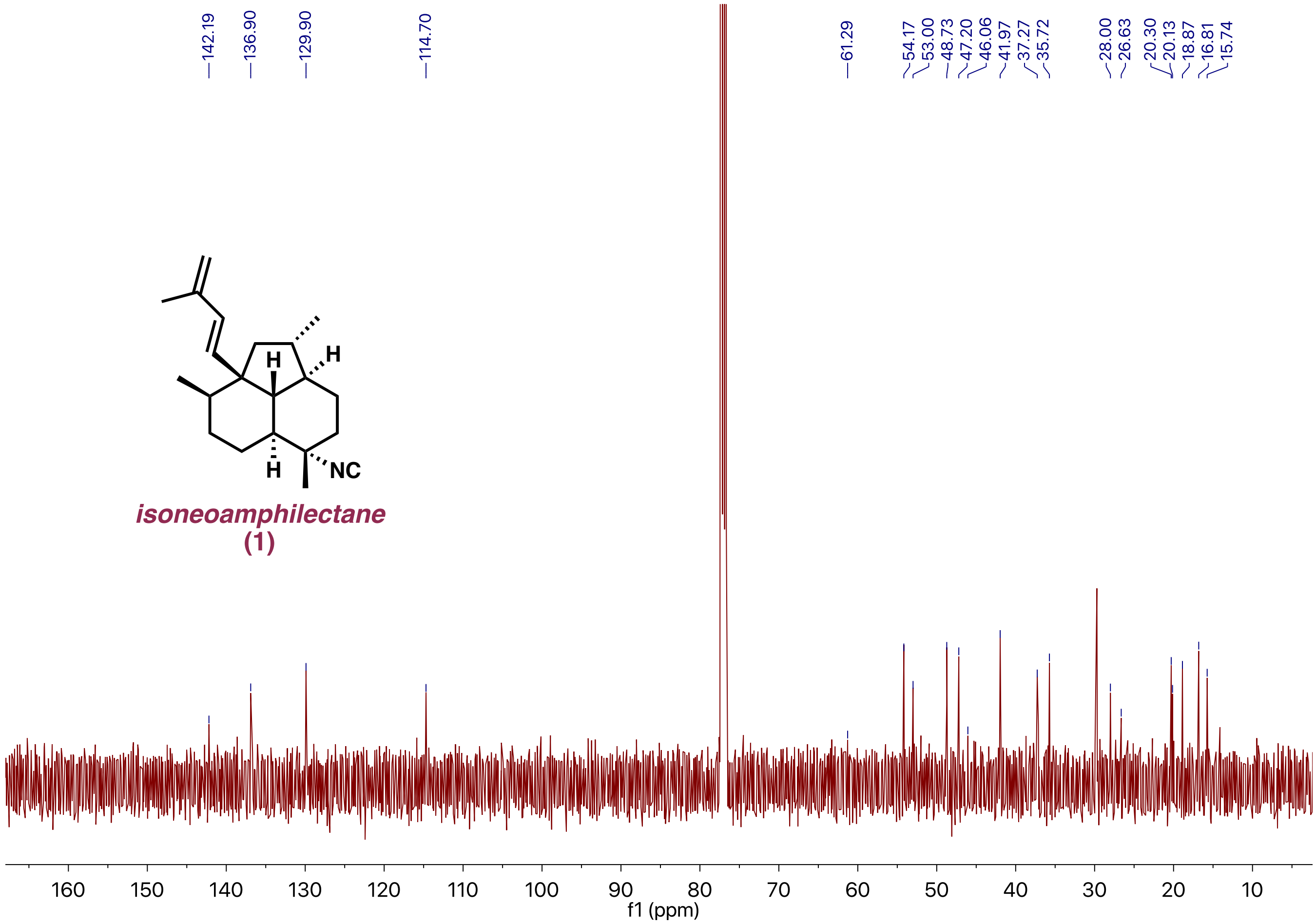

isoneoamphilectane
(1)

F. Biological Data

NCD4180

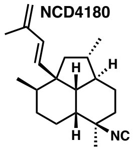

43

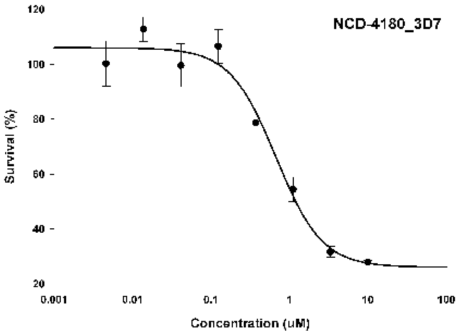

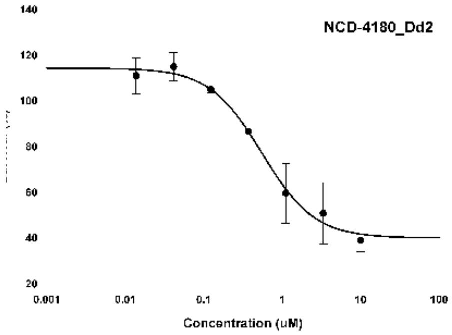

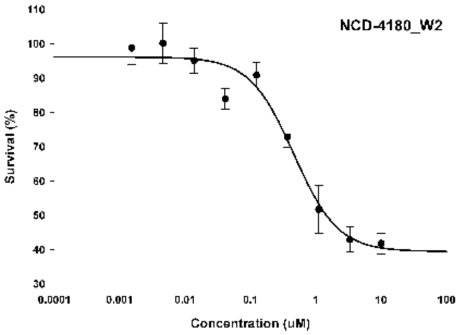

NCD6120

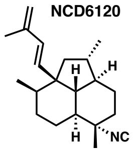

1

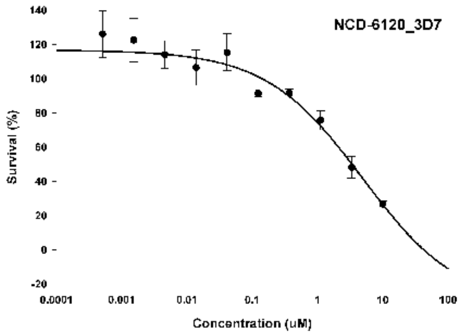

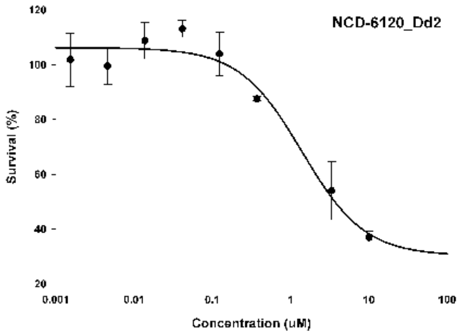

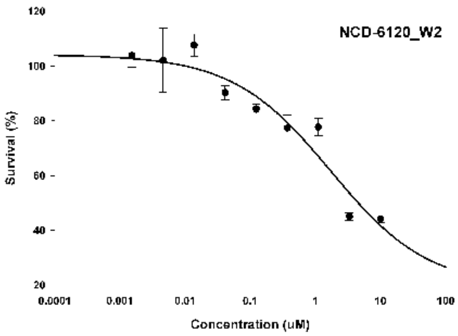

NCD6129

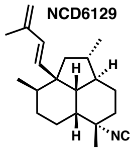

12

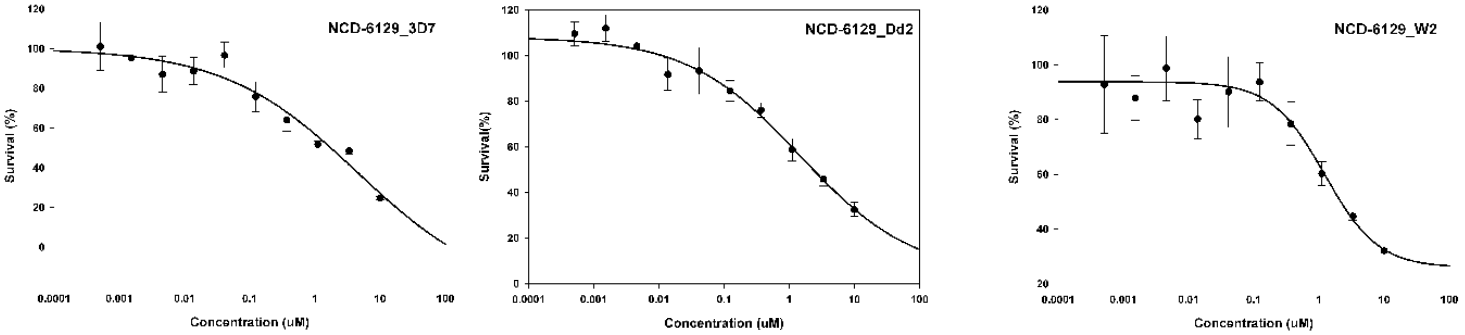

	EC50 (uM)					
**Compound**	**3D7**	**Stdev**	**Dd2**	**Stdev**	**W2**	**Stdev**
**NCD-4180**	0.6752	0.174	0.5505	0.1044	0.4619	0.146
**NCD-6120**	4.8729	1.2	1.3965	0.6968	1.718	0.322
**NCD-6129**	4.515	1.6	1.473	0.185	1.2283	0.7363

	Sample Name	Molecular Weight (g/mol)	Amount (mg)	volume DMSO (uL)	Stock Concentrations
**NCD compounds**	6120	297.86	0.30000	100.72	10mM stock
	6129	297.86	1.00000	335.73	10mM stock
	4180	297.86	1.10000	369.30	10mM stock

G. References

1. Purchased from Sigma Aldrich or prepared by pig liver esterase asymmetric ester hydrolysis of dimethyl 3-methylglutarate using the procedure from: Lehr, K.; Mariz, R.; Leseurre, L.; Gabor, B.; Fürstner, A. Total Synthesis of Tulearin C. *Angewandte Chemie International Edition* **2011**, *50*, 11373–11377.

2. Anžiček, N.; Williams, S.; Housden, M. P.; Paterson, I. Toward Aplyronine Payloads for Antibody–Drug Conjugates: Total Synthesis of Aplyronines A and D. *Org. Biomol. Chem.* **2018**, *16*, 1343–1350.

3. Hicken, E. J.; Corey, E. J. Stereoselective Synthesis of Woody Fragrances Related to Georgyone and Arborone. *Org. Lett.* **2008**, *10*, 1135–1138.

4. Roosen, P. C.; Vanderwal, C. D. A Formal Enantiospecific Synthesis of 7,20-Diisocyanoadociane. *Angew. Chem. Int. Ed.* **2016**, *55*, 7180–7183.

5. Ackermann, J.; Waespe-Šarčevič, N.; Tamm, C. Approaches to the Synthesis of Cytochalasans. Part 6. Synthesis of the C(14)–C(23) Subunit of Cytochalasins A, B. F and Desoxaphomin. *Helvetica Chimica Acta* **1984**, *67*, 254–260.

6. Nagase, R.; Osada, J.; Tamagaki, H.; Tanabe, Y. Pentafluorophenylammonium Trifluoromethanesulfonimide: Mild, Powerful, and Robust Catalyst for Mukaiyama Aldol and Mannich Reactions between Ketene Silyl Acetals and Ketones or Oxime Ethers. *Adv. Synth. Catal.* **2010**, *352*, 1128 –1134.

7. Avilés, E.; Rodríguez, A. D.; Vicente, J. Two Rare-Class Tricyclic Diterpenes with Antitubercular Activity from the Caribbean Sponge *Svenzea Flava* Application of Vibrational Circular Dichroism Spectroscopy for Determining Absolute Configuration. *J. Org. Chem.* **2013**, *78*, 11294–11301.

8. König, G. M.; Wright, A. D.; Angerhofer, C. K. Novel Potent Antimalarial Diterpene Isocyanates, Isothiocyanates, and Isonitriles from the Tropical Marine Sponge *Cymbastela Hooperi*. *J. Org. Chem.* **1996**, *61*, 3259–3267.

9. Spartan 18, Wavefunction Inc., Irvine CA: Shao, Y.; Gan, Z.; Epifanovsky, E.; Gilbert, A. T. B.; Wormit, M.; Kussmann, J.; Lange, A. W.; Behn, A.; Deng, J.; Feng, X.; Ghosh, D.; Goldey, M.; Horn, P. R.; Jacobson, L. D.; Kaliman, I.; Khaliullin, R. Z.; Kuś, T.; Landau, A.; Liu, J.; Proynov, E. I.; Rhee, Y. M.; Richard, R. M.; Rohrdanz, M. A.; Steele, R. P.; Sundstrom, E. J.; Woodcock, H. L., III; Zimmerman, P. M.; Zuev, D.; Albrecht, B.; Alguire, E.; Austin, B.; Beran, G. J. O.; Bernard, Y. A.; Berquist, E.; Brandhorst, K.; Bravaya, K. B.; Brown, S. T.; Casanova, D.; Chang, C.-M.; Chen, Y.; Chien, S. H.; Closser, K. D.; Crittenden, D. L.; Diedenhofen, M.; DiStasio, R. A., Jr.; Do, H.; Dutoi, A. D.; Edgar, R. G.; Fatehi, S.; Fusti-Molnar, L.; Ghysels, A.; Golubeva-Zadorozhnaya, A.; Gomes, J.; Hanson-Heine, M. W. D.; Harbach, P. H. P.; Hauser, A. W.; Hohenstein, E. G.; Holden, Z. C.; Jagau, T.-C.; Ji, H.; Kaduk, B.; Khistyaev, K.; Kim, J.; Kim, J.; King, R. A.; Klunzinger, P.; Kosenkov, D.; Kowalczyk, T.; Krauter, C. M.; Lao, K. U.; Laurent, A. D.; Lawler, K. V.; Levchenko, S. V.; Lin, C. Y.; Liu, F.; Livshits, E.; Lochan, R. C.; Luenser, A.; Manohar, P.; Manzer, S. F.; Mao, S.-P.; Mardirossian, N.; Marenich, A. V.;

Maurer, S. A.; Mayhall, N. J.; Neuscamman, E.; Oana, C. M.; Olivares-Amaya, R.; O'Neill, D. P.; Parkhill, J. A.; Perrine, T. M.; Peverati, R.; Prociuk, A.; Rehn, D. R.; Rosta, E.; Russ, N. J.; Sharada, S. M.; Sharma, S.; Small, D. W.; Sodt, A.; Stein, T.; Stück, D.; Su, Y.-C.; Thom, A. J. W.; Tsuchimochi, T.; Vanovschi, V.; Vogt, L.; Vydrov, O.; Wang, T.; Watson, M. A.; Wenzel, J.; White, A.; Williams, C. F.; Yang, J.; Yeganeh, S.; Yost, S. R.; You, Z.-Q.; Zhang, I. Y.; Zhang, X.; Zhao, Y.; Brooks, B. R.; Chan, G. K. L.; Chipman, D. M.; Cramer, C. J.; Goddard, W. A., III; Gordon, M. S.; Hehre, W. J.; Klamt, A.; Schaefer, H. F., III; Schmidt, M. W.; Sherrill, C. D.; Truhlar, D. G.; Warshel, A.; Xu, X.; Aspuru-Guzik, A.; Baer, R.; Bell, A. T.; Besley, N. A.; Chai, J.-D.; Dreuw, A.; Dunietz, B. D.; Furlani, T. R.; Gwaltney, S. R.; Hsu, C.-P.; Jung, Y.; Kong, J.; Lambrecht, D. S.; Liang, W.; Ochsenfeld, C.; Rassolov, V. A.; Slipchenko, L. V.; Subotnik, J. E.; Van Voorhis, T.; Herbert, J. M.; Krylov, A. I.; Gill, P. M. W.; Head-Gordon, M. Advances in Molecular Quantum Chemistry Contained in the Q-Chem 4 Program Package. *Molecular Physics* **2014**, *113*, 184–215.

10. Gaussian 16, Revision A.03, Frisch, M. J.; Trucks, G. W.; Schlegel, H. B.; Scuseria, G. E.; Robb, M. A.; Cheeseman, J. R.; Scalmani, G.; Barone, V.; Petersson, G. A.; Nakatsuji, H.; Li, X.; Caricato, M.; Marenich, A. V.; Bloino, J.; Janesko, B. G.; Gomperts, R.; Mennucci, B.; Hratchian, H. P.; Ortiz, J. V.; Izmaylov, A. F.; Sonnenberg, J. L.; Williams-Young, D.; Ding, F.; Lipparini, F.; Egidi, F.; Goings, J.; Peng, B.; Petrone, A.; Henderson, T.; Ranasinghe, D.; Zakrzewski, V. G.; Gao, J.; Rega, N.; Zheng, G.; Liang, W.; Hada, M.; Ehara, M.; Toyota, K.; Fukuda, R.; Hasegawa, J.; Ishida, M.; Nakajima, T.; Honda, Y.; Kitao, O.; Nakai, H.; Vreven, T.; Throssell, K.; Montgomery, J. A., Jr.; Peralta, J. E.; Ogliaro, F.; Bearpark, M. J.; Heyd, J. J.; Brothers, E. N.; Kudin, K. N.; Staroverov, V. N.; Keith, T. A.; Kobayashi, R.; Normand, J.; Raghavachari, K.; Rendell, A. P.; Burant, J. C.; Iyengar, S. S.; Tomasi, J.; Cossi, M.; Millam, J. M.; Klene, M.; Adamo, C.; Cammi, R.; Ochterski, J. W.; Martin, R. L.; Morokuma, K.; Farkas, O.; Foresman, J. B.; Fox, D. J. Gaussian, Inc., Wallingford CT, 2016.
